# Urinary Metabolomics for the Prediction of Radiation-Induced Cardiac Dysfunction

**DOI:** 10.3390/metabo13040525

**Published:** 2023-04-06

**Authors:** Yaoxiang Li, Shivani Bansal, Vijayalakshmi Sridharan, Sunil Bansal, Meth M. Jayatilake, Jose A. Fernández, John H. Griffin, Marjan Boerma, Amrita K. Cheema

**Affiliations:** 1 Department of Oncology, Lombardi Comprehensive Cancer Centre, Georgetown University Medical Center, Washington, DC 20057, USA; 2 Departments of Biochemistry, Molecular, and Cellular Biology, Georgetown University Medical Center, Washington, DC 20057, USA; 3 Division of Radiation Health, Department of Pharmaceutical Sciences, 4301 West Markham #522-10, University of Arkansas for Medical Sciences, Little Rock, AR 72205, USA; 4 Department of Molecular Medicine, Scripps Research Institute, La Jolla, CA 92037, USA

**Keywords:** metabolomics, radiation-induced cardiac dysfunction, non-invasive indicators, mouse model, urinary metabolites, activated protein C (APCHi), ionizing radiation

## Abstract

Survivors of acute radiation exposure are likely to experience delayed effects that manifest as injury in late-responding organs such as the heart. Non-invasive indicators of radiation-induced cardiac dysfunction are important in the prediction and diagnosis of this disease. In this study, we aimed to identify urinary metabolites indicative of radiation-induced cardiac damage by analyzing previously collected urine samples from a published study. The samples were collected from male and female wild-type (C57BL/6N) and transgenic mice constitutively expressing activated protein C (APCHi), a circulating protein with potential cardiac protective properties, who were exposed to 9.5 Gy of γ-rays. We utilized LC-MS-based metabolomics and lipidomics for the analysis of urine samples collected at 24 h, 1 week, 1 month, 3 months, and 6 months post-irradiation. Radiation caused perturbations in the TCA cycle, glycosphingolipid metabolism, fatty acid oxidation, purine catabolism, and amino acid metabolites, which were more prominent in the wild-type (WT) mice compared to the APCHi mice, suggesting a differential response between the two genotypes. After combining the genotypes and sexes, we identified a multi-analyte urinary panel at early post-irradiation time points that predicted heart dysfunction using a logistic regression model with a discovery validation study design. These studies demonstrate the utility of a molecular phenotyping approach to develop a urinary biomarker panel predictive of the delayed effects of ionizing radia-tion. It is important to note that no live mice were used or assessed in this study; instead, we focused solely on analyzing previously collected urine samples.

## 1. Introduction

A complex cascade of molecular events is triggered as a consequence of exposure to ionizing radiation (IR), which impacts metabolic activities and various physiological functions. Acute radiation syndrome (ARS) usually manifests shortly after exposure as hematopoietic and gastrointestinal syndrome depending on the dose and type of radi-ation. Survivors of ARS are likely to experience delayed effects that can manifest as organ injury to vital organs such as the heart, brain, lungs, and kidneys (also known as the delayed effects of acute radiation exposure or DEARE) [1,2,3]. The degree and extent of tissue damage are heavily dependent on the dose, dose rate, and duration of exposure to IR. The time-dependent progression of organ injury remains asymptomatic in the latent period as such a molecular phenotyping approach is attractive for identifying initial molecular alterations that are predictive of organ dysfunction before the appearance of gross clinical symptoms [4,5,6,7,8,9].

Metabolomics is a promising approach to capturing radiation-induced metabolic perturbations and identifying early indicators of radiation injuries [10]. In this study, we leveraged a murine model of radiation exposure to identify bi-omarkers in urine that are predictive of cardiac dysfunction at early time points post-irradiation. Activated protein C (APC) is a vitamin-K-dependent natural protein in plasma that has anti-coagulant and anti-inflammatory properties and is known to improve endothelial function through the activation of several endothelial cell surface receptors [11]. APC has previously been shown to have potent radiation mitigative effects in a mouse model of ARS [12]. We used a transgenic mouse model that constitutively overexpresses APC, hence being termed APCHi mice [13,14] to show the sex-dependent effects of APC upregulation on radiation-induced cardiac dysfunction [15]. In this prior study, we found that for a single dose of 9.5 Gy γ-rays, with hind-leg shielding from radiation to allow long-term survival, the APCHi genotype had no effect on radiation-induced changes in cardiac function but caused a more severe increase in radiation-induced cardiac collagen deposition compared to the WT genotype in female mice, while protecting female mice from a radiation-induced decrease in cardiac microvascular density. In the current study, we used urine samples obtained from these same sham-irradiated and irradiated mice to determine whether differences in radiation-induced cardiac outcomes in APCHi mice are reflected in an altered metabolic response post-IR.

**Figure 1:**
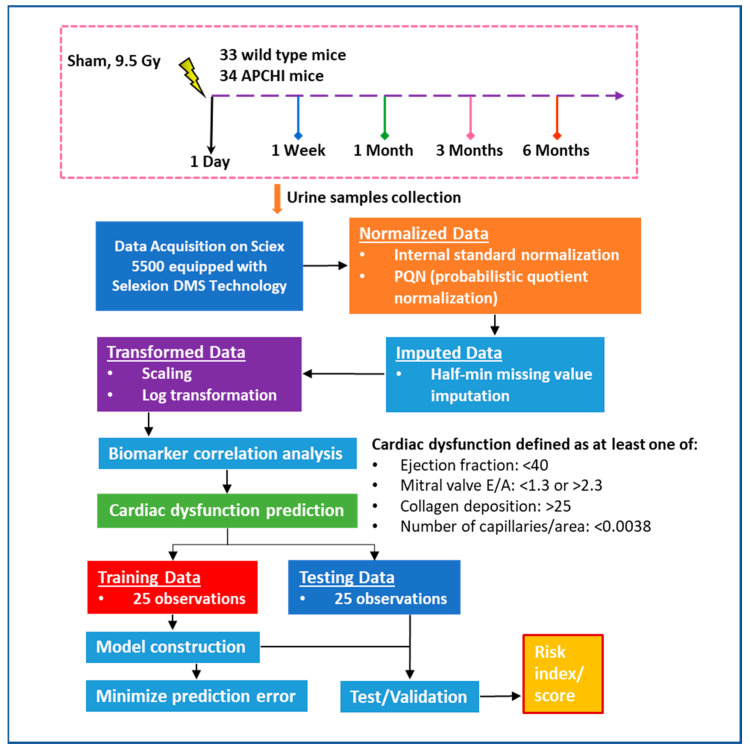
The flowchart outlines the framework of our metabolomics-based biomarker study aimed at predicting radiation-induced heart dysfunction. We utilized a cohort of 33 wild type mice and 34 APCHi mice, which included both irradiated and sham mice. Out of the 50 mice exposed to radiation and evaluated for cardiac dysfunction outcomes, 18 mice had normal results while 32 mice showed signs of cardiac dysfunction. Models were developed using a training dataset of 25 mice, and the test dataset of 25 mice was used to calculate the AUC and evaluate the risk score for radiation-induced heart injury.

In previous studies, we have demonstrated IR-induced perturbations in plasma, plasma-derived extra-cellular vesicles, and tissue samples using LC-MS-based metabolomics [16,17,18,19]. The present study was designed with several objectives: firstly to identify the metabolic consequences of irradiation and to identify the impact of genotype as well as sex on radiation response over time. Additionally, to assess the feasibility of using urine me-tabolites as predictive biomarkers, we performed correlative analyses of metabolomics data with heart dysfunction indicators (increased collagen deposition, reduced capil-lary density, reduced ejection fraction, and/or altered mitral valve E/A ratio) as deter-mined by histology and cardiac ultrasound at 6 months post-irradiation. We posit that a metabolomics-based molecular phenotyping approach could be used as a predictive tool to pre-emptively identify individuals at risk of radiation-induced organ injury. To our knowledge, this is the first study to develop a urine metabolomics-based prediction model for radiation-induced cardiac injury using a logistic regression model. The workflow of the study is illustrated in Figure 1.

## 2. Materials and Methods

### 2.1. Animal Procedures

Animal purchase, housing, irradiation, and assessment of cardiac function and histology were completed in a prior study [15]. All urine samples used in this study were collected from a previous publication, and no live mice were used or assessed. All animal work was performed at the University of Arkansas for Medical Sciences (UAMS) under approved IACUC protocols (#3763 and 3982). A detailed description of the methods has been provided in a previous publication [15].

Briefly, male and female WT or APCHi mice on a C57BL/6N background were subjected to partial body irradiation at a single dose of 9.5 Gy (cesium-137 source, Mark 1, Model 68A, JL Shepherd & Associates, San Fernando, CA, USA), with both hind legs shielded from radiation. Sham-irradiated mice received the same treatment but were not exposed to radiation. Dosimetry was performed with Gafchromic film (DOSE-MAP, Ashland Specialty Ingredients, Wayne, NJ, USA) and an ion chamber (Exradin A20, Standard Imaging, Middleton, WI, USA) and electrometer (X4000, Standard Imaging) that are calibrated for γ-rays once a year. Cardiac dysfunction was defined using previously published data based on the following criteria: mitral valve E/A ratio below 1.3 or above 2.3, ejection fraction ≤40, cardiac collagen content above 25%, or the number of myocardial capillaries/areas ≤0.0038 [15].

All urine samples were collected in the morning, between 8 a.m. and 10 a.m. Each mouse was carefully picked up and held vertically, and their abdomen was briefly stroked. In that position, the mice released urine, which was collected in an Eppendorf tube and immediately placed on ice. Within one hour of collection, the samples were centrifuged at 2700× *g* for 5 min and the supernatant was flash frozen and stored at −80 °C until metabolomics. Urine samples were collected at 5-time points: 24 h, 1 week, 1 month, 3 months, and 6 months post-irradiation in Eppendorf tubes, centrifuged and aliquoted. Urine samples were stored at −80 °C until LC-MS analysis.

### 2.2. Chemicals

All LC-MS grade solvents including acetonitrile and water were purchased from Fisher Optima grade, Fisher Scientific (Pittsburgh, PA, USA). High purity formic acid (99%) LC–MS grade methanol, dichloromethane, and ammonium acetate were purchased from Fisher Scientific and HPLC grade 1-propanol, debrisoquine, and 4-nitrobenzoic acid were purchased from Sigma-Aldrich (Saint Louis, MO, USA). Milli-Q water was obtained from an in-house Ultrapure Water System by EMD Millipore (Billerica, MA, USA). The Lipidyzer isotope-labeled internal standards mixture consisting of 54 isotopes from 13 lipid classes was purchased from SCIEX (Framingham, MA, USA).

### 2.3. Targeted Metabolomics Analysis

The targeted metabolomics method was developed in-house to quantitate 270 endogenous molecules using a QTRAP^®^ 5500 LC-MS/MS System (SCIEX). For this purpose, 10 µL of each urine sample was extracted in 50 μL of chilled isopropanol containing internal standards (debrisoquine 200 ng/mL for positive mode and taurine-d4 200 ng/mL for negative mode). The samples were vortexed for 1 min and kept on ice for 30 min. The samples were incubated at −20 °C for 2 h for complete protein precipitation. The samples were centrifuged at 13,000 rpm for 20 min at 4 °C. The supernatant was transferred to an MS vial for LC-MS analysis. Exactly 5 μL of the prepared sample was injected onto a Kinetex 2.6 μm 100 Å 100 × 2.1 mm (Phenomenex, CA, USA) using a SIL-30 AC autosampler (Shimazdu, Kyoto, Japan) connected with a high flow LC-30AD solvent delivery unit (Shimazdu) and CBM-20A communication bus module (Shimazdu) online with QTRAP 5500 (SCIEX) operating in positive and negative ion mode. A binary solvent comprising water with 0.1% formic acid (solvent A) and acetonitrile with 0.1% formic acid (solvent B) was used. The extracted metabolites were resolved at a 0.2 mL/min flow rate starting with 100% of solvent A and holding for 2.1 min and moving to 5% of solvent A over a time period of 12 min and holding for 1 min and equilibrating to initial conditions over a time period of 7 min using an autosampler temperature of 15 °C and an oven temperature 30 °C. The source and gas setting for the method were as follow: curtain gas  =  35, CAD gas  =  medium, ion spray voltage  =  2500 V in positive mode and −4500 V in negative mode, temperature  =  400 °C, nebulizing gas  =  60 and heater gas  =  70. The data were normalized to the internal standard area and processed using MultiQuant 3.0.3 (SCIEX). To ensure the high quality and reproducibility of the LC-MS data, the column was conditioned using pooled QC samples initially, and the pooled QC samples were also injected periodically (after every 10 sample injections) to monitor shifts in signal inten-sities and retention time.

### 2.4. Targeted Lipidomics Analysis

Lipids were extracted from 50 µL of urine using the modified Bligh-Dyer method [20]. The frozen urine samples were thawed at room temperature (25 °C) for 30 min and vortexed; 50 μL of each urine sample was transferred to a borosilicate glass culture tube (16 × 100 mm). Next, 950 μL of water, 2 mL of methanol, and 900 μL of dichloromethane were added to all of the samples. The mixture was vortexed for 5 s and 50 μL of the isotope-labeled internal standards mixture was added to the tube. Over 50 stable isotope labeled internal standards spanning all 13 lipid classes were added to each sample prior to extraction for accurate quantitation. The samples were left to incubate at room temperature for 30 min. Next, another 1 mL of water and 1 mL of dichloromethane were added to the tube, followed by gentle vortexing for 5 s, and centrifugation at 2500× *g* at 15 °C for 10 min. The bottom organic layer was transferred to a new tube and 1 mL of dichloromethane was added to the original tube for a second extraction. The combined extracts were concentrated under nitrogen and reconstituted in 250 μL of the running solution (10 mM ammonium acetate in 50:50 methanol: dichloromethane) and analyzed using DI-MS/MS with differential mobility spectrometry (DMS) separation. Quantitative lipidomics was performed on the Lipidyzer platform consisting of Shimadzu Nexera X2 LC-30AD pumps, a Shimadzu Nexera X2 SIL-30AC autosampler, and a QTRAP^®^ 5500 mass spectrometer equipped with SelexION^®^ for DMS (SCIEX). The samples were introduced to the mass spectrometer by flow injection analysis at 8 μL/minute. The urine samples were analyzed using the direct infusion-tandem mass spectrometry (DI-MS/MS) Lipidyzer platform (SCIEX). Lipid molecular species were measured using multiple reaction monitoring (MRM) and positive/negative polarity switching. MRM transitions for a total of 1070 lipids and fatty acids were targeted in the analysis. The data were acquired and processed using Multiquant 3.0.3 (SCIEX) and Lipidomics Workflow Manager 1.0.5.0 (SCIEX). The results provided the concentration (μM) and fatty acid composition (mol%) of total lipid classes as well as individual lipid species.

### 2.5. Data Processing and Statistical Analysis

A targeted LC-MS-based metabolomics and lipidomics approach was employed to quantify the abundance of urinary metabolites in both positive and negative modes. The metabolite intensity was normalized to internal standards and further processed using advanced data pre-processing techniques including log transformation and Pareto scaling. Differential expression of each metabolite was calculated using a rigorous statistical analysis approach, incorporating an unpaired t-test, and considering multiple confounding factors such as sex and genotype with a significance threshold of *p* < 0.05. To ensure the robustness and accuracy of the data, stringent quality control measures were implemented, including the application of a 20% coefficient of variation filter criteria [21] and missing value imputation techniques such as the half min algorithm. Additionally, analytical drift was effectively corrected using the quality control sample based robust LOESS (locally estimated scatterplot smoothing) signal correction (QC-RLSC) method, resulting in highly reliable and accurate data for the identification of biomarkers associated with radiation-induced cardiac dysfunction.

## 3. Results

### 3.1. Radiation Induces a Robust Metabolic Response in the Urine Samples of Mice in a Time-Dependent Manner

Two groups of mice were utilized in this metabolomics study. The first group consisted of C57BL6/N WT mice, with a total sample size of *n* = 33, stratified by sex with *n* = 20 males and *n* = 13 females. These mice were further divided into radiation-exposed (9.5 Gy γ-rays) and sham-treated subgroups. The second group consisted of transgenic APCHi mice, with a total sample size of *n* = 34, also stratified by sex with *n* = 17 males and *n* = 17 females. These mice were also divided into radiation-exposed (9.5 Gy) and sham-treated subgroups. Longitudinally collected urine samples—(24 h, 1 week, 1 month, 3 months, and 6 months post-irradiation) were subject to LC–MS-based targeted metabolomic and lipidomic analyses in order to delineate longitudinal metabolic alterations that accompany exposure to IR. The LC-MS data from the in-house developed targeted metabolomic panel was pre-processed using MultiQuant 3.0.3 software (SCIEX) which yielded 425 metabolites and lipid features that were selected for downstream statistical analyses. First, we assessed all of the mice, combining their genotypes and sex. The urine samples showed dysregulation of multiple metabolites and lipids at the early time point (24 h); however, most of these metabolic dysregulations stabilized at the later time points (Supplementary Table S1). Several metabolites showed oscillatory patterns of urinary abundance. For example, urinary levels of acetyl-CoA and ascorbate showed a significant increase at 24 h after irradiation but a decrease at 1 month and 3 months, suggesting early changes in energy metabolism and oxidative stress. We also observed a decline in the levels of cholesterol esters (CEs) and cysteine while the levels of triacylglycerols (TAGs), ceramide (16:0), sphingomyelin (26:1), diacylglycerol (DAG 18:1/22:6), hexosyl ceramide (HCER), and amino acid conjugates (O-Acetylserine, N-Glycyl-L-proline) were upregulated. At 6 months post-irradiation, we observed a decrease in CER (16:0), oxoglutarate, phenyl propionic acid, n-acetyl alanine, and TAG. These results suggest that metabolic shifts persist for months after irradiation.

### 3.2. Genotype Impacts the Response to Radiation Injury

**Figure 2:**
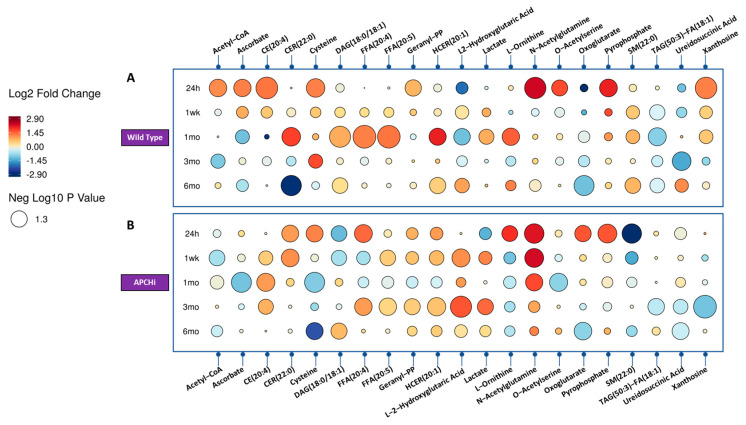
Raindrop illustration of metabolites altered in urine at different time points in Wild Type (Panel **A**) and APCHi (Panel **B**) mice. The colors of each circle represent fold change, with red representing an increase and blue representing a decrease. The size of the circle represents the level of statistical significance, with larger circles indicating greater statistical significance.

Next, we asked if radiation response was modulated by genotype; hence we com-pared the urinary metabolic profiles of the sham and irradiated C57BL6N and APCHi mice separately (Supplementary Tables S2 and S3). The results indicate that there is a clear difference in radiation response between WT and APCHi mice at the metabolic level over time. For example, there was a decrease in the levels of certain compounds such as TAGs and an increase in the levels of other compounds such as CE, betaine, pyrophosphate, acetyl-CoA, orotate, pyridoxamine, xanthosine, ascorbate, and amino acid/conjugates (cysteine, aminoadipate, o-acetylserine, N-acetyl glutamine, and 3-methylamino-l-alanine) at 24 h post-irradiation in the WT mice, many of which were persistent throughout the 6-month follow-up period of the study. In contrast, APCHi mice showed fewer metabolic perturbations at this time point, suggesting they have better recovery mechanisms. This is illustrated in the raindrop plot (Figure 2) of temporal changes in the metabolic response of both genotypes. The differences between WT and APCHi mice seen at 24 h but also later time points do not exclude the possibility that APCHi mice are more resistant to radiation and/or have better recovery mechanisms.

### 3.3. Sex Impacts the Response to Radiation Injury

**Figure 3:**
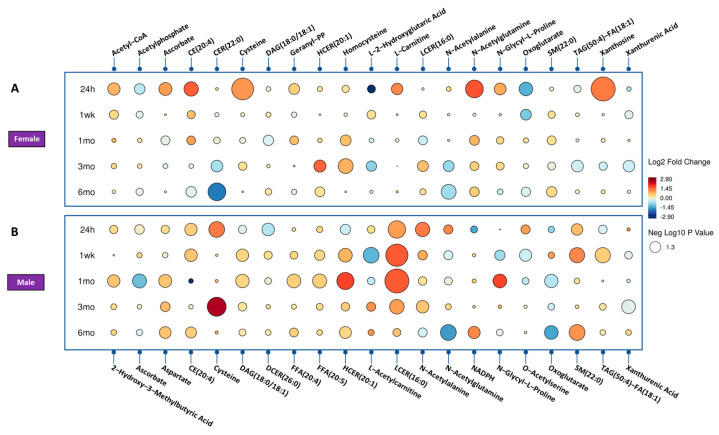
Radiation effect of mice for significantly dysregulated biomarkers across time. (Panel **A**) showing the female mice radiation response and (Panel **B**) shows the male mice radiation effect. Each circle in the figure denotes a fold change, where red indicates an increase and blue indicates a decrease. The size of each circle corresponds to the level of statistical significance, with larger circles indicating greater statistical significance.

**Figure 4:**
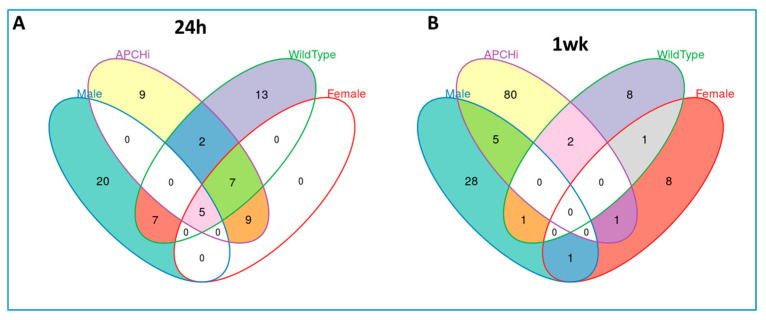
Venn diagram detailing the unique biomarkers for radiation response specific to each group comparing radiation versus sham dependent and independent of sex and genotype, analyzed by targeted mass spectrometry. (Panel **A**). 24 h’ post-irradiation, (Panel **B**). 1 week after radiation.

Next, we sought to investigate the effect of radiation on urinary metabolic profiles considering sex as a biological variable. A Venn diagram detailing the unique biomarkers of radiation response specific to each group, comparing radiation to sham, dependent and independent of sex and genotype, 24 h and 1 week after irradiation, is shown in Figure 3 and Figure 4.

In the female mice, we observed upregulation of acetyl-CoA and pyrophosphate, key intermediates in the citric acid cycle or TCA cycle. Furthermore, we observed a downregulation of acetylphosphate and oxoglutarate, indicating a decrease in energy production, and changes in the levels of lipids such as DAG and a decrease in others such as N-acetylornithine and TAG.

In contrast, in the male mice, we observed a decrease in methylphenylacetate levels at 24 h post-irradiation, together with an increase in LCER (16:0) and several amino acids and conjugates. After one month, we observed an increase in ascorbate, CE, indole-3-carboxylic acid, cysteine, N-acetylglutamine, and oxoglutarate. At the 3-month time point, we observed an increase in ADP, LCER, and cysteine levels.

These findings suggest that the energy metabolism pathways and amino acid metabolism pathways are affected differently between female and male mice upon irradiation. The upregulation of TCA cycle intermediates in the female mice suggests increased energy production and the increase in certain amino acids and conjugates, such as cysteine, suggests increased protein synthesis and repair.

### 3.4. Prediction Model for Radiation-Induced Cardiac Dysfunction

We used the urinary metabolomics data to determine whether urine metabolite profiles may serve as predictors of late radiation effects in the heart. For all the mice included in this study, at 6 months after irradiation, cardiac ultrasound and histological staining were used to measure ejection fraction, mitral valve E/A, cardiac collagen deposition, and myocardial capillary density. Those results were published previously [15]. We defined heart dysfunction if any one of the following conditions was satisfied: (1) mitral valve E/A below 1.3 or above 2.3 (for this parameter, both low and high extreme values are an indication of cardiac dysfunction); (2) ejection fraction ≤40; (3) cardiac collagen content above 25% as an indicator of myocardial radiation fibrosis; or (4) the number of myocardial capillaries/areas ≤0.0038, a common manifestation of adverse myocardial remodeling in the irradiated heart [22]. Test statistics for heart dysfunction were determined as a binary outcome (yes vs. no) (Supplementary Table S4).

A logistic regression model was developed to relate urinary metabolite profiles at 1 week and 1 month after irradiation to heart dysfunction (yes or no) at 6 months. To focus on the effect of radiation exposure, we combined the both genotypes and the sex of the mice. Therefore, our study included a cohort of 50 mice who were exposed to ra-diation and had their cardiac dysfunction outcomes measured. Among them, 18 mice showed normal results while 32 mice displayed signs of cardiac dysfunction. The data were split into a training and testing set in a 1:1 ratio.

**Figure 5:**
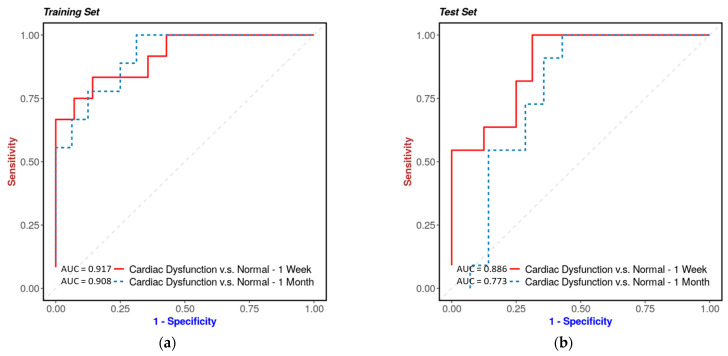
Urinary metabolite predictors of cardiac dysfunction. The prediction of cardiac dysfunction using urinary metabolite predictors developed through logistic regression modeling. A cohort of 50 mice, including 33 wild-type and 34 APCHi mice exposed to radiation, had their cardiac dysfunction outcomes measured, with 18 mice showing normal results and 32 mice displaying signs of cardiac dysfunction. The data were split into a 1:1 ratio for training and testing sets. The 1-week model panel included SM(22:0), homocysteine, TAG56:7-FA20:4, FFA(16:1), and SM(24:0), while the 1-month model panel consisted of anthranilic acid, TAG54:4-FA16:0, CE(22:6), spermidine, and mesaconic acid. Cross-validation was used to calculate true positive rates and false positive rates, and a ROC curve for training set (Panel **a**) and testing set (Panel **b**) was drawn to estimate the model’s performance.

The logistic regression model demonstrated high predictive accuracy for heart dysfunction with an area under the curve (AUC) greater than 0.8 using a test valida-tion study design. Cross-validation was conducted to calculate true positive rates and false positive rates, and a receiver operating characteristic (ROC) curve was constructed to estimate the model’s performance (Figure 5a,b). The models were trained and tested using a discovery set, resulting in an AUC of 0.917 and 0.908 for the 1-week and 1-month models, respectively. These models were then used to generate risk scores, which were calculated as linear combinations of the concentrations of the selected metabolites. The performance of these models was evaluated on subsets of 1-week and 1-month post-irradiation data, with AUC values indicating high predictive accuracy for heart dysfunction.

The five metabolites that were found to fit the regression model (Table 1) at 1 week (SM (22:0), homocysteine, TAG56:7-FA20:4, palmitoleic acid (FFA C16:1), and SM (24:0)) and the 1-month model (anthranilic acid, TAG54:4-FA16:0, CE (22:6), spermidine, and mesaconic acid) are involved in the structural integrity of the cell membrane, inflammation, energy production, and cellular growth regulation and could have implications for heart function or injury. However, it is important to note that the mechanistic role of these metabolites should be further validated with more research.

## 4. Discussion

Exposure to non-lethal doses of IR can trigger complex cellular responses, leading to alterations in metabolomic profiles that are dependent on the type and dose of radiation. This occurs in part, due to the production of reactive oxygen species that interact with cellular components such as proteins and lipids, leading to inflammatory and cellular repair responses [23,24,25,26,27,28].

The availability of urinary biomarkers for predicting radiation-induced cardiac injury has important clinical implications. Urine collection is a non-invasive procedure that is ideal for delineating individuals at risk of radiation-induced organ injury in a radiological scenario. Urine tests, are routinely used in the clinic to diagnose an array of patho-physiologies including urinary tract infections, kidney disease, and bladder cancer among others. Our findings highlight the potential of using urinary metabolomic biomarkers to identify patients at risk of radiation-induced cardiac injury, allowing for early intervention and personalized treatment strategies. Understanding the biological effects of radiation exposure is especially important in today’s world due to a variety of factors such as the increased use of radiation in medical treatments, occupational exposure, and the potential risk of radiological incidents or accidents [29,30,31]. The discovery of urinary biomarkers for radiation-induced cardiac injury has the potential to improve the safety and efficacy of radiation therapy, improve radiation protection for nuclear industry workers, and contribute to the development of countermeasures in the event of a radiological emergency. Furthermore, research into radiation-induced injury has far-reaching implications for understanding the mechanisms of tissue damage and repair, as well as identifying novel therapeutic targets for a variety of diseases.

Urinary metabolomics has evolved as one of the most promising biomarker plat-forms since urine is one of the most accessible fluids and has a quite stable matrix compared to other bio-fluids [32]. Lipidomic analysis of urine may provide further insights into our understanding of various biological processes, particularly those related to cardiovascular disease. Lipidomics enables the in-depth investigation of lipids, which are essential cellular components that act as signaling molecules, energy sources, and structural building blocks. Lipidomic analysis in the context of cardiovascular disease can help identify specific lipid signatures, providing insights into disease mechanisms, progression, and potential therapeutic targets. Herein, we collected urine samples at various time points and used LC-MS-based targeted metabolomic and lipidomic analyses to identify longitudinal metabolic changes that follow exposure to IR. Our working hypothesis was that urinary metabolite profiles may depend on sex, and genotype and may predict the development of cardiac radiation injury. At 6 months post-irradiation in WT mice, a persistent decrease in certain ceramides, oxoglutarate, phenyl propionic acid, n-acetyl alanine, and TAGs was observed, indicating that metabolic shifts persist months after the initial exposure to radiation and can lead to the development of late radiation toxicities to tissues or organs.

While plasma APC levels were not measured in this study, our study also found that genotype plays a significant role in determining the response to radiation injury. The irradiated WT mice showed persistent changes in several metabolites, including those associated with fatty acid metabolism. Upregulation of free fatty acids may contribute to inflammation and oxidative stress, which are known to play a role in both radiation toxicity, oxidative stress [33], and cardiac dysfunction [34]. Similarly, changes in SM and ceramides may reflect an effect of IR on cell membrane function [35]. On the other hand, the APCHi mice exhibited fewer pronounced metabolic perturbations, suggesting that the APCHi mice are more resilient to IR injury and have better recovery mechanisms.

Our analysis also revealed that the response to IR is affected by sex. Specifically, the upregulation of L-carnitine observed only in female mice at 24 h post-IR might have been related to estrogen levels [36]. On the other hand, dysregulation of L-acetyl carnitine was specific to male mice. Carnitines are involved in the mitochondrial oxidation of fatty acids, and radiation-induced dysregulation in carnitines can perturb mitochondrial function [37,38,39]. The downregulation of these carnitines may indicate a decrease in mitochondrial function or accelerated aging [37,40,41]. Homocysteine was observed as a specifically perturbed metabolite in female mice at the 1-month time point. Homocysteine has been implicated in oxidative stress and has been found to correlate with cardiovascular disease [42,43,44]. Altogether, our findings suggest that sex plays a significant role in the response to IR. Further research is needed to understand the underlying mechanisms of these sex differences.

Currently, only a limited number of radiological and clinical parameters are used to identify IR-exposed individuals at high risk of heart abnormalities, and novel approaches for early detection and prediction are needed. We developed a statistical regression model to relate urine metabolomics data (1 week and 1-month post-IR) to late outcomes of cardiac dysfunction (6 months).

One of the primary goals of this study was to identify a urinary metabolomic pattern that could predict late radiation-induced cardiac changes. We used logistic regression as our prediction model to accomplish this. Logistic regression is a popular statistical technique for predicting the relationship between a binary outcome (in this case, the presence or absence of radiation-induced cardiac dysfunction) and a set of predictor variables (urinary metabolites in our study). This method was chosen for its ease of use, interpretability, and robustness in dealing with potential confounding factors such as genotype and sex. Furthermore, by incorporating variable selection techniques such as stepwise regression or LASSO, logistic regression allows us to identify the most significant predictor variables (metabolites), allowing us to create a parsimonious and easily interpretable model.

Logistic regression has several advantages over other prediction models found in the literature. In some cases, machine learning techniques such as support vector machines (SVM), random forests, or deep learning approaches, for example, may provide better predictive performance. However, these methods frequently necessitate larger sample sizes, are more computationally intensive, and can be less interpretable, making understanding the underlying biological mechanisms difficult. Furthermore, the use of these methods may result in overfitting, especially when dealing with a large number of predictor variables in comparison to the number of samples. In our study, logistic regression provided an appropriate balance of model complexity, interpretability, and predictive performance.

The prediction model was based on five markers with high sensitivity, specificity, and AUC. The pathway analysis showed that several of these metabolites are closely linked with fatty acid oxidation and mitochondrial metabolism. Our panel also contained TAGs, the main energy storage resource in mammals, with a central role in the balance of lipid handling and signaling mechanisms. TAG dynamics are profoundly altered in both pathologically stressed hearts and diabetics [45]. In addition, spermidine was identified, a polyamine essential for the proper function of many metabolic processes and cellular function, including the activation of autophagy, DNA stability, transcription, translation and apoptosis [46]. Mesaconic acid, a biomarker of isovaleric academia may have been formed from the metabolism of methylsuccinic acid in urine [47]. Lastly, palmitoleic acid was included in the 1-week prediction model. Palmitoleic acid (FFA C16:1) plays a role in cell functions such as growth and proliferation but also endoplasmic reticulum stress [48]. Palmitoleic acid is also postulated to have anti-thrombotic effects [49]. Exposure to 9.5 Gy of X-rays resulted in only mild signs of cardiac toxicity, which is one limitation of our study. In order to better understand the full spectrum of effects, future research should include multiple radiation doses. Further studies with large animal models such as non-human primates (NHPs) as well as clinical cohort studies are needed to test potential for clinical translation. However, the findings from our study provide a proof of concept of using a molecular phenotyping approach for preemptively identifying at-risk individuals as a ground-work for future clinical trials and to further investigate the implications of this study.

## 5. Conclusions

In conclusion, our study demonstrates longitudinal genotype and sex specific changes in urine metabolites after exposure to IR and shows the potential of utilizing metabolomics as a high-throughput, cost-effective approach for biomarker discovery to predict radiation-induced heart dysfunction. The lipidomic analysis presented in this study has provided important insights into the role of lipid metabolism in predicting radiation-induced cardiac injury. Further validation studies with larger sample sizes will be essential in developing a robust biomarker panel for early prediction of cardiac dysfunction and radiation injury. The methods for early prediction of cardiac dysfunction presented here may also be incorporated into future research involving the combination of radiomics and other “omics-approaches” for improved accuracy.

## Figures and Tables

**Table 1:** Fold Changes and Test Statistics of Key Metabolites in the 1-Week and 1-Month Regression Models.

1 Week Regression Model				
Name	*p*-Value	FDR	Fold Change	Log2(FC)
FFA(16:1)	0.00031	0.067	0.493	−1.020
Homocysteine	0.00083	0.081	0.375	−1.415
TAG56:7-FA20:4	0.00114	0.081	1.883	0.913
SM(24:0)	0.00056	0.079	2.385	1.254
SM(22:0)	0.00003	0.011	2.314	1.211
**1 Month Regression Model**				
**Name**	***p*-Value**	**FDR**	**Fold Change**	**Log2(FC)**
TAG54:4-FA16:0	0.00077	0.327	2.094	1.067
Spermidine	0.00709	0.511	0.670	−0.578
CE(22:6)	0.00929	0.511	2.981	1.576
Anthranilic acid	0.03527	0.511	0.660	−0.601
Mesaconic acid	0.22154	0.595	1.392	0.477

## Data Availability

All data generated or analyzed during this study are included in this published article (and its supplementary information files).

## Supplementary Materials

The following supporting information can be downloaded at: https://www.mdpi.com/article/10.3390/metabo13040525/s1, Table S1: List of dysregulated metabolites following exposure to 9.5 Gy of Gamma-radiation, 24 h, 1 week, 1 month, 3 month and 6 months post-irradiation. Table S2A: List of dysregulated metabolites for female, male, Wild type and APCHi mice following exposure to 9.5 Gy of Gamma-radiation, 24 h post-irradiation showing penotype dependent response of radiation. Table S2B: List of dysregulated metabolites for female, male, Wild type and APCHi mice following exposure to 9.5 Gy of Gamma-radiation, 1 week post-irradiation showing penotype dependent response of radiation. Table S2C: List of dysregulated metabolites for female, male, Wild type and APCHi mice following exposure to 9.5 Gy of Gamma-radiation, 1 month post-irradiation showing penotype dependent response of radiation. Supplementary Table S2D: List of dysregulated metabolites for female, male, Wild type and APCHi mice following exposure to 9.5 Gy of Gamma-radiation, 3 months post-irradiation showing penotype dependent response of radiation. Supplementary Table S2E: List of dysregulated metabolites for female, male, Wild type and APCHi mice following exposure to 9.5 Gy of Gamma-radiation, 6 months post-irradiation showing penotype dependent response of radiation. Supplementary Table S3A: List of dysregulated metabolites for female mice following exposure to 9.5 Gy of Gamma-radiation, 24 h, 1 week, 1 month, 3 month and 6 months post-irradiation. Supplementary Table S3B: List of dysregulated metabolites for male mice following exposure to 9.5 Gy of Gamma-radiation, 24 h, 1 week, 1 month, 3 month and 6 months post-irradiation. Supplementary Table S3C: List of dysregulated metabolites for wildtype mice following exposure to 9.5 Gy of Gamma-radiation, 24 h, 1 week, 1 month, 3 month and 6 months post-irradiation. Table S3D: List of dysregulated metabolites for APCHi mice following exposure to 9.5 Gy of Gamma-radiation, 24 h, 1 week, 1 month, 3 month and 6 months post-irradiation. Supplementary Table S4: List of metabolites showing co-relation with heart dysfuction following 9.5 Gy of Gamma-radiation at 1 week and 1 month post-irradiation.
