# Supplementary material for: Urinary Metabolomics for the Prediction of Radiation-Induced Cardiac Dysfunction
Source: Metabolites. 2023 Apr 6;13(4):525. doi: 10.3390/metabo13040525 (PMC10146652; doi:10.3390/metabo13040525)
Supplement: Supplementary file 1 [file metabolites-13-00525-s001.zip › metabolites-2306312-supplementary.pdf]

Urinary metabolites are predictive of radiation induced cardiac dysfunction in mice

[$]Yaoxiang Li,[1] [$]Shivani Bansal,[1] Vijayalakshmi Sridharan,[2] Michael Girgis,[1] Sunil Bansal,[1] Meth Jayatilake,[1] Jose A. Fernández[3], John H. Griffin[3], Marjan Boerma,[2] Amrita K Cheema[1,4 *]

[1]Department of Oncology, Lombardi Comprehensive Cancer Centre, Georgetown University Medical Center, Washington D.C, 20057, USA.

[2]Division of Radiation Health, Department of Pharmaceutical Sciences, 4301 West Markham #522-10, University of Arkansas for Medical Sciences, Little Rock, Arkansas, 72205, USA.

[3]Department of Molecular Medicine, Scripps Research Institute, La Jolla, CA 92037, USA.

[4]Departments of Biochemistry, Molecular and Cellular Biology, Georgetown University Medical Center, Washington D.C, 20057, USA.

$: Both authors contributed equally

*Correspondence: Amrita K Cheema Professor; Georgetown University Medical Center, GC2 Pre-Clinical Science Building, 3900 Reservoir Road NW Washington DC 20057; Phone (202) 687-2756; Fax (202) 687-8860; e-mail: akc27@georgetown.edu

Supplementary Table S1. List of dysregulated metabolites following exposure to 9.5 Gy of Gamma-radiation, 24 h, 1 week, 1 month, 3 month and 6 months post-irradiation.

	9.5Gy / 0.0Gy				9.5Gy / 0.0Gy				9.5Gy / 0.0Gy				9.5Gy / 0.0Gy				9.5Gy / 0.0Gy			
	24h				1wk				1mo				3mo				6mo			
									All											
name	p-value	FDR	FC	LOG2FC	p-value	FDR	FC	LOG2FC	p-value	FDR	FC	LOG2FC	p-value	FDR	FC	LOG2FC	p-value	FDR	FC	LOG2FC
METHYL PHENYLACETATE_neg_3	1.52E-05	0.006471	0.36516 ↓	-1.453	0.010049	0.28471	0.5543 ↓	-0.851	0.11365	0.76666	1.5431 ↑	0.6258	0.31777	0.65459	1.4505 ↑	0.5366	0.15543	0.39557	0.87418 ↓	-0.194
CE204	6.68E-05	0.014205	2.4885 ↑	1.3153	0.005893	0.26577	1.7554 ↑	0.8118	0.60827	0.91609	0.32993 ↓	-1.6	0.55675	0.81895	0.91942 ↓	-0.121	0.88338	0.94759	0.91564 ↓	-0.127
CE226	0.000139	0.019712	2.1312 ↑	1.0917	0.32033	0.7222	1.3484 ↑	0.4313	0.39096	0.89952	0.55925 ↓	-0.838	0.001711	0.18171	2.1944 ↑	1.1338	0.11832	0.34075	1.3832 ↑	0.468
CYSTEINE_neg_5	0.000333	0.035381	2.5263 ↑	1.337	0.80777	0.9346	1.0471 ↑	0.0664	0.7155	0.92337	1.1578 ↑	0.2114	0.002319	0.18171	2.5899 ↑	1.3729	0.65851	0.83047	1.0464 ↑	0.0655
XANTHOSINE_neg_1	0.000512	0.037313	2.2773 ↑	1.1873	0.098354	0.46573	1.3964 ↑	0.4817	0.26167	0.86881	1.2117 ↑	0.277	0.607	0.834	0.75255 ↓	-0.41	0.68022	0.83692	1.0422 ↑	0.0596
TAG567-FA226	0.000527	0.037313	2.2403 ↑	1.1637	0.56746	0.81005	0.76066 ↓	-0.395	0.86929	0.95219	0.67309 ↓	-0.571	0.22362	0.57251	0.74352 ↓	-0.428	0.82413	0.92273	0.77271 ↓	-0.372
TAG501-FA181	0.001294	0.067873	1.2809 ↑	0.3571	0.3286	0.72737	0.90404 ↓	-0.146	0.69491	0.91799	0.9504 ↓	-0.073	0.3681	0.70986	0.90983 ↓	-0.136	0.01093	0.085834	0.77433 ↓	-0.369
PYROPHOSPHATE_neg_3	0.001347	0.067873	3.072 ↑	1.6192	0.25119	0.67141	2.8461 ↑	1.509	0.084168	0.67934	2.0401 ↑	1.0286	0.94052	0.98253	1.3193 ↑	0.3998	0.53954	0.74208	1.2257 ↑	0.2937
NORMETANEPHRINE_pos_1	0.001437	0.067873	2.2184 ↑	1.1495	0.094518	0.46158	2.1339 ↑	1.0935	0.52561	0.91257	0.89827 ↓	-0.155	0.28362	0.62132	1.9391 ↑	0.9554	0.58218	0.76801	1.2425 ↑	0.3132
LCER160	0.002228	0.091932	3.0365 ↑	1.6024	0.00331	0.26577	3.6881 ↑	1.8829	0.003756	0.38156	3.155 ↑	1.6577	0.027806	0.29511	2.133 ↑	1.0929	0.092174	0.30193	1.8541 ↑	0.8907
N-ACETYLGLUTAMINE_pos_2	0.002379	0.091932	3.507 ↑	1.8102	0.32159	0.7222	0.79744 ↓	-0.327	0.85769	0.9468	1.378 ↑	0.4626	0.15373	0.52981	1.5321 ↑	0.6155	0.39178	0.63311	0.99578 ↓	-0.006
TAG544-FA224	0.003229	0.11435	1.4483 ↑	0.5344	0.2409	0.66054	1.4613 ↑	0.5472	0.79606	0.93191	0.79835 ↓	-0.325	0.49788	0.78662	1.1982 ↑	0.2608	0.41962	0.66051	1.1008 ↑	0.1386
TAG525-FA181	0.003554	0.11619	0.77318 ↓	-0.371	0.054973	0.34545	1.1776 ↑	0.2358	0.43838	0.90445	0.87898 ↓	-0.186	0.20355	0.56099	0.89871 ↓	-0.154	0.69195	0.84616	0.96616 ↓	-0.05
3-METHYLAMINO-L-ALANINE_pos_2	0.003992	0.12119	2.543 ↑	1.3465	0.3134	0.7222	1.2643 ↑	0.3384	0.11334	0.76666	0.75902 ↓	-0.398	0.68372	0.85718	0.84485 ↓	-0.243	0.50201	0.7049	1.3647 ↑	0.4486
OROTATE_neg_1	0.005409	0.15045	2.0278 ↑	1.0199	0.3572	0.74053	0.88827 ↓	-0.171	0.54617	0.91609	1.0161 ↑	0.0231	0.58733	0.82778	0.78814 ↓	-0.343	0.97811	0.99159	1.4089 ↑	0.4946
AMINOADIPATE_pos_1	0.005664	0.15045	1.8557 ↑	0.8919	0.30227	0.70975	0.63051 ↓	-0.665	0.37599	0.89952	0.81115 ↓	-0.302	0.43534	0.7494	2.1888 ↑	1.1301	0.022631	0.13547	0.75435 ↓	-0.407
TAG504-FA161	0.006913	0.17282	0.79817 ↓	-0.323	0.091123	0.45032	0.76128 ↓	-0.394	0.073631	0.67934	0.72322 ↓	-0.468	0.19738	0.55631	0.55407 ↓	-0.852	0.16913	0.40638	0.68261 ↓	-0.551
N-ACETYLALANINE_neg_1	0.007867	0.18574	2.3326 ↑	1.2219	0.31908	0.7222	1.4373 ↑	0.5234	0.296	0.8936	1.074 ↑	0.103	0.94772	0.98367	0.701 ↓	-0.513	0.003295	0.047473	0.62369 ↓	-0.681
TAG522-FA202	0.008967	0.20057	1.3924 ↑	0.4776	0.40145	0.77199	1.3638 ↑	0.4476	0.46583	0.90445	0.75419 ↓	-0.407	0.081061	0.38105	1.4307 ↑	0.5167	0.48852	0.69671	1.0421 ↑	0.0594
BETAINE_pos_2	0.010669	0.22672	2.7561 ↑	1.4626	0.81158	0.9346	1.0479 ↑	0.0675	0.16011	0.82488	0.68633 ↓	-0.543	0.75121	0.87952	0.80671 ↓	-0.31	0.51243	0.70939	1.6226 ↑	0.6983
TAG545-FA205	0.01197	0.2417	0.58021 ↓	-0.785	0.17903	0.61799	1.2434 ↑	0.3143	0.71957	0.92392	0.94405 ↓	-0.083	0.6699	0.85669	0.95736 ↓	-0.063	0.32918	0.58966	1.1992 ↑	0.2621
PHENYLPROPIOLIC ACID_neg_1	0.012512	0.2417	0.17366 ↓	-2.526	0.94846	0.96204	0.7499 ↓	-0.415	0.23785	0.85757	0.94538 ↓	-0.081	0.61029	0.834	0.7402 ↓	-0.434	0.032473	0.1643	0.60914 ↓	-0.715
ACETYL-COA_pos_1	0.015696	0.27472	2.068 ↑	1.0483	0.79595	0.9346	1.0622 ↑	0.087	0.25319	0.85757	1.6581 ↑	0.7295	0.86318	0.94947	1.2789 ↑	0.3549	0.8168	0.92273	0.92409 ↓	-0.114
TAG461-FA141	0.015901	0.27472	1.3787 ↑	0.4633	0.26354	0.67444	0.85035 ↓	-0.234	0.47133	0.90445	1.017 ↑	0.0243	0.49347	0.78255	1.0265 ↑	0.0377	0.061157	0.24233	1.3098 ↑	0.3893
TAG451-FA181	0.016758	0.27472	0.86617 ↓	-0.207	0.2008	0.61799	1.106 ↑	0.1454	0.65165	0.91609	0.99672 ↓	-0.005	0.057597	0.32639	1.1315 ↑	0.1783	0.1776	0.41934	1.0497 ↑	0.07
CE182	0.016806	0.27472	1.3859 ↑	0.4709	0.67414	0.87102	0.56993 ↓	-0.811	0.64545	0.91609	0.12007 ↓	-3.058	0.40255	0.73426	1.0714 ↑	0.0995	0.37903	0.62943	0.92 ↓	-0.12
GERANYL-PP_-HPO3_neg_2	0.017813	0.28039	1.6907 ↑	0.7577	0.67164	0.87102	1.4295 ↑	0.5156	0.5459	0.91609	1.2701 ↑	0.3449	0.71879	0.86769	0.94814 ↓	-0.077	0.70365	0.84685	1.1449 ↑	0.1953
INDOLE-3-CARBOXYLIC ACID_pos_3	0.018473	0.28039	3.1949 ↑	1.6758	0.14894	0.55976	1.5667 ↑	0.6478	0.9269	0.96978	1.4151 ↑	0.5009	0.62134	0.83832	1.005 ↑	0.0072	0.43074	0.66812	1.2747 ↑	0.3502
TAG533-FA182	0.021572	0.3119	0.74598 ↓	-0.423	0.90645	0.94333	0.93198 ↓	-0.102	0.40023	0.90445	0.89031 ↓	-0.168	0.012817	0.28126	0.73729 ↓	-0.44	0.031896	0.1643	0.62353 ↓	-0.681
TAG442-FA120	0.022443	0.3119	0.78967 ↓	-0.341	0.49262	0.79006	0.81237 ↓	-0.3	0.68285	0.91633	0.94568 ↓	-0.081	0.57463	0.82003	1.0706 ↑	0.0985	0.46957	0.68345	1.0493 ↑	0.0695
TAG442-FA182	0.02275	0.3119	0.7902 ↓	-0.34	0.19152	0.61799	1.1772 ↑	0.2353	0.064297	0.65022	1.4798 ↑	0.5654	0.62115	0.83832	1.107 ↑	0.1466	0.31568	0.5758	0.93905 ↓	-0.091
OXOGLUTARATE_neg_2	0.023798	0.3138	0.12961 ↓	-2.948	0.48136	0.789	0.38858 ↓	-1.364	0.033103	0.60889	0.83946 ↓	-0.252	0.27169	0.6072	0.83012 ↓	-0.269	0.003703	0.047687	0.50429 ↓	-0.988
HCER160	0.024365	0.3138	1.9527 ↑	0.9655	0.013828	0.29385	1.7717 ↑	0.8251	0.003439	0.38156	1.8713 ↑	0.9041	0.66979	0.85669	1.1821 ↑	0.2413	0.087316	0.29452	1.5266 ↑	0.6104
TAG521-FA161	0.026562	0.33202	1.2462 ↑	0.3175	0.861	0.94333	1.0806 ↑	0.1118	0.10088	0.73923	0.78192 ↓	-0.355	0.30721	0.65057	1.1569 ↑	0.2103	0.78061	0.89182	1.0761 ↑	0.1058
DAG181/226	0.031679	0.37336	1.3016 ↑	0.3802	0.025284	0.33123	1.2344 ↑	0.3038	0.23686	0.85757	1.1232 ↑	0.1676	0.27212	0.6072	1.1614 ↑	0.2159	0.053616	0.22123	1.215 ↑	0.2809
PYRIDOXAMINE_pos_1	0.032336	0.37336	1.5748 ↑	0.6552	0.51051	0.79092	0.79356 ↓	-0.334	0.33664	0.89952	2.3965 ↑	1.2609	0.44275	0.74968	0.70182 ↓	-0.511	0.46141	0.67752	1.2325 ↑	0.3016
TAG462-FA182	0.032504	0.37336	0.88479 ↓	-0.177	0.88186	0.94333	1.0158 ↑	0.0226	0.91909	0.96978	1.0319 ↑	0.0453	0.58821	0.82778	0.98152 ↓	-0.027	0.1463	0.38391	1.1805 ↑	0.2394
N-GLYCYL-L-PROLINE_pos_1	0.034231	0.37503	2.4701 ↑	1.3046	0.48714	0.79006	1.0285 ↑	0.0406	0.57551	0.91609	1.1221 ↑	0.1663	0.46165	0.76529	0.93281 ↓	-0.1	0.14384	0.38391	2.1903 ↑	1.1311
TAG567-FA180	0.034415	0.37503	0.60402 ↓	-0.727	0.21816	0.63228	1.1685 ↑	0.2247	0.5859	0.91609	0.92654 ↓	-0.11	0.27288	0.6072	0.93768 ↓	-0.093	0.98226	0.99159	0.9821 ↓	-0.026
TAG442-FA161	0.03547	0.37687	0.87329 ↓	-0.195	0.41923	0.77704	0.78635 ↓	-0.347	0.92509	0.96978	0.87991 ↓	-0.185	0.41766	0.74773	1.0914 ↑	0.1261	0.12237	0.34672	1.0881 ↑	0.1219
SM261	0.039928	0.41349	0.60246 ↓	-0.731	0.098625	0.46573	1.1383 ↑	0.1869	0.40744	0.90445	0.74318 ↓	-0.428	0.14962	0.52121	1.7701 ↑	0.8238	0.14356	0.38391	0.87185 ↓	-0.198
PIPECOLATE_pos_3	0.041561	0.41349	5.163 ↑	2.3682	0.75699	0.92624	1.1061 ↑	0.1455	0.042328	0.60889	0.84794 ↓	-0.238	0.2507	0.59631	0.80853 ↓	-0.307	0.74627	0.87416	1.2462 ↑	0.3176
IMIDAZOLE_pos_1	0.041835	0.41349	1.2779 ↑	0.3538	0.66817	0.87102	1.0259 ↑	0.037	0.028655	0.60889	1.735 ↑	0.795	0.7207	0.86769	1.1885 ↑	0.2491	0.5597	0.75958	1.1433 ↑	0.1932
CER160	0.045448	0.43316	1.3993 ↑	0.4847	0.038421	0.33653	0.52357 ↓	-0.934	0.5974	0.91609	0.92909 ↓	-0.106	0.029429	0.29511	0.47788 ↓	-1.065	0.001875	0.04577	0.29818 ↓	-1.746
TAG525-FA225	0.045864	0.43316	0.79798 ↓	-0.326	0.019326	0.30837	1.235 ↑	0.3045	0.74097	0.92465	1.0411 ↑	0.0581	0.94004	0.98253	0.94844 ↓	-0.076	0.5	0.7049	1.0672 ↑	0.0938
TAG565-FA225	0.047757	0.43847	0.73403 ↓	-0.446	0.15015	0.55976	1.1769 ↑	0.2349	0.9245	0.96978	1.0038 ↑	0.0054	0.46187	0.76529	1.0243 ↑	0.0346	0.030485	0.16195	1.1772 ↑	0.2354
ASCORBATE_neg_2	0.049074	0.43847	1.5833 ↑	0.663	0.65412	0.86323	1.3421 ↑	0.4245	0.002732	0.38156	0.52584 ↓	-0.927	0.95065	0.98367	0.86151 ↓	-0.215	0.28103	0.53083	0.78168 ↓	-0.355
O-ACETYLSERINE_pos_3	0.049521	0.43847	2.4496 ↑	1.2925	0.064062	0.37814	0.79758 ↓	-0.326	0.46514	0.90445	0.82101 ↓	-0.285	0.78278	0.90218	1.013 ↑	0.0187	0.93315	0.95908	1.1093 ↑	0.1497
CE205	0.055709	0.46275	0.7985 ↓	-0.325	0.007504	0.26577	1.7189 ↑	0.7815	0.76534	0.92465	0.41416 ↓	-1.272	0.30724	0.65057	1.1931 ↑	0.2547	0.83713	0.92651	0.94976 ↓	-0.074
TAG531-FA181	0.055735	0.46275	0.83743 ↓	-0.256	0.51363	0.79092	1.0509 ↑	0.0717	0.649	0.91609	0.99799 ↓	-0.003	0.13554	0.49662	0.90908 ↓	-0.138	0.035142	0.17367	0.77846 ↓	-0.361
TAG484-FA182	0.056074	0.46275	0.71193 ↓	-0.49	0.18346	0.61799	0.78013 ↓	-0.358	0.60324	0.91609	0.87157 ↓	-0.198	0.38701	0.71215	0.85868 ↓	-0.22	0.33021	0.58966	1.1667 ↑	0.2225
TAG483-FA141	0.056619	0.46275	0.85961 ↓	-0.218	0.34695	0.7364	0.85023 ↓	-0.234	0.47925	0.90445	0.85654 ↓	-0.223	0.18618	0.54197	0.89113 ↓	-0.166	0.98952	0.9942	0.98187 ↓	-0.026
N-ACETYLORNITHINE_neg_3	0.058192	0.46663	1.454 ↑	0.54	0.54507	0.80863	1.3406 ↑	0.4229	0.98843	0.99546	1.0791 ↑	0.1099	0.085257	0.38547	2.0639 ↑	1.0453	0.58875	0.76856	1.0118 ↑	0.0169
TAG483-FA140	0.062592	0.49051	0.67713 ↓	-0.563	0.013693	0.29385	0.63797 ↓	-0.648	0.63085	0.91609	0.764 ↓	-0.388	0.17148	0.53303	0.67757 ↓	-0.562	0.10876	0.33764	0.68817 ↓	-0.539
TAG564-FA181	0.063477	0.49051	0.82758 ↓	-0.273	0.88567	0.94333	0.7552 ↓	-0.405	0.038855	0.60889	0.60709 ↓	-0.72	0.034994	0.29777	0.36184 ↓	-1.467	0.16271	0.39958	0.3719 ↓	-1.427
TAG483-FA120	0.065199	0.4907	0.80911 ↓	-0.306	0.26349	0.67444	1.1172 ↑	0.1598	0.65497	0.91609	0.90142 ↓	-0.15	0.34791	0.69419	0.88168 ↓	-0.182	0.082947	0.28429	1.2199 ↑	0.2868
TAG524-FA204	0.065815	0.4907	0.82641 ↓	-0.275	0.042879	0.33653	1.3448 ↑	0.4274	0.14859	0.82488	1.277 ↑	0.3527	0.31373	0.65459	0.49711 ↓	-1.008	0.66222	0.83267	0.64751 ↓	-0.627
CE150	0.066966	0.4907	1.4044 ↑	0.4899	0.13426	0.53832	1.4016 ↑	0.4871	0.69551	0.91799	0.83409 ↓	-0.262	0.036061	0.30051	1.3672 ↑	0.4512	0.91374	0.95558	0.93485 ↓	-0.097
TAG514-FA182	0.070589	0.50848	0.83786 ↓	-0.255	0.26452	0.67444	0.84366 ↓	-0.245	0.49178	0.90479	0.96134 ↓	-0.057	0.067871	0.34753	0.73278 ↓	-0.449	0.10249	0.32507	0.66166 ↓	-0.596
TAG545-FA225	0.073685	0.52194	0.82491 ↓	-0.278	0.039834	0.33653	1.1884 ↑	0.249	0.54031	0.91609	1.042 ↑	0.0594	0.48954	0.78044	0.98948 ↓	-0.015	0.22736	0.47835	1.1273 ↑	0.1729
TAG483-FA182	0.076838	0.52356	0.91592 ↓	-0.127	0.099769	0.46595	0.84658 ↓	-0.24	0.61048	0.91609	0.87737 ↓	-0.189	0.7034	0.85997	0.93455 ↓	-0.098	0.012118	0.085834	0.87995 ↓	-0.185
TAG527-FA160	0.080096	0.52356	0.9086 ↓	-0.138	0.47104	0.789	1.0915 ↑	0.1263	0.64515	0.91609	0.90684 ↓	-0.141	0.72465	0.86999	0.99838 ↓	-0.002	0.65576	0.82946	1.0074 ↑	0.0106
ACETYLPHOSPHATE_pos_1	0.080158	0.52356	0.60185 ↓	-0.733	0.1862	0.61799	0.78769 ↓	-0.344	0.055693	0.65022	0.8242 ↓	-0.279	0.99876	0.99876	1.1418 ↑	0.1913	0.003163	0.047473	0.67395 ↓	-0.569
TAG462-FA120	0.081215	0.52356	0.78303 ↓	-0.353	0.32456	0.7222	0.85996 ↓	-0.218	0.30714	0.8936	1.0417 ↑	0.059	0.13555	0.49662	0.88311 ↓	-0.179	0.62808	0.80865	1.0197 ↑	0.0282
TAG483-FA181	0.08221	0.52356	0.87271 ↓	-0.196	0.51902	0.79092	0.94345 ↓	-0.084	0.92555	0.96978	0.9218 ↓	-0.117	0.67363	0.85669	0.97906 ↓	-0.031	0.54701	0.74753	1.0363 ↑	0.0514
TAG482-FA160	0.082218	0.52356	0.92777 ↓	-0.108	0.036428	0.33653	0.82063 ↓	-0.285	0.7382	0.92465	0.87126 ↓	-0.199	0.1428	0.51873	0.85628 ↓	-0.224	0.092355	0.30193	0.92174 ↓	-0.118
TAG531-FA180	0.082538	0.52356	0.90913 ↓	-0.137	0.24344	0.66021	1.0858 ↑	0.1188	0.50256	0.90566	0.91132 ↓	-0.134	0.80097	0.90846	1.0006 ↑	0.0009	0.009788	0.085834	0.83306 ↓	-0.264
N-ACETYLPUTRESCINE_neg_1	0.08854	0.54753	0.7033 ↓	-0.508	0.23487	0.64817	1.0911 ↑	0.1257	0.53655	0.91609	1.1834 ↑	0.2429	0.4271	0.7494	1.2735 ↑	0.3488	0.98141	0.99159	1.1498 ↑	0.2014
TAG543-FA183	0.090106	0.54753	1.2111 ↑	0.2764	0.50923	0.79092	1.3208 ↑	0.4014	0.50833	0.90566	0.6818 ↓	-0.553	0.42843	0.7494	1.1401 ↑	0.1891	0.99566	0.99801	0.99076 ↓	-0.013
TAG504-FA181	0.090182	0.54753	0.82541 ↓	-0.277	0.039385	0.33653	1.283 ↑	0.3595	0.93172	0.96978	0.94668 ↓	-0.079	0.23992	0.59631	0.85062 ↓	-0.233	0.92998	0.95908	0.97336 ↓	-0.039
HOMOCYSTEINE_pos_2	0.091989	0.55064	1.552 ↑	0.6342	0.40359	0.77199	0.6991 ↓	-0.516	0.30273	0.8936	0.89041 ↓	-0.167	0.81793	0.91963	1.0062 ↑	0.009	0.46231	0.67752	1.7201 ↑	0.7825
DAG160/181	0.098306	0.57898	0.87943 ↓	-0.185	0.21541	0.63228	1.1105 ↑	0.1512	0.30337	0.8936	0.91493 ↓	-0.128	0.71087	0.86555	0.95176 ↓	-0.071	0.39937	0.64058	1.069 ↑	0.0963
TAG511-FA170	0.099448	0.57898	1.1811 ↑	0.2402	0.49117	0.79006	0.9454 ↓	-0.081	0.022078	0.60889	0.8485 ↓	-0.237	0.37544	0.71132	1.058 ↑	0.0814	0.46194	0.67752	0.97633 ↓	-0.035
CER220	0.10888	0.60732	1.3752 ↑	0.4597	0.73115	0.9086	0.98558 ↓	-0.021	0.33566	0.89952	0.82131 ↓	-0.284	0.211	0.56757	0.61633 ↓	-0.698	0.000198	0.020709	0.15823 ↓	-2.66
BUTYRYL-COA_neg_2	0.10943	0.60732	1.2446 ↑	0.3157	0.13238	0.53672	0.73563 ↓	-0.443	0.26729	0.87508	1.6443 ↑	0.7175	0.24488	0.59631	1.3911 ↑	0.4763	0.88644	0.94759	0.77548 ↓	-0.367
TAG463-FA182	0.10995	0.60732	0.7777 ↓	-0.363	0.94627	0.96204	0.98863 ↓	-0.016	0.69332	0.91799	1.1599 ↑	0.214	0.66085	0.85669	1.0127 ↑	0.0183	0.67702	0.83692	1.0377 ↑	0.0534
MESACONIC ACID_pos_1	0.11003	0.60732	0.76436 ↓	-0.388	0.11803	0.50163	1.1903 ↑	0.2513	0.64752	0.91609	1.264 ↑	0.338	0.79754	0.90846	1.2598 ↑	0.3332	0.9296	0.95908	1.1173 ↑	0.1601
DAG160/160	0.1117	0.6086	0.89279 ↓	-0.164	0.52267	0.79933	1.2003 ↑	0.2634	0.95657	0.97962	0.79602 ↓	-0.329	0.73893	0.87828	1.0862 ↑	0.1194	0.14764	0.38494	1.1215 ↑	0.1654
TAG502-FA182	0.11574	0.6202	1.1126 ↑	0.1539	0.21128	0.62867	0.84825 ↓	-0.237	0.63387	0.91609	0.86604 ↓	-0.207	0.051148	0.32506	0.72258 ↓	-0.469	0.020064	0.12727	0.66195 ↓	-0.595
TAG512-FA182	0.11816	0.6202	0.85756 ↓	-0.222	0.40369	0.77199	0.74837 ↓	-0.418	0.5996	0.91609	0.93698 ↓	-0.094	0.17574	0.53303	0.73993 ↓	-0.435	0.041971	0.18707	0.57958 ↓	-0.787
DAG120/181	0.11857	0.6202	0.88716 ↓	-0.173	0.1326	0.53672	1.2087 ↑	0.2735	0.55808	0.91609	1.0099 ↑	0.0142	0.34582	0.69327	1.0954 ↑	0.1315	0.45059	0.67752	1.0162 ↑	0.0231
INDOLE-3-CARBOXYLIC ACID_neg_1	0.12076	0.6202	1.264 ↑	0.338	0.4652	0.789	1.0915 ↑	0.1264	0.015168	0.60183	1.5998 ↑	0.6779	0.26284	0.6071	1.2009 ↑	0.2641	0.34263	0.60156	1.2368 ↑	0.3066
TAG524-FA180	0.12248	0.6202	0.7631 ↓	-0.39	0.51184	0.79092	1.0662 ↑	0.0925	0.23871	0.85757	1.067 ↑	0.0936	0.3708	0.70986	0.90329 ↓	-0.147	0.67273	0.83692	0.91188 ↓	-0.133
TAG492-FA182	0.12258	0.6202	0.90777 ↓	-0.14	0.30782	0.71488	0.84039 ↓	-0.251	0.73971	0.92465	0.96636 ↓	-0.049	0.22953	0.58413	0.83949 ↓	-0.252	0.006268	0.066946	0.72275 ↓	-0.468
TAG441-FA120	0.12775	0.63395	0.89815 ↓	-0.155	0.82279	0.93749	0.92162 ↓	-0.118	0.79934	0.93191	0.97955 ↓	-0.03	0.43473	0.7494	1.1218 ↑	0.1658	0.042696	0.18707	1.1532 ↑	0.2056
TAG583-FA181	0.12828	0.63395	0.76941 ↓	-0.378	0.44433	0.78535	0.95739 ↓	-0.063	0.027533	0.60889	0.65518 ↓	-0.61	0.073087	0.35845	0.40323 ↓	-1.31	0.50412	0.7049	0.6717 ↓	-0.574
TAG482-FA120	0.13044	0.63424	0.88025 ↓	-0.184	0.79073	0.9346	0.99621 ↓	-0.005	0.61297	0.91609	1.1385 ↑	0.1871	0.67383	0.85669	1.0761 ↑	0.1059	0.89185	0.94759	1.0364 ↑	0.0516
L-2-HYDROXYGLUTARIC ACID_neg_1	0.1323	0.63424	0.20596 ↓	-2.28	0.19726	0.61799	1.0673 ↑	0.0939	0.063887	0.65022	0.68931 ↓	-0.537	0.081323	0.38105	1.1043 ↑	0.1432	0.10583	0.33318	1.2614 ↑	0.335
INDOLEACRYLIC ACID_pos_1	0.13282	0.63424	0.80242 ↓	-0.318	0.7528	0.92469	0.87698 ↓	-0.189	0.062876	0.65022	3.1418 ↑	1.6516	0.21875	0.57251	1.1694 ↑	0.2257	0.2631	0.5153	0.89777 ↓	-0.156
TAG541-FA200	0.13755	0.64955	0.85414 ↓	-0.227	0.53299	0.80043	0.78194 ↓	-0.355	0.45361	0.90445	0.69964 ↓	-0.515	0.002243	0.18171	0.63793 ↓	-0.649	0.35356	0.60396	0.7382 ↓	-0.438
TAG471-FA170	0.1409	0.65752	0.88818 ↓	-0.171	0.90773	0.94333	0.88246 ↓	-0.18	0.78447	0.93191	0.83233 ↓	-0.265	0.051612	0.32506	1.1895 ↑	0.2504	0.65537	0.82946	0.98042 ↓	-0.029
TAG523-FA203	0.14266	0.65752	0.81968 ↓	-0.287	0.81343	0.9346	1.104 ↑	0.1428	0.27971	0.88713	0.83892 ↓	-0.253	0.50304	0.79182	0.97326 ↓	-0.039	0.63299	0.8096	0.98227 ↓	-0.026
LACTATE_neg_1	0.14794	0.65752	2.2453 ↑	1.1669	0.25464	0.67444	0.51641 ↓	-0.953	0.67556	0.91633	1.1082 ↑	0.1482	0.095675	0.42356	0.62828 ↓	-0.671	0.74919	0.87474	1.5965 ↑	0.6749
TAG543-FA202	0.14845	0.65752	2.5943 ↑	1.3753	0.005862	0.26577	1.4563 ↑	0.5424	0.30938	0.8936	1.1207 ↑	0.1644	0.67851	0.85718	1.0467 ↑	0.0659	0.48055	0.68766	1.7808 ↑	0.8326
TAG422-FA182	0.14871	0.65752	0.88507 ↓	-0.176	0.27338	0.67721	1.1374 ↑	0.1858	0.082049	0.67934	1.6334 ↑	0.7079	0.18544	0.54197	1.1588 ↑	0.2126	0.0108	0.085834	1.2151 ↑	0.281
TAG482-FA181	0.15373	0.65752	0.94083 ↓	-0.088	0.63353	0.85206	0.94851 ↓	-0.076	0.70293	0.92337	0.96856 ↓	-0.046	0.48822	0.78044	0.9736 ↓	-0.039	0.85875	0.93823	0.99354 ↓	-0.009
TAG540-FA180	0.15605	0.65752	0.77855 ↓	-0.361	0.59791	0.82772	1.0846 ↑	0.1172	0.17193	0.83988	1.1058 ↑	0.1451	0.80181	0.90846	0.93802 ↓	-0.092	0.42267	0.66286	1.1551 ↑	0.2081
DAG180/182	0.15681	0.65752	1.1266 ↑	0.172	0.049667	0.34545	1.3428 ↑	0.4252	0.23877	0.85757	1.178 ↑	0.2363	0.16682	0.53303	1.2044 ↑	0.2684	0.1965	0.44422	1.2118 ↑	0.2772
TAG503-FA181	0.15861	0.65752	0.9468 ↓	-0.079	0.10775	0.46729	0.90093 ↓	-0.151	0.30357	0.8936	0.7967 ↓	-0.328	0.020284	0.28126	0.74217 ↓	-0.43	0.002687	0.047473	0.79279 ↓	-0.335
TAG563-FA202	0.15933	0.65752	2.5189 ↑	1.3328	0.006778	0.26577	1.4635 ↑	0.5494	0.19604	0.85757	1.1489 ↑	0.2003	0.60145	0.83262	1.053 ↑	0.0745	0.38871	0.63055	1.7037 ↑	0.7687
TAG546-FA226	0.16241	0.65752	1.5557 ↑	0.6376	0.48261	0.789	1.0369 ↑	0.0523	0.64896	0.91609	0.82658 ↓	-0.275	0.46687	0.76529	1.1057 ↑	0.1449	0.94431	0.96707	0.93766 ↓	-0.093
TAG520-FA160	0.16545	0.65752	0.87076 ↓	-0.2	0.5718	0.81005	1.0953 ↑	0.1313	0.76045	0.92465	0.93363 ↓	-0.099	0.77075	0.89744	0.96782 ↓	-0.047	0.99811	0.99811	1.0245 ↑	0.035
TAG544-FA183	0.16547	0.65752	0.59221 ↓	-0.756	0.40483	0.77199	1.2175 ↑	0.284	0.80254	0.93191	0.75733 ↓	-0.401	0.76618	0.89458	0.88228 ↓	-0.181	0.384	0.62943	1.1177 ↑	0.1606
TAG492-FA170	0.16757	0.65752	0.89754 ↓	-0.156	0.30618	0.71488	0.74309 ↓	-0.428	0.27628	0.88713	0.76214 ↓	-0.392	0.063571	0.33772	1.2979 ↑	0.3762	0.96144	0.97988	1.0019 ↑	0.0027
TAG564-FA202	0.17024	0.65752	2.6371 ↑	1.399	0.007394	0.26577	1.4257 ↑	0.5117	0.17925	0.84645	1.1445 ↑	0.1947	0.8973	0.96464	1.0204 ↑	0.0291	0.47388	0.68503	1.8176 ↑	0.862
TAG421-FA161	0.17041	0.65752	0.90052 ↓	-0.151	0.76479	0.92899	0.87413 ↓	-0.194	0.32941	0.89952	0.99529 ↓	-0.007	0.52924	0.80522	1.084 ↑	0.1163	0.08413	0.28604	1.0944 ↑	0.1301
CE225	0.17173	0.65752	0.79617 ↓	-0.329	0.12568	0.52368	1.3226 ↑	0.4034	0.64121	0.91609	0.7242 ↓	-0.466	0.65068	0.85669	1.069 ↑	0.0963	0.24895	0.50182	0.86757 ↓	-0.205
TAG480-FA160	0.17242	0.65752	1.1038 ↑	0.1425	0.34377	0.7364	1.1273 ↑	0.1729	0.66117	0.91609	0.92223 ↓	-0.117	0.74568	0.87952	1.0253 ↑	0.0361	0.91181	0.95558	0.9601 ↓	-0.059
TAG441-FA161	0.17337	0.65752	0.93152 ↓	-0.102	0.48268	0.789	0.8243 ↓	-0.279	0.99323	0.99592	0.88697 ↓	-0.173	0.1066	0.44277	1.1518 ↑	0.2039	0.19974	0.44915	1.0839 ↑	0.1163
TAG461-FA180	0.17428	0.65752	0.87202 ↓	-0.198	0.15864	0.57627	1.2036 ↑	0.2674	0.041769	0.60889	1.341 ↑	0.4233	0.88953	0.96441	1.0156 ↑	0.0224	0.34395	0.60156	1.1153 ↑	0.1574
CE181	0.1762	0.65752	1.0764 ↑	0.1062	0.10715	0.46729	1.1386 ↑	0.1872	0.68396	0.91633	0.29757 ↓	-1.749	0.79838	0.90846	0.92914 ↓	-0.106	0.00081	0.04108	0.79238 ↓	-0.336
TAG526-FA160	0.17762	0.65752	0.54116 ↓	-0.886	0.36406	0.74746	1.1767 ↑	0.2348	0.85348	0.9446	0.89644 ↓	-0.158	0.41873	0.74773	0.88675 ↓	-0.173	0.25031	0.50182	1.1677 ↑	0.2237
TAG462-FA181	0.17837	0.65752	0.92859 ↓	-0.107	0.54796	0.80863	1.0955 ↑	0.1316	0.18475	0.85075	1.3263 ↑	0.4074	0.51913	0.79414	1.0749 ↑	0.1043	0.3817	0.62943	1.1874 ↑	0.2478
TAG440-FA120	0.17897	0.65752	0.91676 ↓	-0.125	0.23212	0.64607	1.0709 ↑	0.0988	0.20171	0.85757	1.1619 ↑	0.2164	0.18134	0.53895	1.0742 ↑	0.1033	0.0104	0.085834	1.233 ↑	0.3022
TAG565-FA202	0.17899	0.65752	2.6828 ↑	1.4238	0.008466	0.27678	1.4179 ↑	0.5038	0.13359	0.78853	1.1649 ↑	0.2202	0.94871	0.98367	1.0164 ↑	0.0234	0.40167	0.64176	1.9874 ↑	0.9909
TAG441-FA160	0.18042	0.65752	0.94143 ↓	-0.087	0.34883	0.7364	1.0936 ↑	0.1291	0.10302	0.74163	1.2656 ↑	0.3398	0.43995	0.74968	1.045 ↑	0.0636	0.2758	0.52418	1.0848 ↑	0.1174
TAG520-FA180	0.18101	0.65752	0.86667 ↓	-0.206	0.45165	0.78641	1.1249 ↑	0.1698	0.48932	0.90445	0.96381 ↓	-0.053	0.7833	0.90218	0.97303 ↓	-0.039	0.74421	0.87416	1.0417 ↑	0.0589
TAG513-FA182	0.19942	0.71302	0.92205 ↓	-0.117	0.029543	0.33653	0.61709 ↓	-0.696	0.6892	0.91799	0.83895 ↓	-0.253	0.14846	0.52121	0.63986 ↓	-0.644	0.000173	0.020709	0.48244 ↓	-1.052
TAG482-FA140	0.19965	0.71302	0.94158 ↓	-0.087	0.28135	0.68721	0.86133 ↓	-0.215	0.95525	0.97962	0.86511 ↓	-0.209	0.052414	0.32506	0.82038 ↓	-0.286	0.041271	0.1866	0.88456 ↓	-0.177
DAG160/161	0.20506	0.71307	1.1409 ↑	0.1901	0.50046	0.79092	1.1253 ↑	0.1703	0.68563	0.91633	0.87037 ↓	-0.2	0.13056	0.48672	1.162 ↑	0.2166	0.74571	0.87416	1.002 ↑	0.0029
TAG566-FA202	0.20642	0.71307	2.4258 ↑	1.2785	0.033617	0.33653	1.4093 ↑	0.495	0.04936	0.63569	1.2182 ↑	0.2847	0.51675	0.79414	0.98378 ↓	-0.024	0.20769	0.45155	2.1131 ↑	1.0794

TAG502-FA180	0.20795	0.71307	0.92949 ↓	-0.105	0.79533	0.9346	0.9407 ↓	-0.088	0.23597	0.85757	1.1414 ↑	0.1908	0.010762	0.25411	0.64385 ↓	-0.635	0.027327	0.15485	0.67874 ↓	-0.559
TAG493-FA182	0.20796	0.71307	0.92875 ↓	-0.107	0.05669	0.34545	0.77089 ↓	-0.375	0.59674	0.91609	0.86143 ↓	-0.215	0.69447	0.85996	0.99018 ↓	-0.014	0.06158	0.24233	0.86033 ↓	-0.217
TAG505-FA160	0.20805	0.71307	0.81295 ↓	-0.299	0.070076	0.39187	1.2373 ↑	0.3072	0.88858	0.96338	0.95229 ↓	-0.071	0.019731	0.28126	0.7798 ↓	-0.359	0.25268	0.50182	0.83722 ↓	-0.256
2-KETOHEXANOIC ACID_pos_2	0.21222	0.72154	1.2262 ↑	0.2942	0.43846	0.78535	1.0369 ↑	0.0523	0.027677	0.60889	1.5349 ↑	0.6181	0.071085	0.35542	1.6782 ↑	0.7469	0.34808	0.60203	1.1685 ↑	0.2247
TAG481-FA140	0.21566	0.72583	1.0624 ↑	0.0873	0.63154	0.85206	0.98028 ↓	-0.029	0.53395	0.91609	1.0349 ↑	0.0494	0.38126	0.71215	0.95832 ↓	-0.061	0.1019	0.32507	0.94692 ↓	-0.079
2-HYDROXY-3-METHYLBUTYRIC ACID_pos	0.22013	0.72583	1.1594 ↑	0.2134	0.89559	0.94333	1.024 ↑	0.0343	0.044091	0.60889	1.4815 ↑	0.567	0.92223	0.97742	1.0834 ↑	0.1155	0.58752	0.76856	1.2414 ↑	0.312
TAG420-FA160	0.22094	0.72583	0.92391 ↓	-0.114	0.095574	0.46158	1.1293 ↑	0.1754	0.07538	0.67934	1.3466 ↑	0.4294	0.25295	0.59631	1.07 ↑	0.0976	0.0293	0.16172	1.2714 ↑	0.3464
TAG482-FA141	0.22095	0.72583	0.92089 ↓	-0.119	0.27075	0.67686	0.89643 ↓	-0.158	0.85287	0.9446	0.90249 ↓	-0.148	0.42208	0.7494	0.93016 ↓	-0.104	0.8188	0.92273	0.97251 ↓	-0.04
L-ORNITHINE_pos_1	0.22306	0.72583	0.52713 ↓	-0.924	0.089531	0.44766	1.9841 ↑	0.9885	0.29257	0.8936	1.1456 ↑	0.1961	0.11636	0.45371	1.5729 ↑	0.6535	0.40848	0.64777	1.3358 ↑	0.4177
TAG503-FA140	0.22627	0.72583	0.90387 ↓	-0.146	0.051709	0.34545	0.78732 ↓	-0.345	0.62586	0.91609	0.72251 ↓	-0.469	0.031076	0.29777	0.59095 ↓	-0.759	0.027228	0.15485	0.59935 ↓	-0.739
TAG472-FA182	0.22685	0.72583	0.90449 ↓	-0.145	0.67705	0.87102	0.93076 ↓	-0.104	0.46022	0.90445	1.0071 ↑	0.0101	0.11947	0.45744	1.168 ↑	0.2241	0.38038	0.62943	0.93356 ↓	-0.099
FFA205	0.22737	0.72583	1.4021 ↑	0.4876	0.029546	0.33653	1.3923 ↑	0.4775	0.13117	0.78853	1.0724 ↑	0.1008	0.11173	0.44797	1.2682 ↑	0.3427	0.37227	0.62535	1.1949 ↑	0.2569
TAG544-FA160	0.22885	0.72583	1.2516 ↑	0.3238	0.66717	0.87102	0.8438 ↓	-0.245	0.50822	0.90566	1.0779 ↑	0.1082	0.63055	0.84537	0.81527 ↓	-0.295	0.74562	0.87416	0.76608 ↓	-0.384
N-GLYCYL-L-PROLINE_neg_3	0.23446	0.73813	0.69115 ↓	-0.533	0.93742	0.96001	1.1748 ↑	0.2324	0.41131	0.90445	1.42 ↑	0.5059	0.64073	0.85097	1.2467 ↑	0.3182	0.21734	0.46453	1.4276 ↑	0.5136
TAG461-FA120	0.24539	0.7623	0.92733 ↓	-0.109	0.92004	0.95011	0.99562 ↓	-0.006	0.16304	0.82488	1.2285 ↑	0.2969	0.96147	0.98367	1.034 ↑	0.0483	0.3722	0.62535	1.0723 ↑	0.1008
TAG441-FA140	0.24573	0.7623	0.93631 ↓	-0.095	0.64533	0.85708	0.8636 ↓	-0.212	0.56332	0.91609	1.0057 ↑	0.0082	0.17276	0.53303	1.1131 ↑	0.1546	0.11217	0.33764	1.1052 ↑	0.1444
FFA203	0.24804	0.76305	1.3321 ↑	0.4137	0.49043	0.79006	1.1394 ↑	0.1882	0.3032	0.8936	0.96035 ↓	-0.058	0.66292	0.85669	1.136 ↑	0.184	0.27376	0.52418	1.1408 ↑	0.19
TAG526-FA181	0.24956	0.76305	0.92747 ↓	-0.109	0.03977	0.33653	1.2195 ↑	0.2863	0.38708	0.89952	1.0284 ↑	0.0404	0.38733	0.71215	1.1317 ↑	0.1785	0.42706	0.66728	1.0628 ↑	0.0879
TAG482-FA182	0.25342	0.7693	0.96029 ↓	-0.058	0.58354	0.82143	0.93399 ↓	-0.099	0.26046	0.86881	1.0071 ↑	0.0102	0.14608	0.52121	0.87768 ↓	-0.188	0.030465	0.16195	0.88241 ↓	-0.18
DAG140/181	0.26079	0.78608	0.89645 ↓	-0.158	0.019973	0.30837	1.3672 ↑	0.4512	0.49844	0.90528	1.093 ↑	0.1283	0.22045	0.57251	1.1671 ↑	0.2229	0.58159	0.76801	1.027 ↑	0.0384
TAG555-FA182	0.26418	0.79069	0.7857 ↓	-0.348	0.06799	0.39049	1.2613 ↑	0.3349	0.63828	0.91609	0.98916 ↓	-0.016	0.53718	0.80672	1.0829 ↑	0.115	0.011929	0.085834	1.1281 ↑	0.1739
TAG587-FA225	0.2692	0.79116	0.61095 ↓	-0.711	0.58884	0.82143	0.89547 ↓	-0.159	0.7148	0.92337	0.9225 ↓	-0.116	0.26768	0.6072	0.96271 ↓	-0.055	0.55997	0.75958	0.9135 ↓	-0.131
FFA204	0.27199	0.79116	1.3174 ↑	0.3977	0.065086	0.37893	1.3753 ↑	0.4598	0.12589	0.78853	1.0953 ↑	0.1314	0.38177	0.71215	1.1292 ↑	0.1753	0.27346	0.52418	1.204 ↑	0.2679
TAG534-FA170	0.27423	0.79116	0.81548 ↓	-0.294	0.89673	0.94333	1.0297 ↑	0.0423	0.82734	0.93765	0.95161 ↓	-0.072	0.28904	0.62673	0.96044 ↓	-0.058	0.10908	0.33764	1.1715 ↑	0.2283
TAG523-FA183	0.27529	0.79116	0.65761 ↓	-0.605	0.32344	0.7222	1.1332 ↑	0.1804	0.61551	0.91609	0.76675 ↓	-0.383	0.1891	0.54255	0.87653 ↓	-0.19	0.3617	0.61244	1.1764 ↑	0.2344
TAG421-FA140	0.27604	0.79116	0.94867 ↓	-0.076	0.90625	0.94333	0.9654 ↓	-0.051	0.19491	0.85757	1.2004 ↑	0.2635	0.053075	0.32506	1.1706 ↑	0.2273	0.076329	0.27224	1.1273 ↑	0.1729
TAG552-FA181	0.27674	0.79116	0.92754 ↓	-0.109	0.90663	0.94333	0.99941 ↓	-9E-04	0.91272	0.96978	0.94957 ↓	-0.075	0.31498	0.65459	0.9811 ↓	-0.028	0.63435	0.8096	0.95448 ↓	-0.067
TAG532-FA160	0.27737	0.79116	0.88985 ↓	-0.168	0.57165	0.81005	0.91445 ↓	-0.129	0.14579	0.82488	0.84776 ↓	-0.238	0.83336	0.93168	1.0235 ↑	0.0336	0.40453	0.64391	0.95728 ↓	-0.063
TAG521-FA201	0.28706	0.81335	1.2548 ↑	0.3275	0.50623	0.79092	1.1269 ↑	0.1723	0.015577	0.60183	0.72493 ↓	-0.464	0.51771	0.79414	0.93873 ↓	-0.091	0.83224	0.92592	0.93241 ↓	-0.101
TAG514-FA161	0.28927	0.81417	0.94931 ↓	-0.075	0.88651	0.94333	1.0221 ↑	0.0315	0.46049	0.90445	0.86937 ↓	-0.202	0.25512	0.59631	0.94186 ↓	-0.086	0.67764	0.83692	0.9615 ↓	-0.057
FFA225	0.29609	0.82773	1.2784 ↑	0.3543	0.053223	0.34545	1.3608 ↑	0.4445	0.16263	0.82488	1.0113 ↑	0.0162	0.54107	0.80717	1.0941 ↑	0.1298	0.27391	0.52418	1.1633 ↑	0.2182
TAG582-FA181	0.29798	0.82773	0.94998 ↓	-0.074	0.92835	0.95348	0.73788 ↓	-0.439	0.36005	0.89952	0.79496 ↓	-0.331	0.040811	0.3212	0.382 ↓	-1.388	0.21751	0.46453	0.48652 ↓	-1.039
TAG491-FA160	0.3042	0.83952	1.0807 ↑	0.112	0.025863	0.33123	0.78066 ↓	-0.357	0.52634	0.91257	0.87261 ↓	-0.197	0.55704	0.81895	0.9613 ↓	-0.057	0.042358	0.18707	0.89316 ↓	-0.163
TAG502-FA160	0.3087	0.8401	1.0378 ↑	0.0536	0.24846	0.67022	0.80094 ↓	-0.32	0.4487	0.90445	0.7307 ↓	-0.453	0.01959	0.28126	0.54816 ↓	-0.867	0.037334	0.1803	0.57661 ↓	-0.794
TAG481-FA181	0.31014	0.8401	1.0689 ↑	0.0961	0.80325	0.9346	1.0023 ↑	0.0033	0.39156	0.89952	1.1014 ↑	0.1394	0.6534	0.85669	0.98887 ↓	-0.016	0.88529	0.94759	1.0442 ↑	0.0625
BUTYRYL-COA_pos_1	0.31191	0.8401	1.0619 ↑	0.0867	0.72058	0.90338	0.85913 ↓	-0.219	0.037312	0.60889	2.2851 ↑	1.1922	0.80811	0.911	0.90777 ↓	-0.14	0.10021	0.32265	1.902 ↑	0.9275
DAG161/182	0.31232	0.8401	1.1663 ↑	0.2219	0.2319	0.64607	1.4388 ↑	0.5249	0.33819	0.89952	1.4099 ↑	0.4956	0.26735	0.6072	1.2969 ↑	0.3751	0.33941	0.60156	1.2208 ↑	0.2878
TAG520-FA200	0.31864	0.85171	0.80524 ↓	-0.313	0.6404	0.85588	0.98863 ↓	-0.016	0.74989	0.92465	0.86001 ↓	-0.218	0.61422	0.83668	0.91903 ↓	-0.122	0.88746	0.94759	1.0868 ↑	0.1201
TAG503-FA141	0.32196	0.8548	0.86439 ↓	-0.21	0.20326	0.61799	0.81662 ↓	-0.292	0.57913	0.91609	0.87574 ↓	-0.191	0.042836	0.32176	0.69337 ↓	-0.528	0.6059	0.78508	0.91095 ↓	-0.135
TAG481-FA161	0.32382	0.8548	1.0694 ↑	0.0968	0.1211	0.50956	0.80926 ↓	-0.305	0.38694	0.89952	0.85791 ↓	-0.221	0.94051	0.98253	1.0033 ↑	0.0047	0.89534	0.94839	1.0038 ↑	0.0054
TAG587-FA180	0.32632	0.85609	0.7681 ↓	-0.381	0.33502	0.7325	0.99158 ↓	-0.012	0.66174	0.91609	0.86778 ↓	-0.205	0.20027	0.55631	0.92118 ↓	-0.118	0.19235	0.44188	0.89482 ↓	-0.16
TAG441-FA181	0.32927	0.85854	0.94746 ↓	-0.078	0.40688	0.77199	1.1224 ↑	0.1666	0.065905	0.65022	1.6508 ↑	0.7232	0.5305	0.80522	1.0836 ↑	0.1158	0.032162	0.1643	1.2879 ↑	0.365
FFA182	0.33749	0.87458	1.2744 ↑	0.3498	0.23258	0.64607	1.104 ↑	0.1428	0.18616	0.85075	1.2172 ↑	0.2836	0.062202	0.33463	1.2103 ↑	0.2753	0.15008	0.38657	1.2025 ↑	0.2661
DCER260	0.33994	0.87561	0.95002 ↓	-0.074	0.56568	0.81005	1.0804 ↑	0.1115	0.79278	0.93191	0.85903 ↓	-0.219	0.99813	0.99876	1.1789 ↑	0.2374	0.6947	0.84616	0.80446 ↓	-0.314
ARGININOSUCCINIC ACID_neg_2	0.34365	0.87894	0.71051 ↓	-0.493	0.4731	0.789	0.66508 ↓	-0.588	0.083354	0.67934	0.73667 ↓	-0.441	0.31716	0.65459	1.0297 ↑	0.0422	0.34106	0.60156	1.6933 ↑	0.7599
TAG533-FA160	0.34744	0.87894	0.89454 ↓	-0.161	0.10605	0.46729	1.1353 ↑	0.1831	0.42111	0.90445	0.89339 ↓	-0.163	0.93041	0.98225	1.042 ↑	0.0594	0.49488	0.70342	0.94614 ↓	-0.08
TAG440-FA180	0.34842	0.87894	0.93503 ↓	-0.097	0.031906	0.33653	1.201 ↑	0.2643	0.029514	0.60889	1.4473 ↑	0.5334	0.30768	0.65057	1.0698 ↑	0.0974	0.050458	0.21413	1.1388 ↑	0.1875
SPERMIDINE_pos_4	0.34951	0.87894	1.6407 ↑	0.7143	0.8845	0.94333	0.87357 ↓	-0.195	0.12079	0.78472	0.82379 ↓	-0.28	0.85575	0.94712	0.90123 ↓	-0.15	0.22548	0.47675	0.84761 ↓	-0.239
TAG563-FA182	0.35383	0.88457	0.85382 ↓	-0.228	0.055964	0.34545	0.6973 ↓	-0.52	0.15561	0.82488	0.75805 ↓	-0.4	0.001114	0.18171	0.40071 ↓	-1.319	0.19227	0.44188	0.60599 ↓	-0.723
TAG561-FA160	0.35645	0.88593	0.92931 ↓	-0.106	0.10218	0.46695	0.7178 ↓	-0.478	0.25405	0.85757	0.84969 ↓	-0.235	0.02779	0.29511	0.51957 ↓	-0.945	0.90967	0.95558	0.74493 ↓	-0.425
TAG501-FA160	0.3632	0.89744	1.1494 ↑	0.2009	0.46246	0.789	0.84165 ↓	-0.249	0.83948	0.93937	0.86697 ↓	-0.206	0.052244	0.32506	0.73348 ↓	-0.447	0.005523	0.063438	0.61511 ↓	-0.701
TAG546-FA160	0.36821	0.9004	1.2939 ↑	0.3718	0.50118	0.79092	0.84834 ↓	-0.237	0.48947	0.90445	1.0284 ↑	0.0404	0.21991	0.57251	0.65999 ↓	-0.599	0.41641	0.6579	0.6266 ↓	-0.674
TAG542-FA202	0.37083	0.9004	2.1977 ↑	1.136	0.043767	0.33653	1.2686 ↑	0.3433	0.35083	0.89952	1.0508 ↑	0.0715	0.27038	0.6072	0.93255 ↓	-0.101	0.80042	0.90957	1.501 ↑	0.5859
TAG400-FA120	0.37507	0.9004	0.92542 ↓	-0.112	0.86502	0.94333	0.91107 ↓	-0.134	0.47767	0.90445	1.0978 ↑	0.1346	0.044766	0.32176	1.2132 ↑	0.2788	0.002394	0.047473	1.3039 ↑	0.3828
TAG567-FA225	0.37647	0.9004	0.49103 ↓	-1.026	0.59143	0.82143	0.88785 ↓	-0.172	0.63632	0.91609	0.92777 ↓	-0.108	0.2077	0.56224	0.96317 ↓	-0.054	0.84181	0.92927	1.0704 ↑	0.0981
TAG505-FA205	0.37677	0.9004	0.82363 ↓	-0.28	0.012443	0.29385	1.335 ↑	0.4168	0.48682	0.90445	1.0927 ↑	0.128	0.86889	0.94947	1.0309 ↑	0.0439	0.38052	0.62943	1.0781 ↑	0.1084
ASPARTATE_pos_3	0.37711	0.9004	1.1649 ↑	0.2202	0.26554	0.67444	1.0844 ↑	0.1169	0.033945	0.60889	1.5578 ↑	0.6396	0.12186	0.45854	1.3531 ↑	0.4363	0.24225	0.49978	1.1882 ↑	0.2488
TAG524-FA161	0.38486	0.91075	1.1277 ↑	0.1733	0.081204	0.42607	0.59787 ↓	-0.742	0.34162	0.89952	0.56293 ↓	-0.829	0.02032	0.28126	0.25462 ↓	-1.974	0.002865	0.047473	0.2934 ↓	-1.769
TAG567-FA204	0.38573	0.91075	0.91977 ↓	-0.121	0.82534	0.93768	0.83182 ↓	-0.266	0.38611	0.89952	0.80268 ↓	-0.317	0.00549	0.23331	0.24329 ↓	-2.039	0.006301	0.066946	0.37165 ↓	-1.428
FFA141	0.38991	0.91373	1.2739 ↑	0.3493	0.020624	0.30837	1.4215 ↑	0.5074	0.2965	0.8936	1.0186 ↑	0.0266	0.10939	0.44277	1.2105 ↑	0.2756	0.82503	0.92273	1.0917 ↑	0.1266
TAG500-FA160	0.39129	0.91373	0.97167 ↓	-0.041	0.29088	0.69064	1.1614 ↑	0.2158	0.72409	0.92465	0.87697 ↓	-0.189	0.83522	0.93168	1.002 ↑	0.0029	0.461	0.67752	0.9715 ↓	-0.042
TAG462-FA140	0.4079	0.94422	0.97179 ↓	-0.041	0.51649	0.79099	0.84234 ↓	-0.248	0.65877	0.91609	0.89431 ↓	-0.161	0.58222	0.82758	1.0874 ↑	0.1209	0.50586	0.7049	1.0089 ↑	0.0127
ADP_pos_1	0.41374	0.94422	1.138 ↑	0.1865	0.042245	0.33653	1.366 ↑	0.4499	0.000919	0.38156	2.1204 ↑	1.0843	0.22233	0.57251	1.3628 ↑	0.4466	0.8582	0.93823	1.1068 ↑	0.1464
TAG502-FA181	0.41759	0.94422	1.0692 ↑	0.0966	0.15218	0.56242	0.8652 ↓	-0.209	0.87986	0.95883	0.75563 ↓	-0.404	0.026757	0.29511	0.72207 ↓	-0.47	0.011851	0.085834	0.77311 ↓	-0.371
1-METHYLADENOSINE_pos_1	0.41811	0.94422	1.2605 ↑	0.334	0.60982	0.83604	1.1526 ↑	0.2049	0.39065	0.89952	1.172 ↑	0.229	0.78869	0.90593	0.84142 ↓	-0.249	0.0994	0.32248	2.4011 ↑	1.2637
TAG543-FA160	0.41921	0.94422	1.1344 ↑	0.182	0.76505	0.92899	0.81404 ↓	-0.297	0.31406	0.8958	0.81355 ↓	-0.298	0.020785	0.28126	0.52049 ↓	-0.942	0.34864	0.60203	0.55388 ↓	-0.852
TAG545-FA160	0.42165	0.94422	1.0621 ↑	0.0869	0.55933	0.81005	0.68233 ↓	-0.551	0.78173	0.93191	0.87953 ↓	-0.185	0.19838	0.55631	0.40026 ↓	-1.321	0.013715	0.095558	0.23112 ↓	-2.113
TAG470-FA140	0.42208	0.94422	0.95665 ↓	-0.064	0.85671	0.94333	0.99341 ↓	-0.01	0.36933	0.89952	1.0166 ↑	0.0237	0.075272	0.36353	1.1106 ↑	0.1513	0.27171	0.52418	1.0435 ↑	0.0614
URIDINE 5-DIPHOSPHATE_neg_2	0.42533	0.94422	0.63389 ↓	-0.658	0.56136	0.81005	0.69099 ↓	-0.533	0.11666	0.77472	0.74157 ↓	-0.431	0.9542	0.98367	1.0925 ↑	0.1276	0.25021	0.50182	2.4342 ↑	1.2835
TAG481-FA160	0.42915	0.94422	1.0552 ↑	0.0776	0.10138	0.46695	0.85795 ↓	-0.221	0.75573	0.92465	0.9479 ↓	-0.077	0.16198	0.53303	0.89095 ↓	-0.167	0.17608	0.41806	0.94254 ↓	-0.085
TAG512-FA170	0.4308	0.94422	0.96635 ↓	-0.049	0.026498	0.33123	0.74118 ↓	-0.432	0.83846	0.93937	0.85246 ↓	-0.23	0.89758	0.96464	0.93709 ↓	-0.094	0.002011	0.04577	0.7145 ↓	-0.485
TAG513-FA161	0.43326	0.94422	0.96969 ↓	-0.044	0.28878	0.6895	0.83756 ↓	-0.256	0.44264	0.90445	0.92763 ↓	-0.108	0.37381	0.71132	1.1201 ↑	0.1636	0.56119	0.75958	1.0839 ↑	0.1163
4-AMINOBENZOIC ACID_pos_1	0.43544	0.94422	0.66085 ↓	-0.598	0.14573	0.55797	0.70153 ↓	-0.511	0.017933	0.60889	0.66538 ↓	-0.588	0.16021	0.53303	0.65858 ↓	-0.603	0.62979	0.80865	1.0851 ↑	0.1178
TAG400-FA140	0.43581	0.94422	0.93827 ↓	-0.092	0.58888	0.82143	0.98245 ↓	-0.026	0.23855	0.85757	1.1795 ↑	0.2381	0.044454	0.32176	1.1954 ↑	0.2575	0.001137	0.043922	1.3097 ↑	0.3892
L-CARNITINE_pos_1	0.43661	0.94422	2.5633 ↑	1.358	0.29437	0.69505	0.54653 ↓	-0.872	0.16216	0.82488	0.75263 ↓	-0.41	0.89343	0.96464	1.1369 ↑	0.1852	0.50587	0.7049	0.97693 ↓	-0.034
TAG525-FA205	0.44067	0.94422	0.89301 ↓	-0.163	0.000661	0.14046	1.8784 ↑	0.9095	0.012299	0.58079	1.6178 ↑	0.694	0.055069	0.32506	1.3643 ↑	0.4482	0.001714	0.04577	1.5215 ↑	0.6055
TAG472-FA140	0.44094	0.94422	0.94966 ↓	-0.075	0.66785	0.87102	0.86444 ↓	-0.21	0.97351	0.98745	0.88909 ↓	-0.17	0.89881	0.96464	1.0576 ↑	0.0807	0.542	0.74306	0.95992 ↓	-0.059
TAG504-FA160	0.44212	0.94422	0.86209 ↓	-0.214	0.87846	0.94333	0.98182 ↓	-0.026	0.78722	0.93191	0.88469 ↓	-0.177	0.99872	0.99876	0.9796 ↓	-0.03	0.16359	0.39958	0.71824 ↓	-0.477
TAG400-FA160	0.45332	0.95335	0.95346 ↓	-0.069	0.11322	0.48603	1.1212 ↑	0.165	0.03837	0.60889	1.4457 ↑	0.5317	0.25492	0.59631	1.0882 ↑	0.122	0.007904	0.079486	1.3117 ↑	0.3914
TAG503-FA161	0.45443	0.95335	1.0849 ↑	0.1176	0.019659	0.30837	0.72521 ↓	-0.464	0.078328	0.67934	0.67521 ↓	-0.567	0.057417	0.32639	0.60021 ↓	-0.736	0.002563	0.047473	0.67234 ↓	-0.573
TAG502-FA161	0.45618	0.95335	1.0697 ↑	0.0972	0.004489	0.26577	0.73222 ↓	-0.45	0.22045	0.85757	0.68226 ↓	-0.552	0.082712	0.38209	0.69329 ↓	-0.528	0.011945	0.085834	0.75931 ↓	-0.397
TAG511-FA150	0.46034	0.95335	1.0297 ↑	0.0422	0.039324	0.33653	0.78907 ↓	-0.342	0.81994	0.93449	0.89443 ↓	-0.161	0.17684	0.53303	0.80309 ↓	-0.316	0.041264	0.1866	0.6777 ↓	-0.561
TAG442-FA181	0.46762	0.95335	0.96347 ↓	-0.054	0.27407	0.67721	1.2009 ↑	0.2641	0.028643	0.60889	1.8934 ↑	0.921	0.50521	0.79231	1.1031 ↑	0.1415	0.20231	0.45155	1.1508 ↑	0.2027
TAG472-FA150	0.47225	0.95335	0.97842 ↓	-0.031	0.28576	0.6895	0.81411 ↓	-0.297	0.7276	0.92465	0.90996 ↓	-0.136	0.71827	0.86769	1.0469 ↑	0.0661	0.91736	0.95558	1.0043 ↑	0.0062
TAG510-FA160	0.47237	0.95335	0.95175 ↓	-0.071	0.61257	0.83712	1.0605 ↑	0.0847	0.67512	0.91633	0.92288 ↓	-0.116	0.86905	0.94947	0.99469 ↓	-0.008	0.77699	0.89008	0.96473 ↓	-0.052
TAG451-FA160	0.47371	0.95335	1.0878 ↑	0.1214	0.60678	0.83457	1.0124 ↑	0.0178	0.8335	0.93937	0.90697 ↓	-0.141	0.040726	0.3212	1.1483 ↑	0.1995	0.088177	0.29508	1.2117 ↑	0.2771
TAG534-FA182	0.47432	0.95335	0.96514 ↓	-0.051	0.03821	0.33653	1.1771 ↑	0.2353	0.43237	0.90445	1.0034 ↑	0.0049	0.51946	0.79414	1.0979 ↑	0.1348	0.25168	0.50182	1.1228 ↑	0.167
TAG524-FA183	0.47481	0.95335	0.68727 ↓	-0.541	0.28562	0.6895	1.238 ↑	0.308	0.52819	0.91257	0.56314 ↓	-0.828	0.41443	0.74773	0.87489 ↓	-0.193	0.12141	0.34632	1.1415 ↑	0.1909
TAG471-FA150	0.47802	0.95335	1.0663 ↑	0.0926	0.20357	0.61799	0.79201 ↓	-0.336	0.82229	0.93449	0.86855 ↓	-0.203	0.43013	0.7494	1.0719 ↑	0.1001	0.9319	0.95908	0.99042 ↓	-0.014
TAG555-FA181	0.48026	0.95335	0.965 ↓	-0.051	0.022493	0.30837	1.2147 ↑	0.2806	0.51208	0.90566	0.98295 ↓	-0.025	0.53246	0.80532	1.1103 ↑	0.1509	0.3486	0.60203	1.0935 ↑	0.129
SM160	0.48236	0.95335	0.76573 ↓	-0.385	0.41141	0.77704	1.1884 ↑	0.249	0.30622	0.8936	1.1537 ↑	0.2063	0.17073	0.53303	1.2059 ↑	0.2701	0.02097	0.12916	1.335 ↑	0.4168
TAG420-FA120	0.48349	0.95335	0.95017 ↓	-0.074	0.96147	0.9683	0.94298 ↓	-0.085	0.3899	0.89952	1.1112 ↑	0.1522	0.04182	0.32176	1.181 ↑	0.24	0.001802	0.04577	1.2942 ↑	0.3721
TAG471-FA140	0.48917	0.95335	0.98236 ↓	-0.026	0.41917	0.77704	0.86182 ↓	-0.215	0.77018	0.92465	0.86058 ↓	-0.217	0.17267	0.53303	1.1117 ↑	0.1527	0.78407	0.89338	0.98799 ↓	-0.017
TAG481-FA120	0.48963	0.95335	0.89504 ↓	-0.16	0.85888	0.94333	1.0227 ↑	0.0324	0.26792	0.87508	1.3714 ↑	0.4557	0.62125	0.83832	1.0045 ↑	0.0064	0.15543	0.39557	1.1748 ↑	0.2325
TAG450-FA140	0.48988	0.95335	0.97069 ↓	-0.043	0.86327	0.94333	0.98466 ↓	-0.022	0.51356	0.90566	0.98374 ↓	-0.024	0.084318	0.38533	1.0929 ↑	0.1282	0.056279	0.2278	1.1472 ↑	0.1981
TAG470-FA170	0.49013	0.95335	0.95732 ↓	-0.063	0.72511	0.90638	1.0153 ↑	0.0219	0.79414	0.93191	0.95553 ↓	-0.066	0.073376	0.35845	1.0956 ↑	0.1317	0.18248	0.42848	1.0611 ↑	0.0856
TAG492-FA181	0.49167	0.95335	0.97551 ↓	-0.036	0.13672	0.53963	0.82581 ↓	-0.276	0.44033	0.90445	0.86015 ↓	-0.217	0.95934	0.98367	0.99995 ↓	-7E-05	0.039513	0.18659	0.90604 ↓	-0.142
CE140	0.49265	0.95335	0.93863 ↓	-0.091	0.089131	0.44766	1.3754 ↑	0.4599	0.96298	0.98381	0.8132 ↓	-0.298	0.36323	0.70813	1.0945 ↑	0.1302	0.065111	0.24489	0.79146 ↓	-0.337
FFA202	0.49637	0.95335	1.2166 ↑	0.2829	0.42806	0.7808	1.1503 ↑	0.202	0.47456	0.90445	0.89474 ↓	-0.16	0.38844	0.71215	1.1624 ↑	0.2171	0.38497	0.62943	1.13 ↑	0.1763
TAG524-FA182	0.49885	0.95335	0.69081 ↓	-0.534	0.70028	0.89107	1.1628 ↑	0.2177	0.3593	0.89952	0.68974 ↓	-0.536	0.1078	0.44277	0.58672 ↓	-0.769	0.85528	0.93823	0.86346 ↓	-0.212
TAG502-FA141	0.50122	0.95335	0.9697 ↓	-0.044	0.75842	0.92624	0.92416 ↓	-0.114	0.86624	0.95148	0.94839 ↓	-0.076	0.69191	0.85996	0.89613 ↓	-0.158	0.24974	0.50182	1.2126 ↑	0.2781
TAG511-FA160	0.50304	0.95335	0.98393 ↓	-0.023	0.16376	0.58981	0.89761 ↓	-0.156	0.24577	0.85757	0.89439 ↓	-0.161	0.34171	0.69155	0.96504 ↓	-0.051	0.026974	0.15485	0.85628 ↓	-0.224
TAG528-FA161	0.50313	0.95335	0.93316 ↓	-0.1	0.84823	0.94333	0.94124 ↓	-0.087	0.6441	0.91609	0.93795 ↓	-0.092	0.43787	0.74968	1.1344 ↑	0.1819	0.46218	0.67752	1.0727 ↑	0.1013
SPERMIDINE_pos_2	0.50483	0.95335	0.75763 ↓	-0.4	0.52762	0.79566	0.7496 ↓	-0.416	0.05774	0.65022	1.9899 ↑	0.9927	0.31485	0.65459	1.4287 ↑	0.5147	0.58953	0.76856	1.1129 ↑	0.1543
TAG421-FA181	0.50994	0.95335	0.94984 ↓	-0.074	0.3617	0.74622	1.1584 ↑	0.2121	0.022829	0.60889	1.8191 ↑	0.8632	0.67032	0.85669	1.0484 ↑	0.0681	0.009523	0.085834	1.3357 ↑	0.4176
TAG542-FA201	0.51216	0.95335	1.4521 ↑	0.5382	0.90782	0.94333	0.94209 ↓	-0.086	0.33835	0.89952	0.71069 ↓	-0.493	0.007323	0.24008	0.53215 ↓	-0.91	0.010011	0.085834	0.57215 ↓	-0.806
FFA180	0.51271	0.95335	0.99172 ↓	-0.012	0.22197	0.63312	1.2182 ↑	0.2848	0.60372	0.91609	0.88056 ↓	-0.184	0.37658	0.71132	1.6238 ↑	0.6994	0.29394	0.54791	1.151 ↑	0.203
SYMMETRIC DIMETHYLARGININE_pos_1	0.5148	0.95335	1.8951 ↑	0.9223	0.20869	0.6246	0.81162 ↓	-0.301	0.4536	0.90445	0.98104 ↓	-0.028	0.85917	0.94843	0.88771 ↓	-0.172	0.14005	0.38391	0.84484 ↓	-0.243
TAG566-FA160	0.51593	0.95335	1.2503 ↑	0.3223	0.56508	0.81005	0.87444 ↓	-0.194	0.40958	0.90445	1.0414 ↑	0.0586	0.86886	0.94947	1.1118 ↑	0.1529	0.59464	0.77285	1.0535 ↑	0.0751
TAG541-FA160	0.52339	0.96294	0.95809 ↓	-0.062	0.013057	0.29385	0.74725 ↓	-0.42	0.36042	0.89952	0.84966 ↓	-0.235	0.021839	0.28126	0.63261 ↓	-0.661	0.16141	0.39883	0.75021 ↓	-0.415
TAG531-FA160	0.53336	0.97317	1.0105 ↑	0.0151	0.25889	0.67444	0.86126 ↓	-0.215	0.61572	0.91609	0.96185 ↓	-0.056	0.57499	0.82003	1.0115 ↑	0.0165	0.75666	0.88038	1.0695 ↑	0.0969
TAG492-FA140	0.53352	0.97317	0.96519 ↓	-0.051	0.2793	0.68615	0.78918 ↓	-0.342	0.087443	0.67934	0.7488 ↓	-0.417	0.4416	0.74968	1.0992 ↑	0.1365	0.014151	0.097001	1.241 ↑	0.3115
TAG521-FA160	0.54356	0.98038	1.1248 ↑	0.1697	0.45334	0.78641	0.81048 ↓	-0.303	0.76949	0.92465	0.89847 ↓	-0.154	0.045425	0.32176	0.66945 ↓	-0.579	0.00519	0.061271	0.56059 ↓	-0.835
TAG472-FA181	0.54431	0.98038	0.96563 ↓	-0.05	0.069385	0.39187	1.1547 ↑	0.2075	0.72538	0.92465	0.97859 ↓	-0.031	0.060316	0.33463	1.152 ↑	0.2041	0.11263	0.33764	1.1847 ↑	0.2445
TAG440-FA140	0.5444	0.98038	0.97243 ↓	-0.04	0.58794	0.82143	1.0078 ↑	0.0113	0.23652	0.85757	1.0976 ↑	0.1343	0.065515	0.33956	1.0983 ↑	0.1353	0.011354	0.085834	1.2047 ↑	0.2687
TAG543-FA181	0.55003	0.98115	0.98402 ↓	-0.023	0.48173	0.789	0.88079 ↓	-0.183	0.23572	0.85757	0.67161 ↓	-0.574	0.007428	0.24008	0.61987 ↓	-0.69	0.23857	0.49461	0.80734 ↓	-0.309
TAG562-FA180	0.55205	0.98115	1.0138 ↑	0.0198	0.18642	0.61799	0.76515 ↓	-0.386	0.73038	0.92465	0.84571 ↓	-0.242	0.016712	0.28126	0.62733 ↓	-0.673	0.080749	0.2813	0.6691 ↓	-0.58
TAG500-FA180	0.56146	0.98115	0.98511 ↓	-0.022	0.20006	0.61799	1.1704 ↑	0.227	0.95173	0.97945	0.90652 ↓	-0.142	0.97881	0.99046	1.0215 ↑	0.0307	0.87374	0.94729	1.0276 ↑	0.0393
TAG491-FA170	0.56368	0.98115	0.98547 ↓	-0.021	0.43896	0.78535	0.8254 ↓	-0.277	0.43225	0.90445	0.83081 ↓	-0.267	0.17848	0.53419	1.1277 ↑	0.1734	0.85454	0.93823	1.0018 ↑	0.0026
MEVALONOLACTONE_pos_2	0.56601	0.98115	1.1693 ↑	0.2256	0.26819	0.67444	1.0845 ↑	0.117	0.066789	0.65022	1.3704 ↑	0.4546	0.4487	0.75374	1.261 ↑	0.3346	0.29369	0.54791	1.1896 ↑	0.2505
TAG521-FA181	0.57249	0.98115	1.1523 ↑	0.2046	0.78968	0.9346	0.89326 ↓	-0.163	0.8053	0.93257	0.88312 ↓	-0.179	0.047275	0.32406	0.70872 ↓	-0.497	0.003238	0.047473	0.5925 ↓	-0.755
CITRACONIC ACID_neg	0.57629	0.98115	0.99006 ↓	-0.014	0.86109	0.94333	0.96458 ↓	-0.052	0.41357	0.90445	1.053 ↑	0.0745	0.78082	0.90218	0.59135 ↓	-0.758	0.97551	0.99159	0.9476 ↓	-0.078
TAG524-FA160	0.57786	0.98115	0.67618 ↓	-0.565	0.54668	0.80863	1.2035 ↑	0.2672	0.58445	0.91609	0.7187 ↓	-0.477	0.23864	0.59631	0.74104 ↓	-0.432	0.30726	0.56686	1.0394 ↑	0.0558
TAG540-FA160	0.57899	0.98115	0.84424 ↓	-0.244	0.74174	0.91568	0.96856 ↓	-0.046	0.71133	0.92337	0.89204 ↓	-0.165	0.47105	0.76703	0.93288 ↓	-0.1	0.35527	0.60396	1.1099 ↑	0.1504
PROPIONYL-COA_pos_1	0.57978	0.98115	1.3873 ↑	0.4722	0.70685	0.89408	1.2159 ↑	0.282	0.24799	0.85757	1.977 ↑	0.9833	0.12192	0.45854	1.5185 ↑	0.6027	0.88418	0.94759	0.73634 ↓	-0.442
XANTHURENIC ACID_pos_2	0.58054	0.98115	2.501 ↑	1.3225	0.41845	0.77704	0.93381 ↓	-0.099	0.75874	0.92465	0.88638 ↓	-0.174	0.00715	0.24008	0.84977 ↓	-0.235	0.57993	0.76801	1.2051 ↑	0.2692
TAG547-FA182	0.58622	0.98115	0.60513 ↓	-0.725	0.43378	0.78535	1.3607 ↑	0.4443	0.7111	0.92337	0.70221 ↓	-0.51	0.77734	0.90218	1.1928 ↑	0.2543	0.000967	0.04108	1.5513 ↑	0.6335

UREIDOSUCCINIC ACID_neg_2	0.59298	0.98115	0.44815 ↓	-1.158	0.82087	0.93749	0.85035 ↓	-0.234	0.78711	0.93191	1.3692 ↑	0.4534	0.57434	0.82003	1.0257 ↑	0.0367	0.44558	0.67633	1.3337 ↑	0.4154
TAG523-FA161	0.59512	0.98115	1.1303 ↑	0.1767	0.015059	0.30477	0.69712 ↓	-0.521	0.2243	0.85757	0.63073 ↓	-0.665	0.034077	0.29777	0.46191 ↓	-1.114	0.003161	0.047473	0.50969 ↓	-0.972
TAG545-FA180	0.60102	0.98115	0.52746 ↓	-0.923	0.072341	0.39417	1.443 ↑	0.529	0.15756	0.82488	0.66463 ↓	-0.589	0.65838	0.85669	0.98218 ↓	-0.026	0.077727	0.27301	1.3155 ↑	0.3956
TAG483-FA160	0.60144	0.98115	0.91006 ↓	-0.136	0.71438	0.90093	0.93179 ↓	-0.102	0.67888	0.91633	0.84038 ↓	-0.251	0.014739	0.28126	0.68162 ↓	-0.553	0.43972	0.67466	0.86701 ↓	-0.206
TAG462-FA161	0.60539	0.98115	0.98755 ↓	-0.018	0.34512	0.7364	0.76443 ↓	-0.388	0.68117	0.91633	0.83834 ↓	-0.254	0.28198	0.62093	1.1144 ↑	0.1562	0.20451	0.45155	1.115 ↑	0.1571
TAG546-FA182	0.60557	0.98115	0.60531 ↓	-0.724	0.44809	0.78535	1.3578 ↑	0.4413	0.98333	0.99267	0.76137 ↓	-0.393	0.96978	0.98367	1.1086 ↑	0.1488	0.000897	0.04108	1.493 ↑	0.5782
TAG504-FA182	0.60635	0.98115	0.99186 ↓	-0.012	0.10542	0.46729	0.77834 ↓	-0.362	0.51997	0.91257	0.76565 ↓	-0.385	0.010396	0.25411	0.57921 ↓	-0.788	0.026855	0.15485	0.69057 ↓	-0.534
TAG541-FA180	0.60653	0.98115	1.0308 ↑	0.0437	0.84299	0.94281	0.80319 ↓	-0.316	0.42448	0.90445	1.0524 ↑	0.0737	0.10905	0.44277	0.67767 ↓	-0.561	0.002046	0.04577	0.48716 ↓	-1.038
TAG500-FA140	0.6111	0.98115	1.062 ↑	0.0868	0.22145	0.63312	1.0896 ↑	0.1238	0.41444	0.90445	1.0697 ↑	0.0971	0.15583	0.52981	1.0879 ↑	0.1216	0.11159	0.33764	1.0987 ↑	0.1358
HCER201	0.61289	0.98115	1.033 ↑	0.0468	0.033079	0.33653	1.4373 ↑	0.5234	0.041428	0.60889	2.2329 ↑	1.1589	0.008533	0.24008	2.5148 ↑	1.3305	0.006948	0.072023	1.3034 ↑	0.3823
SM220	0.61447	0.98115	1.0836 ↑	0.1158	0.021237	0.30837	1.5946 ↑	0.6732	0.18914	0.85516	1.2535 ↑	0.326	0.46515	0.76529	1.0113 ↑	0.0162	0.010266	0.085834	1.5649 ↑	0.646
SM240	0.61584	0.98115	0.98578 ↓	-0.021	0.009509	0.28471	1.6115 ↑	0.6884	0.13312	0.78853	1.2363 ↑	0.3061	0.55881	0.81895	0.97694 ↓	-0.034	0.31935	0.58002	1.1467 ↑	0.1975
DAG140/182	0.6177	0.98115	1.1181 ↑	0.1611	0.021928	0.30837	1.7711 ↑	0.8246	0.008301	0.50401	1.5401 ↑	0.623	0.18874	0.54255	1.2219 ↑	0.2891	0.76231	0.88038	1.059 ↑	0.0826
TAG491-FA140	0.6192	0.98115	1.0592 ↑	0.0829	0.55323	0.81005	1.017 ↑	0.0243	0.64402	0.91609	0.94004 ↓	-0.089	0.85028	0.94599	1.0182 ↑	0.026	0.75983	0.88038	1.011 ↑	0.0158
TAG545-FA181	0.62166	0.98115	0.67468 ↓	-0.568	0.43685	0.78535	1.2349 ↑	0.3044	0.17185	0.83988	0.70754 ↓	-0.499	0.15516	0.52981	0.7541 ↓	-0.407	0.23236	0.485	1.0766 ↑	0.1065
TAG545-FA182	0.62209	0.98115	0.63015 ↓	-0.666	0.54329	0.80863	1.2427 ↑	0.3135	0.59806	0.91609	0.75257 ↓	-0.41	0.25536	0.59631	0.73844 ↓	-0.437	0.22149	0.47067	1.0963 ↑	0.1326
TAG503-FA182	0.62286	0.98115	1.0174 ↑	0.0248	0.017314	0.30837	0.72147 ↓	-0.471	0.41791	0.90445	0.69385 ↓	-0.527	0.023974	0.29111	0.55477 ↓	-0.85	0.000647	0.039297	0.55221 ↓	-0.857
TAG563-FA181	0.62523	0.98115	0.97986 ↓	-0.029	0.13713	0.53963	1.1111 ↑	0.152	0.74073	0.92465	0.97835 ↓	-0.032	0.74798	0.87952	1.018 ↑	0.0257	0.68135	0.83692	1.0503 ↑	0.0708
TAG493-FA160	0.62577	0.98115	0.94476 ↓	-0.082	0.94058	0.96023	1.0503 ↑	0.0708	0.53744	0.91609	1.0999 ↑	0.1374	0.02074	0.28126	0.81094 ↓	-0.302	0.77531	0.89008	1.0824 ↑	0.1143
2-PHOSPHOGLYCERATE_pos_4	0.63729	0.98115	0.98034 ↓	-0.029	0.51926	0.79099	0.98815 ↓	-0.017	0.5518	0.91609	1.067 ↑	0.0936	0.6991	0.85996	1.0627 ↑	0.0877	0.674	0.83692	1.3109 ↑	0.3906
TAG589-FA226	0.64192	0.98115	0.99638 ↓	-0.005	0.89106	0.94333	0.94108 ↓	-0.088	0.13149	0.78853	0.65415 ↓	-0.612	0.4903	0.78044	1.0247 ↑	0.0352	0.8306	0.92592	0.91701 ↓	-0.125
TAG513-FA150	0.64405	0.98115	1.091 ↑	0.1256	0.35	0.7364	0.55643 ↓	-0.846	0.18413	0.85075	0.61738 ↓	-0.696	0.41617	0.74773	0.47097 ↓	-1.086	0.003678	0.047687	0.24047 ↓	-2.056
TAG547-FA183	0.64769	0.98115	0.63301 ↓	-0.66	0.44651	0.78535	1.3357 ↑	0.4176	0.37286	0.89952	0.70512 ↓	-0.504	0.56972	0.82003	1.2683 ↑	0.3429	0.008965	0.084673	1.4992 ↑	0.5842
TAG490-FA170	0.65006	0.98115	0.99541 ↓	-0.007	0.57172	0.81005	1.0543 ↑	0.0763	0.84654	0.94183	0.97458 ↓	-0.037	0.029858	0.29511	1.1263 ↑	0.1716	0.11599	0.33764	1.084 ↑	0.1164
TAG523-FA182	0.65221	0.98115	0.8509 ↓	-0.233	0.89692	0.94333	0.9148 ↓	-0.128	0.36257	0.89952	0.58393 ↓	-0.776	0.046474	0.32379	0.50143 ↓	-0.996	0.1154	0.33764	0.54276 ↓	-0.882
TAG460-FA140	0.65222	0.98115	0.98374 ↓	-0.024	0.48162	0.789	1.0339 ↑	0.0481	0.23097	0.85757	1.0889 ↑	0.1228	0.14409	0.51897	1.0613 ↑	0.0859	0.038597	0.18431	1.1523 ↑	0.2045
MESACONIC ACID_neg_1	0.65224	0.98115	1.0431 ↑	0.0609	0.98551	0.99017	1.0661 ↑	0.0924	0.61634	0.91609	1.1842 ↑	0.2439	0.75098	0.87952	0.48071 ↓	-1.057	0.93426	0.95908	0.71243 ↓	-0.489
TAG566-FA225	0.65615	0.98115	1.0965 ↑	0.1329	0.55882	0.81005	0.88414 ↓	-0.178	0.14473	0.82488	0.66379 ↓	-0.591	0.19021	0.54255	0.86554 ↓	-0.208	0.47725	0.68524	0.85934 ↓	-0.219
TAG522-FA161	0.6566	0.98115	1.0622 ↑	0.0871	0.045716	0.33653	0.74453 ↓	-0.426	0.47668	0.90445	0.87414 ↓	-0.194	0.048341	0.32506	0.60705 ↓	-0.72	0.02193	0.13315	0.66944 ↓	-0.579
TAG420-FA140	0.67714	0.98115	0.98225 ↓	-0.026	0.4196	0.77704	1.0293 ↑	0.0417	0.17468	0.84364	1.1457 ↑	0.1962	0.09875	0.43267	1.1055 ↑	0.1447	0.010385	0.085834	1.2145 ↑	0.2804
SM180	0.67768	0.98115	0.98556 ↓	-0.021	0.32085	0.7222	1.1584 ↑	0.2121	0.87933	0.95883	0.90337 ↓	-0.147	0.002181	0.18171	0.69114 ↓	-0.533	0.14312	0.38391	1.139 ↑	0.1877
METHIONINE_pos_1	0.67803	0.98115	1.8563 ↑	0.8924	0.44696	0.78535	0.8047 ↓	-0.313	0.093014	0.70591	0.75425 ↓	-0.407	0.9024	0.96604	1.0182 ↑	0.026	0.19642	0.44422	1.6262 ↑	0.7015
TAG544-FA182	0.67816	0.98115	0.71835 ↓	-0.477	0.88713	0.94333	1.0522 ↑	0.0734	0.32221	0.89952	0.68582 ↓	-0.544	0.054792	0.32506	0.53167 ↓	-0.911	0.63886	0.81293	0.74367 ↓	-0.427
TAG512-FA160	0.67843	0.98115	0.98463 ↓	-0.022	0.043169	0.33653	1.1731 ↑	0.2303	0.60007	0.91609	0.99511 ↓	-0.007	0.45997	0.76529	1.0923 ↑	0.1274	0.32246	0.58318	1.0917 ↑	0.1266
TAG440-FA160	0.67922	0.98115	0.99452 ↓	-0.008	0.17362	0.61491	1.087 ↑	0.1204	0.11106	0.76666	1.1828 ↑	0.2423	0.20679	0.56224	1.0556 ↑	0.0781	0.029015	0.16172	1.1847 ↑	0.2446
CE170	0.68268	0.98115	1.0566 ↑	0.0794	0.022081	0.30837	1.3959 ↑	0.4812	0.83527	0.93937	1.0051 ↑	0.0073	0.7512	0.87952	1.1111 ↑	0.152	0.069989	0.25435	1.35 ↑	0.4329
DAG181/181	0.68798	0.98115	0.93365 ↓	-0.099	0.010738	0.28524	1.2205 ↑	0.2875	0.61576	0.91609	0.96028 ↓	-0.058	0.73252	0.8745	1.0422 ↑	0.0596	0.075394	0.27155	1.1193 ↑	0.1627
TAG554-FA181	0.68827	0.98115	0.94919 ↓	-0.075	0.19171	0.61799	1.2308 ↑	0.2996	0.44685	0.90445	0.81357 ↓	-0.298	0.95374	0.98367	1.0641 ↑	0.0897	0.80706	0.91467	0.96291 ↓	-0.055
TAG522-FA181	0.69021	0.98115	1.0797 ↑	0.1106	0.64413	0.85708	0.84881 ↓	-0.236	0.67206	0.91633	0.57936 ↓	-0.787	0.017138	0.28126	0.61933 ↓	-0.691	0.020505	0.12816	0.576 ↓	-0.796
ADENOSINE PHOSPHOSULFATE_neg_2	0.69947	0.98115	1.2278 ↑	0.2961	0.002002	0.21266	5.5775 ↑	2.4796	0.054762	0.65022	1.9702 ↑	0.9783	0.25882	0.60109	1.1436 ↑	0.1936	0.71809	0.85676	1.3653 ↑	0.4492
4-IMIDAZOLEACETATE_pos_1	0.69968	0.98115	1.1663 ↑	0.2219	0.18839	0.61799	0.87095 ↓	-0.199	0.004489	0.38156	0.76007 ↓	-0.396	0.42649	0.7494	0.71689 ↓	-0.48	0.69934	0.84685	1.2316 ↑	0.3006
ALLANTOIN_neg_2	0.7071	0.98115	0.88452 ↓	-0.177	0.74332	0.91568	1.1341 ↑	0.1815	0.12186	0.78472	0.87871 ↓	-0.187	0.11636	0.45371	1.4641 ↑	0.5501	0.83542	0.92651	1.1241 ↑	0.1688
CE141	0.70904	0.98115	0.95215 ↓	-0.071	0.44903	0.78535	1.1646 ↑	0.2198	0.9518	0.97945	0.83097 ↓	-0.267	0.35688	0.70254	1.1073 ↑	0.1471	0.2875	0.54065	1.1063 ↑	0.1457
TAG511-FA180	0.7117	0.98115	1.0317 ↑	0.045	0.39122	0.77199	0.87819 ↓	-0.187	0.4036	0.90445	1.0073 ↑	0.0105	0.98379	0.99314	0.93583 ↓	-0.096	0.016893	0.11218	0.7609 ↓	-0.394
TAG543-FA182	0.71367	0.98115	0.86934 ↓	-0.202	0.86633	0.94333	0.84612 ↓	-0.241	0.64428	0.91609	0.77309 ↓	-0.371	0.0566	0.32639	0.44011 ↓	-1.184	0.068358	0.25435	0.48629 ↓	-1.04
RIBOFLAVIN_pos_1	0.71546	0.98115	0.79667 ↓	-0.328	0.034836	0.33653	1.531 ↑	0.6145	0.6729	0.91633	1.1958 ↑	0.2579	0.37076	0.70986	1.114 ↑	0.1557	0.70003	0.84685	1.155 ↑	0.2079
TAG502-FA140	0.71583	0.98115	1.0623 ↑	0.0872	0.51291	0.79092	0.95095 ↓	-0.073	0.56936	0.91609	0.92354 ↓	-0.115	0.16064	0.53303	0.84027 ↓	-0.251	0.010537	0.085834	0.83978 ↓	-0.252
TAG564-FA182	0.71816	0.98115	0.84285 ↓	-0.247	0.40518	0.77199	0.78743 ↓	-0.345	0.26973	0.87508	0.67718 ↓	-0.562	0.06181	0.33463	0.31996 ↓	-1.644	0.74664	0.87416	0.60738 ↓	-0.719
TAG510-FA180	0.72027	0.98115	1.0129 ↑	0.0185	0.39913	0.77199	1.049 ↑	0.069	0.76502	0.92465	1.0102 ↑	0.0147	0.70417	0.85997	1.0212 ↑	0.0303	0.87746	0.94759	0.98115 ↓	-0.027
DAG181/204	0.72116	0.98115	1.0097 ↑	0.0139	0.21869	0.63228	1.145 ↑	0.1954	0.97843	0.99008	0.91804 ↓	-0.123	0.68095	0.85718	1.0085 ↑	0.0122	0.93107	0.95908	1.0091 ↑	0.013
TAG563-FA201	0.72154	0.98115	1.382 ↑	0.4667	0.50777	0.79092	0.86615 ↓	-0.207	0.33227	0.89952	0.74149 ↓	-0.432	0.002993	0.18171	0.50353 ↓	-0.99	0.082133	0.28379	0.59023 ↓	-0.761
ATROLACTIC ACID_neg_2	0.72591	0.98115	1.0577 ↑	0.081	0.38302	0.76115	1.1263 ↑	0.1716	0.006208	0.43971	1.857 ↑	0.893	0.17337	0.53303	1.5078 ↑	0.5924	0.11113	0.33764	2.2496 ↑	1.1697
TAG470-FA160	0.72821	0.98115	0.98593 ↓	-0.02	0.99163	0.993	0.99158 ↓	-0.012	0.4843	0.90445	1.0065 ↑	0.0094	0.16058	0.53303	1.0659 ↑	0.092	0.20698	0.45155	1.0777 ↑	0.108
TAG524-FA181	0.72987	0.98115	0.94318 ↓	-0.084	0.84038	0.94238	0.90889 ↓	-0.138	0.12993	0.78853	0.60171 ↓	-0.733	0.016983	0.28126	0.42882 ↓	-1.222	0.018142	0.11683	0.51471 ↓	-0.958
CE160	0.73323	0.98115	0.95424 ↓	-0.068	0.003772	0.26577	1.3398 ↑	0.422	0.8435	0.94091	0.69811 ↓	-0.518	0.47954	0.77549	1.0394 ↑	0.0557	0.44415	0.67633	0.96963 ↓	-0.044
TAG451-FA150	0.7342	0.98115	0.99954 ↓	-7E-04	0.63648	0.85332	0.83726 ↓	-0.256	0.96879	0.98502	0.84742 ↓	-0.239	0.27087	0.6072	1.1078 ↑	0.1477	0.11389	0.33764	1.1525 ↑	0.2048
TAG522-FA182	0.73442	0.98115	0.86402 ↓	-0.211	0.82944	0.93768	0.8507 ↓	-0.233	0.51157	0.90566	0.79154 ↓	-0.337	0.018878	0.28126	0.49801 ↓	-1.006	0.031038	0.16285	0.49628 ↓	-1.011
TAG546-FA183	0.73448	0.98115	0.56363 ↓	-0.827	0.52794	0.79566	1.3124 ↑	0.3922	0.54931	0.91609	0.73848 ↓	-0.437	0.96511	0.98367	1.1403 ↑	0.1894	0.003141	0.047473	1.3886 ↑	0.4737
TAG483-FA161	0.73503	0.98115	1.0016 ↑	0.0023	0.46639	0.789	0.85228 ↓	-0.231	0.35561	0.89952	0.82883 ↓	-0.271	0.96955	0.98367	0.97683 ↓	-0.034	0.57619	0.76766	1.0745 ↑	0.1037
TAG553-FA181	0.7428	0.98115	0.96516 ↓	-0.051	0.20355	0.61799	1.1543 ↑	0.2071	0.1047	0.74163	0.82848 ↓	-0.271	0.94091	0.98253	1.0214 ↑	0.0306	0.25701	0.50803	0.93376 ↓	-0.099
MALONYL-COA_pos_1	0.7428	0.98115	0.94135 ↓	-0.087	0.39582	0.77199	0.98274 ↓	-0.025	0.68053	0.91633	1.1401 ↑	0.1892	0.35821	0.70254	0.72736 ↓	-0.459	0.46947	0.68345	1.3387 ↑	0.4208
TAG587-FA182	0.74666	0.98115	0.97654 ↓	-0.034	0.60076	0.82897	1.0489 ↑	0.0689	0.71697	0.92337	0.84236 ↓	-0.247	0.32342	0.66084	0.8697 ↓	-0.201	0.069128	0.25435	0.70592 ↓	-0.502
TAG533-FA170	0.74764	0.98115	1.0093 ↑	0.0134	0.8457	0.94333	1.0196 ↑	0.028	0.47721	0.90445	0.85454 ↓	-0.227	0.38875	0.71215	0.95878 ↓	-0.061	0.14274	0.38391	0.81013 ↓	-0.304
TAG512-FA161	0.74835	0.98115	1.0658 ↑	0.0919	0.086645	0.44766	0.79989 ↓	-0.322	0.66013	0.91609	0.9434 ↓	-0.084	0.17618	0.53303	1.1762 ↑	0.2342	0.16457	0.39967	1.2158 ↑	0.2819
TAG463-FA141	0.74888	0.98115	1.0071 ↑	0.0102	0.33954	0.7325	0.85787 ↓	-0.221	0.17173	0.83988	0.77155 ↓	-0.374	0.56743	0.82003	0.94095 ↓	-0.088	0.9162	0.95558	0.97696 ↓	-0.034
TAG491-FA161	0.7548	0.98115	1.037 ↑	0.0524	0.18977	0.61799	0.78876 ↓	-0.342	0.5653	0.91609	0.80755 ↓	-0.308	0.25117	0.59631	1.1204 ↑	0.164	0.87343	0.94729	0.98433 ↓	-0.023
TAG460-FA160	0.75693	0.98115	0.99431 ↓	-0.008	0.42791	0.7808	1.0386 ↑	0.0547	0.42328	0.90445	1.0505 ↑	0.071	0.10788	0.44277	1.0682 ↑	0.0951	0.089601	0.2975	1.1012 ↑	0.139
TAG542-FA180	0.75853	0.98115	0.98636 ↓	-0.02	0.87453	0.94333	0.80929 ↓	-0.305	0.81705	0.93449	0.84738 ↓	-0.239	0.026415	0.29511	0.57284 ↓	-0.804	0.017653	0.11542	0.48812 ↓	-1.035
TAG490-FA160	0.76016	0.98115	1.0274 ↑	0.039	0.6248	0.85109	0.97331 ↓	-0.039	0.64305	0.91609	0.99411 ↓	-0.009	0.16611	0.53303	1.0606 ↑	0.0848	0.24904	0.50182	1.0772 ↑	0.1073
TAG462-FA141	0.76472	0.98115	0.99932 ↓	-1E-03	0.5074	0.79092	0.85583 ↓	-0.225	0.83991	0.93937	0.90253 ↓	-0.148	0.53438	0.80536	1.0457 ↑	0.0644	0.15993	0.39761	1.0708 ↑	0.0987
TAG561-FA181	0.76634	0.98115	0.99024 ↓	-0.014	0.67737	0.87102	1.0253 ↑	0.036	0.73428	0.92465	0.93589 ↓	-0.096	0.72748	0.87093	0.99894 ↓	-0.002	0.98615	0.99316	1.0066 ↑	0.0095
CER240	0.76904	0.98115	0.92674 ↓	-0.11	0.045926	0.33653	0.39069 ↓	-1.356	0.50589	0.90566	0.80961 ↓	-0.305	0.054164	0.32506	0.32784 ↓	-1.609	0.002018	0.04577	0.1063 ↓	-3.234
TAG523-FA160	0.77681	0.98115	0.82564 ↓	-0.276	0.91437	0.94782	0.93499 ↓	-0.097	0.43482	0.90445	0.58683 ↓	-0.769	0.06198	0.33463	0.53198 ↓	-0.911	0.20824	0.45155	0.58137 ↓	-0.782
MYOINOSITOL_neg_3	0.7779	0.98115	0.74136 ↓	-0.432	0.37816	0.76115	0.8408 ↓	-0.25	0.045845	0.60889	0.71157 ↓	-0.491	0.48561	0.78044	0.73022 ↓	-0.454	0.95404	0.97468	0.89068 ↓	-0.167
FFA160	0.78351	0.98115	1.0576 ↑	0.0808	0.36748	0.75086	1.17 ↑	0.2266	0.99592	0.99592	0.83097 ↓	-0.267	0.34419	0.69327	1.4861 ↑	0.5715	0.47339	0.68503	1.1107 ↑	0.1515
TAG532-FA170	0.78373	0.98115	1.1313 ↑	0.178	0.77485	0.9346	0.99819 ↓	-0.003	0.49524	0.90528	0.89144 ↓	-0.166	0.65468	0.85669	0.99615 ↓	-0.006	0.064816	0.24489	0.80755 ↓	-0.308
FFA161	0.78517	0.98115	1.01 ↑	0.0144	0.052564	0.34545	0.60523 ↓	-0.724	0.93134	0.96978	0.83831 ↓	-0.254	0.80372	0.90846	0.83624 ↓	-0.258	0.19456	0.44422	1.4324 ↑	0.5184
TAG510-FA170	0.78568	0.98115	1.0086 ↑	0.0124	0.9539	0.96297	1.0411 ↑	0.0582	0.9201	0.96978	0.99111 ↓	-0.013	0.29197	0.62989	1.0533 ↑	0.0749	0.38678	0.62981	1.0466 ↑	0.0657
TAG501-FA161	0.78666	0.98115	1.015 ↑	0.0215	0.21743	0.63228	0.81397 ↓	-0.297	0.613	0.91609	0.81637 ↓	-0.293	0.29556	0.63441	0.82793 ↓	-0.272	0.004266	0.053323	0.79501 ↓	-0.331
TAG522-FA160	0.78753	0.98115	1.0267 ↑	0.038	0.56005	0.81005	0.82368 ↓	-0.28	0.61039	0.91609	0.60871 ↓	-0.716	0.021356	0.28126	0.60498 ↓	-0.725	0.043786	0.18989	0.56185 ↓	-0.832
DAG160/182	0.79027	0.98115	0.81062 ↓	-0.303	0.15403	0.56435	1.239 ↑	0.3092	0.31118	0.8936	0.92674 ↓	-0.11	0.60656	0.834	1.0315 ↑	0.0448	0.036034	0.17603	1.195 ↑	0.257
TAG492-FA160	0.79125	0.98115	1.0095 ↑	0.0137	0.038688	0.33653	0.83077 ↓	-0.267	0.3475	0.89952	0.91248 ↓	-0.132	0.11922	0.45744	0.87106 ↓	-0.199	7.93E-05	0.020709	0.76367 ↓	-0.389
TAG491-FA150	0.7915	0.98115	1.0128 ↑	0.0183	0.045036	0.33653	0.7878 ↓	-0.344	0.36917	0.89952	0.81377 ↓	-0.297	0.22208	0.57251	0.90041 ↓	-0.151	0.003463	0.047473	0.79245 ↓	-0.336
TAG463-FA161	0.79175	0.98115	1.0711 ↑	0.099	0.79743	0.9346	0.89474 ↓	-0.16	0.59478	0.91609	0.84009 ↓	-0.251	0.93141	0.98225	1.0049 ↑	0.0071	0.47589	0.68524	1.0429 ↑	0.0606
TAG525-FA183	0.79397	0.98115	0.62283 ↓	-0.683	0.47829	0.789	1.2773 ↑	0.3531	0.20325	0.85757	0.57372 ↓	-0.802	0.10247	0.44277	0.85379 ↓	-0.228	0.002836	0.047473	1.4786 ↑	0.5642
SM241	0.79636	0.98115	0.80782 ↓	-0.308	0.000231	0.097996	1.6321 ↑	0.7067	0.81175	0.93357	0.91915 ↓	-0.122	0.35871	0.70254	1.2876 ↑	0.3646	0.89021	0.94759	1.1624 ↑	0.2171
TAG450-FA160	0.79791	0.98115	1.0081 ↑	0.0116	0.77971	0.9346	1.0026 ↑	0.0037	0.44389	0.90445	1.013 ↑	0.0187	0.10318	0.44277	1.0853 ↑	0.1181	0.059771	0.23965	1.1359 ↑	0.1838
TAG493-FA161	0.7992	0.98115	0.99636 ↓	-0.005	0.07149	0.39417	0.79813 ↓	-0.325	0.74335	0.92465	0.83951 ↓	-0.252	0.47989	0.77549	0.96739 ↓	-0.048	0.15998	0.39761	0.9304 ↓	-0.104
TAG460-FA120	0.79931	0.98115	0.96511 ↓	-0.051	0.26283	0.67444	1.0856 ↑	0.1185	0.25227	0.85757	1.1939 ↑	0.2557	0.66051	0.85669	1.0396 ↑	0.0561	0.052394	0.21831	1.1429 ↑	0.1928
TAG480-FA140	0.80217	0.98115	1.0127 ↑	0.0182	0.20246	0.61799	1.0821 ↑	0.1138	0.35287	0.89952	1.0901 ↑	0.1244	0.67019	0.85669	1.0107 ↑	0.0153	0.44876	0.67752	1.0664 ↑	0.0928
TAG589-FA181	0.80506	0.98115	1.0109 ↑	0.0156	0.993	0.993	1.0183 ↑	0.0261	0.75404	0.92465	0.90926 ↓	-0.137	0.24135	0.59631	0.79955 ↓	-0.323	0.030146	0.16195	0.65523 ↓	-0.61
DAG181/182	0.80528	0.98115	0.78497 ↓	-0.349	0.22613	0.64069	1.1355 ↑	0.1833	0.81275	0.93357	1.007 ↑	0.01	0.54128	0.80717	1.075 ↑	0.1044	0.11465	0.33764	1.1069 ↑	0.1466
TAG568-FA204	0.80643	0.98115	1.0297 ↑	0.0422	0.10551	0.46729	1.1401 ↑	0.1892	0.22486	0.85757	1.07 ↑	0.0976	0.90613	0.9676	0.99639 ↓	-0.005	0.44365	0.67633	1.608 ↑	0.6853
TAG512-FA181	0.80698	0.98115	1.0011 ↑	0.0016	0.24916	0.67022	1.0847 ↑	0.1174	0.3046	0.8936	0.9281 ↓	-0.108	0.96756	0.98367	1.0169 ↑	0.0241	0.71967	0.85676	1.0043 ↑	0.0062
TAG441-FA141	0.81043	0.98115	1.0389 ↑	0.055	0.94216	0.96023	0.87507 ↓	-0.193	0.46464	0.90445	0.99738 ↓	-0.004	0.14813	0.52121	1.1364 ↑	0.1845	0.45675	0.67752	1.0178 ↑	0.0255
TAG562-FA160	0.81109	0.98115	0.99413 ↓	-0.008	0.83399	0.93768	0.79631 ↓	-0.329	0.071761	0.67774	0.75166 ↓	-0.412	0.18487	0.54197	0.50931 ↓	-0.973	0.14538	0.38391	0.60162 ↓	-0.733
DAG160/204	0.81518	0.98115	1.0756 ↑	0.1051	0.076959	0.41402	1.3407 ↑	0.423	0.045264	0.60889	1.298 ↑	0.3763	0.55296	0.81884	1.0486 ↑	0.0685	0.16924	0.40638	1.4065 ↑	0.4921
TAG503-FA160	0.81691	0.98115	1.0037 ↑	0.0054	0.03581	0.33653	0.69274 ↓	-0.53	0.27891	0.88713	0.64151 ↓	-0.64	0.004981	0.23331	0.379 ↓	-1.4	0.008784	0.084673	0.46517 ↓	-1.104
TAG522-FA180	0.82031	0.98115	0.95049 ↓	-0.073	0.90525	0.94333	0.79873 ↓	-0.324	0.59888	0.91609	0.85059 ↓	-0.233	0.017931	0.28126	0.4416 ↓	-1.179	0.011149	0.085834	0.41497 ↓	-1.269
TAG542-FA160	0.8238	0.98115	1.0005 ↑	0.0008	0.047437	0.33909	0.7047 ↓	-0.505	0.3748	0.89952	0.75802 ↓	-0.4	0.008545	0.24008	0.49587 ↓	-1.012	0.17416	0.41584	0.63035 ↓	-0.666
TAG501-FA140	0.82428	0.98115	1.0301 ↑	0.0427	0.26705	0.67444	0.94849 ↓	-0.076	0.24382	0.85757	1.2401 ↑	0.3105	0.36502	0.70838	0.91096 ↓	-0.135	0.56424	0.76127	0.9615 ↓	-0.057
TAG523-FA181	0.82459	0.98115	0.87465 ↓	-0.193	0.83189	0.93768	0.88649 ↓	-0.174	0.25051	0.85757	0.55389 ↓	-0.852	0.023522	0.29111	0.47535 ↓	-1.073	0.063573	0.24489	0.52875 ↓	-0.919
TAG534-FA160	0.82498	0.98115	1.0214 ↑	0.0305	0.63213	0.85206	0.93629 ↓	-0.095	0.90491	0.96978	0.98669 ↓	-0.019	0.029674	0.29511	0.80372 ↓	-0.315	0.67935	0.83692	0.88878 ↓	-0.17
TAG542-FA182	0.82685	0.98115	0.97404 ↓	-0.038	0.19718	0.61799	1.1801 ↑	0.2389	0.43748	0.90445	0.85067 ↓	-0.233	0.2046	0.56099	0.80542 ↓	-0.312	0.006178	0.066946	0.63981 ↓	-0.644
TAG421-FA120	0.83105	0.98115	0.96874 ↓	-0.046	0.38326	0.76115	0.81774 ↓	-0.29	0.89521	0.96653	1.0039 ↑	0.0056	0.070844	0.35542	1.2116 ↑	0.2769	0.008042	0.079486	1.2779 ↑	0.3538
ANTHRANILIC ACID_pos_4	0.83369	0.98115	0.87642 ↓	-0.19	0.56536	0.81005	0.87536 ↓	-0.192	0.054551	0.65022	0.68949 ↓	-0.536	0.57122	0.82003	0.78157 ↓	-0.356	0.82106	0.92273	1.213 ↑	0.2786
L-ACETYLCARNITINE_pos_1	0.83402	0.98115	1.9162 ↑	0.9382	0.045581	0.33653	0.50849 ↓	-0.976	0.087914	0.67934	0.68857 ↓	-0.538	0.6721	0.85669	1.6203 ↑	0.6963	0.38506	0.62943	2.1658 ↑	1.1149
TAG544-FA180	0.843	0.98115	0.74598 ↓	-0.423	0.47714	0.789	1.1572 ↑	0.2106	0.15333	0.82488	0.76649 ↓	-0.384	0.065341	0.33956	0.63871 ↓	-0.647	0.72297	0.85827	0.84722 ↓	-0.239
TAG567-FA160	0.84877	0.98115	0.94526 ↓	-0.081	0.89911	0.94333	0.88429 ↓	-0.177	0.43208	0.90445	0.91392 ↓	-0.13	0.58487	0.82778	0.97617 ↓	-0.035	0.70641	0.84685	0.99381 ↓	-0.009
L-DIHYDROOROTIC ACID_neg_1	0.85025	0.98115	0.58505 ↓	-0.773	0.25712	0.67444	0.71879 ↓	-0.476	0.28557	0.8936	1.0418 ↑	0.059	0.31882	0.65459	0.94997 ↓	-0.074	0.58369	0.76801	1.2342 ↑	0.3036
TAG490-FA150	0.8507	0.98115	0.97412 ↓	-0.038	0.80975	0.9346	0.99861 ↓	-0.002	0.8009	0.93191	0.98373 ↓	-0.024	0.35734	0.70254	1.0418 ↑	0.0591	0.26746	0.52143	0.96677 ↓	-0.049
TAG587-FA160	0.85484	0.98115	0.9863 ↓	-0.02	0.73354	0.9089	1.0561 ↑	0.0788	0.77862	0.93191	0.93919 ↓	-0.091	0.70011	0.85996	1.0288 ↑	0.0409	0.062311	0.24295	1.257 ↑	0.33
CE180	0.85579	0.98115	1.0252 ↑	0.0359	0.001975	0.21266	1.4168 ↑	0.5026	0.61406	0.91609	1.0012 ↑	0.0017	0.64065	0.85097	1.0178 ↑	0.0254	0.3081	0.56686	1.0645 ↑	0.0902
TAG421-FA160	0.8572	0.98115	0.99968 ↓	-5E-04	0.17322	0.61491	1.1819 ↑	0.2411	0.084448	0.67934	1.6213 ↑	0.6972	0.57297	0.82003	1.0654 ↑	0.0914	0.20736	0.45155	1.1455 ↑	0.1959
TAG482-FA161	0.86252	0.98115	1.0312 ↑	0.0443	0.14192	0.55328	0.76104 ↓	-0.394	0.48514	0.90445	0.82329 ↓	-0.281	0.85487	0.94712	0.97487 ↓	-0.037	0.62572	0.8083	1.0339 ↑	0.048
TAG562-FA201	0.86487	0.98115	1.8047 ↑	0.8518	0.92104	0.95011	0.96964 ↓	-0.044	0.62782	0.91609	0.82763 ↓	-0.273	0.51332	0.79414	0.79482 ↓	-0.331	0.2582	0.50803	0.77869 ↓	-0.361
TAG546-FA181	0.86846	0.98115	0.58269 ↓	-0.779	0.28815	0.6895	1.305 ↑	0.3841	0.13744	0.80019	0.7073 ↓	-0.5	0.43147	0.7494	0.97537 ↓	-0.036	0.015098	0.10185	1.2856 ↑	0.3624
TAG523-FA180	0.86952	0.98115	1.0164 ↑	0.0235	0.46174	0.789	0.87996 ↓	-0.184	0.35021	0.89952	0.80564 ↓	-0.312	0.009039	0.24008	0.29922 ↓	-1.741	0.11866	0.34075	0.35571 ↓	-1.491
TAG565-FA181	0.87001	0.98115	1.3687 ↑	0.4528	0.17921	0.61799	0.4942 ↓	-1.017	0.085023	0.67934	0.57715 ↓	-0.793	0.002735	0.18171	0.23664 ↓	-2.079	0.004625	0.056161	0.20931 ↓	-2.256
TAG480-FA180	0.87197	0.98115	0.99355 ↓	-0.009	0.1432	0.55328	1.1053 ↑	0.1444	0.20591	0.85757	1.1361 ↑	0.1841	0.60058	0.83262	1.0171 ↑	0.0244	0.18489	0.43175	1.1429 ↑	0.1927
TAG521-FA180	0.87444	0.98115	1.1259 ↑	0.1711	0.87625	0.94333	0.8475 ↓	-0.239	0.62644	0.91609	0.96684 ↓	-0.049	0.043335	0.32176	0.64873 ↓	-0.624	0.001746	0.04577	0.49528 ↓	-1.014
TAG532-FA181	0.87619	0.98115	1.0011 ↑	0.0015	0.058047	0.34747	1.1526 ↑	0.205	0.82235	0.93449	0.94498 ↓	-0.082	0.51706	0.79414	1.0737 ↑	0.1026	0.76081	0.88038	1.0188 ↑	0.0269
TAG551-FA160	0.87698	0.98115	1.0833 ↑	0.1155	0.14805	0.55976	0.73536 ↓	-0.443	0.67658	0.91633	1.0455 ↑	0.0642	0.38382	0.71215	0.83954 ↓	-0.252	0.76473	0.88079	1.1393 ↑	0.1882
TAG545-FA183	0.87709	0.98115	0.65661 ↓	-0.607	0.80961	0.9346	1.1957 ↑	0.2579	0.64076	0.91609	0.73201 ↓	-0.45	0.80362	0.90846	1.0032 ↑	0.0046	0.07002	0.25435	1.2162 ↑	0.2824
BIOTIN_pos_4	0.88082	0.98115	1.1886 ↑	0.2493	0.78136	0.9346	0.98373 ↓	-0.024	0.059801	0.65022	1.3165 ↑	0.3967	0.20015	0.55631	1.2281 ↑	0.2964	0.20515	0.45155	1.2024 ↑	0.2659
TAG512-FA150	0.88181	0.98115	1.0804 ↑	0.1116	0.17588	0.61775	0.72458 ↓	-0.465	0.8664	0.95148	0.78291 ↓	-0.353	0.41549	0.74773	0.70903 ↓	-0.496	0.0034	0.047473	0.49226 ↓	-1.023
DAG161/181	0.88205	0.98115	0.89844 ↓	-0.155	0.044433	0.33653	1.3485 ↑	0.4313	0.38765	0.89952	1.1521 ↑	0.2042	0.51897	0.79414	1.1026 ↑	0.141	0.39942	0.64058	1.0154 ↑	0.0221

FFA241	0.88374	0.98115	1.0952 ↑	0.1312	0.65289	0.86323	1.1222 ↑	0.1663	0.63316	0.91609	0.78721 ↓	-0.345	0.99806	0.99876	1.1417 ↑	0.1911	0.34989	0.60203	1.1685 ↑	0.2246
METANEPHRINE_pos_1	0.88399	0.98115	1.1147 ↑	0.1567	0.82031	0.93749	0.90699 ↓	-0.141	0.01204	0.58079	1.2913 ↑	0.3688	0.9622	0.98367	1.0619 ↑	0.0867	0.14954	0.38657	0.90965 ↓	-0.137
TAG442-FA160	0.88652	0.98115	1.1433 ↑	0.1932	0.19619	0.61799	1.2234 ↑	0.2909	0.021668	0.60889	1.2421 ↑	0.3128	0.83297	0.93168	0.97032 ↓	-0.043	0.89757	0.94839	1.0686 ↑	0.0957
TAG526-FA182	0.88824	0.98115	0.89885 ↓	-0.154	0.90439	0.94333	1.0073 ↑	0.0104	0.76142	0.92465	0.92999 ↓	-0.105	0.34075	0.69155	0.88371 ↓	-0.178	0.32779	0.58966	1.1516 ↑	0.2036
TAG422-FA120	0.89128	0.98115	1.29 ↑	0.3674	0.81365	0.9346	0.96682 ↓	-0.049	0.92526	0.96978	0.8655 ↓	-0.208	0.27619	0.61135	0.92104 ↓	-0.119	0.2506	0.50182	1.1717 ↑	0.2286
N-ACETYLALANINE_pos_2	0.89477	0.98115	2.1918 ↑	1.1321	0.77844	0.9346	1.0086 ↑	0.0123	0.79124	0.93191	1.508 ↑	0.5926	0.16948	0.53303	2.7284 ↑	1.4481	0.4356	0.6732	2.1252 ↑	1.0876
TAG525-FA161	0.89573	0.98115	0.88476 ↓	-0.177	0.9288	0.95348	0.77101 ↓	-0.375	0.29398	0.8936	0.65774 ↓	-0.604	0.00511	0.23331	0.22237 ↓	-2.169	0.12831	0.36115	0.32549 ↓	-1.619
FFA181	0.89584	0.98115	1.0611 ↑	0.0856	0.49092	0.79006	1.0772 ↑	0.1073	0.93556	0.96978	1.2153 ↑	0.2813	0.087747	0.39255	1.1945 ↑	0.2564	0.86717	0.94499	1.0631 ↑	0.0883
TAG568-FA160	0.89594	0.98115	0.99601 ↓	-0.006	0.18779	0.61799	0.81477 ↓	-0.296	0.21494	0.85757	0.86181 ↓	-0.215	0.69618	0.85996	0.90857 ↓	-0.138	0.35415	0.60396	0.86355 ↓	-0.212
TAG568-FA161	0.89605	0.98115	1.1195 ↑	0.1629	0.69725	0.88988	0.92073 ↓	-0.119	0.89603	0.96653	0.91344 ↓	-0.131	0.68317	0.85718	0.93829 ↓	-0.092	0.18681	0.43384	1.202 ↑	0.2655
TAG564-FA201	0.89739	0.98115	0.87913 ↓	-0.186	0.44107	0.78535	1.029 ↑	0.0412	0.17842	0.84645	0.69517 ↓	-0.525	0.035031	0.29777	0.36769 ↓	-1.444	0.15814	0.39761	0.47312 ↓	-1.08
GLUCOSE 1-PHOSPHATE_pos_2	0.89952	0.98115	0.71107 ↓	-0.492	0.3705	0.7534	0.74737 ↓	-0.42	0.34174	0.89952	1.4404 ↑	0.5265	0.87507	0.95232	1.0076 ↑	0.0109	0.46182	0.67752	1.18 ↑	0.2388
TAG511-FA181	0.90234	0.98115	1.0318 ↑	0.0451	0.47031	0.789	0.8723 ↓	-0.197	0.75584	0.92465	0.89215 ↓	-0.165	0.25432	0.59631	0.9267 ↓	-0.11	0.000244	0.020709	0.74149 ↓	-0.432
TAG490-FA180	0.90582	0.98115	0.97617 ↓	-0.035	0.31483	0.7222	1.0759 ↑	0.1056	0.99421	0.99592	0.97891 ↓	-0.031	0.25518	0.59631	1.0446 ↑	0.063	0.57082	0.7653	0.9814 ↓	-0.027
TAG503-FA183	0.90827	0.98115	0.95644 ↓	-0.064	0.67985	0.87102	1.082 ↑	0.1137	0.34714	0.89952	0.7859 ↓	-0.348	0.67528	0.85669	0.94519 ↓	-0.081	0.21375	0.46113	1.2071 ↑	0.2716
DAG180/181	0.90914	0.98115	0.98505 ↓	-0.022	0.018791	0.30837	1.3255 ↑	0.4066	0.25163	0.85757	1.0931 ↑	0.1284	0.24638	0.59631	1.1187 ↑	0.1618	0.1324	0.37018	1.0886 ↑	0.1225
TAG568-FA205	0.91259	0.98115	0.98669 ↓	-0.019	0.54182	0.80863	1.3138 ↑	0.3937	0.47378	0.90445	0.74794 ↓	-0.419	0.10571	0.44277	0.88584 ↓	-0.175	0.57413	0.76731	0.94468 ↓	-0.082
TAG461-FA161	0.91284	0.98115	1.016 ↑	0.0229	0.35396	0.74053	0.79028 ↓	-0.34	0.93788	0.96983	0.87858 ↓	-0.187	0.25471	0.59631	1.1152 ↑	0.1573	0.14457	0.38391	1.0936 ↑	0.1291
CHOLINE_pos_1	0.91406	0.98115	0.92005 ↓	-0.12	0.458	0.789	0.82657 ↓	-0.275	0.96664	0.98502	1.2704 ↑	0.3453	0.46818	0.76529	0.70578 ↓	-0.503	0.14634	0.38391	1.411 ↑	0.4967
TAG481-FA180	0.91464	0.98115	1.0187 ↑	0.0267	0.50124	0.79092	1.048 ↑	0.0676	0.037043	0.60889	1.2735 ↑	0.3488	0.60129	0.83262	1.0457 ↑	0.0645	0.66606	0.83503	0.99265 ↓	-0.011
TAG541-FA181	0.91496	0.98115	1.011 ↑	0.0158	0.054915	0.34545	1.1683 ↑	0.2245	0.46503	0.90445	1.0168 ↑	0.024	0.92212	0.97742	1.0368 ↑	0.0521	0.51153	0.70939	1.0598 ↑	0.0838
TAG491-FA181	0.92159	0.98115	1.0123 ↑	0.0177	0.088337	0.44766	0.837 ↓	-0.257	0.81127	0.93357	0.89949 ↓	-0.153	0.44817	0.75374	0.94705 ↓	-0.078	0.000388	0.027453	0.8245 ↓	-0.278
TAG544-FA181	0.92228	0.98115	0.74743 ↓	-0.42	0.63091	0.85206	1.0515 ↑	0.0725	0.21826	0.85757	0.65672 ↓	-0.607	0.03785	0.30935	0.55272 ↓	-0.855	0.42874	0.66746	0.70742 ↓	-0.499
TAG471-FA181	0.92398	0.98115	1.0322 ↑	0.0458	0.83384	0.93768	1.0053 ↑	0.0076	0.77013	0.92465	0.92143 ↓	-0.118	0.032795	0.29777	1.1363 ↑	0.1844	0.14609	0.38391	1.1117 ↑	0.1528
TAG525-FA182	0.92457	0.98115	0.688 ↓	-0.54	0.78775	0.9346	1.0736 ↑	0.1025	0.33221	0.89952	0.71086 ↓	-0.492	0.052414	0.32506	0.47672 ↓	-1.069	0.43806	0.67455	0.73416 ↓	-0.446
TAG461-FA140	0.92716	0.98115	1.0163 ↑	0.0234	0.33819	0.7325	0.84111 ↓	-0.25	0.52822	0.91257	0.99816 ↓	-0.003	0.60144	0.83262	1.06 ↑	0.084	0.064309	0.24489	1.1139 ↑	0.1556
TAG501-FA180	0.92806	0.98115	1.0415 ↑	0.0587	0.71774	0.90248	0.94004 ↓	-0.089	0.39017	0.89952	1.1567 ↑	0.21	0.11596	0.45371	0.80321 ↓	-0.316	0.001451	0.04577	0.74649 ↓	-0.422
TAG463-FA181	0.93194	0.98281	0.9863 ↓	-0.02	0.66367	0.87102	1.096 ↑	0.1322	0.32419	0.89952	1.1909 ↑	0.2521	0.59082	0.8287	1.0573 ↑	0.0804	0.039999	0.1866	1.3668 ↑	0.4508
2-HYDROXY-3-METHYLBUTYRIC ACID_neg	0.94943	0.98793	1.1316 ↑	0.1783	0.39513	0.77199	0.40374 ↓	-1.309	0.25425	0.85757	1.2663 ↑	0.3407	0.62473	0.84022	0.34679 ↓	-1.528	0.5666	0.76205	1.0085 ↑	0.0122
FFA226	0.95325	0.98793	1.0942 ↑	0.1299	0.20811	0.6246	1.1604 ↑	0.2146	0.20373	0.85757	0.98738 ↓	-0.018	0.91392	0.97347	1.0185 ↑	0.0264	0.27627	0.52418	1.0875 ↑	0.1211
TAG462-FA160	0.95386	0.98793	1.0837 ↑	0.116	0.33353	0.7325	0.89611 ↓	-0.158	0.71011	0.92337	1.0025 ↑	0.0036	0.73982	0.87828	0.97263 ↓	-0.04	0.2328	0.485	1.0882 ↑	0.1219
TAG564-FA204	0.95435	0.98793	1.026 ↑	0.037	0.33654	0.7325	1.4617 ↑	0.5477	0.93184	0.96978	0.74301 ↓	-0.429	0.69833	0.85996	0.95039 ↓	-0.073	0.49752	0.70482	0.83622 ↓	-0.258
TAG442-FA140	0.96042	0.98793	1.1865 ↑	0.2467	0.95361	0.96297	0.94024 ↓	-0.089	0.93486	0.96978	0.89519 ↓	-0.16	0.21818	0.57251	1.057 ↑	0.0799	0.15746	0.39761	1.1702 ↑	0.2268
TAG492-FA150	0.96374	0.98793	1.0153 ↑	0.0219	0.078193	0.4154	0.73311 ↓	-0.448	0.716	0.92337	0.8135 ↓	-0.298	0.21783	0.57251	0.83529 ↓	-0.26	0.000244	0.020709	0.70434 ↓	-0.506
TAG481-FA141	0.96424	0.98793	1.0548 ↑	0.077	0.051238	0.34545	0.75333 ↓	-0.409	0.41852	0.90445	1.0332 ↑	0.0471	0.54757	0.8137	0.96356 ↓	-0.054	0.050887	0.21413	1.1907 ↑	0.2519
TAG460-FA180	0.96429	0.98793	1.0027 ↑	0.0038	0.056897	0.34545	1.1661 ↑	0.2216	0.067317	0.65022	1.3119 ↑	0.3917	0.43553	0.7494	1.0415 ↑	0.0587	0.11528	0.33764	1.115 ↑	0.157
TAG461-FA160	0.96662	0.98793	1.0138 ↑	0.0198	0.42396	0.78001	0.87765 ↓	-0.188	0.61817	0.91609	1.0132 ↑	0.0189	0.61008	0.834	1.0502 ↑	0.0707	0.076869	0.27224	1.1086 ↑	0.1488
TAG471-FA160	0.96748	0.98793	1.0141 ↑	0.0202	0.37842	0.76115	0.84136 ↓	-0.249	0.91932	0.96978	0.88793 ↓	-0.171	0.28513	0.62143	1.0908 ↑	0.1254	0.34113	0.60156	1.0717 ↑	0.0998
TAG470-FA150	0.96856	0.98793	1.0112 ↑	0.016	0.80654	0.9346	0.97472 ↓	-0.037	0.76185	0.92465	0.97803 ↓	-0.032	0.033732	0.29777	1.1213 ↑	0.1652	0.53543	0.73883	1.0216 ↑	0.0308
TAG543-FA161	0.96916	0.98793	1.087 ↑	0.1204	0.58984	0.82143	0.82048 ↓	-0.285	0.23431	0.85757	0.9034 ↓	-0.147	0.081589	0.38105	0.61704 ↓	-0.697	0.70609	0.84685	0.8631 ↓	-0.212
TAG530-FA160	0.96984	0.98793	0.986 ↓	-0.02	0.42051	0.77704	1.0665 ↑	0.0929	0.4736	0.90445	0.85203 ↓	-0.231	0.87614	0.95232	0.99467 ↓	-0.008	0.70737	0.84685	1.0324 ↑	0.046
NADPH_pos_1	0.97083	0.98793	1.1645 ↑	0.2197	0.3566	0.74053	0.80728 ↓	-0.309	0.094919	0.70773	2.2022 ↑	1.139	0.7128	0.86555	1.5271 ↑	0.6108	0.45864	0.67752	0.94526 ↓	-0.081
FFA201	0.97233	0.98793	1.127 ↑	0.1725	0.047872	0.33909	1.2983 ↑	0.3766	0.3424	0.89952	1.0023 ↑	0.0033	0.16667	0.53303	1.1851 ↑	0.245	0.2974	0.55195	1.1567 ↑	0.21
TAG543-FA180	0.97656	0.98793	0.90367 ↓	-0.146	0.79039	0.9346	0.90848 ↓	-0.138	0.49728	0.90528	0.7878 ↓	-0.344	0.029063	0.29511	0.5167 ↓	-0.953	0.055193	0.22555	0.5289 ↓	-0.919
TAG525-FA160	0.97989	0.98793	0.61567 ↓	-0.7	0.77408	0.9346	1.2798 ↑	0.3559	0.24438	0.85757	0.6651 ↓	-0.588	0.56436	0.82003	0.89716 ↓	-0.157	0.040614	0.1866	1.3664 ↑	0.4504
TAG461-FA181	0.98014	0.98793	1.0314 ↑	0.0447	0.70271	0.8915	1.0442 ↑	0.0624	0.19953	0.85757	1.3021 ↑	0.3809	0.69328	0.85996	1.0528 ↑	0.0742	0.045878	0.19695	1.2302 ↑	0.2988
CE161	0.98095	0.98793	1.0165 ↑	0.0236	0.38084	0.76115	1.1043 ↑	0.1432	0.14971	0.82488	0.44597 ↓	-1.165	0.24637	0.59631	1.1481 ↑	0.1993	0.8993	0.94839	0.91931 ↓	-0.121
TAG542-FA181	0.99011	0.99423	1.0341 ↑	0.0484	0.68042	0.87102	0.82076 ↓	-0.285	0.92344	0.96978	0.81385 ↓	-0.297	0.032683	0.29777	0.59788 ↓	-0.742	0.033082	0.16541	0.56155 ↓	-0.833
TAG492-FA161	0.99189	0.99423	1.013 ↑	0.0186	0.13201	0.53672	0.77049 ↓	-0.376	0.43623	0.90445	0.79555 ↓	-0.33	0.46423	0.76529	1.0732 ↑	0.1019	0.69485	0.84616	0.98362 ↓	-0.024
TAG531-FA170	0.996	0.996	1.03 ↑	0.0426	0.72874	0.90826	0.98568 ↓	-0.021	0.88779	0.96338	0.95065 ↓	-0.073	0.63534	0.84912	1.0021 ↑	0.003	0.31519	0.5758	0.92231 ↓	-0.117

Supplementary Table S2A. List of dysregulated metabolites for female, male, Wild type and APCHi mice following exposure to 9.5 Gy of Gamma-radiation, 24 h post-irradiation showing penotype dependent response of radiation.

	9.5Gy / 0.0Gy				9.5Gy / 0.0Gy				9.5Gy / 0.0Gy				9.5Gy / 0.0Gy			
	Female				Male				WildType				APCHi			
	24h															
name	p-value	FDR	FC	LOG2FC	p-value	FDR	FC	LOG2FC	p-value	FDR	FC	LOG2FC	p-value	FDR	FC	LOG2FC
XANTHOSINE_neg_1	6.97E-07	0.000148	3.3658 ↑	1.7509	0.005664	0.2407	2.4211 ↑	1.2756	0.009274	0.42014	2.3964 ↑	1.2609	0.015143	0.52739	2.6491 ↑	1.4055
CYSTEINE_neg_5	0.002393	0.16951	5.9315 ↑	2.5684	0.043996	0.58433	1.391 ↑	0.4761	0.001529	0.27225	2.6901 ↑	1.4277	0.023941	0.55845	1.9966 ↑	0.9976
PYROPHOSPHATE_neg_3	0.004755	0.25259	0.20202 ↓	-2.307	0.46392	0.85354	0.52654 ↓	-0.925	0.46008	0.93557	0.12072 ↓	-3.05	0.006983	0.42218	0.13069 ↓	-2.936
N-ACETYLGLUTAMINE_pos_2	0.006909	0.29364	2.0563 ↑	1.0401	0.008954	0.31711	2.5052 ↑	1.3249	0.03033	0.51706	2.3156 ↑	1.2114	0.018711	0.55845	2.1597 ↑	1.1108
INDOLE-3-CARBOXYLIC ACID_neg_1	0.027135	0.697	2.729 ↑	1.4484	0.004853	0.2407	1.8434 ↑	0.8824	0.031632	0.51706	1.654 ↑	0.726	0.001499	0.21242	3.1033 ↑	1.6338
CE204	0.035713	0.79883	1.3368 ↑	0.4187	0.19916	0.72412	1.2204 ↑	0.2873	0.044573	0.60334	1.3326 ↑	0.4142	0.03792	0.55845	1.598 ↑	0.6763
N-ACETYLORNITHINE_neg_3	0.060398	0.95219	0.84467 ↓	-0.244	0.41447	0.83033	0.91471 ↓	-0.129	0.52916	0.94319	0.94664 ↓	-0.079	0.048825	0.55845	0.78731 ↓	-0.345
OXOGLUTARATE_neg_2	0.12359	0.96989	1.1526 ↑	0.2049	0.25672	0.77754	1.208 ↑	0.2726	0.87528	0.99234	1.0409 ↑	0.0578	0.043903	0.55845	1.3645 ↑	0.4484
CE182	0.13573	0.96989	0.8103 ↓	-0.303	0.11927	0.72412	0.76512 ↓	-0.386	0.55704	0.94319	0.88953 ↓	-0.169	0.006022	0.42218	0.65944 ↓	-0.601
NORMETANEPHRINE_pos_1	0.1857	0.96989	0.87244 ↓	-0.197	0.16723	0.72412	0.78674 ↓	-0.346	0.44919	0.9283	0.89169 ↓	-0.165	0.014653	0.52739	0.67664 ↓	-0.564
ASCORBATE_neg_2	0.20445	0.96989	0.84866 ↓	-0.237	0.92254	0.9716	1.0032 ↑	0.0046	0.42125	0.92598	1.1697 ↑	0.2261	0.041969	0.55845	0.75285 ↓	-0.41
ACETYL-COA_pos_1	0.2095	0.96989	1.782 ↑	0.8335	0.001595	0.14065	2.6904 ↑	1.4278	0.080836	0.67363	1.7843 ↑	0.8353	0.001346	0.21242	2.8012 ↑	1.486
N-GLYCYL-L-PROLINE_neg_3	0.24586	0.96989	0.31441 ↓	-1.669	0.47972	0.8616	0.27759 ↓	-1.849	0.43559	0.92598	0.18999 ↓	-2.396	0.004664	0.42218	0.12578 ↓	-2.991
AMINOADIPATE_pos_1	0.24666	0.96989	0.89071 ↓	-0.167	0.41369	0.83033	0.91154 ↓	-0.134	0.83263	0.99234	1.04 ↑	0.0566	0.029949	0.55845	0.77834 ↓	-0.362
L-CARNITINE_pos_1	0.28883	0.96989	1.0836 ↑	0.1159	0.47332	0.85601	0.75408 ↓	-0.407	0.15478	0.77388	1.5723 ↑	0.6528	0.040165	0.55845	0.44975 ↓	-1.153
CE226	0.29835	0.96989	0.88425 ↓	-0.177	0.000218	0.09259	0.21171 ↓	-2.24	0.008059	0.42014	0.28761 ↓	-1.798	0.000577	0.21242	0.48516 ↓	-1.044
TAG566-FA225	0.30452	0.96989	1.2772 ↑	0.353	0.001203	0.14065	1.274 ↑	0.3493	0.018851	0.42014	1.1584 ↑	0.2122	0.009319	0.42218	1.4434 ↑	0.5295
GERANYL-PP_-HPO3_neg_2	0.32477	0.96989	0.92396 ↓	-0.114	0.19613	0.72412	0.83987 ↓	-0.252	0.50159	0.94319	0.95256 ↓	-0.07	0.035634	0.55845	0.73685 ↓	-0.441
TAG544-FA224	0.36504	0.96989	0.78541 ↓	-0.348	0.19476	0.72412	0.87888 ↓	-0.186	0.35404	0.92598	0.79328 ↓	-0.334	0.036992	0.55845	0.77637 ↓	-0.365
ACETYLPHOSPHATE_pos_1	0.4256	0.96989	0.91278 ↓	-0.132	0.28062	0.78188	0.84327 ↓	-0.246	0.55967	0.94389	1.0701 ↑	0.0978	0.016132	0.52739	0.68804 ↓	-0.539
N-ACETYLPUTRESCINE_pos_1	0.46953	0.96989	0.87575 ↓	-0.191	0.38661	0.82612	2.4572 ↑	1.297	0.76978	0.99234	0.88029 ↓	-0.184	0.041408	0.55845	4.6865 ↑	2.2285
TAG522-FA202	0.56368	0.96989	1.4634 ↑	0.5494	0.014195	0.43092	2.5221 ↑	1.3346	0.13452	0.74113	1.8637 ↑	0.8982	0.029921	0.55845	3.0959 ↑	1.6303
MESACONIC ACID_pos_1	0.67617	0.97467	0.94295 ↓	-0.085	0.41895	0.83035	2.5946 ↑	1.3755	0.66652	0.99046	0.87071 ↓	-0.2	0.036055	0.55845	5.2573 ↑	2.3943
TAG565-FA181	0.77568	0.98701	0.95426 ↓	-0.068	0.50825	0.87452	2.611 ↑	1.3846	0.5512	0.94319	0.85212 ↓	-0.231	0.03639	0.55845	5.4807 ↑	2.4544
SPERMIDINE_pos_4	0.80345	0.98701	0.94868 ↓	-0.076	0.46804	0.85576	2.5022 ↑	1.3232	0.6343	0.98776	0.8571 ↓	-0.222	0.037697	0.55845	5.1752 ↑	2.3716
TAG483-FA181	0.82339	0.98701	0.53495 ↓	-0.903	0.10879	0.72412	1.8258 ↑	0.8686	0.57998	0.9517	2.0303 ↑	1.0217	0.009438	0.42218	3.0733 ↑	1.6198
TAG520-FA200	0.84194	0.98847	1.2994 ↑	0.3779	0.10283	0.72412	1.5178 ↑	0.602	0.97062	0.99401	0.81463 ↓	-0.296	0.02342	0.55845	2.2635 ↑	1.1786
TAG501-FA140	0.86499	0.99469	0.46526 ↓	-1.104	0.56256	0.8992	2.1559 ↑	1.1083	0.74388	0.99234	1.0475 ↑	0.067	0.038272	0.55845	3.8055 ↑	1.9281
ATROLACTIC ACID_neg_2	0.88295	0.99469	1.0218 ↑	0.0311	0.001655	0.14065	2.0271 ↑	1.0194	0.12827	0.74022	1.9495 ↑	0.9631	0.006677	0.42218	4.5883 ↑	2.198
TAG582-FA181	0.92289	0.99469	1.2488 ↑	0.3205	0.025904	0.53437	0.62916 ↓	-0.669	0.73765	0.99234	0.97716 ↓	-0.033	0.009934	0.42218	0.60856 ↓	-0.717
TAG527-FA160	0.95573	0.99469	0.9806 ↓	-0.028	0.33329	0.7915	1.1222 ↑	0.1663	0.56627	0.94632	0.96911 ↓	-0.045	0.035824	0.55845	1.2806 ↑	0.3568
TAG545-FA205	0.98526	0.99469	0.8428 ↓	-0.247	0.096232	0.72412	0.78908 ↓	-0.342	0.35303	0.92598	0.75501 ↓	-0.405	0.039263	0.55845	0.68059 ↓	-0.555

Supplementary Table S2B. List of dysregulated metabolites for female, male, Wild type and APCHi mice following exposure to 9.5 Gy of Gamma-radiation, 1 week post-irradiation showing penotype dependent response of radiation.

	9.5Gy / 0.0Gy				9.5Gy / 0.0Gy				9.5Gy / 0.0Gy				9.5Gy / 0.0Gy			
	Female				Male				WildType				APCHi			
	1wk															
name	p-value	FDR	FC	LOG2FC	p-value	FDR	FC	LOG2FC	p-value	FDR	FC	LOG2FC	p-value	FDR	FC	LOG2FC
CE182	0.19654	0.95194	1.5876	0.66684	2.22E-05	0.005935	1.8333	0.87441	0.002567	0.92112	1.6029	0.68071	0.012612	0.12705	1.7614	0.81673
NORMETANEPHRINE_pos_1	0.12634	0.95194	1.997	0.99784	0.005353	0.27165	1.7693	0.82315	0.010437	0.92112	1.9158	0.93791	0.02484	0.17893	1.8933	0.92088
GERANYL-PP_-HPO3_neg_2	0.16098	0.95194	2.2096	1.1438	0.038224	0.52469	1.5625	0.64389	0.16872	0.92112	1.5095	0.59408	0.016303	0.14742	2.1295	1.0905
N-ACETYLPUTRESCINE_pos_1	0.62544	0.97562	1.199	0.26179	0.92509	0.99	0.43651	-1.1959	0.45355	0.93084	0.35468	-1.4954	0.003508	0.053246	1.5209	0.60496
TAG545-FA205	0.26551	0.95194	0.80639	-0.31046	0.48448	0.89138	0.84306	-0.2463	0.93486	0.98807	0.98919	-0.01568	0.039781	0.21415	0.60095	-0.73468
TAG552-FA181	0.15672	0.95194	1.3565	0.4399	0.013503	0.37637	1.3897	0.47473	0.51066	0.96398	1.1516	0.20361	8.5E-05	0.01386	1.8644	0.89872
TAG463-FA141	0.94525	0.98297	1.0198	0.028313	0.06138	0.63625	1.1956	0.25778	0.49013	0.95869	0.91846	-0.12271	0.000234	0.019885	1.5125	0.59692
TAG490-FA170	0.4378	0.97562	1.3302	0.41164	0.37964	0.89138	1.1109	0.15172	0.41911	0.92238	0.78797	-0.34378	0.00116	0.043099	2.0637	1.0453
ARGININOSUCCINIC ACID_neg_2	0.31223	0.95194	1.3902	0.47527	0.65892	0.93511	1.1219	0.16597	0.38522	0.92112	0.74409	-0.42645	0.006836	0.085445	2.3279	1.219
FFA141	0.025203	0.95194	1.8226	0.86599	0.22707	0.89138	1.1687	0.22492	0.44024	0.93084	1.1161	0.15844	0.002169	0.046168	1.69	0.75704
TAG514-FA161	0.12231	0.95194	1.3641	0.44798	0.032234	0.50675	1.2872	0.36426	0.69001	0.96398	1.0504	0.070896	6.72E-05	0.01386	1.8487	0.88651
FFA226	0.1411	0.95194	1.2453	0.31644	0.89919	0.99	1.0091	0.013126	0.82641	0.9749	0.95752	-0.06263	0.010081	0.11275	1.2724	0.34755
FFA181	0.1407	0.95194	2.0616	1.0438	0.45316	0.89138	1.4112	0.4969	0.5564	0.96398	1.1905	0.25158	0.009306	0.10952	2.1891	1.1303
TAG461-FA140	0.89376	0.98297	0.93884	-0.09104	0.11983	0.76624	0.75255	-0.41015	0.88656	0.98395	1.0577	0.080923	0.019451	0.15598	0.58896	-0.76375
TAG567-FA204	0.70298	0.98297	1.1656	0.22109	0.74006	0.95402	1.0677	0.094497	0.38541	0.92112	0.83649	-0.25759	0.015138	0.13987	1.5591	0.64071
TAG501-FA181	0.68307	0.97562	1.007	0.010026	0.31185	0.89138	0.71358	-0.48685	0.73441	0.96398	0.85568	-0.22486	0.048475	0.23411	0.60267	-0.73055
FFA205	0.26217	0.95194	1.2094	0.27424	0.66228	0.93511	1.0283	0.040261	0.86094	0.98395	0.96704	-0.04836	0.028798	0.1974	1.266	0.34031
TAG440-FA140	0.087808	0.95194	0.59365	-0.75232	0.071834	0.69389	0.81494	-0.29524	0.32235	0.92112	0.82381	-0.27962	0.011139	0.12139	0.63699	-0.65065
SM261	0.21453	0.95194	1.2449	0.31601	0.94399	0.99	0.99083	-0.01329	0.90114	0.98395	0.93605	-0.09534	0.028415	0.1974	1.2763	0.35192
TAG511-FA150	0.15725	0.95194	0.75597	-0.40361	0.097474	0.76624	0.75882	-0.39818	0.49089	0.95869	1.1615	0.21604	0.000894	0.042231	0.48903	-1.032
TAG525-FA225	0.2332	0.95194	1.279	0.35502	0.10256	0.76624	1.3061	0.38525	0.26885	0.92112	1.2184	0.285	0.021742	0.16217	1.594	0.67261
TAG544-FA160	0.83779	0.98297	0.82655	-0.27483	0.10889	0.76624	0.70325	-0.50789	0.8524	0.98176	1.0169	0.024244	0.026412	0.18709	0.51447	-0.95883
TAG512-FA181	0.051674	0.95194	1.5059	0.59065	0.26548	0.89138	1.1192	0.16252	0.66082	0.96398	1.0259	0.03686	0.003001	0.049048	1.5786	0.65869
TAG542-FA202	0.84996	0.98297	0.95955	-0.05957	0.44436	0.89138	0.81604	-0.29329	0.36504	0.92112	1.1405	0.18961	0.041026	0.21415	0.63564	-0.65372
CE161	0.21781	0.95194	0.60396	-0.72748	0.079505	0.71893	0.78051	-0.35751	0.72329	0.96398	0.88388	-0.17807	0.004979	0.067646	0.56886	-0.81386
TAG564-FA181	0.9732	0.98479	0.99605	-0.00571	0.033386	0.50675	1.3354	0.41732	0.92647	0.98807	1.0631	0.088231	0.007266	0.088233	1.6003	0.67831
TAG531-FA181	0.21673	0.95194	0.55671	-0.84499	0.04322	0.55662	0.80671	-0.30988	0.58561	0.96398	0.80096	-0.3202	0.001217	0.043099	0.55469	-0.85024
TAG461-FA161	0.20897	0.95194	1.2986	0.37693	0.41979	0.89138	1.0472	0.066481	0.70164	0.96398	0.86884	-0.20283	0.001017	0.043099	1.6126	0.68942
TAG490-FA180	0.085391	0.95194	1.3573	0.44069	0.33249	0.89138	1.3416	0.42394	0.55754	0.96398	1.171	0.22773	0.036262	0.21405	1.6975	0.76342
TAG491-FA181	0.19881	0.95194	1.3391	0.42128	0.15352	0.85739	1.1263	0.17156	0.99161	0.99785	0.90226	-0.14838	0.000615	0.041618	1.7743	0.82725
MALONYL-COA_pos_1	0.67405	0.97562	1.1698	0.2262	0.5881	0.89995	1.0925	0.12765	0.49709	0.96398	0.8125	-0.29956	0.01702	0.14762	1.7394	0.79861
TAG512-FA150	0.63928	0.97562	1.1011	0.13896	0.058366	0.63604	1.1887	0.24943	0.94686	0.98807	1.027	0.038379	0.011847	0.12588	1.388	0.47305
TAG562-FA160	0.45778	0.97562	1.3567	0.4401	0.72393	0.9445	1.0737	0.10256	0.41022	0.92238	0.78628	-0.34689	0.012854	0.12705	1.9691	0.97757
TAG470-FA170	0.91195	0.98297	1.0502	0.070687	0.25563	0.89138	1.1451	0.19544	0.75325	0.96398	0.86492	-0.20936	0.034912	0.20995	1.6522	0.72439
TAG511-FA181	0.71411	0.98297	1.0607	0.084957	0.008339	0.27531	1.4857	0.57117	0.98419	0.99666	1.1276	0.17323	0.002376	0.046168	1.9054	0.93012
TAG546-FA183	0.2027	0.95194	1.285	0.36173	0.25248	0.89138	1.1028	0.14114	0.72117	0.96398	0.88522	-0.17589	9.78E-05	0.01386	1.689	0.75617
TAG501-FA161	0.98255	0.9902	1.0921	0.12704	0.82209	0.97366	0.99006	-0.01441	0.14774	0.92112	0.71556	-0.48286	0.048207	0.23411	1.6752	0.74436
FFA161	0.34596	0.95726	1.205	0.269	0.14291	0.80983	1.1232	0.16763	0.5516	0.96398	1.0611	0.085556	0.04242	0.21462	1.2973	0.37554
TAG545-FA225	0.094699	0.95194	2.3398	1.2264	0.048962	0.59454	1.4705	0.55634	0.23351	0.92112	1.5242	0.60808	0.034991	0.20995	2.1497	1.1041
FFA204	0.68231	0.97562	0.75982	-0.39628	0.7741	0.97247	1.0417	0.058889	0.074498	0.92112	0.61106	-0.71061	0.047034	0.23244	1.5248	0.60863
BUTYRYL-COA_pos_1	0.94342	0.98297	0.67544	-0.5661	0.020173	0.37637	1.7709	0.82447	0.47943	0.95214	1.0477	0.067207	0.013829	0.13061	2.1907	1.1314
TAG492-FA160	0.080576	0.95194	1.436	0.52205	0.34592	0.89138	1.0779	0.10817	0.76489	0.97038	0.99538	-0.00669	0.013194	0.12744	1.5004	0.58539
TAG400-FA160	0.090349	0.95194	0.56763	-0.81697	0.75538	0.95832	1.0868	0.12007	0.9477	0.98807	0.96797	-0.04696	0.040526	0.21415	0.66118	-0.59689
TAG502-FA161	0.23918	0.95194	0.7833	-0.35235	0.18129	0.89138	0.88833	-0.17083	0.79384	0.9749	0.92171	-0.11761	0.002077	0.046168	0.7204	-0.47313
TAG491-FA160	0.27562	0.95194	1.2376	0.30758	0.40795	0.89138	1.0511	0.07188	0.65015	0.96398	0.84543	-0.24224	0.002755	0.04683	1.6498	0.72229
TAG555-FA182	0.66959	0.97562	0.68043	-0.55548	0.62128	0.91862	1.0515	0.072463	0.090554	0.92112	0.57055	-0.80958	0.037987	0.21415	1.5243	0.60815
TAG500-FA140	0.20078	0.95194	1.2491	0.32084	0.46118	0.89138	1.0787	0.10934	0.57153	0.96398	0.85867	-0.21983	0.018974	0.15598	1.5825	0.66223
TAG545-FA180	0.050638	0.95194	0.61633	-0.69823	0.066693	0.67487	0.81032	-0.30344	0.33187	0.92112	0.85391	-0.22784	0.00146	0.04527	0.59675	-0.7448
TAG547-FA182	0.089664	0.95194	0.68383	-0.54829	0.2052	0.89138	0.84474	-0.24342	0.42321	0.92238	0.89389	-0.16183	0.021749	0.16217	0.65148	-0.61822
TAG471-FA150	0.4846	0.97562	1.1012	0.13912	0.019662	0.37637	1.2292	0.29772	0.16464	0.92112	1.1539	0.20647	0.032616	0.20385	1.323	0.40382
N-ACETYLALANINE_neg_1	0.29686	0.95194	0.71653	-0.4809	0.30349	0.89138	0.78158	-0.35554	0.97763	0.99639	1.0034	0.004873	0.039764	0.21415	0.55516	-0.84903
TAG534-FA170	0.55525	0.97562	0.91815	-0.1232	0.30603	0.89138	1.0825	0.11437	0.20303	0.92112	0.89893	-0.15372	0.03071	0.20385	1.2198	0.28664
ALLANTOIN_neg_2	0.50863	0.97562	1.1826	0.24192	0.042551	0.55662	1.3134	0.39328	0.23712	0.92112	1.2453	0.31651	0.040411	0.21415	1.4438	0.52987
TAG522-FA181	0.69125	0.97602	1.071	0.099008	0.67878	0.93856	1.0241	0.034396	0.25968	0.92112	0.8383	-0.25446	0.035074	0.20995	1.4039	0.48947
TAG441-FA141	0.4643	0.97562	0.59053	-0.75992	0.39208	0.89138	1.0891	0.12319	0.057131	0.92112	0.53733	-0.89612	0.032139	0.20385	1.483	0.56855
TAG589-FA181	0.039359	0.95194	0.6076	-0.71881	0.85386	0.98587	0.9095	-0.13685	0.82165	0.9749	0.97048	-0.04323	0.005253	0.067646	0.48575	-1.0417
CITRACONIC ACID_neg	0.21903	0.95194	1.2192	0.28595	0.54817	0.89728	1.0421	0.05953	0.55643	0.96398	0.90896	-0.13771	0.002732	0.04683	1.43	0.51601
TAG546-FA181	0.44845	0.97562	1.219	0.28575	0.01894	0.37637	1.4132	0.499	0.42939	0.92414	1.2167	0.283	0.00239	0.046168	1.7305	0.79117
TAG563-FA181	0.14073	0.95194	0.70107	-0.51237	0.24058	0.89138	0.83259	-0.26432	0.62563	0.96398	1.0822	0.11395	0.002536	0.04683	0.5308	-0.91377
TAG543-FA202	0.86624	0.98297	1.0306	0.043552	0.1021	0.76624	1.1442	0.1944	0.86823	0.98395	0.99448	-0.00799	0.032078	0.20385	1.2897	0.36707
TAG510-FA170	0.32346	0.95194	1.3068	0.38602	0.01721	0.37637	1.4243	0.51028	0.39955	0.92112	1.2341	0.30351	0.001806	0.046168	1.8086	0.85491
TAG503-FA161	0.28723	0.95194	0.78814	-0.34347	0.3243	0.89138	0.8446	-0.24366	0.95899	0.99206	0.99942	-0.00083	0.045241	0.2262	0.66203	-0.59504
TAG543-FA181	0.13103	0.95194	0.57286	-0.80373	0.49741	0.89138	0.85788	-0.22115	0.79467	0.9749	0.89537	-0.15945	0.002292	0.046168	0.47805	-1.0648
DAG161/182	0.057158	0.95194	0.61122	-0.71025	0.25482	0.89138	0.82913	-0.27034	0.8394	0.97588	0.91657	-0.12568	0.000783	0.041618	0.53133	-0.91232
GLUCOSE 1-PHOSPHATE_pos_2	0.48934	0.97562	0.56525	-0.82303	0.000268	0.030918	8.2873	3.0509	0.12605	0.92112	4.9164	2.2976	0.00519	0.067646	6.3293	2.6621
2-HYDROXY-3-METHYLBUTYRIC ACID_po	0.21481	0.95194	1.3506	0.43359	0.9261	0.99	0.97904	-0.03057	0.72667	0.96398	0.90797	-0.13929	0.020459	0.16102	1.3724	0.45668
TAG533-FA170	0.32225	0.95194	0.33505	-1.5776	0.17506	0.89138	0.72938	-0.45526	0.39372	0.92112	0.37599	-1.4112	0.019412	0.15598	0.35863	-1.4794
TAG524-FA183	0.31487	0.95194	0.72867	-0.45666	0.24777	0.89138	0.78545	-0.3484	0.80291	0.9749	1.0431	0.060841	0.024083	0.17647	0.53747	-0.89573
PROPIONYL-COA_pos_1	0.3498	0.95726	1.3034	0.38226	0.021015	0.37637	1.3938	0.47907	0.46142	0.93084	1.2077	0.27227	0.001953	0.046168	1.7715	0.82499
TAG522-FA182	0.36269	0.95901	1.3006	0.3792	0.016614	0.37637	1.4327	0.51876	0.36729	0.92112	1.2511	0.32315	0.003299	0.051932	1.796	0.8448
TAG503-FA141	0.31767	0.95194	0.80249	-0.31745	0.24141	0.89138	0.78659	-0.34632	0.31067	0.92112	1.2069	0.27135	0.004826	0.067646	0.52549	-0.92826
TAG504-FA161	0.080072	0.95194	0.59919	-0.73892	0.21441	0.89138	0.85367	-0.22825	0.36053	0.92112	0.81557	-0.29412	0.016886	0.14762	0.59938	-0.73847
DAG181/204	0.23773	0.95194	1.2764	0.35211	0.075882	0.70108	1.1821	0.2413	0.21907	0.92112	1.1411	0.1904	0.037066	0.21415	1.3438	0.42635
LACTATE_neg_1	0.18385	0.95194	0.6923	-0.53053	0.32723	0.89138	0.81374	-0.29736	0.69144	0.96398	1.0246	0.035038	0.004198	0.061525	0.509	-0.97427
TAG587-FA180	0.32767	0.95194	1.2081	0.27276	0.096849	0.76624	1.1543	0.20696	0.36717	0.92112	1.0967	0.13312	0.041919	0.21462	1.3081	0.38752
TAG524-FA182	0.23369	0.95194	1.3161	0.39624	2.79E-05	0.005935	3.1023	1.6333	0.11589	0.92112	3.0087	1.5892	0.009535	0.10952	4.7942	2.2613
TAG543-FA182	0.18749	0.95194	0.72734	-0.4593	0.36485	0.89138	0.84515	-0.24271	0.99785	0.99785	1.0241	0.034326	0.031708	0.20385	0.61821	-0.69383
TAG492-FA140	0.93499	0.98297	0.88933	-0.16921	0.41084	0.89138	0.74542	-0.42387	0.28214	0.92112	1.2476	0.3192	0.021525	0.16217	0.45474	-1.1369
FFA241	0.24343	0.95194	0.74058	-0.43327	0.65209	0.93511	0.81778	-0.29022	0.70811	0.96398	1.0422	0.059647	0.040409	0.21415	0.54566	-0.87394
O-ACETYLSERINE_pos_3	0.045167	0.95194	1.9754	0.98216	0.038271	0.52469	1.6774	0.74622	0.32719	0.92112	1.3269	0.40809	0.001491	0.04527	2.4282	1.2799
TAG544-FA183	0.26156	0.95194	0.80853	-0.30663	0.29211	0.89138	0.69295	-0.52919	0.33311	0.92112	1.1806	0.23951	0.000206	0.019885	0.43056	-1.2157
TAG524-FA181	0.92017	0.98297	0.99782	-0.00315	0.32208	0.89138	1.0792	0.10999	0.05834	0.92112	0.88815	-0.17112	0.030513	0.20385	1.3045	0.38352
MYOINOSITOL_neg_3	0.18842	0.95194	1.4673	0.55318	0.5835	0.89995	1.1023	0.14046	0.54481	0.96398	0.90863	-0.13823	0.001934	0.046168	1.7227	0.78464
TAG544-FA181	0.092607	0.95194	1.5939	0.67258	0.33378	0.89138	1.1023	0.14056	0.28447	0.92112	1.1956	0.25768	0.038703	0.21415	1.3544	0.43765
TAG530-FA160	0.27212	0.95194	1.301	0.37965	0.13527	0.80983	1.1707	0.22741	0.45067	0.93084	1.1135	0.15506	0.01925	0.15598	1.3899	0.47495
TAG525-FA182	0.3431	0.95726	1.2399	0.31021	0.27862	0.89138	1.0917	0.12652	0.81746	0.9749	0.95719	-0.06312	0.012615	0.12705	1.4338	0.51986
TAG565-FA225	0.49125	0.97562	0.98561	-0.02091	0.003548	0.25129	2.4871	1.3145	0.10058	0.92112	1.5287	0.61231	0.041319	0.21415	1.7705	0.8242
TAG503-FA181	0.089355	0.95194	1.2959	0.37391	0.51391	0.89138	1.1155	0.15767	0.27564	0.92112	0.90298	-0.14723	0.000695	0.041618	1.6898	0.75689

Supplementary Table S2C. List of dysregulated metabolites for female, male, Wild type and APCHi mice following exposure to 9.5 Gy of Gamma-radiation, 1 month post-irradiation showing penotype dependent response of radiation.

	9.5Gy / 0.0Gy				9.5Gy / 0.0Gy				9.5Gy / 0.0Gy				9.5Gy / 0.0Gy			
	Female				Male				WildType				APCHi			
	1mo															
name	p-value	FDR	FC	LOG2FC	p-value	FDR	FC	LOG2FC	p-value	FDR	FC	LOG2FC	p-value	FDR	FC	LOG2FC
ASCORBATE_neg_2	0.10102	0.89093	0.80214	-0.31807	0.017433	0.324	0.42884	-1.2215	0.072301	0.22594	0.43997	-1.1845	0.00514	0.16803	0.47959	-1.0601
TAG566-FA225	0.43322	0.99953	0.71244	-0.48915	0.22305	0.64952	0.6245	-0.67923	0.90521	0.94062	0.65659	-0.60694	0.039781	0.26836	0.62067	-0.6881
TAG544-FA224	0.013325	0.62598	0.38637	-1.372	0.21399	0.64834	1.1608	0.2151	0.028438	0.12722	1.7612	0.81652	0.043448	0.27978	0.57725	-0.79274
TAG501-FA140	0.31908	0.99215	1.2734	0.34868	0.50791	0.81765	1.1789	0.23747	0.56211	0.76038	0.9058	-0.14274	0.032673	0.25181	1.5252	0.60904
ATROLACTIC ACID_neg_2	0.65053	0.99953	1.188	0.24851	0.005527	0.24745	2.1122	1.0788	0.17951	0.38532	2.0219	1.0157	0.020336	0.22709	1.6913	0.75809
TAG483-FA182	0.5075	0.99953	1.121	0.1648	0.43582	0.78215	0.77479	-0.36812	0.003112	0.045599	0.447	-1.1616	0.004866	0.16803	1.4046	0.49012
TAG521-FA201	0.002293	0.46622	0.54014	-0.88858	0.3504	0.73321	0.90501	-0.144	0.52748	0.73853	0.95037	-0.07343	0.023405	0.22824	0.64953	-0.62254
TAG521-FA161	0.008581	0.61582	0.58627	-0.77035	0.82358	0.94791	0.94416	-0.08289	0.55515	0.75896	1.0883	0.12206	0.021283	0.22709	0.64927	-0.62311
TAG442-FA182	0.77211	0.99953	1.1856	0.24567	0.051775	0.43146	1.5234	0.60733	0.84973	0.90059	1.3354	0.41727	0.015674	0.22433	1.6437	0.71693
ADP_pos_1	0.070854	0.87596	2.6794	1.4219	0.000562	0.1195	1.9971	0.99788	0.031983	0.1373	1.7857	0.8365	0.002066	0.16803	2.6607	1.4118
TAG564-FA204	0.2361	0.985	0.65622	-0.60774	0.23881	0.64952	1.1224	0.16665	0.001354	0.034775	3.1709	1.6649	0.036086	0.25181	0.46537	-1.1035
IMIDAZOLE_pos_1	0.14906	0.91055	1.6868	0.75432	0.058087	0.4396	1.8301	0.87188	0.23953	0.47794	1.9621	0.97243	0.038326	0.26272	1.6514	0.72365
TAG442-FA181	0.63393	0.99953	1.5932	0.67192	0.033603	0.39891	1.8674	0.901	0.37291	0.59582	2.2594	1.176	0.016784	0.22709	1.8858	0.91516
MEVALONOLACTONE_pos_2	0.30637	0.99215	1.7333	0.79351	0.065133	0.4396	1.2557	0.32846	0.29072	0.53798	1.147	0.19792	0.044569	0.28157	1.6942	0.7606
ARGININOSUCCINIC ACID_neg_2	0.26838	0.99215	0.77934	-0.35967	0.12339	0.52084	0.6823	-0.55152	0.60797	0.77209	0.91698	-0.12504	0.021275	0.22709	0.48504	-1.0438
TAG482-FA140	0.18478	0.93892	1.1706	0.22727	0.59814	0.86173	0.74683	-0.42114	0.004937	0.053803	0.45983	-1.1208	0.013453	0.21636	1.3345	0.41625
TAG482-FA141	0.26537	0.99215	1.1889	0.24961	0.54318	0.83754	0.78394	-0.35118	0.000847	0.034775	0.48689	-1.0383	0.013341	0.21636	1.3724	0.45672
TAG562-FA180	0.6052	0.99953	0.77675	-0.36447	0.54058	0.83754	1.0921	0.12705	0.002092	0.035955	2.2509	1.1705	0.042297	0.27926	0.62181	-0.68546
TAG461-FA181	0.43465	0.99953	1.3549	0.43813	0.46831	0.80341	1.212	0.27735	0.36392	0.58809	0.91781	-0.12374	0.008292	0.21636	1.6365	0.71059
TAG441-FA160	0.75018	0.99953	1.0934	0.12888	0.1164	0.52084	1.2605	0.33403	0.79164	0.86714	1.0595	0.083383	0.019462	0.22709	1.4253	0.51122
TAG462-FA181	0.55263	0.99953	1.2705	0.34535	0.39175	0.77005	1.2649	0.33901	0.52827	0.73853	1.0454	0.064023	0.024603	0.22824	1.5518	0.63396
TAG461-FA140	0.3954	0.99953	1.23	0.29865	0.92735	0.96836	0.88873	-0.17018	0.009001	0.065952	0.56313	-0.82845	0.007165	0.20301	1.4581	0.54406
TAG441-FA140	0.7056	0.99953	1.1081	0.14806	0.90667	0.96389	0.93344	-0.09937	0.052903	0.18271	0.5987	-0.7401	0.047972	0.29114	1.4095	0.49517
TAG461-FA120	0.67734	0.99953	1.1695	0.22593	0.26607	0.65908	1.1774	0.23557	0.41181	0.61838	0.87164	-0.19819	0.015734	0.22433	1.5204	0.60445
4-IMIDAZOLEACETATE_pos_1	0.19472	0.93892	0.83161	-0.26603	0.018296	0.324	0.75054	-0.41399	0.34777	0.58192	0.95413	-0.06774	0.000904	0.16803	0.49723	-1.008
URIDINE 5-DIPHOSPHATE_neg_2	0.10802	0.89093	0.76587	-0.38482	0.1811	0.61953	0.68804	-0.53945	0.57834	0.76365	0.90307	-0.14709	0.029798	0.24396	0.52661	-0.92519
TAG461-FA160	0.51914	0.99953	1.1658	0.22133	0.87557	0.95387	0.92565	-0.11146	0.008948	0.065952	0.59278	-0.75444	0.005126	0.16803	1.4423	0.52838
4-AMINOBENZOIC ACID_pos_1	0.82134	0.99953	0.82747	-0.27322	0.018202	0.324	0.60309	-0.72956	0.062816	0.2029	0.68457	-0.54672	0.029062	0.24396	0.57532	-0.79756
TAG482-FA181	0.38862	0.99953	1.1812	0.24026	0.7861	0.93401	0.86056	-0.21665	0.014609	0.083905	0.52439	-0.93127	0.003703	0.16803	1.4681	0.55393
DAG160/182	0.11739	0.89093	0.85072	-0.23325	0.6817	0.90614	0.97696	-0.03364	0.31416	0.55402	1.1982	0.26085	0.027738	0.24396	0.75479	-0.40586
TAG589-FA226	0.92116	0.99953	0.90625	-0.14202	0.1042	0.52084	0.57014	-0.81061	0.55493	0.75896	1.2117	0.27702	0.021245	0.22709	0.48768	-1.036
TAG564-FA181	0.31246	0.99215	0.73698	-0.4403	0.11432	0.52084	0.65238	-0.61623	0.63841	0.79903	1.0077	0.011001	0.036142	0.25181	0.48473	-1.0447
TAG481-FA141	0.18956	0.93892	1.369	0.45314	0.60427	0.86553	0.89095	-0.16658	0.10062	0.27066	0.66906	-0.57979	0.015835	0.22433	1.382	0.46673
TAG441-FA181	0.53218	0.99953	1.4642	0.55014	0.12262	0.52084	1.6096	0.68673	0.60714	0.77209	1.706	0.77061	0.022441	0.22709	1.7543	0.81086
TAG472-FA140	0.72793	0.99953	1.0979	0.13478	0.9437	0.97585	0.80116	-0.31984	0.023653	0.11047	0.45642	-1.1316	0.03604	0.25181	1.4159	0.50168
TAG482-FA160	0.19548	0.93892	1.1646	0.21985	0.36036	0.73668	0.75628	-0.403	0.002115	0.035955	0.4807	-1.0568	0.013378	0.21636	1.2979	0.37618
TAG491-FA181	0.29069	0.99215	1.1251	0.17002	0.49732	0.8154	0.8104	-0.3033	0.001051	0.034775	0.54482	-0.87614	0.045051	0.28157	1.2412	0.31172
TAG553-FA181	0.020484	0.66968	0.67082	-0.576	0.56062	0.84263	0.89498	-0.16007	0.94752	0.96324	0.97888	-0.0308	0.024704	0.22824	0.73457	-0.44503
TAG421-FA181	0.82449	0.99953	1.3086	0.38807	0.014703	0.31736	1.951	0.96425	0.12772	0.31929	2.6259	1.3928	0.026828	0.24259	1.7626	0.8177
TAG421-FA140	0.24058	0.99215	1.2902	0.3676	0.63726	0.89385	1.1077	0.14752	0.94964	0.96324	0.97972	-0.02955	0.046518	0.28652	1.3655	0.44939
TAG547-FA183	0.27741	0.99215	0.75049	-0.41409	0.84732	0.95387	0.68328	-0.54945	0.11532	0.30253	1.8631	0.89772	0.035988	0.25181	0.53533	-0.90151
TAG525-FA181	0.031602	0.7334	0.72885	-0.45631	0.87845	0.95387	0.94833	-0.07654	0.27187	0.52047	1.0778	0.10813	0.029304	0.24396	0.78707	-0.34543
TAG587-FA182	0.98999	0.99953	0.8169	-0.29176	0.83905	0.95092	0.93749	-0.09313	0.006324	0.062783	1.7736	0.8267	0.04271	0.27926	0.56344	-0.82767
TAG561-FA181	0.096902	0.89093	0.82006	-0.28621	0.38894	0.77005	1.0393	0.055651	0.007234	0.062783	1.3749	0.45938	0.018115	0.22709	0.81571	-0.29387
INDOLEACRYLIC ACID_pos_1	0.20204	0.93892	4.1684	2.0595	0.1534	0.58736	2.8101	1.4906	0.27954	0.53038	3.4807	1.7994	0.035929	0.25181	3.9306	1.9748
TAG481-FA180	0.53147	0.99953	1.1745	0.23211	0.062462	0.4396	1.2689	0.34358	0.86507	0.91004	1.0167	0.023896	0.011844	0.21636	1.4907	0.57597
TAG543-FA183	0.011421	0.62598	0.33658	-1.571	0.23825	0.64952	1.1289	0.17495	0.020686	0.10105	1.7908	0.84058	0.012534	0.21636	0.44998	-1.1521
METHIONINE_pos_1	0.22582	0.95973	0.70949	-0.49515	0.2576	0.65557	0.77848	-0.36127	0.90009	0.93759	0.93371	-0.09896	0.013728	0.21636	0.53885	-0.89205
TAG546-FA181	0.12779	0.91055	0.76907	-0.37882	0.42839	0.78215	0.6812	-0.55385	0.24662	0.4875	1.0524	0.073656	0.004011	0.16803	0.56923	-0.81291
TAG481-FA160	0.077846	0.89093	1.235	0.30449	0.76274	0.93401	0.83291	-0.26376	0.006095	0.062783	0.53072	-0.91397	0.004074	0.16803	1.3949	0.48019
TAG563-FA181	0.005613	0.59639	0.68527	-0.54525	0.089548	0.49525	1.1696	0.22606	0.00126	0.034775	1.7297	0.79053	0.031448	0.25181	0.82739	-0.27336
TAG462-FA120	0.099909	0.89093	1.4474	0.53349	0.98249	0.99242	0.88725	-0.17259	0.044524	0.16895	0.52444	-0.93116	0.003902	0.16803	1.6897	0.75674
2-KETOHEXANOIC ACID_pos_2	0.41737	0.99953	1.8679	0.90143	0.013817	0.31736	1.4449	0.53102	0.13556	0.32922	1.2803	0.3565	0.032661	0.25181	1.9187	0.94012
TAG542-FA182	0.50265	0.99953	0.89859	-0.15426	0.80984	0.93783	0.96852	-0.04614	0.013832	0.0835	1.5927	0.67148	0.033205	0.25181	0.68038	-0.55559
TAG525-FA160	0.6381	0.99953	0.86266	-0.21314	0.3322	0.72506	0.5864	-0.77004	0.4959	0.70962	0.71304	-0.48796	0.048637	0.29114	0.63654	-0.65167
DAG140/182	0.015706	0.62598	2.241	1.1642	0.20954	0.64834	1.2941	0.37198	0.76308	0.86023	1.1229	0.16723	0.006184	0.18773	1.7549	0.81143
PIPECOLATE_pos_3	0.3695	0.99953	0.94882	-0.07579	0.079813	0.48271	0.79951	-0.32281	0.78803	0.86714	1.1469	0.19768	0.002154	0.16803	0.49982	-1.0005
TAG421-FA161	0.57642	0.99953	1.168	0.22406	0.57428	0.85041	0.89894	-0.1537	0.15127	0.35011	0.5595	-0.83779	0.023821	0.22824	1.4597	0.54564
TAG482-FA182	0.14642	0.91055	1.2261	0.29411	0.47883	0.8154	0.91075	-0.13487	0.043422	0.16777	0.56226	-0.83069	0.01109	0.21636	1.4853	0.57073
TAG583-FA181	0.25752	0.99215	0.78158	-0.35554	0.068171	0.4527	0.73272	-0.44867	0.88805	0.92733	1.3167	0.39688	0.022129	0.22709	0.52357	-0.93354
TAG545-FA181	0.54658	0.99953	0.88382	-0.17817	0.3621	0.73668	0.65756	-0.6048	0.21773	0.44488	1.0162	0.023214	0.013586	0.21636	0.59858	-0.74038
CER220	0.3019	0.99215	0.92977	-0.10505	0.83617	0.95092	1.1085	0.14864	0.008879	0.065952	3.7927	1.9232	0.005035	0.16803	0.48655	-1.0394
LCER160	0.1072	0.89093	0.62274	-0.6833	4.55E-06	0.001935	2.959	1.5651	0.14477	0.34567	2.4937	1.3183	0.020693	0.22709	3.2833	1.7152
TAG546-FA226	0.024607	0.69719	0.6386	-0.64702	0.45689	0.79626	0.99385	-0.0089	0.017421	0.092552	1.7113	0.77506	0.002937	0.16803	0.61691	-0.69687
BETAINE_pos_2	0.20988	0.93892	0.57169	-0.80669	0.49883	0.8154	0.82669	-0.27458	0.78017	0.86714	1.036	0.051082	0.013087	0.21636	0.35035	-1.5131
TAG481-FA140	0.26577	0.99215	1.1879	0.24846	0.87676	0.95387	0.94404	-0.08309	0.087604	0.25328	0.60702	-0.72017	0.011703	0.21636	1.4657	0.55156
TAG481-FA181	0.20533	0.93892	1.323	0.40384	0.87157	0.95387	0.98314	-0.02452	0.11772	0.30321	0.64713	-0.62788	0.005124	0.16803	1.5622	0.64356
FFA241	0.44905	0.99953	0.68291	-0.55023	0.32947	0.72506	0.87194	-0.19771	0.012867	0.081618	1.9197	0.94091	0.029849	0.24396	0.51508	-0.95713
TAG544-FA180	0.74411	0.99953	1.0515	0.072442	0.22751	0.64952	0.67185	-0.57378	0.31231	0.55402	1.1871	0.24749	0.019984	0.22709	0.65596	-0.60832
ASPARTATE_pos_3	0.32954	0.99215	1.8868	0.91597	0.030484	0.38105	1.4773	0.56301	0.2819	0.53247	1.2953	0.37324	0.013745	0.21636	1.9828	0.98756
BIOTIN_pos_4	0.26021	0.99215	1.5111	0.59565	0.058679	0.4396	1.2789	0.35487	0.56791	0.76038	1.121	0.16475	0.022153	0.22709	1.5687	0.64959

Supplementary Table S2D. List of dysregulated metabolites for female, male, Wild type and APCHi mice following exposure to 9.5 Gy of Gamma-radiation, 3 months post-irradiation showing penotype dependent response of radiation.

	9.5Gy / 0.0Gy				9.5Gy / 0.0Gy				9.5Gy / 0.0Gy				9.5Gy / 0.0Gy			
	Female				Male				WildType				APCHi			
	3mo															
name	p-value	FDR	FC	LOG2FC	p-value	FDR	FC	LOG2FC	p-value	FDR	FC	LOG2FC	p-value	FDR	FC	LOG2FC
CYSTEINE_neg_5	0.14091	0.33558	1.3473	0.43009	0.000531	0.22564	6.3248	2.661	0.068388	0.80329	3.534	1.8213	0.018044	0.31888	2.0079	1.0057
CE226	0.006099	0.1372	2.7635	1.4665	0.024579	0.99441	1.7456	0.80376	0.20003	0.80329	1.4064	0.49198	0.004002	0.28348	2.9239	1.5479
TAG522-FA202	0.21117	0.40795	1.6024	0.6802	0.25046	0.99441	1.323	0.4038	0.89065	0.97744	1.0949	0.13082	0.026206	0.33448	1.8093	0.85544
TAG565-FA181	0.00351	0.13256	0.15593	-2.681	0.1959	0.99441	0.77464	-0.3684	0.033546	0.80329	0.22613	-2.1448	0.042853	0.33448	0.24502	-2.029
TAG582-FA181	0.006539	0.1372	0.19481	-2.3599	0.53628	0.99441	1.0416	0.058736	0.80528	0.95886	0.49193	-1.0235	0.012692	0.31888	0.31193	-1.6807
TAG568-FA205	0.15318	0.35191	0.80456	-0.31373	0.36274	0.99441	0.93073	-0.10356	0.96321	0.9936	1.2176	0.28402	0.017164	0.31888	0.61947	-0.6909
FFA201	0.2469	0.44582	1.215	0.28093	0.45424	0.99441	1.1731	0.23037	0.65266	0.90034	0.94045	-0.08857	0.01379	0.31888	1.4531	0.53912
FFA182	0.10256	0.28678	1.3037	0.38263	0.39516	0.99441	1.1495	0.20096	0.89143	0.97744	0.98237	-0.02566	0.005577	0.31888	1.4559	0.54187
FFA141	0.1825	0.38685	1.2649	0.339	0.25313	0.99441	1.2098	0.27471	0.74617	0.94946	0.90843	-0.13856	0.018246	0.31888	1.5035	0.5883
TAG482-FA140	0.071362	0.23511	0.71496	-0.48406	0.39798	0.99441	0.93302	-0.10002	0.50219	0.88561	0.91171	-0.13336	0.037483	0.33448	0.75235	-0.41053
FFA181	0.13068	0.32113	1.2777	0.35353	0.39229	0.99441	1.1426	0.19236	0.85525	0.96928	0.9684	-0.04633	0.017773	0.31888	1.4212	0.50709
FFA205	0.16276	0.36793	1.3744	0.45878	0.41245	0.99441	1.2073	0.27177	0.65117	0.90034	0.96734	-0.0479	0.012472	0.31888	1.559	0.64062
TAG525-FA161	0.012458	0.1372	0.12542	-2.9951	0.19941	0.99441	0.83313	-0.26339	0.12345	0.80329	0.20356	-2.2965	0.016136	0.31888	0.23971	-2.0606
4-AMINOBENZOIC ACID_pos_1	0.13333	0.32566	0.62746	-0.67241	0.62694	0.99441	0.68557	-0.54462	0.49257	0.8833	1.0674	0.094129	0.010325	0.31888	0.45874	-1.1242
DAG181/226	0.12035	0.30627	1.4824	0.56792	0.87147	0.99441	1.0055	0.007969	0.66358	0.90034	0.9674	-0.04781	0.023962	0.33448	1.3673	0.45138
TAG502-FA181	0.033249	0.15925	0.58347	-0.77728	0.39331	0.99441	0.92079	-0.11905	0.45153	0.87228	0.81041	-0.30327	0.014255	0.31888	0.65462	-0.61126
TAG511-FA181	0.26252	0.46488	0.90332	-0.14669	0.61086	0.99441	0.95895	-0.06047	0.88451	0.97744	1.03	0.042672	0.01522	0.31888	0.85174	-0.23151
TAG532-FA181	0.36854	0.59049	1.1639	0.21892	0.91983	0.99441	1.0119	0.017059	0.38832	0.87102	0.92335	-0.11505	0.04621	0.33448	1.2231	0.29054
HCER201	0.014677	0.1372	5.6808	2.5061	0.2352	0.99441	1.4581	0.54408	0.79978	0.95886	1.499	0.584	0.002748	0.23372	3.1023	1.6333
FFA204	0.46728	0.66904	1.1156	0.15781	0.62314	0.99441	1.1533	0.20577	0.41734	0.87102	0.88373	-0.17832	0.034115	0.33448	1.3993	0.48466
TAG504-FA182	0.015685	0.1372	0.39388	-1.3442	0.34575	0.99441	0.93256	-0.10073	0.13338	0.80329	0.59097	-0.75884	0.043491	0.33448	0.57014	-0.81062
DAG120/181	0.054149	0.19503	1.5273	0.61097	0.35653	0.99441	0.89297	-0.16332	0.2302	0.80329	0.81154	-0.30126	0.020153	0.32942	1.3829	0.46768
TAG563-FA182	0.003743	0.13256	0.27979	-1.8376	0.099327	0.99441	0.73795	-0.4384	0.008797	0.80329	0.32925	-1.6027	0.047962	0.33448	0.45441	-1.1379
DAG180/181	0.75527	0.86287	1.0743	0.10335	0.23153	0.99441	1.1442	0.19438	0.55486	0.89473	0.97074	-0.04284	0.018758	0.31888	1.2748	0.35032
ALLANTOIN_neg_2	0.43075	0.6401	1.5122	0.59666	0.16175	0.99441	1.445	0.53104	0.98728	0.99666	1.2138	0.27948	0.041824	0.33448	1.7998	0.84782
TAG400-FA140	0.013391	0.1372	1.6112	0.68816	0.95	0.99441	0.98933	-0.01547	0.48136	0.87857	1.0562	0.07889	0.046781	0.33448	1.3358	0.41775
TAG522-FA181	0.022253	0.14379	0.4631	-1.1106	0.35411	0.99441	0.87883	-0.18634	0.23267	0.80329	0.69644	-0.52192	0.03691	0.33448	0.5606	-0.83496
XANTHURENIC ACID_pos_2	0.022219	0.14379	0.67942	-0.55762	0.019796	0.99441	0.85596	-0.22439	0.85466	0.96928	1.3931	0.47825	0.001051	0.23372	0.46406	-1.1076
TAG541-FA200	4.22E-06	0.001795	0.32611	-1.6165	0.86769	0.99441	0.96011	-0.05873	0.24178	0.80329	0.70952	-0.49508	0.002734	0.23372	0.5804	-0.78487
TAG483-FA160	0.017988	0.1372	0.52734	-0.92319	0.33869	0.99441	0.8479	-0.23803	0.44908	0.87149	0.76779	-0.38121	0.007337	0.31888	0.61718	-0.69623
TAG481-FA160	0.20718	0.40577	0.84802	-0.23783	0.49502	0.99441	0.9387	-0.09126	0.99163	0.99678	1.0042	0.00608	0.025524	0.33448	0.8088	-0.30614
TAG462-FA120	0.27353	0.47839	0.87088	-0.19945	0.3245	0.99441	0.89132	-0.16599	0.97267	0.9957	1.0054	0.007736	0.045428	0.33448	0.79104	-0.33818
TAG512-FA160	0.31284	0.52661	1.2002	0.26329	0.89335	0.99441	1.0206	0.029462	0.42138	0.87102	0.92175	-0.11755	0.039845	0.33448	1.2658	0.34
2-KETOHEXANOIC ACID_pos_2	0.28336	0.49153	1.832	0.87338	0.14435	0.99441	1.5869	0.6662	0.796	0.95886	1.012	0.017184	0.049372	0.33448	2.2674	1.181
TAG561-FA160	0.01802	0.1372	0.33416	-1.5814	0.66007	0.99441	0.91698	-0.12504	0.90137	0.97744	0.68655	-0.54257	0.002655	0.23372	0.42007	-1.2513
TAG543-FA181	0.014733	0.1372	0.4579	-1.1269	0.25349	0.99441	0.88747	-0.17223	0.22211	0.80329	0.69887	-0.51691	0.014476	0.31888	0.56105	-0.83379
TAG522-FA160	0.023339	0.14379	0.43882	-1.1883	0.45821	0.99441	0.91003	-0.13601	0.25167	0.80329	0.67919	-0.55811	0.044376	0.33448	0.54913	-0.86479
TAG483-FA140	0.031021	0.15925	0.4339	-1.2046	0.67767	0.99441	1.044	0.062067	0.91144	0.97744	0.82932	-0.26999	0.049997	0.33448	0.56899	-0.81353
TAG533-FA182	0.010579	0.1372	0.57543	-0.79729	0.52865	0.99441	0.9344	-0.09789	0.19785	0.80329	0.77456	-0.36855	0.0375	0.33448	0.70753	-0.49914
TAG503-FA141	0.035591	0.15925	0.51578	-0.95518	0.54658	0.99441	0.90201	-0.14878	0.96855	0.99429	0.91688	-0.1252	0.007806	0.31888	0.54706	-0.87023
TAG512-FA182	0.088387	0.2694	0.59514	-0.74871	0.96072	0.99441	0.96918	-0.04516	0.65536	0.90034	0.92807	-0.10769	0.017978	0.31888	0.62341	-0.68174
TAG534-FA182	0.41247	0.62433	1.209	0.27383	0.94868	0.99441	1.0241	0.03432	0.3365	0.85641	0.89077	-0.16687	0.045841	0.33448	1.3173	0.39756
SM180	0.025164	0.144	0.60628	-0.72195	0.038441	0.99441	0.75398	-0.4074	0.04019	0.80329	0.67896	-0.5586	0.027882	0.33448	0.71155	-0.49097
TAG525-FA205	0.060172	0.20962	1.9126	0.93557	0.52293	0.99441	1.105	0.14409	0.80187	0.95886	0.95187	-0.07116	0.00275	0.23372	1.963	0.97305
TAG472-FA181	0.066479	0.22603	1.2993	0.37769	0.60085	0.99441	1.0599	0.08392	0.86987	0.97346	1.0112	0.01606	0.017963	0.31888	1.2844	0.3611
TAG502-FA180	0.004223	0.13316	0.47835	-1.0639	0.51563	0.99441	0.88638	-0.17401	0.38627	0.87102	0.75602	-0.40351	0.008719	0.31888	0.5595	-0.83779
TAG472-FA182	0.027018	0.14917	1.2735	0.34877	0.56608	0.99441	1.1004	0.13807	0.82674	0.96583	1.0889	0.12293	0.037406	0.33448	1.2192	0.2859
TAG526-FA181	0.40169	0.62418	1.2517	0.3239	0.78436	0.99441	1.0535	0.075256	0.42519	0.87102	0.9208	-0.11904	0.025708	0.33448	1.3532	0.43639
TAG565-FA225	0.26744	0.47163	1.1533	0.20574	0.90328	0.99441	0.94338	-0.08409	0.1926	0.80329	0.77113	-0.37495	0.029594	0.33448	1.3498	0.43272
L-ORNITHINE_pos_1	0.21627	0.41403	2.0904	1.0638	0.38386	0.99441	1.3454	0.42808	0.8457	0.96667	0.77885	-0.36058	0.035454	0.33448	2.6276	1.3937

Supplementary Table S2E. List of dysregulated metabolites for female, male, Wild type and APCHi mice following exposure to 9.5 Gy of Gamma-radiation, 6 months post-irradiation showing penotype dependent response of radiation.

	9.5Gy / 0.0Gy				9.5Gy / 0.0Gy				9.5Gy / 0.0Gy				9.5Gy / 0.0Gy			
	Female				Male				WildType				APCHi			
	6mo															
name	p-value	FDR	FC	LOG2FC	p-value	FDR	FC	LOG2FC	p-value	FDR	FC	LOG2FC	p-value	FDR	FC	LOG2FC
AMINOADIPATE_pos_1	0.34267	0.58212	0.95122	-0.07215	0.02556	0.54839	0.66344	-0.59197	0.31058	0.5617	1.6698	0.73971	0.036019	0.56697	0.42531	-1.2334
TAG527-FA160	0.004944	0.071904	1.2361	0.3058	0.10315	0.55082	0.84433	-0.24412	0.44956	0.66918	0.89842	-0.15454	0.040443	0.57294	1.1284	0.17429
TAG460-FA140	0.019348	0.10611	1.279	0.35497	0.87954	0.96019	1.0325	0.046153	0.42671	0.64769	1.0358	0.050781	0.048083	0.59265	1.2577	0.3308
TAG513-FA150	0.005164	0.071904	0.13814	-2.8558	0.54187	0.85329	0.85871	-0.21976	0.069294	0.3309	0.19924	-2.3275	0.022643	0.5398	0.29529	-1.7598
CHOLINE_pos_1	0.15023	0.34144	1.2631	0.337	0.28424	0.70059	1.6589	0.73023	0.93842	0.96751	1.1714	0.22819	0.033379	0.56697	1.6611	0.73217
TAG440-FA160	0.013333	0.094441	1.3623	0.446	0.79438	0.95228	1.0306	0.043491	0.40339	0.6303	1.0395	0.055837	0.036016	0.56697	1.3177	0.39802
TAG440-FA120	0.009268	0.07792	1.3873	0.47224	0.75107	0.93951	1.0845	0.11708	0.34305	0.57989	1.0637	0.089129	0.010686	0.5398	1.3863	0.47121
TAG440-FA140	0.005377	0.071904	1.3488	0.43165	0.94604	0.97514	1.0667	0.093156	0.31583	0.56636	1.0588	0.082443	0.016246	0.5398	1.3407	0.42294
TAG523-FA161	0.007104	0.07364	0.36093	-1.4702	0.55235	0.85329	0.96589	-0.05007	0.033121	0.26873	0.51489	-0.95766	0.047097	0.59265	0.50492	-0.98588
TAG501-FA180	0.053682	0.18855	0.73033	-0.45337	0.011116	0.39402	0.82748	-0.2732	0.006611	0.1261	0.72176	-0.47041	0.04919	0.59265	0.76798	-0.38087
TAG491-FA181	0.006479	0.072593	0.80691	-0.30952	0.03644	0.54839	0.85745	-0.22187	0.00222	0.096167	0.79923	-0.32331	0.035101	0.56697	0.84761	-0.23853
TAG421-FA181	0.005541	0.071904	1.6527	0.72487	0.80888	0.95228	1.0966	0.13304	0.16494	0.43882	1.1289	0.1749	0.029347	0.54228	1.5122	0.59665
TAG512-FA150	0.001228	0.047281	0.35394	-1.4984	0.76487	0.95206	0.90386	-0.14582	0.10419	0.37691	0.43177	-1.2117	0.010935	0.5398	0.56235	-0.83047
TAG511-FA181	0.003008	0.060885	0.66244	-0.59414	0.075178	0.54839	0.88644	-0.17391	0.001882	0.096167	0.67619	-0.56449	0.039973	0.57294	0.80733	-0.30877
TAG492-FA160	0.000423	0.029944	0.65523	-0.60992	0.12264	0.57117	0.9128	-0.13162	0.010606	0.15025	0.76294	-0.39037	0.002934	0.5398	0.7642	-0.38798
TAG400-FA160	0.002883	0.060885	1.5297	0.61328	0.78908	0.95228	1.1269	0.17236	0.2779	0.5393	1.0887	0.12262	0.012	0.5398	1.5126	0.59699
TAG420-FA140	0.006873	0.073023	1.3512	0.43429	0.80296	0.95228	1.0744	0.10351	0.25649	0.5191	1.0767	0.10666	0.018949	0.5398	1.3408	0.42315
N-ACETYLALANINE_neg_1	0.001572	0.047281	0.40158	-1.3162	0.19231	0.62869	0.7937	-0.33333	0.085356	0.3448	0.73504	-0.4441	0.017134	0.5398	0.51894	-0.94637
TAG400-FA140	0.00228	0.052941	1.4558	0.54183	0.28847	0.70059	1.1593	0.21329	0.14308	0.41651	1.1504	0.20216	0.002668	0.5398	1.4613	0.54729
TAG400-FA120	0.008994	0.07792	1.4171	0.50298	0.2319	0.67154	1.1688	0.22498	0.12844	0.39984	1.1546	0.20734	0.008467	0.5398	1.4414	0.52743
TAG492-FA182	0.019878	0.10611	0.58132	-0.78261	0.18924	0.62869	0.8946	-0.16069	0.12313	0.39984	0.69617	-0.52249	0.020146	0.5398	0.74807	-0.41875
TAG503-FA161	0.004343	0.071904	0.52141	-0.93952	0.40944	0.80947	0.93463	-0.09753	0.045277	0.29047	0.72834	-0.45733	0.025691	0.54228	0.62725	-0.67288
TAG421-FA120	0.024865	0.12274	1.3127	0.39248	0.3303	0.75069	1.1706	0.22726	0.17381	0.4469	1.1213	0.16522	0.023553	0.5398	1.4213	0.50722
TAG420-FA120	0.005524	0.071904	1.3689	0.45299	0.23579	0.67154	1.1836	0.24319	0.14256	0.41651	1.141	0.1903	0.004973	0.5398	1.4351	0.52112
TAG492-FA150	0.001013	0.04305	0.57997	-0.78595	0.14739	0.59096	0.88981	-0.16843	0.002365	0.096167	0.66411	-0.5905	0.029262	0.54228	0.74473	-0.4252
CER160	1.23E-05	0.004552	0.10801	-3.2107	0.28965	0.70059	1.2682	0.34276	0.074951	0.33329	0.27215	-1.8775	0.009063	0.5398	0.33228	-1.5895
TAG503-FA182	0.00577	0.071904	0.41085	-1.2833	0.10117	0.55082	0.88469	-0.17676	0.004666	0.098784	0.5313	-0.91241	0.044907	0.59265	0.57111	-0.80816
TAG493-FA182	0.021591	0.11106	0.77148	-0.37429	0.6906	0.90885	0.94592	-0.08021	0.9402	0.96751	0.93549	-0.09621	0.024132	0.5398	0.79533	-0.33037
TAG512-FA170	0.001669	0.047281	0.5679	-0.8163	0.38945	0.79813	0.93398	-0.09854	0.03102	0.26735	0.6853	-0.54518	0.02767	0.54228	0.74472	-0.42522
CER220	9.1E-05	0.009668	0.10827	-3.2073	0.8077	0.95228	0.89453	-0.1608	0.003484	0.096167	0.12673	-2.9801	0.022114	0.5398	0.21186	-2.2388
TAG525-FA205	0.009611	0.07792	1.4543	0.54028	0.10887	0.55082	1.4957	0.58078	0.049199	0.29221	1.289	0.36628	0.017638	0.5398	1.7611	0.81652
TAG513-FA182	0.001614	0.047281	0.3535	-1.5002	0.093341	0.54839	0.8645	-0.21006	0.004731	0.098784	0.42901	-1.2209	0.012482	0.5398	0.54851	-0.8664
TAG492-FA140	0.55177	0.73743	1.0665	0.092842	0.00816	0.34678	1.347	0.42971	0.17973	0.4574	1.1429	0.19267	0.037586	0.57051	1.3506	0.43364
TAG503-FA181	0.021689	0.11106	0.71589	-0.48219	0.12335	0.57117	0.91509	-0.12801	0.053557	0.29221	0.83752	-0.25581	0.023486	0.5398	0.7574	-0.40088

Supplementary Table S3A. List of dysregulated metabolites for female mice following exposure to 9.5 Gy of Gamma-radiation, 24 h, 1 week, 1 month, 3 month and 6 months post-irradiation.

	9.5Gy / 0.0Gy				9.5Gy / 0.0Gy				9.5Gy / 0.0Gy				9.5Gy / 0.0Gy				9.5Gy / 0.0Gy			
	24h				1wk				1mo				3mo				6mo			
	Female																			
name	p-value	FDR	FC	LOG2FC	p-value	FDR	FC	LOG2FC	p-value	FDR	FC	LOG2FC	p-value	FDR	FC	LOG2FC	p-value	FDR	FC	LOG2FC
XANTHOSINE_neg_1	3.2E-08	1.36E-05	4.6848 ↑	2.228	0.88496	0.98297	0.93958 ↓	-0.09	0.68509	0.99953	1.0607 ↑	0.085	0.14544	0.3415	0.66773 ↓	-0.583	0.68019	0.85048	1.1155 ↑	0.1577
CYSTEINE_neg_5	6.97E-07	0.000148	3.3658 ↑	1.7509	0.67261	0.97562	1.0865 ↑	0.1198	0.39023	0.99953	0.8642 ↓	-0.211	0.14091	0.33558	1.3473 ↑	0.4301	0.95458	0.98388	0.96587 ↓	-0.05
PYROPHOSPHATE_neg_3	2.68E-05	0.003799	3.4684 ↑	1.7943	0.61496	0.97562	1.3679 ↑	0.452	0.14722	0.91055	1.5253 ↑	0.6091	0.18751	0.38685	0.80233 ↓	-0.318	0.43869	0.6588	1.0687 ↑	0.0959
N-ACETYLGLUTAMINE_pos_2	8.84E-05	0.009395	6.7286 ↑	2.7503	0.79526	0.98297	0.78748 ↓	-0.345	0.064659	0.87596	2.4339 ↑	1.2833	0.11043	0.2916	1.7889 ↑	0.8391	0.072779	0.22304	1.8374 ↑	0.8777
INDOLE-3-CARBOXYLIC ACID_neg_1	0.000493	0.041908	1.8298 ↑	0.8717	0.23707	0.95194	1.2021 ↑	0.2656	0.11093	0.89093	1.2226 ↑	0.29	0.2221	0.41821	1.2237 ↑	0.2912	0.91486	0.9732	0.88097 ↓	-0.183
CE204	0.002393	0.16951	5.9315 ↑	2.5684	0.16098	0.95194	2.2096 ↑	1.1438	0.11694	0.89093	3.2394 ↑	1.6957	0.58502	0.73583	0.81133 ↓	-0.302	0.043476	0.16498	0.73603 ↓	-0.442
N-ACETYLORNITHINE_neg_3	0.003118	0.18928	2.1245 ↑	1.0871	0.96042	0.98357	1.0483 ↑	0.068	0.000196	0.083154	0.37793 ↓	-1.404	0.056825	0.20295	1.7991 ↑	0.8473	0.74547	0.88952	1.4685 ↑	0.5543
OXOGLUTARATE_neg_2	0.004755	0.25259	0.20202 ↓	-2.307	0.034941	0.95194	0.28826 ↓	-1.795	0.49713	0.99953	1.2927 ↑	0.3704	0.50156	0.68985	0.94293 ↓	-0.085	0.087383	0.24116	0.71599 ↓	-0.482
CE182	0.005901	0.27867	2.2682 ↑	1.1816	0.62544	0.97562	1.199 ↑	0.2618	0.1903	0.93892	1.795 ↑	0.844	0.56063	0.73066	1.1159 ↑	0.1583	0.052314	0.18683	0.7843 ↓	-0.351
NORMETANEPHRINE_pos_1	0.006909	0.29364	2.0563 ↑	1.0401	0.19929	0.95194	2.3139 ↑	1.2103	0.42754	0.99953	0.41801 ↓	-1.258	0.78533	0.87833	1.1398 ↑	0.1887	0.025126	0.12274	1.6771 ↑	0.746
ASCORBATE_neg_2	0.007663	0.29606	3.1427 ↑	1.652	0.93711	0.98297	1.3343 ↑	0.4161	0.10102	0.89093	0.80214 ↓	-0.318	0.54456	0.72099	0.71944 ↓	-0.475	0.91825	0.9732	0.92817 ↓	-0.108
ACETYL-COA_pos_1	0.012974	0.45949	2.4651 ↑	1.3016	0.094709	0.95194	1.5474 ↑	0.6298	0.64969	0.99953	2.5213 ↑	1.3342	0.46698	0.66904	1.5207 ↑	0.6048	0.74719	0.88952	0.98153 ↓	-0.027
N-GLYCYL-L-PROLINE_neg_3	0.017907	0.58541	2.7965 ↑	1.4836	0.87897	0.98297	0.63118 ↓	-0.664	0.22238	0.95973	1.5106 ↑	0.5951	0.17601	0.38685	1.4758 ↑	0.5615	0.42407	0.6483	0.56107 ↓	-0.834
AMINOADIPATE_pos_1	0.020786	0.63101	2.2144 ↑	1.1469	0.083223	0.95194	0.34915 ↓	-1.518	0.58947	0.99953	1.3954 ↑	0.4807	0.80078	0.88628	3.4808 ↑	1.7994	0.34267	0.58212	0.95122 ↓	-0.072
L-CARNITINE_pos_1	0.022692	0.64294	4.0538 ↑	2.0193	0.93182	0.98297	0.74546 ↓	-0.424	0.73067	0.99953	0.96537 ↓	-0.051	0.99868	0.99868	1.7876 ↑	0.838	0.89231	0.96531	1.3288 ↑	0.4102
CE226	0.027135	0.697	2.729 ↑	1.4484	0.67435	0.97562	1.1055 ↑	0.1448	0.37269	0.99953	1.6446 ↑	0.7178	0.006099	0.1372	2.7635 ↑	1.4665	0.059905	0.19456	1.6599 ↑	0.7311
TAG566-FA225	0.02788	0.697	1.5091 ↑	0.5937	0.97292	0.98479	1.0034 ↑	0.0048	0.43322	0.99953	0.71244 ↓	-0.489	0.15081	0.35025	0.79429 ↓	-0.332	0.52093	0.71418	0.83292 ↓	-0.264
GERANYL-PP_-HPO3_neg_2	0.035002	0.79883	1.7401 ↑	0.7991	0.48233	0.95194	0.73966 ↓	-0.435	0.11669	0.89093	2.0914 ↑	1.0645	0.94662	0.98125	1.3726 ↑	0.4569	0.48426	0.68679	0.88212 ↓	-0.181
TAG544-FA224	0.035713	0.79883	1.3368 ↑	0.4187	0.51234	0.97562	1.4422 ↑	0.5283	0.013325	0.62598	0.38637 ↓	-1.372	0.46877	0.66904	1.3295 ↑	0.4109	0.86436	0.95915	1.1016 ↑	0.1396
ACETYLPHOSPHATE_pos_1	0.041064	0.87261	0.55006 ↓	-0.862	0.26551	0.95194	0.80639 ↓	-0.31	0.59464	0.99953	1.1401 ↑	0.1892	0.59481	0.74351	1.1232 ↑	0.1676	0.23042	0.45128	0.83663 ↓	-0.257
N-ACETYLPUTRESCINE_pos_1	0.049127	0.95219	0.10127 ↓	-3.304	0.65039	0.97562	0.93202 ↓	-0.102	0.59714	0.99953	0.95079 ↓	-0.073	0.10744	0.29083	1.4687 ↑	0.5545	0.98156	0.99089	1.1382 ↑	0.1868
TAG522-FA202	0.050845	0.95219	1.3133 ↑	0.3932	0.6287	0.97562	1.5137 ↑	0.5981	0.008694	0.61582	0.3735 ↓	-1.421	0.21117	0.40795	1.6024 ↑	0.6802	0.50108	0.70516	1.0996 ↑	0.1369
TAG565-FA181	0.057481	0.95219	2.7694 ↑	1.4696	0.58603	0.97562	0.47221 ↓	-1.083	0.47983	0.99953	0.8001 ↓	-0.322	0.00351	0.13256	0.15593 ↓	-2.681	0.003245	0.062679	0.14568 ↓	-2.779
TAG582-FA181	0.082354	0.96989	1.7279 ↑	0.789	0.29267	0.95194	0.49568 ↓	-1.013	0.97481	0.99953	0.94037 ↓	-0.089	0.006539	0.1372	0.19481 ↓	-2.36	0.009489	0.07792	0.29764 ↓	-1.748
TAG527-FA160	0.082836	0.96989	0.8191 ↓	-0.288	0.29285	0.95194	1.2397 ↑	0.31	0.31004	0.99215	0.76025 ↓	-0.395	0.29759	0.51205	1.0911 ↑	0.1258	0.004944	0.071904	1.2361 ↑	0.3058
TAG545-FA205	0.092485	0.96989	0.50992 ↓	-0.972	0.019211	0.95194	1.9741 ↑	0.9812	0.14665	0.91055	0.53627 ↓	-0.899	0.40516	0.62418	0.90818 ↓	-0.139	0.462	0.67408	1.2898 ↑	0.3672
TAG521-FA201	0.095023	0.96989	1.4201 ↑	0.506	0.94371	0.98297	1.0156 ↑	0.0223	0.002293	0.46622	0.54014 ↓	-0.889	0.18658	0.38685	0.83588 ↓	-0.259	0.46313	0.67408	0.77546 ↓	-0.367
TAG521-FA161	0.12162	0.96989	1.112 ↑	0.1531	0.54025	0.97562	1.0011 ↑	0.0016	0.008581	0.61582	0.58627 ↓	-0.77	0.9125	0.96231	1.099 ↑	0.1362	0.10837	0.27253	0.83986 ↓	-0.252
TAG462-FA182	0.13404	0.96989	0.86917 ↓	-0.202	0.61429	0.97562	1.0685 ↑	0.0955	0.68986	0.99953	0.97463 ↓	-0.037	0.81042	0.88877	1.0924 ↑	0.1275	0.038752	0.1543	1.2727 ↑	0.3479
SM160	0.14303	0.96989	0.74695 ↓	-0.421	0.023922	0.95194	1.9239 ↑	0.9441	0.054429	0.87596	1.5577 ↑	0.6394	0.11185	0.29163	1.3709 ↑	0.4551	0.037528	0.1543	1.394 ↑	0.4792
TAG441-FA161	0.14717	0.96989	0.85804 ↓	-0.221	0.69408	0.97676	0.75457 ↓	-0.406	0.78633	0.99953	1.1549 ↑	0.2077	0.034831	0.15925	1.4501 ↑	0.5362	0.19013	0.39917	1.0939 ↑	0.1295
METANEPHRINE_pos_1	0.14789	0.96989	1.3314 ↑	0.4129	0.024607	0.95194	0.79574 ↓	-0.33	0.14086	0.91055	1.1402 ↑	0.1893	0.76274	0.86443	0.88925 ↓	-0.169	0.41516	0.64261	1.1487 ↑	0.2
TAG564-FA204	0.16308	0.96989	1.2441 ↑	0.3151	0.60423	0.97562	1.4779 ↑	0.5635	0.2361	0.985	0.65622 ↓	-0.608	0.14923	0.34847	0.79099 ↓	-0.338	0.022749	0.1151	0.67357 ↓	-0.57
PYRIDOXAMINE_pos_1	0.17121	0.96989	1.242 ↑	0.3127	0.92673	0.98297	0.86981 ↓	-0.201	0.76521	0.99953	4.3561 ↑	2.123	0.017691	0.1372	0.4036 ↓	-1.309	0.82736	0.93768	0.95739 ↓	-0.063
TAG422-FA120	0.17293	0.96989	0.77015 ↓	-0.377	0.56333	0.97562	1.1116 ↑	0.1527	0.28434	0.99215	0.58992 ↓	-0.761	0.018672	0.1372	1.7611 ↑	0.8165	0.25181	0.48425	1.1831 ↑	0.2426
L-2-HYDROXYGLUTARIC ACID_neg_1	0.17343	0.96989	0.028371 ↓	-5.14	0.12025	0.95194	1.2941 ↑	0.372	0.56571	0.99953	0.67398 ↓	-0.569	0.049587	0.18816	0.33158 ↓	-1.593	0.7774	0.91018	0.85762 ↓	-0.222
CE181	0.17672	0.96989	1.1604 ↑	0.2147	0.94525	0.98297	1.0198 ↑	0.0283	0.64468	0.99953	1.219 ↑	0.2857	0.22051	0.41821	0.79966 ↓	-0.323	0.009499	0.07792	0.73441 ↓	-0.445
TAG523-FA180	0.19009	0.96989	1.6064 ↑	0.6838	0.83643	0.98297	0.60178 ↓	-0.733	0.9185	0.99953	1.0828 ↑	0.1147	0.013141	0.1372	0.1748 ↓	-2.516	0.12916	0.30665	0.26507 ↓	-1.916
TAG460-FA140	0.19104	0.96989	0.8637 ↓	-0.211	0.36514	0.95901	1.1494 ↑	0.2009	0.91433	0.99953	1.0016 ↑	0.0023	0.032218	0.15925	1.2558 ↑	0.3286	0.019348	0.10611	1.279 ↑	0.355
TAG421-FA160	0.20321	0.96989	0.87122 ↓	-0.199	0.4378	0.97562	1.3302 ↑	0.4116	0.94908	0.99953	1.2326 ↑	0.3017	0.2725	0.47839	1.3587 ↑	0.4422	0.00931	0.07792	1.8154 ↑	0.8603
TAG442-FA181	0.20445	0.96989	0.84866 ↓	-0.237	0.31223	0.95194	1.3902 ↑	0.4753	0.63393	0.99953	1.5932 ↑	0.6719	0.56934	0.73416	1.2501 ↑	0.3221	0.02936	0.13417	1.717 ↑	0.7799
TAG505-FA205	0.21143	0.96989	0.6573 ↓	-0.605	0.025203	0.95194	1.8226 ↑	0.866	0.12914	0.91055	0.65029 ↓	-0.621	0.84961	0.91645	1.0452 ↑	0.0638	0.37745	0.60529	1.1231 ↑	0.1675
TAG490-FA170	0.21997	0.96989	0.86083 ↓	-0.216	0.5611	0.97562	1.0939 ↑	0.1294	0.58323	0.99953	0.88872 ↓	-0.17	0.024343	0.14379	1.3098 ↑	0.3893	0.019973	0.10611	1.2343 ↑	0.3037
TAG511-FA180	0.22746	0.96989	1.2997 ↑	0.3782	0.31564	0.95194	0.74595 ↓	-0.423	0.78282	0.99953	0.99252 ↓	-0.011	0.47767	0.67222	0.80905 ↓	-0.306	0.008748	0.07792	0.64923 ↓	-0.623
TAG480-FA140	0.23917	0.96989	0.90072 ↓	-0.151	0.1411	0.95194	1.2453 ↑	0.3164	0.70004	0.99953	0.92426 ↓	-0.114	0.15561	0.35555	1.1579 ↑	0.2116	0.037957	0.1543	1.319 ↑	0.3994
TAG504-FA181	0.24019	0.96989	0.78486 ↓	-0.349	0.71191	0.98297	0.97814 ↓	-0.032	0.80412	0.99953	0.85969 ↓	-0.218	0.018816	0.1372	0.64605 ↓	-0.63	0.66809	0.84005	0.92726 ↓	-0.109
HOMOCYSTEINE_pos_2	0.24598	0.96989	1.2222 ↑	0.2895	0.74685	0.98297	0.93111 ↓	-0.103	0.035008	0.7334	2.1908 ↑	1.1315	0.001573	0.13256	2.6966 ↑	1.4311	0.95368	0.98388	1.0003 ↑	0.0004
TAG568-FA160	0.24721	0.96989	1.3753 ↑	0.4597	0.89376	0.98297	0.93884 ↓	-0.091	0.90617	0.99953	1.0432 ↑	0.061	0.06171	0.21322	0.63361 ↓	-0.658	0.018626	0.10611	0.59799 ↓	-0.742
TAG562-FA180	0.25467	0.96989	1.486 ↑	0.5714	0.66989	0.97562	0.72759 ↓	-0.459	0.6052	0.99953	0.77675 ↓	-0.364	0.000732	0.13256	0.35394 ↓	-1.498	0.12449	0.30062	0.56405 ↓	-0.826
TAG461-FA181	0.26224	0.96989	0.90205 ↓	-0.149	0.76714	0.98297	1.082 ↑	0.1137	0.43465	0.99953	1.3549 ↑	0.4381	0.24023	0.43819	1.229 ↑	0.2975	0.019549	0.10611	1.4813 ↑	0.5669
TAG441-FA160	0.2667	0.96989	0.90791 ↓	-0.139	0.47604	0.97562	1.2053 ↑	0.2694	0.75018	0.99953	1.0934 ↑	0.1289	0.10729	0.29083	1.2574 ↑	0.3304	0.011895	0.090273	1.5277 ↑	0.6114
TAG491-FA150	0.27364	0.96989	1.1916 ↑	0.2529	0.038576	0.95194	0.61163 ↓	-0.709	0.81097	0.99953	0.94992 ↓	-0.074	0.19449	0.39175	0.82566 ↓	-0.276	0.007947	0.076901	0.69681 ↓	-0.521
TAG461-FA140	0.27686	0.96989	0.91281 ↓	-0.132	0.71665	0.98297	0.81806 ↓	-0.29	0.3954	0.99953	1.23 ↑	0.2987	0.10137	0.28532	1.2801 ↑	0.3562	0.01985	0.10611	1.2204 ↑	0.2874
TAG450-FA160	0.27965	0.96989	0.87912 ↓	-0.186	0.53603	0.97562	1.0893 ↑	0.1235	0.9071	0.99953	0.95634 ↓	-0.064	0.029162	0.15701	1.3202 ↑	0.4007	0.037341	0.1543	1.2301 ↑	0.2988
TAG441-FA120	0.28101	0.96989	0.89293 ↓	-0.163	0.73179	0.98297	0.9449 ↓	-0.082	0.92104	0.99953	0.91859 ↓	-0.123	0.08054	0.251	1.4571 ↑	0.5431	0.002367	0.052941	1.4454 ↑	0.5315
TAG441-FA140	0.28195	0.96989	0.91622 ↓	-0.126	0.94154	0.98297	0.84366 ↓	-0.245	0.7056	0.99953	1.1081 ↑	0.1481	0.012778	0.1372	1.4722 ↑	0.558	0.016834	0.10149	1.2857 ↑	0.3626
TAG461-FA120	0.28318	0.96989	0.90973 ↓	-0.136	0.75018	0.98297	0.9876 ↓	-0.018	0.67734	0.99953	1.1695 ↑	0.2259	0.40475	0.62418	1.1717 ↑	0.2286	0.042121	0.16364	1.3404 ↑	0.4227
TAG513-FA150	0.287	0.96989	1.816 ↑	0.8608	0.73193	0.98297	0.42296 ↓	-1.241	0.91973	0.99953	0.83447 ↓	-0.261	0.041889	0.17454	0.3037 ↓	-1.719	0.005164	0.071904	0.13814 ↓	-2.856
TAG567-FA204	0.29099	0.96989	1.4724 ↑	0.5582	0.86896	0.98297	0.85331 ↓	-0.229	0.63789	0.99953	0.97026 ↓	-0.044	0.005013	0.13316	0.133 ↓	-2.911	0.003931	0.071904	0.27661 ↓	-1.854
TAG440-FA160	0.29163	0.96989	0.89531 ↓	-0.16	0.26217	0.95194	1.2094 ↑	0.2742	0.84531	0.99953	1.029 ↑	0.0413	0.035971	0.15925	1.3157 ↑	0.3959	0.013333	0.094441	1.3623 ↑	0.446
TAG450-FA140	0.29311	0.96989	0.87607 ↓	-0.191	0.59867	0.97562	1.0472 ↑	0.0666	0.96668	0.99953	1.0116 ↑	0.0167	0.034557	0.15925	1.3222 ↑	0.403	0.042354	0.16364	1.2293 ↑	0.2978
TAG541-FA160	0.29366	0.96989	1.2696 ↑	0.3443	0.087808	0.95194	0.59365 ↓	-0.752	0.56561	0.99953	1.0772 ↑	0.1073	0.002339	0.13256	0.3547 ↓	-1.495	0.085799	0.2399	0.58614 ↓	-0.771
TAG451-FA181	0.29575	0.96989	0.9121 ↓	-0.133	0.27563	0.95194	1.2623 ↑	0.3361	0.53123	0.99953	0.81137 ↓	-0.302	0.071345	0.23511	1.2965 ↑	0.3746	0.036897	0.1543	1.2254 ↑	0.2933
TAG440-FA120	0.30086	0.96989	0.9047 ↓	-0.144	0.21453	0.95194	1.2449 ↑	0.316	0.91022	0.99953	0.97207 ↓	-0.041	0.042852	0.17665	1.3167 ↑	0.397	0.009268	0.07792	1.3873 ↑	0.4722
TAG564-FA201	0.3073	0.96989	1.24 ↑	0.3103	0.9014	0.98297	0.69683 ↓	-0.521	0.85951	0.99953	0.81928 ↓	-0.288	0.004763	0.13316	0.18962 ↓	-2.399	0.24889	0.48081	0.38961 ↓	-1.36
TAG442-FA140	0.30906	0.96989	0.89325 ↓	-0.163	0.91424	0.98297	0.8402 ↓	-0.251	0.70234	0.99953	0.8411 ↓	-0.25	0.015245	0.1372	1.6312 ↑	0.7059	0.13196	0.30876	1.307 ↑	0.3862
TAG440-FA140	0.31347	0.96989	0.89762 ↓	-0.156	0.4285	0.97562	1.1167 ↑	0.1592	0.89734	0.99953	1.0237 ↑	0.0338	0.01913	0.1372	1.3588 ↑	0.4423	0.005377	0.071904	1.3488 ↑	0.4317
TAG460-FA160	0.31925	0.96989	0.89631 ↓	-0.158	0.32488	0.95194	1.1586 ↑	0.2123	0.97918	0.99953	0.97623 ↓	-0.035	0.03451	0.15925	1.2456 ↑	0.3168	0.014441	0.10041	1.28 ↑	0.3561
SM261	0.32155	0.96989	0.88333 ↓	-0.179	0.80356	0.98297	0.93183 ↓	-0.102	0.082415	0.89093	0.53195 ↓	-0.911	0.046843	0.17935	3.3918 ↑	1.7621	0.81364	0.92907	1.009 ↑	0.0129
TAG532-FA170	0.34796	0.96989	1.1687 ↑	0.2249	0.84128	0.98297	0.91976 ↓	-0.121	0.99765	0.99953	1.0099 ↑	0.0142	0.71281	0.82777	0.96368 ↓	-0.053	0.029006	0.13417	0.70537 ↓	-0.504
TAG461-FA160	0.35052	0.96989	0.92814 ↓	-0.108	0.81229	0.98297	0.86578 ↓	-0.208	0.51914	0.99953	1.1658 ↑	0.2213	0.097164	0.27715	1.218 ↑	0.2846	0.037076	0.1543	1.2219 ↑	0.2891
TAG525-FA161	0.35296	0.96989	1.1682 ↑	0.2242	0.68963	0.97602	0.5135 ↓	-0.962	0.82323	0.99953	1.0755 ↑	0.105	0.012458	0.1372	0.12542 ↓	-2.995	0.01066	0.082376	0.15367 ↓	-2.702
TAG511-FA150	0.35376	0.96989	1.2497 ↑	0.3216	0.13188	0.95194	0.63342 ↓	-0.659	0.89318	0.99953	0.92568 ↓	-0.111	0.011052	0.1372	0.63822 ↓	-0.648	0.016594	0.10149	0.51748 ↓	-0.95
TAG470-FA140	0.36835	0.96989	0.90668 ↓	-0.141	0.58217	0.97562	1.0609 ↑	0.0853	0.65681	0.99953	1.0857 ↑	0.1186	0.018934	0.1372	1.3897 ↑	0.4748	0.02718	0.12979	1.2394 ↑	0.3097
TAG512-FA181	0.3718	0.96989	1.0719 ↑	0.1001	0.60746	0.97562	1.0961 ↑	0.1324	0.003291	0.46622	0.69702 ↓	-0.521	0.76206	0.86443	1.0511 ↑	0.0719	0.31076	0.55199	0.9288 ↓	-0.107
TAG522-FA161	0.37227	0.96989	1.367 ↑	0.451	0.21781	0.95194	0.60396 ↓	-0.727	0.88238	0.99953	0.91628 ↓	-0.126	0.009489	0.1372	0.3493 ↓	-1.518	0.009934	0.078187	0.45676 ↓	-1.131
TAG542-FA202	0.37253	0.96989	1.1188 ↑	0.1619	0.9732	0.98479	0.99605 ↓	-0.006	0.030454	0.7334	0.66534 ↓	-0.588	0.76148	0.86443	1.0542 ↑	0.0762	0.88043	0.96438	1.0773 ↑	0.1074
TAG523-FA161	0.37591	0.96989	1.3958 ↑	0.4811	0.21673	0.95194	0.55671 ↓	-0.845	0.98312	0.99953	0.93817 ↓	-0.092	0.014427	0.1372	0.27612 ↓	-1.857	0.007104	0.07364	0.36093 ↓	-1.47
TAG420-FA160	0.37945	0.96989	0.91814 ↓	-0.123	0.20897	0.95194	1.2986 ↑	0.3769	0.86495	0.99953	1.0272 ↑	0.0387	0.090925	0.27151	1.3453 ↑	0.4279	0.009717	0.07792	1.4803 ↑	0.5659
TAG564-FA181	0.3824	0.96989	1.4647 ↑	0.5506	0.65922	0.97562	0.61671 ↓	-0.697	0.31246	0.99215	0.73698 ↓	-0.44	0.004879	0.13316	0.19081 ↓	-2.39	0.084001	0.23821	0.28405 ↓	-1.816
TAG440-FA180	0.40001	0.96989	0.90864 ↓	-0.138	0.19881	0.95194	1.3391 ↑	0.4213	0.9439	0.99953	1.0814 ↑	0.1129	0.080271	0.251	1.3902 ↑	0.4753	0.001848	0.049077	1.5481 ↑	0.6305
TAG441-FA181	0.40198	0.96989	0.96293 ↓	-0.055	0.67405	0.97562	1.1698 ↑	0.2262	0.53218	0.99953	1.4642 ↑	0.5501	0.18589	0.38685	1.3849 ↑	0.4698	0.016041	0.10149	1.5905 ↑	0.6694
TAG491-FA181	0.40917	0.96989	1.084 ↑	0.1164	0.16779	0.95194	0.75957 ↓	-0.397	0.29069	0.99215	1.1251 ↑	0.17	0.52048	0.69561	0.93727 ↓	-0.093	0.006479	0.072593	0.80691 ↓	-0.31
TAG553-FA181	0.41911	0.96989	1.097 ↑	0.1336	0.17943	0.95194	1.4517 ↑	0.5377	0.020484	0.66968	0.67082 ↓	-0.576	0.74423	0.85255	1.0821 ↑	0.1139	0.28336	0.52133	0.9074 ↓	-0.14
TAG541-FA181	0.42214	0.96989	1.0667 ↑	0.0931	0.63928	0.97562	1.1011 ↑	0.139	0.036478	0.7334	0.73758 ↓	-0.439	0.94512	0.98125	1.0574 ↑	0.0805	0.81121	0.92907	1.029 ↑	0.0413
TAG421-FA181	0.42415	0.96989	0.95305 ↓	-0.069	0.45778	0.97562	1.3567 ↑	0.4401	0.82449	0.99953	1.3086 ↑	0.3881	0.26015	0.46261	1.3222 ↑	0.403	0.005541	0.071904	1.6527 ↑	0.7249
TAG422-FA182	0.4256	0.96989	0.91278 ↓	-0.132	0.91195	0.98297	1.0502 ↑	0.0707	0.74244	0.99953	1.2411 ↑	0.3116	0.032187	0.15925	1.5825 ↑	0.6622	0.000527	0.031712	1.7766 ↑	0.8292
TAG546-FA160	0.42603	0.96989	1.3918 ↑	0.4769	0.83098	0.98297	0.81488 ↓	-0.295	0.19154	0.93892	1.3641 ↑	0.4479	0.017719	0.1372	0.35419 ↓	-1.497	0.073202	0.22304	0.3537 ↓	-1.499
TAG421-FA140	0.42924	0.96989	0.92339 ↓	-0.115	0.72274	0.98297	0.98861 ↓	-0.017	0.24058	0.99215	1.2902 ↑	0.3676	0.007759	0.1372	1.5621 ↑	0.6435	0.004699	0.071904	1.4968 ↑	0.5819
TAG470-FA160	0.42967	0.96989	0.90691 ↓	-0.141	0.58539	0.97562	1.0828 ↑	0.1148	0.72424	0.99953	1.0403 ↑	0.057	0.043522	0.17665	1.2508 ↑	0.3228	0.16545	0.36739	1.1089 ↑	0.1492
TAG512-FA150	0.43667	0.96989	1.3897 ↑	0.4748	0.29734	0.95194	0.58824 ↓	-0.766	0.81745	0.99953	0.88074 ↓	-0.183	0.078593	0.25016	0.49008 ↓	-1.029	0.001228	0.047281	0.35394 ↓	-1.498
TAG562-FA160	0.446	0.96989	1.2726 ↑	0.3478	0.38662	0.95901	0.63489 ↓	-0.655	0.9346	0.99953	1.0042 ↑	0.0061	0.001492	0.13256	0.21407 ↓	-2.224	0.074521	0.22304	0.45925 ↓	-1.123
TAG470-FA170	0.44674	0.96989	0.89958 ↓	-0.153	0.63915	0.97562	1.0382 ↑	0.054	0.63686	0.99953	0.87804 ↓	-0.188	0.035688	0.15925	1.3256 ↑	0.4067	0.081274	0.23659	1.1737 ↑	0.2311
TAG547-FA183	0.45216	0.96989	0.30028 ↓	-1.736	0.91934	0.98297	0.65552 ↓	-0.609	0.27741	0.99215	0.75049 ↓	-0.414	0.33769	0.55843	1.4611 ↑	0.5471	0.016956	0.10149	1.9368 ↑	0.9537
TAG493-FA160	0.45645	0.96989	1.1473 ↑	0.1982	0.19163	0.95194	0.79472 ↓	-0.331	0.71087	0.99953	1.0733 ↑	0.102	0.00194	0.13256	0.65282 ↓	-0.615	0.48479	0.68679	0.86209 ↓	-0.214
TAG525-FA181	0.45674	0.96989	0.84127 ↓	-0.249	0.8915	0.98297	1.0159 ↑	0.0228	0.031602	0.7334	0.72885 ↓	-0.456	0.18069	0.38685	0.78284 ↓	-0.353	0.34379	0.58212	0.89786 ↓	-0.155
TAG492-FA170	0.45991	0.96989	0.93494 ↓	-0.097	0.22079	0.95194	0.62927 ↓	-0.668	0.78867	0.99953	1.0119 ↑	0.0171	0.045831	0.1787	1.5663 ↑	0.6474	0.449	0.66956	0.90672 ↓	-0.141
TAG521-FA160	0.46455	0.96989	1.4578 ↑	0.5438	0.44957	0.97562	0.63855 ↓	-0.647	0.55539	0.99953	1.1396 ↑	0.1885	0.023343	0.14379	0.50355 ↓	-0.99	0.035039	0.15338	0.47728 ↓	-1.067
TAG525-FA183	0.46769	0.96989	0.3526 ↓	-1.504	0.9246	0.98297	0.71617 ↓	-0.482	0.76108	0.99953	0.85922 ↓	-0.219	0.23008	0.42715	0.80918 ↓	-0.305	0.006491	0.072593	1.9686 ↑	0.9772
TAG461-FA141	0.46948	0.96989	0.94595 ↓	-0.08	0.70857	0.98297	0.89747 ↓	-0.156	0.76755	0.99953	1.0594 ↑	0.0832	0.092896	0.27151	1.2162 ↑	0.2824	0.018762	0.10611	1.2731 ↑	0.3484
TAG501-FA160	0.46959	0.96989	1.422 ↑	0.5079	0.27478	0.95194	0.6511 ↓	-0.619	0.36044	0.99953	1.2657 ↑	0.3399	0.039182	0.16652	0.57935 ↓	-0.787	0.038368	0.1543	0.54216 ↓	-0.883
TAG460-FA180	0.47	0.96989	0.91873 ↓	-0.122	0.2027	0.95194	1.285 ↑	0.3617	0.98357	0.99953	1.0652 ↑	0.0911	0.12343	0.31225	1.2628 ↑	0.3367	0.009147	0.07792	1.4408 ↑	0.5269
TAG502-FA181	0.47043	0.96989	1.1546 ↑	0.2074	0.11016	0.95194	0.76067 ↓	-0.395	0.52181	0.99953	1.0599 ↑	0.0839	0.033249	0.15925	0.58347 ↓	-0.777	0.055786	0.19107	0.68232 ↓	-0.551
TAG481-FA120	0.47355	0.96989	1.1066 ↑	0.1461	0.98255	0.9902	1.0921 ↑	0.127	0.78845	0.99953	1.2258 ↑	0.2937	0.47226	0.66904	0.96841 ↓	-0.046	0.03967	0.15611	1.4734 ↑	0.5591
TAG511-FA181	0.47561	0.96989	1.1836 ↑	0.2432	0.20681	0.95194	0.72683 ↓	-0.46	0.70771	0.99953	1.0015 ↑	0.0022	0.26252	0.46488	0.90332 ↓	-0.147	0.003008	0.060885	0.66244 ↓	-0.594
TAG546-FA183	0.48137	0.96989	0.34101 ↓	-1.552	0.82479	0.98297	0.72519 ↓	-0.464	0.45176	0.99953	0.8307 ↓	-0.268	0.93652	0.97794	1.1447 ↑	0.195	0.000843	0.039819	1.7238 ↑	0.7856
TAG526-FA182	0.48386	0.96989	0.97382 ↓	-0.038	0.31343	0.95194	1.1666 ↑	0.2223	0.64744	0.99953	0.98984 ↓	-0.015	0.008042	0.1372	0.56871 ↓	-0.814	0.3037	0.5446	1.1333 ↑	0.1806
TAG501-FA161	0.48744	0.96989	1.1105 ↑	0.1512	0.079122	0.95194	0.67316 ↓	-0.571	0.67572	0.99953	1.0569 ↑	0.0798	0.11293	0.29266	0.66647 ↓	-0.585	0.021393	0.11106	0.71664 ↓	-0.481
TAG532-FA181	0.49082	0.96989	1.0547 ↑	0.0768	0.34596	0.95726	1.205 ↑	0.269	0.013493	0.62598	0.67663 ↓	-0.564	0.36854	0.59049	1.1639 ↑	0.2189	0.89262	0.96531	0.9904 ↓	-0.014
CE225	0.49699	0.96989	1.0677 ↑	0.0946	0.004958	0.95194	1.8514 ↑	0.8886	0.41959	0.99953	0.69326 ↓	-0.529	0.76868	0.86655	1.0751 ↑	0.1045	0.64708	0.82585	1.3156 ↑	0.3957
TAG521-FA181	0.50167	0.96989	1.4499 ↑	0.5359	0.68231	0.97562	0.75982 ↓	-0.396	0.53931	0.99953	1.1195 ↑	0.1628	0.038153	0.16379	0.57378 ↓	-0.801	0.036622	0.1543	0.54156 ↓	-0.885
HCER201	0.5048	0.96989	1.365 ↑	0.449	0.94342	0.98297	0.67544 ↓	-0.566	0.59919	0.99953	1.4789 ↑	0.5645	0.014677	0.1372	5.6808 ↑	2.5061	0.064062	0.20388	1.3246 ↑	0.4056

TAG471-FA181	0.50938	0.96989	0.96619 ↓	-0.05	0.9584	0.98357	0.99918 ↓	-0.001	0.66335	0.99953	0.89279 ↓	-0.164	0.012501	0.1372	1.3518 ↑	0.4348	0.068828	0.21352	1.1636 ↑	0.2186
TAG492-FA160	0.51786	0.96989	1.1182 ↑	0.1612	0.23918	0.95194	0.7833 ↓	-0.352	0.81274	0.99953	1.1369 ↑	0.1852	0.11115	0.2916	0.78205 ↓	-0.355	0.000423	0.029944	0.65523 ↓	-0.61
TAG542-FA201	0.51887	0.96989	1.3795 ↑	0.4641	0.38976	0.95901	0.66957 ↓	-0.579	0.65012	0.99953	0.78992 ↓	-0.34	0.003564	0.13256	0.32654 ↓	-1.615	0.006377	0.072593	0.44045 ↓	-1.183
TAG400-FA160	0.51918	0.96989	0.95212 ↓	-0.071	0.27562	0.95194	1.2376 ↑	0.3076	0.79999	0.99953	1.0008 ↑	0.0011	0.069627	0.233	1.4428 ↑	0.5289	0.002883	0.060885	1.5297 ↑	0.6133
TAG521-FA180	0.52404	0.96989	1.5417 ↑	0.6246	0.66959	0.97562	0.68043 ↓	-0.555	0.56582	0.99953	1.1222 ↑	0.1663	0.040762	0.17152	0.50322 ↓	-0.991	0.030758	0.13906	0.43404 ↓	-1.204
TAG502-FA161	0.5269	0.96989	1.1458 ↑	0.1963	0.050638	0.95194	0.61633 ↓	-0.698	0.72855	0.99953	1.0331 ↑	0.047	0.035902	0.15925	0.50041 ↓	-0.999	0.007733	0.076901	0.59452 ↓	-0.75
TAG504-FA182	0.5307	0.96989	0.85675 ↓	-0.223	0.19567	0.95194	0.64471 ↓	-0.633	0.34783	0.99953	1.1886 ↑	0.2492	0.015685	0.1372	0.39388 ↓	-1.344	0.005922	0.071904	0.54847 ↓	-0.867
TAG491-FA160	0.53326	0.96989	1.0936 ↑	0.1291	0.089664	0.95194	0.68383 ↓	-0.548	0.4145	0.99953	1.1255 ↑	0.1706	0.52043	0.69561	0.93456 ↓	-0.098	0.0049	0.071904	0.76891 ↓	-0.379
TAG555-FA182	0.53377	0.96989	0.68295 ↓	-0.55	0.62435	0.97562	1.0047 ↑	0.0068	0.13892	0.91055	0.84245 ↓	-0.247	0.56529	0.73247	1.1528 ↑	0.2051	0.035367	0.15338	1.206 ↑	0.2703
TAG500-FA140	0.53386	0.96989	0.93665 ↓	-0.094	0.14746	0.95194	1.2723 ↑	0.3474	0.254	0.99215	0.7513 ↓	-0.413	0.077816	0.25016	1.2583 ↑	0.3314	0.012979	0.093493	1.3358 ↑	0.4177
TAG524-FA180	0.54034	0.96989	1.0437 ↑	0.0618	0.92735	0.98297	1.1221 ↑	0.1662	0.7363	0.99953	0.77798 ↓	-0.362	0.033053	0.15925	0.61392 ↓	-0.704	0.057031	0.19224	0.60009 ↓	-0.737
TAG547-FA182	0.54143	0.96989	0.30021 ↓	-1.736	0.81094	0.98297	0.71377 ↓	-0.486	0.46028	0.99953	0.79751 ↓	-0.326	0.91183	0.96231	1.1476 ↑	0.1986	4.18E-05	0.005925	2.1612 ↑	1.1119
TAG420-FA140	0.54214	0.96989	0.95952 ↓	-0.06	0.38693	0.95901	1.1319 ↑	0.1787	0.80389	0.99953	0.92551 ↓	-0.112	0.016749	0.1372	1.4631 ↑	0.549	0.006873	0.073023	1.3512 ↑	0.4343
TAG563-FA182	0.54627	0.96989	0.87346 ↓	-0.195	0.82177	0.98297	0.69165 ↓	-0.532	0.79355	0.99953	1.0327 ↑	0.0464	0.003743	0.13256	0.27979 ↓	-1.838	0.13222	0.30876	0.47355 ↓	-1.078
TAG587-FA182	0.55039	0.96989	1.3847 ↑	0.4696	0.68875	0.97602	1.0409 ↑	0.0578	0.98999	0.99953	0.8169 ↓	-0.292	0.016371	0.1372	0.55827 ↓	-0.841	0.014901	0.10041	0.52181 ↓	-0.938
TAG546-FA182	0.5512	0.96989	0.31645 ↓	-1.66	0.91491	0.98297	0.67261 ↓	-0.572	0.96382	0.99953	0.92795 ↓	-0.108	0.74	0.85014	0.98807 ↓	-0.017	0.002152	0.052941	1.8118 ↑	0.8574
TAG563-FA201	0.55569	0.96989	1.2716 ↑	0.3466	0.13529	0.95194	0.57325 ↓	-0.803	0.44514	0.99953	0.74145 ↓	-0.432	0.003148	0.13256	0.31698 ↓	-1.658	0.026482	0.12789	0.49626 ↓	-1.011
N-ACETYLALANINE_neg_1	0.56368	0.96989	1.4634 ↑	0.5494	0.88553	0.98297	0.78377 ↓	-0.352	0.90954	0.99953	0.90229 ↓	-0.148	0.033175	0.15925	0.39094 ↓	-1.355	0.001572	0.047281	0.40158 ↓	-1.316
DAG180/181	0.56914	0.97245	0.89447 ↓	-0.161	0.50863	0.97562	1.1826 ↑	0.2419	0.048485	0.87596	0.72046 ↓	-0.473	0.75527	0.86287	1.0743 ↑	0.1034	0.31171	0.55199	1.1177 ↑	0.1606
TAG400-FA140	0.58719	0.97467	0.99221 ↓	-0.011	0.48188	0.97562	1.069 ↑	0.0963	0.59767	0.99953	0.90521 ↓	-0.144	0.013391	0.1372	1.6112 ↑	0.6882	0.00228	0.052941	1.4558 ↑	0.5418
TAG543-FA183	0.59232	0.97467	1.0581 ↑	0.0815	0.32787	0.95194	1.5662 ↑	0.6473	0.011421	0.62598	0.33658 ↓	-1.571	0.64415	0.78443	1.1776 ↑	0.2358	0.85639	0.95529	1.0135 ↑	0.0194
TAG470-FA150	0.60163	0.97467	0.91677 ↓	-0.125	0.65557	0.97562	1.0752 ↑	0.1046	0.59141	0.99953	0.89525 ↓	-0.16	0.025033	0.144	1.2961 ↑	0.3742	0.11854	0.28954	1.1396 ↑	0.1886
TAG522-FA181	0.6026	0.97467	1.3289 ↑	0.4102	0.5125	0.97562	0.64415 ↓	-0.635	0.62345	0.99953	1.008 ↑	0.0115	0.022253	0.14379	0.4631 ↓	-1.111	0.05997	0.19456	0.49715 ↓	-1.008
TAG503-FA140	0.60552	0.97467	1.1413 ↑	0.1907	0.20327	0.95194	0.67956 ↓	-0.557	0.54241	0.99953	0.97715 ↓	-0.033	0.021823	0.14379	0.37773 ↓	-1.405	0.074037	0.22304	0.49052 ↓	-1.028
TAG541-FA180	0.60894	0.97467	1.4856 ↑	0.5711	0.4643	0.97562	0.59053 ↓	-0.76	0.51147	0.99953	1.1391 ↑	0.1879	0.044114	0.17687	0.49142 ↓	-1.025	0.018407	0.10611	0.39938 ↓	-1.324
TAG484-FA182	0.61147	0.97467	1.0594 ↑	0.0833	0.039359	0.95194	0.6076 ↓	-0.719	0.10018	0.89093	1.604 ↑	0.6817	0.33152	0.55037	0.72576 ↓	-0.462	0.34311	0.58212	0.76494 ↓	-0.387
XANTHURENIC ACID_pos_2	0.61224	0.97467	0.45804 ↓	-1.126	0.15816	0.95194	0.75781 ↓	-0.4	0.88732	0.99953	1.2151 ↑	0.2811	0.022219	0.14379	0.67942 ↓	-0.558	0.78427	0.91353	0.72545 ↓	-0.463
TAG541-FA200	0.61728	0.97467	1.3357 ↑	0.4176	0.25317	0.95194	0.60463 ↓	-0.726	0.66306	0.99953	0.99761 ↓	-0.003	4.22E-06	0.001795	0.32611 ↓	-1.617	0.78456	0.91353	0.85739 ↓	-0.222
TAG589-FA181	0.61897	0.97467	1.2145 ↑	0.2804	0.63094	0.97562	1.1538 ↑	0.2064	0.6005	0.99953	0.76414 ↓	-0.388	0.093239	0.27151	0.64443 ↓	-0.634	2.14E-05	0.004552	0.40271 ↓	-1.312
TAG400-FA120	0.6192	0.97467	0.98153 ↓	-0.027	0.67681	0.97562	1.0061 ↑	0.0087	0.38709	0.99953	0.79459 ↓	-0.332	0.010021	0.1372	1.7265 ↑	0.7879	0.008994	0.07792	1.4171 ↑	0.503
TAG480-FA180	0.62637	0.97467	0.95227 ↓	-0.071	0.21903	0.95194	1.2192 ↑	0.286	0.99636	0.99953	0.99177 ↓	-0.012	0.18534	0.38685	1.1712 ↑	0.228	0.028681	0.13417	1.3523 ↑	0.4354
TAG531-FA180	0.63277	0.97467	1.1177 ↑	0.1606	0.35105	0.95726	1.2199 ↑	0.2867	0.1503	0.91055	0.74491 ↓	-0.425	0.95782	0.98357	1.0196 ↑	0.028	0.007962	0.076901	0.74348 ↓	-0.428
TAG492-FA182	0.64727	0.97467	1.1157 ↑	0.158	0.18983	0.95194	0.749 ↓	-0.417	0.19921	0.93892	1.4634 ↑	0.5493	0.17988	0.38685	0.75617 ↓	-0.403	0.019878	0.10611	0.58132 ↓	-0.783
TAG542-FA180	0.64872	0.97467	1.3577 ↑	0.4411	0.77422	0.98297	0.63613 ↓	-0.653	0.7918	0.99953	1.024 ↑	0.0342	0.02436	0.14379	0.40571 ↓	-1.302	0.052151	0.18683	0.41212 ↓	-1.279
TAG483-FA160	0.65498	0.97467	0.8093 ↓	-0.305	0.44363	0.97562	0.77316 ↓	-0.371	0.10131	0.89093	1.3406 ↑	0.4229	0.017988	0.1372	0.52734 ↓	-0.923	0.19936	0.41131	0.74686 ↓	-0.421
TAG542-FA181	0.65602	0.97467	1.3502 ↑	0.4332	0.44858	0.97562	0.57674 ↓	-0.794	0.64401	0.99953	0.96915 ↓	-0.045	0.023233	0.14379	0.42758 ↓	-1.226	0.083328	0.23821	0.50276 ↓	-0.992
SM241	0.65677	0.97467	0.9262 ↓	-0.111	0.19654	0.95194	1.5876 ↑	0.6668	0.60648	0.99953	0.89628 ↓	-0.158	0.045513	0.1787	1.8844 ↑	0.9141	0.45857	0.67408	1.201 ↑	0.2643
SM220	0.65817	0.97467	0.91833 ↓	-0.123	0.23645	0.95194	1.3197 ↑	0.4002	0.10474	0.89093	1.4402 ↑	0.5262	0.18595	0.38685	0.8848 ↓	-0.177	0.045895	0.16815	1.5165 ↑	0.6007
TAG545-FA183	0.66454	0.97467	0.44087 ↓	-1.182	0.83044	0.98297	0.71255 ↓	-0.489	0.86093	0.99953	0.93545 ↓	-0.096	0.50969	0.69355	0.8597 ↓	-0.218	0.029287	0.13417	1.4466 ↑	0.5326
TAG565-FA202	0.66462	0.97467	0.93639 ↓	-0.095	0.44845	0.97562	1.219 ↑	0.2858	0.033116	0.7334	0.61283 ↓	-0.706	0.84538	0.91645	1.1265 ↑	0.1719	0.46271	0.67408	1.3264 ↑	0.4075
TAG546-FA181	0.67089	0.97467	0.37913 ↓	-1.399	0.61504	0.97562	0.80997 ↓	-0.304	0.12779	0.91055	0.76907 ↓	-0.379	0.31349	0.52661	0.87099 ↓	-0.199	0.01264	0.092791	1.4373 ↑	0.5233
TAG563-FA181	0.67118	0.97467	1.0317 ↑	0.045	0.86624	0.98297	1.0306 ↑	0.0436	0.005613	0.59639	0.68527 ↓	-0.545	0.55845	0.73066	0.97941 ↓	-0.03	0.76766	0.90126	0.98935 ↓	-0.015
TAG543-FA202	0.67617	0.97467	0.94295 ↓	-0.085	0.32346	0.95194	1.3068 ↑	0.386	0.016823	0.62598	0.55495 ↓	-0.85	0.48139	0.67299	1.2084 ↑	0.2731	0.52053	0.71418	1.3031 ↑	0.3819
TAG522-FA180	0.69741	0.97467	1.2879 ↑	0.3651	0.6832	0.97562	0.56804 ↓	-0.816	0.67277	0.99953	1.1961 ↑	0.2584	0.019138	0.1372	0.27893 ↓	-1.842	0.016388	0.10149	0.30475 ↓	-1.714
TAG542-FA182	0.6977	0.97467	1.0697 ↑	0.0972	0.86114	0.98297	0.99112 ↓	-0.013	0.50265	0.99953	0.89859 ↓	-0.154	0.073154	0.23916	0.62969 ↓	-0.667	0.023183	0.11592	0.5336 ↓	-0.906
TAG561-FA160	0.69947	0.97467	1.2556 ↑	0.3284	0.13103	0.95194	0.57286 ↓	-0.804	0.77373	0.99953	1.188 ↑	0.2485	0.01802	0.1372	0.33416 ↓	-1.581	0.37988	0.60529	0.59666 ↓	-0.745
TAG503-FA161	0.70386	0.97527	1.1079 ↑	0.1478	0.057158	0.95194	0.61122 ↓	-0.71	0.81856	0.99953	1.0667 ↑	0.0931	0.025412	0.144	0.40204 ↓	-1.315	0.004343	0.071904	0.52141 ↓	-0.94
TAG421-FA120	0.70601	0.97527	1.0001 ↑	0.0001	0.97182	0.98479	0.87367 ↓	-0.195	0.48038	0.99953	0.79634 ↓	-0.329	0.009339	0.1372	1.866 ↑	0.8999	0.024865	0.12274	1.3127 ↑	0.3925
TAG543-FA181	0.71134	0.97527	1.0189 ↑	0.0269	0.48392	0.97562	0.64624 ↓	-0.63	0.7825	0.99953	0.9367 ↓	-0.094	0.014733	0.1372	0.4579 ↓	-1.127	0.29524	0.53395	0.83384 ↓	-0.262
TAG525-FA160	0.71504	0.97527	0.3757 ↓	-1.412	0.99684	0.99684	0.72084 ↓	-0.472	0.6381	0.99953	0.86266 ↓	-0.213	0.18903	0.38776	0.68113 ↓	-0.554	0.01512	0.10041	1.7126 ↑	0.7762
TAG420-FA120	0.71596	0.97527	0.99131 ↓	-0.013	0.59818	0.97562	1.0379 ↑	0.0537	0.4555	0.99953	0.83472 ↓	-0.261	0.007922	0.1372	1.6052 ↑	0.6827	0.005524	0.071904	1.3689 ↑	0.453
DAG140/182	0.72704	0.9811	0.90207 ↓	-0.149	0.932	0.98297	1.1647 ↑	0.22	0.015706	0.62598	2.241 ↑	1.1642	0.093675	0.27151	1.5134 ↑	0.5978	0.47395	0.68285	0.81305 ↓	-0.299
GLUCOSE 1-PHOSPHATE_pos_2	0.73725	0.98176	0.70142 ↓	-0.512	0.96028	0.98357	0.91356 ↓	-0.13	0.037964	0.7334	3.1204 ↑	1.6417	0.84733	0.91645	1.1878 ↑	0.2483	0.85107	0.95437	1.14 ↑	0.1891
TAG460-FA120	0.73929	0.98176	0.99923 ↓	-0.001	0.21481	0.95194	1.3506 ↑	0.4336	0.84657	0.99953	0.97876 ↓	-0.031	0.3094	0.52388	1.2233 ↑	0.2908	0.005674	0.071904	1.4899 ↑	0.5752
TAG492-FA150	0.74116	0.98176	1.1376 ↑	0.1859	0.066807	0.95194	0.5792 ↓	-0.788	0.90094	0.99953	1.0003 ↑	0.0005	0.10624	0.29083	0.68914 ↓	-0.537	0.001013	0.04305	0.57997 ↓	-0.786
CER160	0.74381	0.98176	1.316 ↑	0.3962	0.25686	0.95194	0.4295 ↓	-1.219	0.85623	0.99953	1.2951 ↑	0.373	0.036469	0.15979	0.29201 ↓	-1.776	1.23E-05	0.004552	0.10801 ↓	-3.211
TAG502-FA160	0.74383	0.98176	1.1236 ↑	0.1681	0.29505	0.95194	0.53188 ↓	-0.911	0.44621	0.99953	1.2878 ↑	0.3649	0.021258	0.14379	0.36387 ↓	-1.459	0.045245	0.16721	0.42994 ↓	-1.218
CER240	0.75147	0.98311	1.5293 ↑	0.6129	0.32225	0.95194	0.33505 ↓	-1.578	0.96958	0.99953	1.0885 ↑	0.1223	0.067871	0.22893	0.21553 ↓	-2.214	0.000242	0.020551	0.04419 ↓	-4.5
TAG421-FA161	0.75902	0.98311	0.92976 ↓	-0.105	0.62674	0.97562	0.95038 ↓	-0.073	0.57642	0.99953	1.168 ↑	0.2241	0.6999	0.82232	1.1862 ↑	0.2463	0.018306	0.10611	1.2924 ↑	0.37
TAG522-FA160	0.76671	0.98444	1.2493 ↑	0.3212	0.58558	0.97562	0.61674 ↓	-0.697	0.57907	0.99953	1.1181 ↑	0.1611	0.023339	0.14379	0.43882 ↓	-1.188	0.056196	0.19107	0.45741 ↓	-1.128
TAG483-FA140	0.77438	0.98701	0.86432 ↓	-0.21	0.019739	0.95194	0.45999 ↓	-1.12	0.16228	0.93126	1.4855 ↑	0.571	0.031021	0.15925	0.4339 ↓	-1.205	0.16923	0.36884	0.59031 ↓	-0.76
TAG564-FA202	0.77568	0.98701	0.95426 ↓	-0.068	0.3498	0.95726	1.3034 ↑	0.3823	0.017675	0.62598	0.58018 ↓	-0.785	0.57005	0.73416	1.1699 ↑	0.2264	0.56302	0.74776	1.289 ↑	0.3663
TAG503-FA182	0.78445	0.98701	1.2023 ↑	0.2658	0.055904	0.95194	0.56417 ↓	-0.826	0.70786	0.99953	1.0801 ↑	0.1111	0.016889	0.1372	0.37834 ↓	-1.402	0.00577	0.071904	0.41085 ↓	-1.283
PROPIONYL-COA_pos_1	0.79003	0.98701	1.075 ↑	0.1043	0.91302	0.98297	0.74543 ↓	-0.424	0.2095	0.93892	4.4807 ↑	2.1637	0.045155	0.1787	2.4206 ↑	1.2754	0.3519	0.5865	0.4675 ↓	-1.097
TAG503-FA160	0.80181	0.98701	1.1822 ↑	0.2415	0.30016	0.95194	0.52916 ↓	-0.918	0.79283	0.99953	1.1182 ↑	0.1611	0.02377	0.14379	0.2525 ↓	-1.986	0.016103	0.10149	0.32531 ↓	-1.62
TAG563-FA202	0.80345	0.98701	0.94868 ↓	-0.076	0.36269	0.95901	1.3006 ↑	0.3792	0.022246	0.67533	0.54046 ↓	-0.888	0.40619	0.62418	1.2198 ↑	0.2866	0.38674	0.6133	1.3578 ↑	0.4413
TAG543-FA160	0.80573	0.98701	1.2902 ↑	0.3676	0.41551	0.97562	0.67717 ↓	-0.562	0.80352	0.99953	1.1059 ↑	0.1452	0.001537	0.13256	0.27366 ↓	-1.87	0.015846	0.10149	0.34959 ↓	-1.516
TAG533-FA182	0.80701	0.98701	1.0157 ↑	0.0224	0.74472	0.98297	0.69507 ↓	-0.525	0.77732	0.99953	1.1815 ↑	0.2407	0.010579	0.1372	0.57543 ↓	-0.797	0.044264	0.16648	0.50667 ↓	-0.981
TAG522-FA182	0.80706	0.98701	1.0971 ↑	0.1337	0.68099	0.97562	0.61871 ↓	-0.693	0.52887	0.99953	1.2384 ↑	0.3085	0.022854	0.14379	0.33936 ↓	-1.559	0.043456	0.16498	0.38528 ↓	-1.376
TAG542-FA160	0.81417	0.98701	1.2557 ↑	0.3285	0.42284	0.97562	0.59154 ↓	-0.757	0.59139	0.99953	0.99887 ↓	-0.002	0.007427	0.1372	0.30493 ↓	-1.713	0.038847	0.1543	0.42434 ↓	-1.237
TAG503-FA141	0.8192	0.98701	0.91488 ↓	-0.128	0.32926	0.95194	0.83487 ↓	-0.26	0.067997	0.87596	1.3725 ↑	0.4569	0.035591	0.15925	0.51578 ↓	-0.955	0.20802	0.42301	0.7423 ↓	-0.43
TAG583-FA181	0.82164	0.98701	1.0199 ↑	0.0284	0.47303	0.97562	0.66395 ↓	-0.591	0.25752	0.99215	0.78158 ↓	-0.356	0.010761	0.1372	0.21859 ↓	-2.194	0.05584	0.19107	0.47132 ↓	-1.085
TAG526-FA160	0.82773	0.98701	0.63342 ↓	-0.659	0.28034	0.95194	1.2527 ↑	0.325	0.89039	0.99953	0.74389 ↓	-0.427	0.13784	0.33188	0.74023 ↓	-0.434	0.001467	0.047281	1.9112 ↑	0.9345
TAG523-FA181	0.83204	0.98701	0.9539 ↓	-0.068	0.65486	0.97562	0.56283 ↓	-0.829	0.77816	0.99953	1.0172 ↑	0.0245	0.023473	0.14379	0.30636 ↓	-1.707	0.044942	0.16721	0.38495 ↓	-1.377
TAG523-FA182	0.83213	0.98701	0.87209 ↓	-0.197	0.64649	0.97562	0.53459 ↓	-0.904	0.64207	0.99953	1.1072 ↑	0.147	0.033653	0.15925	0.32496 ↓	-1.622	0.10172	0.26361	0.41553 ↓	-1.267
TAG493-FA182	0.83518	0.98701	1.0164 ↑	0.0234	0.13344	0.95194	0.75793 ↓	-0.4	0.49985	0.99953	1.1215 ↑	0.1654	0.79165	0.88308	0.90439 ↓	-0.145	0.021591	0.11106	0.77148 ↓	-0.374
TAG512-FA182	0.83605	0.98701	1.1468 ↑	0.1976	0.62385	0.97562	0.72774 ↓	-0.459	0.37441	0.99953	1.5255 ↑	0.6093	0.088387	0.2694	0.59514 ↓	-0.749	0.014782	0.10041	0.39646 ↓	-1.335
TAG512-FA170	0.83852	0.98717	1.1236 ↑	0.1681	0.080072	0.95194	0.59919 ↓	-0.739	0.37152	0.99953	1.1891 ↑	0.2499	0.64134	0.78443	0.85761 ↓	-0.222	0.001669	0.047281	0.5679 ↓	-0.816
CER220	0.84194	0.98847	1.2994 ↑	0.3779	0.64705	0.97562	0.7512 ↓	-0.413	0.3019	0.99215	0.92977 ↓	-0.105	0.019369	0.1372	0.42968 ↓	-1.219	9.1E-05	0.009668	0.10827 ↓	-3.207
TAG504-FA161	0.84985	0.99228	1.0152 ↑	0.0218	0.18385	0.95194	0.6923 ↓	-0.531	0.33071	0.99215	0.83279 ↓	-0.264	0.043643	0.17665	0.32934 ↓	-1.602	0.66195	0.83729	0.61469 ↓	-0.702
SM180	0.87399	0.99469	1.0233 ↑	0.0332	0.023924	0.95194	1.6296 ↑	0.7045	0.89088	0.99953	0.82253 ↓	-0.282	0.025164	0.144	0.60628 ↓	-0.722	0.30013	0.54048	1.1846 ↑	0.2444
LCER160	0.88295	0.99469	1.0218 ↑	0.0311	0.23369	0.95194	1.3161 ↑	0.3962	0.1072	0.89093	0.62274 ↓	-0.683	0.033642	0.15925	2.2117 ↑	1.1451	0.36585	0.59866	1.3544 ↑	0.4377
TAG523-FA160	0.88471	0.99469	0.86445 ↓	-0.21	0.80913	0.98297	0.56449 ↓	-0.825	0.69053	0.99953	1.1088 ↑	0.149	0.037817	0.16379	0.34892 ↓	-1.519	0.091824	0.25143	0.43815 ↓	-1.191
TAG546-FA226	0.8876	0.99469	0.93903 ↓	-0.091	0.4916	0.97562	0.90165 ↓	-0.149	0.024607	0.69719	0.6386 ↓	-0.647	0.99195	0.99682	1.0077 ↑	0.0111	0.83753	0.94416	0.88529 ↓	-0.176
TAG524-FA182	0.8918	0.99469	0.48828 ↓	-1.034	0.89026	0.98297	0.6241 ↓	-0.68	0.62028	0.99953	1.1464 ↑	0.1971	0.046664	0.17935	0.37613 ↓	-1.411	0.5549	0.73928	0.72595 ↓	-0.462
TAG525-FA205	0.89726	0.99469	1.0039 ↑	0.0056	0.12634	0.95194	1.997 ↑	0.9978	0.84247	0.99953	0.96286 ↓	-0.055	0.060172	0.20962	1.9126 ↑	0.9356	0.009611	0.07792	1.4543 ↑	0.5403
TAG513-FA182	0.9074	0.99469	1.1682 ↑	0.2243	0.32401	0.95194	0.60306 ↓	-0.73	0.64151	0.99953	1.2172 ↑	0.2836	0.017078	0.1372	0.46276 ↓	-1.112	0.001614	0.047281	0.3535 ↓	-1.5
TAG543-FA161	0.91172	0.99469	1.1858 ↑	0.2458	0.93499	0.98297	0.88933 ↓	-0.169	0.73903	0.99953	0.89332 ↓	-0.163	0.006736	0.1372	0.32284 ↓	-1.631	0.053489	0.18855	0.51376 ↓	-0.961
TAG543-FA182	0.9125	0.99469	0.93083 ↓	-0.103	0.60406	0.97562	0.52046 ↓	-0.942	0.71836	0.99953	1.0775 ↑	0.1077	0.029185	0.15701	0.27835 ↓	-1.845	0.084073	0.23821	0.37828 ↓	-1.403
CE205	0.92289	0.99469	1.2488 ↑	0.3205	0.045167	0.95194	1.9754 ↑	0.9822	0.29736	0.99215	1.2455 ↑	0.3167	0.99135	0.99682	1.0192 ↑	0.0274	0.88816	0.96531	0.91421 ↓	-0.129
TAG451-FA160	0.92735	0.99469	1.0108 ↑	0.0156	0.74164	0.98297	1.0392 ↑	0.0555	0.48361	0.99953	0.83871 ↓	-0.254	0.01826	0.1372	1.4222 ↑	0.5081	0.10963	0.27363	1.1747 ↑	0.2322
TAG543-FA180	0.9286	0.99469	1.0127 ↑	0.0182	0.99086	0.9932	0.68552 ↓	-0.545	0.9861	0.99953	1.0358 ↑	0.0508	0.018655	0.1372	0.32945 ↓	-1.602	0.031458	0.14072	0.41069 ↓	-1.284
TAG502-FA180	0.93845	0.99469	1.0704 ↑	0.0981	0.17335	0.95194	0.81573 ↓	-0.294	0.31674	0.99215	1.1646 ↑	0.2198	0.004223	0.13316	0.47835 ↓	-1.064	0.005586	0.071904	0.53156 ↓	-0.912
TAG544-FA182	0.94397	0.99469	0.59108 ↓	-0.759	0.80875	0.98297	0.60268 ↓	-0.731	0.71911	0.99953	1.0251 ↑	0.0358	0.030629	0.15925	0.34094 ↓	-1.552	0.39261	0.61572	0.62934 ↓	-0.668
TAG524-FA181	0.94434	0.99469	0.94978 ↓	-0.074	0.72747	0.98297	0.6282 ↓	-0.671	0.74502	0.99953	0.87401 ↓	-0.194	0.015282	0.1372	0.25757 ↓	-1.957	0.031786	0.14072	0.38202 ↓	-1.388
TAG524-FA161	0.95011	0.99469	1.3685 ↑	0.4526	0.25939	0.95194	0.42212 ↓	-1.244	0.82245	0.99953	0.92 ↓	-0.12	0.01355	0.1372	0.15274 ↓	-2.711	0.006858	0.073023	0.19376 ↓	-2.368
TAG514-FA182	0.95597	0.99469	1.0259 ↑	0.0369	0.90592	0.98297	0.84197 ↓	-0.248	0.98367	0.99953	1.2004 ↑	0.2635	0.098668	0.27956	0.57163 ↓	-0.807	0.000597	0.031712	0.4855 ↓	-1.043
TAG545-FA160	0.95767	0.99469	1.735 ↑	0.7949	0.926	0.98297	0.72333 ↓	-0.467	0.95884	0.99953	1.0819 ↑	0.1136	0.011245	0.1372	0.18945 ↓	-2.4	0.012663	0.092791	0.16593 ↓	-2.591
TAG544-FA180	0.96839	0.99469	0.65454 ↓	-0.611	0.80092	0.98297	0.82942 ↓	-0.27	0.74411	0.99953	1.0515 ↑	0.0724	0.031153	0.15925	0.42711 ↓	-1.227	0.26859	0.50734	0.73158 ↓	-0.451
TAG544-FA181	0.97112	0.99469	0.67723 ↓	-0.562	0.97241	0.98479	0.64302 ↓	-0.637	0.9455	0.99953	0.89201 ↓	-0.165	0.027025	0.14917	0.36238 ↓	-1.464	0.19066	0.39917	0.57495 ↓	-0.798
TAG472-FA182	0.97127	0.99469	0.9519 ↓	-0.071	0.61269	0.97562	0.8702 ↓	-0.201	0.90235	0.99953	0.9734 ↓	-0.039	0.027018	0.14917	1.2735 ↑	0.3488	0.54256	0.73299	1.0383 ↑	0.0542
TAG525-FA182	0.98058	0.99469	0.57754 ↓	-0.792	0.89777	0.98297	0.63063 ↓	-0.665	0.99553	0.99953	1.027 ↑	0.0384	0.011305	0.1372	0.27039 ↓	-1.887	0.3611	0.59715	0.62122 ↓	-0.687
TAG565-FA225	0.98526	0.99469	0.8428 ↓	-0.247	0.089355	0.95194	1.2959 ↑	0.3739	0.029339	0.7334	0.64705 ↓	-0.628	0.26744	0.47163	1.1533 ↑	0.2057	0.10472	0.26812	1.2412 ↑	0.3118
TAG503-FA181	0.98542	0.99469	1.0263 ↑	0.0375	0.086754	0.95194	0.80124 ↓	-0.32	0.85804	0.99953	0.93942 ↓	-0.09	0.014545	0.1372	0.55918 ↓	-0.839	0.021689	0.11106	0.71589 ↓	-0.482
TAG564-FA182	0.99556	0.99716	0.81342 ↓	-0.298	0.33544	0.95726	0.50566 ↓	-0.984	0.93308	0.99953	0.89597 ↓	-0.158	0.00284	0.13256	0.15131 ↓	-2.724	0.19425	0.40469	0.44758 ↓	-1.16

Supplementary Table S3B. List of dysregulated metabolites for male mice following exposure to 9.5 Gy of Gamma-radiation, 24 h, 1 week, 1 month, 3 month and 6 months post-irradiation.

	9.5Gy / 0.0Gy				9.5Gy / 0.0Gy				9.5Gy / 0.0Gy				9.5Gy / 0.0Gy				9.5Gy / 0.0Gy			
	24h				1wk				1mo				3mo				6mo			
									Male											
name	p-value	FDR	FC	LOG2FC	p-value	FDR	FC	LOG2FC	p-value	FDR	FC	LOG2FC	p-value	FDR	FC	LOG2FC	p-value	FDR	FC	LOG2FC
CYSTEINE_neg_5	0.005664	0.2407	2.4211	1.2756	0.91515	0.99	1.144	0.1941	0.86697	0.95387	1.5675	0.6485	0.000531	0.22564	6.3248	2.661	0.85351	0.96019	1.6482	0.7209
N-ACETYLGLUTAMINE_pos_2	0.18447	0.72412	2.3125	1.2095	0.19463	0.89138	0.83612	-0.258	0.28843	0.67701	0.95505	-0.066	0.51279	0.99441	1.3436	0.4261	0.005404	0.31319	0.33434	-1.581
INDOLE-3-CARBOXYLIC ACID_neg_1	0.75207	0.9716	0.90106	-0.15	0.83391	0.97366	0.98555	-0.021	0.046714	0.42979	1.7823	0.8338	0.47803	0.99441	1.1376	0.186	0.32968	0.75069	1.4227	0.5086
CE204	0.043996	0.58433	1.391	0.4761	0.038224	0.52469	1.5625	0.6439	0.67999	0.90614	0.12577	-2.991	0.70336	0.99441	0.97961	-0.03	0.084451	0.54839	1.4214	0.5073
OXOGLUTARATE_neg_2	0.46392	0.85354	0.52654	-0.925	0.45557	0.89138	1.9469	0.9612	0.024106	0.345	0.67972	-0.557	0.42619	0.99441	0.76006	-0.396	0.021151	0.52879	0.36105	-1.47
NORMETANEPHRINE_pos_1	0.008954	0.31711	2.5052	1.3249	0.1644	0.87306	2.2393	1.1631	0.072039	0.46976	2.1062	1.0747	0.19608	0.99441	3.2913	1.7187	0.77737	0.95228	0.97435	-0.037
ASCORBATE_neg_2	0.15783	0.72412	1.0162	0.0232	0.59089	0.90011	1.3082	0.3876	0.017433	0.324	0.42884	-1.222	0.66707	0.99441	0.96939	-0.045	0.46266	0.8197	0.84056	-0.251
N-GLYCYL-L-PROLINE_neg_3	0.39626	0.82612	0.35371	-1.499	0.83261	0.97366	1.2194	0.2862	0.75347	0.9336	1.3441	0.4267	0.8856	0.99441	1.1255	0.1706	0.048501	0.54839	2.2647	1.1793
AMINOADIPATE_pos_1	0.022137	0.53437	2.2338	1.1595	0.72449	0.9445	1.2051	0.2691	0.090424	0.49525	0.6102	-0.713	0.39441	0.99441	1.3462	0.4289	0.02556	0.54839	0.66344	-0.592
CE226	0.004853	0.2407	1.8434	0.8824	0.09432	0.76624	1.5977	0.676	0.4235	0.78215	0.33145	-1.593	0.024579	0.99441	1.7456	0.8038	0.14148	0.58674	1.3325	0.4142
ACETYLPHOSPHATE_pos_1	0.80127	0.9716	0.96267	-0.055	0.48448	0.89138	0.84306	-0.246	0.091013	0.49525	0.7124	-0.489	0.61462	0.99441	1.157	0.2104	0.005155	0.31319	0.56029	-0.836
ATROLACTIC ACID_neg_2	0.99955	0.99955	0.84269	-0.247	0.72382	0.9445	1.0156	0.0224	0.005527	0.24745	2.1122	1.0788	0.29773	0.99441	1.5064	0.5912	0.16212	0.60235	2.4597	1.2985
TAG582-FA181	0.09492	0.72412	0.71668	-0.481	0.11025	0.76624	1.339	0.4212	0.42251	0.78215	0.92405	-0.114	0.53628	0.99441	1.0416	0.0587	0.034434	0.54839	1.5972	0.6756
TAG483-FA182	0.25655	0.77754	0.92598	-0.111	0.38783	0.89138	0.88273	-0.18	0.43582	0.78215	0.77479	-0.368	0.84085	0.99441	1.005	0.0072	0.026097	0.54839	0.85848	-0.22
OROTATE_neg_1	0.061379	0.67761	1.7334	0.7936	0.022658	0.38519	0.73303	-0.448	0.9845	0.99242	0.89922	-0.153	0.24315	0.99441	0.66598	-0.586	0.80426	0.95228	1.3936	0.4788
DCER260	0.040208	0.55896	0.6432	-0.637	0.27259	0.89138	1.1914	0.2526	0.41869	0.78215	0.91296	-0.131	0.70868	0.99441	1.2663	0.3406	0.54259	0.85329	1.0948	0.1307
CE180	0.894	0.9716	1.0521	0.0733	0.013503	0.37637	1.3897	0.4747	0.36579	0.74028	1.0266	0.0378	0.47895	0.99441	1.0303	0.043	0.51724	0.85329	1.014	0.0201
TAG442-FA182	0.11927	0.72412	0.76512	-0.386	0.54073	0.89728	1.0797	0.1106	0.051775	0.43146	1.5234	0.6073	0.81652	0.99441	0.96922	-0.045	0.035444	0.54839	0.79976	-0.322
L-ACETYLCARNITINE_pos_1	0.3282	0.7915	1.2559	0.3287	0.005753	0.27165	0.41131	-1.282	0.33513	0.72506	0.69525	-0.524	0.19475	0.99441	2.1224	1.0857	0.49087	0.84804	2.1329	1.0928
ADP_pos_1	0.075926	0.67761	1.4224	0.5083	0.00156	0.13259	2.0516	1.0367	0.000562	0.1195	1.9971	0.9979	0.0319	0.99441	2.3289	1.2197	0.2891	0.70059	1.5376	0.6207
3-METHYLAMINO-L-ALANINE_pos_2	0.038541	0.55896	2.5148	1.3305	0.58724	0.89995	1.228	0.2963	0.44136	0.78215	0.83707	-0.257	0.62894	0.99441	0.93134	-0.103	0.41639	0.81279	1.4284	0.5144
TAG540-FA180	0.1292	0.72412	0.82766	-0.273	0.79674	0.97366	1.0504	0.071	0.03379	0.39891	1.2946	0.3725	0.76538	0.99441	0.99322	-0.01	0.80566	0.95228	0.99175	-0.012
TAG421-FA160	0.76067	0.9716	1.023	0.0328	0.37964	0.89138	1.1109	0.1517	0.042192	0.42979	1.6608	0.7319	0.66963	0.99441	0.906	-0.142	0.012815	0.39402	0.78174	-0.355
TAG442-FA181	0.92254	0.9716	1.0032	0.0046	0.65892	0.93511	1.1219	0.166	0.033603	0.39891	1.8674	0.901	0.72497	0.99441	1.012	0.0173	0.39437	0.79813	0.82693	-0.274
TAG442-FA160	0.92132	0.9716	1.1266	0.172	0.82749	0.97366	0.98908	-0.016	0.045606	0.42979	1.2323	0.3014	0.26061	0.99441	0.87195	-0.198	0.13597	0.58674	0.87814	-0.187
TAG567-FA226	0.001595	0.14065	2.6904	1.4278	0.27794	0.89138	1.1056	0.1448	0.8755	0.95387	0.60537	-0.724	0.82081	0.99441	0.99042	-0.014	0.078084	0.54839	1.4356	0.5217
CE160	0.63131	0.95315	0.92339	-0.115	0.032234	0.50675	1.2872	0.3643	0.67856	0.90614	0.60487	-0.725	0.81699	0.99441	0.97673	-0.034	0.005839	0.31319	0.84232	-0.248
TAG480-FA140	0.89013	0.9716	1.0493	0.0695	0.89919	0.99	1.0091	0.0131	0.21087	0.64834	1.1161	0.1585	0.29476	0.99441	0.92045	-0.12	0.045149	0.54839	0.8921	-0.165
TAG504-FA181	0.22909	0.76067	0.82304	-0.281	0.007712	0.27531	1.402	0.4875	0.94612	0.97597	0.95349	-0.069	0.42151	0.99441	1.112	0.1531	0.54977	0.85329	1.1047	0.1437
FFA141	0.65333	0.96411	1.2049	0.2689	0.10912	0.76624	1.373	0.4573	0.045581	0.42979	1.397	0.4824	0.25313	0.99441	1.2098	0.2747	0.29978	0.7067	1.3785	0.4631
TAG441-FA160	0.26128	0.77754	0.94569	-0.081	0.74574	0.95402	1.0427	0.0603	0.1164	0.52084	1.2605	0.334	0.44745	0.99441	0.92254	-0.116	0.002078	0.31319	0.81512	-0.295
TAG471-FA170	0.35068	0.80541	0.89081	-0.167	0.65387	0.93511	0.89542	-0.159	0.48547	0.8154	0.72565	-0.463	0.38712	0.99441	1.0681	0.0951	0.011541	0.39402	0.82042	-0.286
RIBOFLAVIN_pos_1	0.74036	0.9716	0.81426	-0.296	0.016302	0.37637	1.6943	0.7607	0.39045	0.77005	1.4534	0.5395	0.33534	0.99441	1.4942	0.5793	0.91056	0.96019	0.78515	-0.349
TAG451-FA181	0.034561	0.54402	0.83117	-0.267	0.69702	0.94138	1.0392	0.0555	0.49561	0.8154	1.0094	0.0135	0.52832	0.99441	1.0319	0.0453	0.033786	0.54839	0.89987	-0.152
METHYL PHENYLACETATE_neg_3	0.000218	0.09259	0.21171	-2.24	0.006446	0.27393	0.43679	-1.195	0.05918	0.4396	1.8752	0.9071	0.6543	0.99441	1.4084	0.4941	0.25058	0.67154	0.9213	-0.118
TAG501-FA181	0.001203	0.14065	1.274	0.3493	0.51747	0.89138	0.93172	-0.102	0.98064	0.99242	0.85937	-0.219	0.98641	0.99759	0.99057	-0.014	0.076911	0.54839	0.83543	-0.259
FFA205	0.48398	0.86425	1.3537	0.4369	0.11901	0.76624	1.3406	0.4229	0.016214	0.324	1.4397	0.5258	0.41245	0.99441	1.2073	0.2718	0.54606	0.85329	1.2589	0.3322
TAG510-FA160	0.45955	0.85287	1.0567	0.0796	0.91659	0.99	0.99406	-0.009	0.91096	0.96399	0.98263	-0.025	0.76015	0.99441	0.97908	-0.031	0.044755	0.54839	0.82472	-0.278
4-IMIDAZOLEACETATE_pos_1	0.94324	0.97541	1.051	0.0718	0.20239	0.89138	0.82822	-0.272	0.018296	0.324	0.75054	-0.414	0.98962	0.99759	0.85868	-0.22	0.51986	0.85329	1.6402	0.7139
SM261	0.56253	0.8992	0.7721	-0.373	0.017277	0.37637	1.5719	0.6525	0.46881	0.80341	1.0826	0.1146	0.83522	0.99441	1.1034	0.1419	0.25282	0.67154	0.96597	-0.05
4-AMINOBENZOIC ACID_pos_1	0.72532	0.9716	0.60763	-0.719	0.10889	0.76624	0.70325	-0.508	0.018202	0.324	0.60309	-0.73	0.62694	0.99441	0.68557	-0.545	0.45919	0.8197	1.1751	0.2328
DAG181/226	0.57903	0.91144	1.071	0.0989	0.16639	0.87306	1.14	0.189	0.039491	0.42979	1.2667	0.3411	0.87147	0.99441	1.0055	0.008	0.16299	0.60235	1.1633	0.2182
TAG482-FA181	0.26105	0.77754	0.92944	-0.106	0.61225	0.913	0.94789	-0.077	0.7861	0.93401	0.86056	-0.217	0.53281	0.99441	0.9548	-0.067	0.044499	0.54839	0.87187	-0.198
TAG470-FA140	0.73711	0.9716	0.98523	-0.021	0.46907	0.89138	0.95535	-0.066	0.64158	0.89695	0.95092	-0.073	0.46616	0.99441	0.95868	-0.061	0.037009	0.54839	0.88998	-0.168
TAG544-FA160	0.019393	0.53356	1.7038	0.7688	0.84941	0.98587	0.86312	-0.212	0.21339	0.64834	1.0783	0.1087	0.13439	0.99441	1.4417	0.5278	0.050756	0.54839	1.5189	0.603
DAG160/182	0.35196	0.80541	1.0885	0.1224	0.4569	0.89138	1.2243	0.292	0.6817	0.90614	0.97696	-0.034	0.74027	0.99441	1.0285	0.0406	0.046958	0.54839	1.2631	0.337
TAG542-FA202	0.97219	0.9935	2.2058	1.1413	0.033386	0.50675	1.3354	0.4173	0.014935	0.31736	1.3184	0.3988	0.19785	0.99441	0.85701	-0.223	0.58884	0.86812	1.8794	0.9103
TAG523-FA161	0.89851	0.9716	1.0537	0.0754	0.04322	0.55662	0.80671	-0.31	0.14653	0.57134	0.51728	-0.951	0.66907	0.99441	1.0321	0.0456	0.55235	0.85329	0.96589	-0.05
TAG589-FA226	0.72768	0.9716	1.0819	0.1136	0.18886	0.89138	1.3376	0.4196	0.1042	0.52084	0.57014	-0.811	0.03192	0.99441	1.3816	0.4663	0.30215	0.7067	1.3447	0.4273
TAG420-FA160	0.2205	0.74915	0.90841	-0.139	0.41979	0.89138	1.0472	0.0665	0.021011	0.33072	1.4086	0.4942	0.7262	0.99441	0.92015	-0.12	0.90763	0.96019	1.0917	0.1266
TAG481-FA141	0.68385	0.9716	1.1209	0.1646	0.018102	0.37637	0.64849	-0.625	0.60427	0.86553	0.89095	-0.167	0.60595	0.99441	0.9099	-0.136	0.71673	0.92697	0.97757	-0.033
TAG440-FA180	0.45272	0.85287	0.93353	-0.099	0.15352	0.85739	1.1263	0.1716	0.007569	0.24745	1.5478	0.6302	0.62002	0.99441	0.89939	-0.153	0.15343	0.60235	0.87794	-0.188
TAG501-FA180	0.93539	0.97541	1.0108	0.0155	0.76831	0.96894	1.0159	0.0227	0.56223	0.84263	1.1631	0.2179	0.5712	0.99441	0.9254	-0.112	0.011116	0.39402	0.82748	-0.273
TAG491-FA181	0.89056	0.9716	1.0055	0.0079	0.38158	0.89138	0.88947	-0.169	0.49732	0.8154	0.8104	-0.303	0.69739	0.99441	0.96127	-0.057	0.03644	0.54839	0.85745	-0.222
TAG541-FA181	0.32302	0.7915	0.9323	-0.101	0.058366	0.63604	1.1887	0.2494	0.049203	0.43146	1.1957	0.2578	0.99068	0.99759	1.0178	0.0255	0.49831	0.85054	1.0882	0.1219
TAG421-FA181	0.48047	0.8616	0.89717	-0.157	0.72393	0.9445	1.0737	0.1026	0.014703	0.31736	1.951	0.9643	0.59373	0.99441	0.89657	-0.158	0.80888	0.95228	1.0966	0.133
TAG562-FA160	0.86892	0.9716	1.0074	0.0106	0.45442	0.89138	1.0163	0.0234	0.028572	0.37947	0.69176	-0.532	0.03331	0.99441	1.4764	0.5621	0.7419	0.93742	1.0531	0.0746
TAG525-FA181	0.004652	0.2407	0.7223	-0.469	0.036166	0.52469	1.2532	0.3256	0.87845	0.95387	0.94833	-0.077	0.74015	0.99441	0.9981	-0.003	0.60146	0.86812	1.047	0.0662
TAG461-FA141	0.025103	0.53437	1.344	0.4265	0.005048	0.27165	0.79573	-0.33	0.76702	0.93401	0.96341	-0.054	0.27433	0.99441	0.91728	-0.125	0.96093	0.98408	1.2301	0.2988
TAG566-FA202	0.38661	0.82612	2.4572	1.297	0.008339	0.27531	1.4857	0.5712	0.002673	0.22723	1.5576	0.6393	0.85476	0.99441	0.97256	-0.04	0.29587	0.70249	2.7757	1.4729
TAG460-FA180	0.98487	0.99423	1.0074	0.0106	0.25248	0.89138	1.1028	0.1411	0.025165	0.345	1.3535	0.4367	0.49415	0.99441	0.91556	-0.127	0.11525	0.55485	0.89402	-0.162
CE225	0.092004	0.72412	0.72428	-0.465	0.65954	0.93511	1.1391	0.1879	0.89134	0.95881	0.70836	-0.497	0.81807	0.99441	1.0525	0.0738	0.039216	0.54839	0.60765	-0.719
HCER160	0.15178	0.72412	1.5256	0.6094	0.048962	0.59454	1.4705	0.5563	0.004926	0.24745	1.753	0.8099	0.78801	0.99441	1.1208	0.1645	0.15466	0.60235	1.3755	0.46
FFA161	0.040771	0.55896	0.76766	-0.381	0.000291	0.030918	0.50315	-0.991	0.93587	0.97011	0.76141	-0.393	0.024788	0.99441	0.72667	-0.461	0.40024	0.80237	1.3405	0.4227
TAG521-FA181	0.35237	0.80541	1.1355	0.1833	0.7741	0.97247	1.0417	0.0589	0.92319	0.96737	0.80541	-0.312	0.53409	0.99441	0.89627	-0.158	0.048595	0.54839	0.82044	-0.286
HCER201	0.11929	0.72412	0.77487	-0.368	0.020173	0.37637	1.7709	0.8245	0.002133	0.22668	3.4405	1.7826	0.2352	0.99441	1.4581	0.5441	0.05465	0.54839	1.3078	0.3872
FFA204	0.78356	0.9716	1.1989	0.2617	0.29959	0.89138	1.2785	0.3544	0.020156	0.33072	1.3881	0.4731	0.62314	0.99441	1.1533	0.2058	0.20811	0.63629	1.3938	0.479
TAG400-FA160	0.42462	0.83222	0.92457	-0.113	0.40795	0.89138	1.0511	0.0719	0.009043	0.25623	1.5882	0.6674	0.66875	0.99441	0.90318	-0.147	0.78908	0.95228	1.1269	0.1724
TAG521-FA180	0.54089	0.88884	1.0918	0.1267	0.62128	0.91862	1.0515	0.0725	0.58546	0.85799	0.97508	-0.036	0.4944	0.99441	0.89175	-0.165	0.016239	0.43136	0.7703	-0.377
TAG524-FA180	0.03588	0.5446	0.70239	-0.51	0.2815	0.89138	1.1076	0.1474	0.092059	0.49525	1.2703	0.3452	0.46529	0.99441	1.1737	0.2311	0.2049	0.63629	1.3777	0.4623
TAG563-FA182	0.83121	0.9716	1.0072	0.0103	0.055655	0.63604	0.78706	-0.345	0.04753	0.42979	0.68543	-0.545	0.099327	0.99441	0.73795	-0.438	0.8419	0.96019	0.98511	-0.022
DAG181/181	0.44979	0.85287	0.93718	-0.094	0.019662	0.37637	1.2292	0.2977	0.72945	0.91893	1.0156	0.0223	0.93284	0.99441	1.0151	0.0216	0.4559	0.8197	1.0888	0.1227
N-ACETYLALANINE_neg_1	0.014195	0.43092	2.5221	1.3346	0.16404	0.87306	1.9001	0.9261	0.24086	0.64952	1.1236	0.1681	0.040203	0.99441	1.5667	0.6478	0.19231	0.62869	0.7937	-0.333
DAG180/181	0.27222	0.78188	0.87619	-0.191	0.042551	0.55662	1.3134	0.3933	0.024427	0.345	1.2889	0.3662	0.23153	0.99441	1.1442	0.1944	0.38655	0.79813	1.0469	0.0661
TAG534-FA170	0.65261	0.96411	1.0154	0.0221	0.6846	0.93856	1.1058	0.1451	0.68867	0.90614	0.90843	-0.139	0.88273	0.99441	1.1165	0.1589	0.003655	0.31319	1.8248	0.8677
TAG481-FA180	0.41435	0.83033	0.96102	-0.057	0.67878	0.93856	1.0241	0.0344	0.062462	0.4396	1.2689	0.3436	0.77564	0.99441	0.98759	-0.018	0.005573	0.31319	0.83587	-0.259
NADPH_pos_1	0.99144	0.99612	1.3024	0.3811	0.11742	0.76624	0.58476	-0.774	0.020533	0.33072	3.5062	1.8099	0.81175	0.99441	1.6467	0.7196	0.41889	0.81279	0.86165	-0.215
TAG484-FA182	0.020087	0.53356	0.59745	-0.743	0.85386	0.98587	0.9095	-0.137	0.042723	0.42979	0.67529	-0.566	0.8132	0.99441	0.9607	-0.058	0.050006	0.54839	1.5346	0.6179
XANTHURENIC ACID_pos_2	0.83684	0.9716	2.1943	1.1338	0.32002	0.89138	0.89005	-0.168	0.80952	0.93783	0.75844	-0.399	0.019796	0.99441	0.85596	-0.224	0.54905	0.85329	1.3136	0.3935
TAG523-FA203	0.08223	0.69896	0.67227	-0.573	0.26799	0.89138	1.3282	0.4095	0.6059	0.86553	0.95556	-0.066	0.02172	0.99441	1.4757	0.5614	0.23959	0.67154	1.1439	0.194
TAG531-FA180	0.028688	0.53437	0.81774	-0.29	0.43319	0.89138	1.051	0.0718	0.78473	0.93401	1.0295	0.0419	0.6498	0.99441	0.98236	-0.026	0.68078	0.90885	0.97444	-0.037
SM241	0.19364	0.72412	1.0518	0.0729	2.22E-05	0.005935	1.8333	0.8744	0.86629	0.95387	0.95123	-0.072	0.6111	0.99441	0.99607	-0.006	0.88637	0.96019	1.2814	0.3577
SM220	0.06619	0.67761	1.6294	0.7044	0.008421	0.27531	2.2471	1.168	0.54588	0.83754	1.1852	0.2451	0.80712	0.99441	1.1529	0.2053	0.005338	0.31319	2.1942	1.1337
DAG180/182	0.067977	0.67761	1.2799	0.3561	0.25791	0.89138	1.2865	0.3634	0.038363	0.42979	1.5649	0.6461	0.1115	0.99441	1.3456	0.4283	0.22853	0.66984	1.2752	0.3507
TAG565-FA202	0.49553	0.86825	2.6831	1.4239	0.01894	0.37637	1.4132	0.499	0.004814	0.24745	1.4482	0.5343	0.67761	0.99441	0.94624	-0.08	0.66194	0.90885	2.3849	1.2539
ANTHRANILIC ACID_pos_4	0.9173	0.9716	0.725	-0.464	0.7091	0.94138	1.004	0.0057	0.009662	0.25623	0.56723	-0.818	0.81369	0.99441	0.73803	-0.438	0.42074	0.81279	1.4537	0.5398
TAG543-FA202	0.41895	0.83035	2.5946	1.3755	0.01721	0.37637	1.4243	0.5103	0.007276	0.24745	1.3889	0.474	0.73977	0.99441	0.94902	-0.075	0.743	0.93742	2.0694	1.0492
DAG160/160	0.029747	0.53437	0.83907	-0.253	0.90988	0.99	1.0671	0.0937	0.50761	0.81765	0.91986	-0.121	0.64839	0.99441	0.99706	-0.004	0.98167	0.99336	0.99001	-0.014
TAG490-FA150	0.8517	0.9716	0.99762	-0.003	0.5107	0.89138	0.97026	-0.044	0.63698	0.89385	0.99122	-0.013	0.80393	0.99441	0.98318	-0.024	0.032964	0.54839	0.90076	-0.151
2-KETOHEXANOIC ACID_pos_2	0.19656	0.72412	1.3059	0.385	0.56468	0.89995	1.0031	0.0044	0.013817	0.31736	1.4449	0.531	0.14435	0.99441	1.5869	0.6662	0.0843	0.54839	1.4462	0.5323
TAG503-FA181	0.25519	0.77754	1.1233	0.1678	0.25482	0.89138	0.82913	-0.27	0.04618	0.42979	0.53554	-0.901	0.98859	0.99759	0.98747	-0.018	0.40944	0.80947	0.93463	-0.098
DAG161/182	0.88001	0.9716	0.97848	-0.031	0.30899	0.89138	1.4085	0.4942	0.029752	0.38105	1.6402	0.7139	0.10021	0.99441	1.4356	0.5217	0.52201	0.85329	1.0766	0.1064
ADENOSINE PHOSPHOSULFATE_neg	0.72047	0.9716	1.3889	0.474	0.000268	0.030918	8.2873	3.0509	0.001808	0.22668	4.1672	2.0591	0.7818	0.99441	0.81246	-0.3	0.94761	0.97514	1.3002	0.3787
DAG140/182	0.81048	0.9716	0.9592	-0.06	0.027058	0.44229	1.7923	0.8418	0.20954	0.64834	1.2941	0.372	0.84148	0.99441	1.0822	0.1139	0.53096	0.85329	1.1317	0.1785
CE150	0.062569	0.67761	1.4537	0.5397	0.32894	0.89138	1.3723	0.4565	0.64532	0.89921	0.71465	-0.485	0.007494	0.99441	1.5832	0.6628	0.52854	0.85329	1.0708	0.0987
2-HYDROXY-3-METHYLBUTYRIC ACII	0.26048	0.77754	1.1964	0.2587	0.96746	0.99	1.0107	0.0153	0.043427	0.42979	1.4759	0.5616	0.53023	0.99441	1.2553	0.328	0.55149	0.85329	1.359	0.4426
TAG502-FA160	0.006568	0.25375	1.2331	0.3023	0.83024	0.97366	1.0908	0.1254	0.13611	0.54035	0.50475	-0.986	0.5414	0.99441	0.95757	-0.063	0.76613	0.95206	1.0259	0.037
TAG482-FA182	0.33051	0.7915	0.94218	-0.086	0.81096	0.97366	0.98949	-0.015	0.47883	0.8154	0.91075	-0.135	0.47965	0.99441	0.95096	-0.073	0.013974	0.39593	0.83194	-0.265
TAG564-FA202	0.50825	0.87452	2.611	1.3846	0.021015	0.37637	1.3938	0.4791	0.005859	0.24745	1.4181	0.5039	0.56479	0.99441	0.92921	-0.106	0.68149	0.90885	2.1468	1.1022
TAG563-FA202	0.46804	0.85576	2.5022	1.3232	0.016614	0.37637	1.4327	0.5188	0.006765	0.24745	1.453	0.539	0.71262	0.99441	0.95302	-0.069	0.73699	0.93742	1.9027	0.928
TAG543-FA160	0.032869	0.54007	1.4862	0.5716	0.35788	0.89138	1.064	0.0895	0.28992	0.67701	0.73318	-0.448	0.67359	0.99441	1.105	0.1441	0.01298	0.39402	1.3932	0.4784
TAG533-FA182	0.004532	0.2407	0.70078	-0.513	0.4137	0.89138	1.1437	0.1937	0.109	0.52084	0.80235	-0.318	0.52865	0.99441	0.9344	-0.098	0.70625	0.91931	0.94101	-0.088
TAG524-FA160	0.15184	0.72412	1.1767	0.2348	0.50246	0.89138	1.6478	0.7206	0.44063	0.78215	0.53024	-0.915	0.72045	0.99441	1.1257	0.1708	0.047463	0.54839	1.295	0.373
TAG526-FA160	0.033039	0.54007	0.6084	-0.717	0.44144	0.89138	1.1956	0.2577	0.76377	0.93401	0.9508	-0.073	0.93091	0.99441	0.95062	-0.073	0.27048	0.68836	0.78766	-0.344
TAG504-FA161	0.005184	0.2407	0.70164	-0.511	0.32723	0.89138	0.81374	-0.297	0.19324	0.63664	0.68761	-0.54	0.49121	0.99441	1.0787	0.1093	0.14496	0.58674	0.84058	-0.251
SM180	0.18951	0.72412	0.90247	-0.148	0.82829	0.97366	1.0069	0.01	0.97721	0.99242	0.95279	-0.07	0.038441	0.99441	0.75398	-0.407	0.46184	0.8197	1.0844	0.1169
LCER160	0.001655	0.14065	2.0271	1.0194	2.79E-05	0.005935	3.1023	1.6333	4.55E-06	0.001935	2.959	1.5651	0.012064	0.99441	1.9713	0.9791	0.29084	0.70059	1.4806	0.5662
TAG525-FA205	0.026626	0.53437	0.74728	-0.42	0.005353	0.27165	1.7693	0.8232	0.006742	0.24745	1.9223	0.9429	0.52293	0.99441	1.105	0.1441	0.10887	0.55082	1.4957	0.5808
BETAINE_pos_2	0.012766	0.41736	2.6097	1.3839	0.60414	0.90984	0.94069	-0.088	0.49883	0.8154	0.82669	-0.275	0.58837	0.99441	0.75896	-0.398	0.66181	0.90885	1.5616	0.643
TAG481-FA140	0.19421	0.72412	1.092	0.127	0.81878	0.97366	0.98957	-0.015	0.87676	0.95387	0.94404	-0.083	0.4825	0.99441	0.9405	-0.089	0.005895	0.31319	0.80791	-0.308
TAG524-FA204	0.030176	0.53437	0.56973	-0.812	0.021254	0.37637	1.5836	0.6632	0.024848	0.345	1.693	0.7596	0.43065	0.99441	1.0912	0.1259	0.42538	0.81621	1.2279	0.2962
TAG492-FA140	0.68885	0.9716	1.2264	0.2945	0.65209	0.93511	0.81778	-0.29	0.19998	0.64834	0.70832	-0.498	0.31416	0.99441	1.1494	0.2009	0.00816	0.34678	1.347	0.4297

CE205	0.025904	0.53437	0.62916	↓	-0.669	0.038271	0.52469	1.6774	↑	0.7462	0.65328	0.90438	0.3005	↓	-1.735	0.22127	0.99441	1.3039	↑	0.3828	0.78678	0.95228	0.97992	↓	-0.029
O-ACETYLSERINE_pos_3	0.18715	0.72412	2.0492	↑	1.0351	0.048402	0.59454	0.73532	↓	-0.444	0.40671	0.77512	0.72612	↓	-0.462	0.82674	0.99441	1.0636	↑	0.0889	0.90894	0.96019	1.0129	↑	0.0185
TAG544-FA183	0.20675	0.72412	0.76318	↓	-0.39	0.30285	0.89138	1.5735	↑	0.654	0.48649	0.8154	0.59663	↓	-0.745	0.54046	0.99441	1.1352	↑	0.1829	0.041117	0.54839	1.5314	↑	0.6148
TAG502-FA182	0.001212	0.14065	1.3434	↑	0.4259	0.53752	0.89587	0.99988	↓	-2E-04	0.24289	0.64952	0.69552	↓	-0.524	0.69353	0.99441	0.98149	↓	-0.027	0.41899	0.81279	0.9415	↓	-0.087
TAG524-FA161	0.025975	0.53437	1.3428	↑	0.4252	0.37498	0.89138	0.93278	↓	-0.1	0.16365	0.59496	0.40092	↓	-1.319	0.64863	0.99441	0.92973	↓	-0.105	0.46289	0.8197	0.89102	↓	-0.166
TAG502-FA140	0.53182	0.88822	1.0953	↑	0.1313	0.86496	0.986	0.98865	↓	-0.016	0.8158	0.94216	0.81193	↓	-0.301	0.70852	0.99441	0.9675	↓	-0.048	0.046	0.54839	0.86154	↓	-0.215
TAG461-FA180	0.28656	0.78188	0.84951	↓	-0.235	0.5835	0.89995	1.1023	↑	0.1405	0.008251	0.25047	1.4686	↑	0.5545	0.20628	0.99441	0.88423	↓	-0.178	0.25887	0.67735	0.8758	↓	-0.191
MYOINOSITOL_neg_3	0.69403	0.9716	0.55353	↓	-0.853	0.50361	0.89138	0.84704	↓	-0.239	0.012975	0.31736	0.58412	↓	-0.776	0.49663	0.99441	0.62893	↓	-0.669	0.69708	0.91156	0.67767	↓	-0.561
ASPARTATE_pos_3	0.25997	0.77754	1.2131	↑	0.2787	0.58481	0.89995	0.9979	↓	-0.003	0.030484	0.38105	1.4773	↑	0.563	0.15291	0.99441	1.5975	↑	0.6758	0.060721	0.54839	1.4918	↑	0.577
TAG472-FA182	0.19253	0.72412	0.85769	↓	-0.221	0.92084	0.99	0.96578	↓	-0.05	0.41407	0.78213	1.0062	↑	0.009	0.56608	0.99441	1.1004	↑	0.1381	0.007358	0.34678	0.84219	↓	-0.248
TAG568-FA204	0.50722	0.87452	0.91645	↓	-0.126	0.27862	0.89138	1.0917	↑	0.1265	0.043545	0.42979	1.2326	↑	0.3017	0.72618	0.99441	1.0258	↑	0.0367	0.55676	0.85329	1.9027	↑	0.928
SM240	0.19681	0.72412	0.90956	↓	-0.137	0.003548	0.25129	2.4871	↑	1.3145	0.125	0.52084	1.338	↑	0.42	0.80761	0.99441	0.96977	↓	-0.044	0.68687	0.90885	1.115	↑	0.1571

Supplementary Table S3C. List of dysregulated metabolites for wildtype mice following exposure to 9.5 Gy of Gamma-radiation, 24 h, 1 week, 1 month, 3 month and 6 months post-irradiation.

	9.5Gy / 0.0Gy				9.5Gy / 0.0Gy				9.5Gy / 0.0Gy				9.5Gy / 0.0Gy				9.5Gy / 0.0Gy			
	24h				1wk				1mo				3mo				6mo			
	WildType																			
name	p-value	FDR	FC	LOG2FC	p-value	FDR	FC	LOG2FC	p-value	FDR	FC	LOG2FC	p-value	FDR	FC	LOG2FC	p-value	FDR	FC	LOG2FC
XANTHOSINE_neg_1	0.001628	0.27225	2.5482	1.3495	0.11152	0.92112	1.3709	0.45509	0.078053	0.23527	1.5013	0.58618	0.44197	0.87102	0.5508	-0.86041	0.49426	0.70254	0.97529	-0.03609
CYSTEINE_neg_5	0.009274	0.42014	2.3964	1.2609	0.22642	0.92112	1.5092	0.59382	0.60859	0.77209	1.7783	0.83053	0.068388	0.80329	3.534	1.8213	0.45323	0.67019	0.83194	-0.26545
PYROPHOSPHATE_neg_3	0.013629	0.42014	4.7208	2.239	0.48252	0.95381	3.4897	1.8031	0.42327	0.62738	2.1417	1.0988	0.64663	0.90034	1.0507	0.071371	0.59565	0.76819	1.3075	0.38682
N-ACETYLGLUTAMINE_pos_2	0.001922	0.27225	5.8258	2.5425	0.46214	0.93084	0.7982	-0.32518	0.7206	0.84835	1.2416	0.31224	0.67318	0.90034	1.2764	0.35211	0.15534	0.43149	1.0154	0.022023
CE204	0.001529	0.27225	2.6901	1.4277	0.16872	0.92112	1.5095	0.59408	0.7694	0.86278	0.13108	-2.9314	0.33517	0.85641	0.87976	-0.18482	0.97004	0.98244	0.92129	-0.11827
OXOGLUTARATE_neg_2	0.46008	0.93557	0.12072	-3.0503	0.71953	0.96398	0.38294	-1.3848	0.1653	0.36589	0.85051	-0.2336	0.17878	0.80329	0.66721	-0.58379	0.004073	0.096167	0.44246	-1.1764
NORMETANEPHRINE_pos_1	0.03033	0.51706	2.3156	1.2114	0.68153	0.96398	1.4455	0.53156	0.15034	0.35011	3.001	1.5854	0.57118	0.89473	0.90455	-0.14472	0.86848	0.93681	1.1874	0.24781
ASCORBATE_neg_2	0.016726	0.42014	2.4919	1.3173	0.15216	0.92112	1.9249	0.94479	0.072301	0.22594	0.43997	-1.1845	0.53761	0.89334	0.91183	-0.13317	0.14617	0.42261	0.6195	-0.69083
ACETYL-COA_pos_1	0.015224	0.42014	2.2577	1.1749	0.5689	0.96398	0.86268	-0.2131	0.97108	0.9803	0.97645	-0.03439	0.064487	0.80329	0.48002	-1.0588	0.64247	0.81024	1.1514	0.20336
AMINOADIPATE_pos_1	0.01987	0.42014	2.3907	1.2574	0.83039	0.9749	0.75565	-0.40421	0.67528	0.82234	1.0614	0.085907	0.85965	0.97168	1.1475	0.19846	0.31058	0.5617	1.6698	0.73971
CE226	0.031632	0.51706	1.654	0.72599	0.78942	0.9749	1.1942	0.25603	0.59522	0.77209	0.26626	-1.9091	0.20003	0.80329	1.4064	0.49198	0.46203	0.67413	1.2804	0.35663
TAG566-FA225	0.43103	0.92598	0.90211	-0.14862	0.14018	0.92112	0.7194	-0.47513	0.90521	0.94062	0.65659	-0.60694	0.026794	0.80329	0.55359	-0.8531	0.48543	0.69231	0.88227	-0.1807
GERANYL-PP_-HPO3_neg_2	0.026683	0.49306	1.7411	0.79997	0.57723	0.96398	1.5458	0.62831	0.6602	0.81329	0.80591	-0.31131	0.93103	0.98186	1.2262	0.29425	0.57358	0.75705	0.96765	-0.04745
TAG544-FA224	0.044573	0.60334	1.3326	0.41422	0.1947	0.92112	1.5742	0.65464	0.028438	0.12722	1.7612	0.81652	0.62928	0.90034	0.9173	-0.12454	0.50995	0.71527	1.07	0.097653
ACETYLPHOSPHATE_pos_1	0.083378	0.6741	0.42353	-1.2395	0.93486	0.98807	0.98919	-0.01568	0.66573	0.81538	0.86834	-0.20366	0.88585	0.97744	1.2289	0.29732	0.004015	0.096167	0.60446	-0.72628
TAG565-FA181	0.84697	0.99234	0.88768	-0.17189	0.74326	0.96398	0.46684	-1.099	0.56901	0.76038	0.92283	-0.11586	0.033546	0.80329	0.22613	-2.1448	0.030879	0.26735	0.18395	-2.4426
TAG501-FA140	0.98838	0.99794	1.0621	0.086871	0.031027	0.92112	0.69763	-0.51946	0.56211	0.76038	0.9058	-0.14274	0.82156	0.96459	1.0081	0.011658	0.52346	0.72467	0.91544	-0.12747
TAG527-FA160	0.017205	0.42014	0.83746	-0.25591	0.50303	0.96398	1.0872	0.12064	0.33261	0.56544	1.2071	0.27153	0.36532	0.86725	0.77906	-0.36018	0.44956	0.66918	0.89842	-0.15454
TAG545-FA205	0.034846	0.5218	0.51974	-0.94413	0.83898	0.97588	1.0426	0.060126	0.42367	0.62738	1.4898	0.57516	0.54921	0.89371	0.87297	-0.196	0.5916	0.76819	0.85403	-0.22765
TAG483-FA182	0.62524	0.98776	0.97133	-0.04197	0.18543	0.92112	0.87339	-0.1953	0.003112	0.045599	0.447	-1.1616	0.51879	0.89334	1.052	0.073198	0.083354	0.34394	0.88571	-0.1751
OROTATE_neg_1	0.047338	0.60966	2.1738	1.1202	0.40096	0.92112	0.76624	-0.38413	0.50256	0.71196	0.73909	-0.43617	0.89862	0.97744	1.1083	0.1484	0.34749	0.57989	1.3213	0.40195
DAG160/204	0.31142	0.9255	1.595	0.67357	0.12597	0.92112	1.3885	0.47349	0.002835	0.043037	3.0526	1.61	0.92574	0.98083	0.88028	-0.18397	0.052165	0.29221	2.031	1.0222
ADP_pos_1	0.47752	0.94066	1.2192	0.28594	0.05453	0.92112	1.549	0.63137	0.031983	0.1373	1.7857	0.8365	0.44268	0.87102	1.5749	0.65523	0.80667	0.90302	0.96157	-0.05654
SM160	0.92107	0.99234	0.8225	-0.28191	0.17776	0.92112	1.5072	0.59191	0.011652	0.078206	1.749	0.80657	0.40719	0.87102	1.3846	0.46946	0.073545	0.33329	1.2898	0.3671
TAG564-FA204	0.29613	0.9254	0.7964	-0.32844	0.23162	0.92112	1.4402	0.52629	0.001354	0.034775	3.1709	1.6649	0.16757	0.80329	0.61967	-0.69043	0.68288	0.82921	0.94501	-0.0816
PYRIDOXAMINE_pos_1	0.010487	0.42014	2.0965	1.068	0.20163	0.92112	0.59765	-0.74263	0.57073	0.76038	1.7007	0.76609	0.32963	0.85641	0.44386	-1.1718	0.58985	0.76819	1.2093	0.27416
L-2-HYDROXYGLUTARIC ACID_neg_1	0.14301	0.74123	0.24129	-2.0511	0.085548	0.92112	1.1306	0.17707	0.024261	0.11208	0.43151	-1.2125	0.2151	0.80329	0.7959	-0.32934	0.057669	0.29221	1.9511	0.96429
CE181	0.40887	0.92598	1.0504	0.070873	0.49013	0.95869	0.91846	-0.12271	0.25255	0.49272	0.10209	-3.2921	0.76859	0.95473	0.98459	-0.0224	0.001603	0.096167	0.73912	-0.43613
TAG568-FA161	0.65752	0.98776	1.1818	0.24098	0.023568	0.92112	1.494	0.57915	0.1556	0.35553	0.54427	-0.8776	0.16791	0.80329	1.2782	0.35415	0.36021	0.58919	1.1827	0.24211
FFA201	0.38907	0.92598	0.92572	-0.11135	0.1508	0.92112	1.2616	0.33525	0.01806	0.094674	1.8038	0.85103	0.65266	0.90034	0.94045	-0.08857	0.84349	0.92216	1.0191	0.027303
IMIDAZOLE_pos_1	0.016685	0.42014	1.498	0.58302	0.67253	0.96398	0.89698	-0.15686	0.23953	0.47794	1.9621	0.97243	0.99526	0.99678	1.0536	0.075385	0.13639	0.40298	1.4032	0.48869
3-METHYLAMINO-L-ALANINE_pos_2	0.044661	0.60334	2.146	1.1017	0.23078	0.92112	1.3866	0.47156	0.19439	0.41308	0.6455	-0.63151	0.40932	0.87102	0.78995	-0.34016	0.19179	0.47815	2.3113	1.2087
FFA182	0.54201	0.94319	1.0997	0.1371	0.38795	0.92112	1.1013	0.13919	0.015176	0.084864	2.1363	1.0951	0.89143	0.97744	0.98237	-0.02566	0.46455	0.67413	1.0662	0.092512
TAG540-FA180	0.061326	0.61472	0.58478	-0.77403	0.71585	0.96398	0.97565	-0.03556	0.004649	0.053283	1.9789	0.98469	0.063334	0.80329	0.64921	-0.62325	0.66646	0.82295	1.1159	0.15815
TAG463-FA141	0.54912	0.94319	1.1168	0.15936	0.93556	0.98807	1.0816	0.11322	0.097405	0.26708	0.53124	-0.91258	0.92006	0.98001	1.1125	0.15375	0.04589	0.29047	1.2072	0.27172
TAG567-FA180	0.018172	0.42014	0.4691	-1.092	0.6458	0.96398	1.1372	0.18552	0.028939	0.12812	1.6254	0.7008	0.10098	0.80329	0.73252	-0.44906	0.2805	0.53943	0.78847	-0.34288
TAG442-FA160	0.16718	0.80971	1.425	0.51093	0.89026	0.98395	1.041	0.057946	0.001419	0.034775	2.0634	1.045	0.57104	0.89473	1.0235	0.033441	0.79515	0.89878	1.1696	0.22605
TAG505-FA205	0.22304	0.8777	0.65952	-0.60052	0.44024	0.93084	1.1161	0.15844	0.042228	0.16646	1.835	0.8758	0.75743	0.9533	0.95448	-0.06721	0.28358	0.54151	1.1336	0.18088
TAG520-FA160	0.016191	0.42014	0.68048	-0.55538	0.87906	0.98395	1.0723	0.10075	0.022383	0.1057	1.5825	0.6622	0.059757	0.80329	0.68236	-0.5514	0.84405	0.92216	0.98735	-0.01836
ARGININOSUCCINIC ACID_neg_2	0.46507	0.93603	0.60446	-0.72628	0.65732	0.96398	0.57919	-0.78788	0.60797	0.77209	0.91698	-0.12504	0.70916	0.92392	0.71807	-0.47781	0.043315	0.29047	3.2224	1.6881
FFA141	0.82925	0.99234	0.97993	-0.02924	0.091353	0.92112	1.3726	0.4569	0.00685	0.062783	2.2389	1.1628	0.74617	0.94946	0.90843	-0.13856	0.43236	0.65392	0.85079	-0.23313
TAG482-FA140	0.91421	0.99234	0.99051	-0.01376	0.18301	0.92112	0.87434	-0.19373	0.004937	0.053803	0.45983	-1.1208	0.50219	0.88561	0.91171	-0.13336	0.034566	0.26873	0.86765	-0.20482
PHENYLPROPIOLIC ACID_neg_1	0.43559	0.92598	0.18999	-2.396	0.33055	0.92112	1.5504	0.63263	0.20718	0.42951	0.85299	-0.22939	0.70857	0.92392	0.89576	-0.15882	0.009623	0.14102	0.45136	-1.1476
TAG482-FA141	0.83263	0.99234	1.04	0.056613	0.4592	0.93084	0.94543	-0.08096	0.000847	0.034775	0.48689	-1.0383	0.80087	0.95886	1.0467	0.06586	0.67373	0.82465	1.0028	0.004008
FFA226	0.37764	0.92598	0.8926	-0.16391	0.71994	0.96398	1.0868	0.12015	0.00421	0.053283	2.0492	1.0351	0.10446	0.80329	0.71578	-0.48241	0.24192	0.51246	1.1383	0.18683
TAG562-FA180	0.87526	0.99234	0.99672	-0.00474	0.46936	0.94094	0.72372	-0.46649	0.002092	0.035955	2.2509	1.1705	0.080098	0.80329	0.65481	-0.61084	0.21784	0.50044	0.67904	-0.55842
TAG491-FA150	0.91989	0.99234	0.99466	-0.00773	0.28959	0.92112	0.83951	-0.25239	0.001473	0.034775	0.54516	-0.87524	0.92528	0.98083	1.0247	0.035243	0.020922	0.26022	0.76008	-0.39578
TAG461-FA140	0.7967	0.99234	1.021	0.03003	0.65274	0.96398	0.92956	-0.10539	0.009001	0.065952	0.56313	-0.82845	0.24177	0.80329	1.216	0.2821	0.26558	0.52446	1.0675	0.094169
TAG441-FA120	0.26129	0.9166	0.91649	-0.12581	0.89909	0.98395	0.92378	-0.11439	0.042301	0.16646	0.64182	-0.63976	0.40009	0.87102	1.0958	0.13205	0.21123	0.49055	1.0834	0.1156
TAG567-FA204	0.55558	0.94319	0.81243	-0.29969	0.70374	0.96398	0.74286	-0.42883	0.31311	0.55402	1.1517	0.20371	0.005698	0.80329	0.20571	-2.2813	0.007906	0.1261	0.40754	-1.295
TAG471-FA140	0.9603	0.99234	0.99879	-0.00175	0.7065	0.96398	1.0522	0.073351	0.026941	0.12181	0.47426	-1.0762	0.14817	0.80329	1.2187	0.2853	0.401	0.63004	1.0306	0.043464
TAG451-FA181	0.034288	0.5218	0.82377	-0.27968	0.30926	0.92112	1.0965	0.13285	0.72843	0.85424	0.96929	-0.045	0.15052	0.80329	1.1351	0.18288	0.28762	0.54151	1.0694	0.096775
METHYL PHENYLACETATE_neg_3	0.008059	0.42014	0.28761	-1.7978	0.011513	0.92112	0.41917	-1.2544	0.64442	0.79991	1.677	0.74586	0.33943	0.85641	0.98673	-0.01927	0.51513	0.71746	1.1612	0.21556
TAG501-FA181	0.018851	0.42014	1.1584	0.21216	0.046167	0.92112	0.73648	-0.44128	0.66303	0.81441	0.67413	-0.5689	0.9837	0.99592	0.97678	-0.03389	0.002154	0.096167	0.64091	-0.6418
FFA205	0.95267	0.99234	0.98074	-0.02806	0.26056	0.92112	1.2587	0.33193	0.000865	0.034775	2.5798	1.3673	0.65117	0.90034	0.96734	-0.0479	0.78137	0.8903	0.93625	-0.09504
TAG520-FA180	0.021748	0.42014	0.68558	-0.54459	0.90102	0.98395	1.0484	0.068177	0.004635	0.053283	1.8318	0.87323	0.090539	0.80329	0.73029	-0.45345	0.68688	0.82933	0.94978	-0.07433
TAG483-FA141	0.50159	0.94319	0.95256	-0.07012	0.94855	0.98807	0.98418	-0.023	0.001205	0.034775	0.56007	-0.83631	0.12738	0.80329	0.8508	-0.23311	0.23294	0.51246	1.0626	0.087541
TAG462-FA161	0.67426	0.99234	0.98017	-0.0289	0.73396	0.96398	1.0156	0.022386	0.040141	0.16247	0.43793	-1.1912	0.22676	0.80329	1.2801	0.35622	0.2078	0.49055	1.0841	0.11656
TAG462-FA140	0.65833	0.98776	1.0293	0.041684	0.78956	0.9749	1.0041	0.005884	0.007054	0.062783	0.50981	-0.97198	0.43999	0.87102	1.1749	0.23252	0.22754	0.5117	1.0756	0.10515
TAG461-FA160	0.93241	0.99234	0.99512	-0.00706	0.61918	0.96398	0.90863	-0.13823	0.008948	0.065952	0.59278	-0.75444	0.35552	0.85923	1.1568	0.21013	0.31792	0.56771	1.0537	0.075483
TAG511-FA150	0.70074	0.99234	1.1295	0.17571	0.032099	0.92112	0.67173	-0.57405	0.21447	0.44034	0.79559	-0.32991	0.87039	0.97346	0.91059	-0.13513	0.20821	0.49055	0.63249	-0.66089
DAG181/226	0.14947	0.76265	1.2612	0.33475	0.10164	0.92112	1.2719	0.34694	0.002039	0.035955	2.4724	1.3059	0.66358	0.90034	0.9674	-0.04781	0.15245	0.42754	1.253	0.32533
TAG483-FA161	0.76252	0.99234	0.98118	-0.02741	0.36504	0.92112	1.1405	0.18961	0.00472	0.053283	0.45922	-1.1227	0.54235	0.89334	1.1415	0.19096	0.24465	0.51246	1.0745	0.10366
TAG482-FA181	0.78415	0.99234	0.98669	-0.01933	0.28022	0.92112	0.85226	-0.23064	0.014609	0.083905	0.52439	-0.93127	0.67542	0.90034	1.0888	0.1227	0.55886	0.75282	1.0162	0.023224
TAG544-FA160	0.35168	0.92598	1.141	0.19031	0.39732	0.92112	0.82476	-0.27795	0.038908	0.16054	1.6541	0.72604	0.41358	0.87102	0.84603	-0.24122	0.3389	0.57989	0.72748	-0.45902
DAG160/182	0.58599	0.95419	1.0387	0.054749	0.13177	0.92112	1.3291	0.41046	0.31416	0.55402	1.1982	0.26085	0.7814	0.95886	0.93874	-0.09121	0.003635	0.096167	1.4437	0.52979
TAG542-FA202	0.19962	0.83997	0.81887	-0.28829	0.92647	0.98807	1.0631	0.088231	0.008776	0.065952	1.5805	0.66035	0.26364	0.80329	0.86298	-0.2126	0.2423	0.51246	0.83809	-0.25482
TAG523-FA161	0.38432	0.92598	1.0695	0.096932	0.58561	0.96398	0.80096	-0.3202	0.049569	0.18161	0.39673	-1.3338	0.15931	0.80329	0.4547	-1.137	0.033121	0.26873	0.51489	-0.95766
FFA180	0.062946	0.61472	0.82943	-0.26981	0.2775	0.92112	1.2257	0.29365	0.014124	0.0835	1.873	0.90533	0.31695	0.85641	0.79345	-0.33379	0.74041	0.87165	1.0101	0.01454
CE161	0.87534	0.99234	0.98647	-0.01965	0.091776	0.92112	1.2307	0.29944	0.000207	0.034775	0.17692	-2.4989	0.12001	0.80329	1.3036	0.38255	0.83447	0.91923	0.87719	-0.18903
TAG531-FA181	0.035605	0.5218	0.72724	-0.4595	0.92337	0.98807	0.88923	-0.16936	0.35685	0.58192	0.86016	-0.21733	0.51277	0.89147	0.95653	-0.06411	0.001819	0.096167	0.69696	-0.52085
TAG461-FA161	0.85644	0.99234	0.99045	-0.01385	0.82833	0.9749	1.0118	0.016945	0.020313	0.10105	0.44627	-1.164	0.13711	0.80329	1.2973	0.37552	0.30476	0.5583	1.0593	0.083136
TAG440-FA180	0.42021	0.92598	0.93336	-0.09949	0.99161	0.99785	0.90226	-0.14838	0.021434	0.10235	2.2297	1.1568	0.67564	0.90034	1.0679	0.094775	0.41654	0.6412	1.0442	0.062418
TAG472-FA140	0.9983	0.9983	1.0311	0.044167	0.54252	0.96398	1.0733	0.10208	0.023653	0.11047	0.45642	-1.1316	0.80765	0.95886	1.1053	0.14446	0.99614	0.99681	0.95877	-0.06074
TAG501-FA180	0.8463	0.99234	1.0205	0.029333	0.040684	0.92112	0.70888	-0.4964	0.54945	0.75817	0.92807	-0.10769	0.6102	0.90034	0.89154	-0.16563	0.006611	0.1261	0.72176	-0.47041
TAG482-FA160	0.70084	0.99234	0.98137	-0.02713	0.16516	0.92112	0.86748	-0.20509	0.002115	0.035955	0.4807	-1.0568	0.7321	0.93436	0.93223	-0.10124	0.028388	0.26272	0.87696	-0.18942
TAG491-FA181	0.74328	0.99234	1.0121	0.017405	0.19023	0.92112	0.86819	-0.20391	0.001051	0.034775	0.54482	-0.87614	0.63541	0.90034	1.0649	0.090726	0.00222	0.096167	0.79923	-0.32331
TAG541-FA181	0.21673	0.87371	0.89245	-0.16415	0.94686	0.98807	1.027	0.038379	0.000962	0.034775	1.7535	0.8102	0.2075	0.80329	0.88479	-0.17659	0.71967	0.85864	1.0135	0.01928
TAG422-FA182	0.55967	0.94389	1.0701	0.097795	0.75325	0.96398	0.86492	-0.20936	0.32944	0.56544	2.1943	1.1338	0.60488	0.90034	1.1136	0.15517	0.023879	0.26022	1.2951	0.37308
TAG547-FA183	0.4395	0.92598	1.1265	0.17188	0.30249	0.92112	1.2555	0.32828	0.11532	0.30253	1.8631	0.89772	0.44266	0.87102	1.6234	0.69898	0.000381	0.081031	1.8045	0.85163
TAG525-FA181	0.011024	0.42014	0.74246	-0.42962	0.18697	0.92112	1.1652	0.22059	0.27187	0.52047	1.0778	0.10813	0.062324	0.80329	0.77254	-0.37232	0.72126	0.85864	1.0207	0.029499
TAG521-FA160	0.59921	0.9647	1.0368	0.052155	0.068026	0.92112	0.60913	-0.71518	0.50155	0.71196	0.80839	-0.30688	0.26962	0.80751	0.7413	-0.43188	0.008021	0.1261	0.46737	-1.0974
TAG525-FA183	0.6445	0.98776	1.1308	0.1774	0.39174	0.92112	1.2037	0.26744	0.74709	0.85584	0.54828	-0.86701	0.49786	0.88531	0.9679	-0.04707	0.003058	0.096167	1.6119	0.68878
TAG566-FA202	0.76978	0.99234	0.88029	-0.18394	0.98419	0.99666	1.1276	0.17323	0.001816	0.035955	2.0544	1.0387	0.48501	0.87857	0.8939	-0.16181	0.77084	0.88507	0.91193	-0.13301
TAG501-FA160	0.38122	0.92598	1.0443	0.062523	0.079992	0.92112	0.64837	-0.62512	0.66954	0.81769	0.60362	-0.7283	0.41707	0.87102	0.80964	-0.30464	0.003617	0.096167	0.48567	-1.0419
TAG502-FA181	0.73986	0.99234	1.0183	0.026164	0.03181	0.92112	0.71685	-0.48025	0.052071	0.18271	0.37811	-1.4031	0.45153	0.87228	0.81041	-0.30327	0.002871	0.096167	0.68906	-0.53731
FFA225	0.90348	0.99234	0.99882	-0.0017	0.19533	0.92112	1.3243	0.40521	0.002096	0.035955	2.188	1.1296	0.4186	0.87102	0.86388	-0.21109	0.4573	0.67019	1.0728	0.10141
TAG511-FA181	0.34615	0.92598	1.0424	0.05989	0.15313	0.92112	0.79972	-0.32243	0.015587	0.086032	0.61506	-0.70119	0.88451	0.97744	1.03	0.042672	0.001882	0.096167	0.67619	-0.56449
TAG546-FA183	0.40379	0.92598	1.0956	0.13176	0.3981	0.92112	1.2232	0.29063	0.090369	0.25776	1.2392	0.30937	0.71305	0.92392	1.3029	0.38177	0.001426	0.096167	1.5588	0.6404
TAG501-FA161	0.56212	0.94428	0.97398	-0.03803	0.52406	0.96398	0.89505	-0.15996	0.001251	0.034775	0.48548	-1.0425	0.72605	0.93411	0.89658	-0.15749	0.023562	0.26022	0.80541	-0.3122
TAG532-FA181	0.42165	0.92598	0.92348	-0.11484	0.5516	0.96398	1.0611	0.085556	0.038647	0.16054	1.3417	0.42411	0.38832	0.87102	0.92335	-0.11505	0.99037	0.99505	0.98219	-0.02592
TAG511-FA160	0.90643	0.99234	0.99738	-0.00379	0.65155	0.96398	0.92341	-0.11496	0.046855	0.17468	0.75915	-0.39754	0.83322	0.96583	1.0114	0.016315	0.02224	0.26022	0.78424	-0.35064
TAG521-FA181	0.74963	0.99234	1.0393	0.05568	0.074498	0.92112	0.61106	-0.71061	0.74911	0.85584	0.69107	-0.53309	0.34653	0.85898	0.79496	-0.33104	0.00785	0.1261	0.49081	-1.0268
HCER201	0.43823	0.92598	0.95778	-0.06224	0.47943	0.95214	1.0477	0.067207	0.018489	0.094674	4.6243	2.2092	0.79978	0.95886	1.499	0.584	0.027914	0.26272	1.457	0.54303
TAG545-FA225	0.062317	0.61472	0.73459	-0.44499	0.76489	0.97038	0.99538	-0.00669	0.014342	0.0835	1.8788	0.90982	0.42385	0.87102	0.79306	-0.3345	0.91502	0.95784	0.85695	-0.22272
TAG471-FA181	0.019912	0.42014	0.899	-0.1536	0.61206	0.96398	1.0531	0.074711	0.2007	0.42183	0.7354	-0.44341	0.17384	0.80329	1.1196	0.16293	0.30813	0.55963	1.0505	0.071108
FFA204	0.99809	0.9983	0.96994	-0.04403	0.31989	0.92112	1.2647	0.33877	0.000778	0.034775	2.475	1.3074	0.41734	0.87102	0.88373	-0.17832	0.70034	0.84081	1.0174	0.024929
TAG492-FA160	0.66175	0.98776	0.97821	-0.03179	0.79384	0.9749	0.92171	-0.11761	0.05248	0.18271	0.72344	-0.46706	0.38178	0.87102	0.89928	-0.15316	0.010606	0.15025	0.76294	-0.39037
TAG542-FA201	0.18272	0.82613	0.76639	-0.38385	0.14988	0.92112	0.74865	-0.41764	0.69305	0.83118	0.67365	-0.56994	0.059195	0.80329	0.57318	-0.80293	0.008112	0.1261	0.53822	-0.89374
TAG400-FA160	0.45432	0.9283	0.93887	-0.09101	0.65015	0.96398	0.84543	-0.24224	0.021358	0.10235	2.3463	1.2304	0.75875	0.9533	1.0492	0.069289	0.2779	0.5393	1.0887	0.12262
TAG521-FA180	0.8318	0.99234	0.99603	-0.00574	0.090554	0.92112	0.57055	-0.80958	0.3953	0.60859	0.99652	-0.00503	0.26445	0.80329	0.73045	-0.45314	0.004881	0.098784	0.39918	-1.3249
TAG483-FA120	0.092414	0.69359	0.7522	-0.41081	0.57153	0.96398	0.85867	-0.21983	0.073567	0.22656	0.50744	-0.97869	0.6398	0.90034	0.91919	-0.12157	0.042068	0.29047	1.3864	0.47136
TAG502-FA161	0.88845	0.99234	0.98943	-0.01533	0.33187	0.92112	0.85391	-0.22784	0.001183	0.034775	0.36809	-1.4419	0.47801	0.87857	0.74821	-0.41848	0.025241	0.26272	0.73926	-0.43584
TAG504-FA182	0.10163	0.70319	1.1717	0.2286	0.67499	0.96398	0.85966	-0.21816	0.006394	0.062783	0.46389	-1.1081	0.13338	0.80329	0.59097	-0.75884	0.08327	0.34394	0.69634	-0.52214
TAG491-FA160	0.72801	0.99234	1.0175	0.024967	0.42321	0.92238	0.89389	-0.16183	0.003812	0.052257	0.55298	-0.85471	0.65193	0.90034	1.0865	0.1197	0.072401	0.33329	0.85075	-0.23319
TAG555-FA182	0.42859	0.92598	0.92681	-0.10965	0.29863	0.92112	1.1556	0.20864	0.003335	0.047239	1.5939	0.67258	0.56435	0.89473	0.96836	-0.04638	0.030421	0.26735	1.1774	0.23561
TAG545-FA180	0.80979	0.99234	1.0084	0.012091	0.087973	0.92112	1.4373	0.52335	0.79823	0.87142	0.99431	-0.00824	0.65186	0.90034	1.0613	0.085882	0.021729	0.26022	1.7242	0.78591
TAG524-FA180	0.28021	0.9254	0.79201	-0.3364	0.63737	0.96398	1.1152	0.1573	0.020462	0.10105	1.6995	0.76509	0.87302	0.97384	1.0172	0.024668	0.74449	0.87165	1.1532	0.20566
TAG547-FA182	0.48801	0.94275	1.1112	0.15213	0.35946	0.92112	1.2681	0.34265	0.007155	0.062783	2.1336	1.0933	0.44785	0.87149	1.5486	0.63095	8.84E-05	0.037579	1.9057	0.93036
TAG563-FA182	0.43575	0.92598	0.86122	-0.21555	0.26938	0.92112	0.688	-0.53953	0.33596	0.56885	0.96701	-0.04839	0.008797	0.80329	0.32925	-1.6027	0.8922	0.94892	0.74103	-0.4324
TAG587-FA182	0.91909	0.99234	1.0506	0.071168	0.73282	0.96398	0.83477	-0.26055	0.006324	0.062783	1.7736	0.8267	0.19474	0.80329	0.72291	-0.4681	0.046593	0.29047	0.61112	-0.71046
TAG546-FA182	0.39397	0.92598	1.1385	0.18711	0.34188	0.92112	1.287	0.36403	0.00178	0.035955	2.2645	1.1792	0.86648	0.97346	1.3738	0.45813	0.000993	0.096167	1.7034	0.76846

TAG563-FA201	0.076603	0.67266	0.73896	-0.43644	0.19351	0.92112	0.73015	-0.45374	0.42	0.62631	1.0784	0.10895	0.016958	0.80329	0.52875	-0.91934	0.53879	0.74054	0.65778	-0.60433
TAG482-FA161	0.94473	0.99234	1.0048	0.006848	0.97763	0.99639	1.0034	0.004873	0.000921	0.034775	0.4115	-1.281	0.66026	0.90034	1.1187	0.1618	0.35976	0.58919	1.0522	0.073447
TAG561-FA181	0.21035	0.86797	0.89594	-0.15853	0.20303	0.92112	0.89893	-0.15372	0.007234	0.062783	1.3749	0.45938	0.063349	0.80329	0.83165	-0.26595	0.34688	0.57989	1.086	0.11898
DAG180/181	0.39378	0.92598	0.90601	-0.14241	0.23712	0.92112	1.2453	0.31651	0.002221	0.036301	1.8389	0.87884	0.55486	0.89473	0.97074	-0.04284	0.03541	0.26873	1.2147	0.28064
TAG534-FA160	0.59759	0.9647	1.1662	0.22186	0.71098	0.96398	1.0243	0.034569	0.14184	0.34126	1.2723	0.34749	0.003111	0.80329	0.62719	-0.67301	0.93567	0.96751	0.96532	-0.05092
FFA202	0.96813	0.99386	0.99542	-0.00663	0.39598	0.92112	1.1703	0.22693	0.011668	0.078206	1.8895	0.91797	0.53695	0.89334	0.89144	-0.16579	0.96204	0.98058	0.99728	-0.00393
TAG543-FA183	0.25953	0.9166	1.1342	0.18169	0.52151	0.96398	1.3205	0.40111	0.020686	0.10105	1.7908	0.84058	0.50476	0.88646	0.82433	-0.2787	0.33532	0.57989	1.0612	0.085744
TAG522-FA181	0.96131	0.99234	1.0174	0.024866	0.095898	0.92112	0.61277	-0.70658	0.62849	0.79261	0.31644	-1.66	0.23267	0.80329	0.69644	-0.52192	0.026677	0.26272	0.45836	-1.1254
TAG503-FA140	0.65354	0.98776	0.96605	-0.04983	0.045355	0.92112	0.67282	-0.5717	0.3639	0.58809	0.36896	-1.4385	0.32053	0.85641	0.64802	-0.6259	0.073224	0.33329	0.53306	-0.90762
TAG541-FA180	0.51512	0.94319	0.92579	-0.11124	0.057131	0.92112	0.53733	-0.89612	0.05133	0.18271	1.8964	0.92324	0.33277	0.85641	0.73411	-0.44594	0.008056	0.1261	0.39852	-1.3273
TAG484-FA182	0.38058	0.92598	0.8373	-0.25619	0.82165	0.9749	0.97048	-0.04323	0.002352	0.03702	0.45147	-1.1473	0.9685	0.99429	0.97975	-0.02952	0.093559	0.35502	1.5484	0.6308
TAG541-FA200	0.55359	0.94319	0.97238	-0.04041	0.73852	0.96398	0.82258	-0.28177	0.4447	0.64948	0.5134	-0.96185	0.24178	0.80329	0.70952	-0.49508	0.044217	0.29047	0.46003	-1.1202
TAG589-FA181	0.87201	0.99234	1.194	0.25585	0.30213	0.92112	1.2943	0.37214	0.73659	0.85584	1.1567	0.20996	0.29659	0.82929	0.79788	-0.32575	0.028435	0.26272	0.59051	-0.75997
TAG531-FA180	0.30289	0.9255	0.9415	-0.08697	0.84025	0.97588	0.98616	-0.02011	0.29391	0.53841	1.2422	0.31287	0.46237	0.8754	0.95634	-0.0644	0.031453	0.26735	0.77761	-0.36287
TAG500-FA180	0.006078	0.42014	0.86317	-0.21229	0.45448	0.93084	1.1364	0.18452	0.016742	0.090065	1.6122	0.68906	0.2717	0.80751	0.83566	-0.25902	0.20386	0.48949	0.88281	-0.17982
TAG542-FA180	0.77072	0.99234	0.9585	-0.06116	0.23161	0.92112	0.59484	-0.74943	0.46731	0.67553	1.0519	0.073049	0.20741	0.80329	0.63983	-0.64424	0.039646	0.2813	0.39591	-1.3368
TAG483-FA160	0.72415	0.99234	0.97351	-0.03873	0.82427	0.9749	0.90318	-0.14691	0.01844	0.094674	0.46778	-1.0961	0.44908	0.87149	0.76779	-0.38121	0.91457	0.95784	0.95582	-0.06519
TAG542-FA181	0.82707	0.99234	0.98907	-0.01586	0.12071	0.92112	0.58357	-0.77701	0.74394	0.85584	0.83847	-0.25417	0.25663	0.80329	0.66854	-0.58091	0.033482	0.26873	0.41958	-1.253
SM241	0.44447	0.92598	0.74561	-0.4235	0.002567	0.92112	1.6029	0.68071	0.063017	0.2029	1.3638	0.44765	0.9511	0.99024	1.1289	0.17488	0.86787	0.93681	1.092	0.12695
DAG181/182	0.52274	0.94319	1.056	0.078661	0.21341	0.92112	1.174	0.23149	0.01359	0.0835	1.4798	0.56538	0.57262	0.89473	0.96239	-0.05531	0.025411	0.26272	1.2236	0.29109
SM220	0.48676	0.94275	1.1078	0.14772	0.091906	0.92112	1.5475	0.6299	0.076406	0.23195	1.7002	0.76567	0.73087	0.93436	1.0035	0.004998	0.034601	0.26873	1.7012	0.76654
DAG180/182	0.076903	0.67266	1.328	0.4093	0.20256	0.92112	1.3364	0.41838	0.000198	0.034775	2.6516	1.4069	0.53461	0.89334	1.0804	0.1115	0.047909	0.29087	1.3912	0.47635
TAG545-FA183	0.39959	0.92598	1.1009	0.1387	0.69833	0.96398	1.1227	0.16699	0.5807	0.76365	0.67948	-0.5575	0.66511	0.90034	1.1656	0.22107	0.008308	0.1261	1.384	0.46886
TAG565-FA202	0.65405	0.98776	0.88238	-0.18052	0.42939	0.92414	1.2167	0.283	0.007386	0.062783	1.8278	0.87009	0.19602	0.80329	0.84806	-0.23776	0.41942	0.6412	0.85687	-0.22285
ANTHRANILIC ACID_pos_4	0.5369	0.94319	0.96264	-0.05493	0.61153	0.96398	1.159	0.21293	0.013071	0.081695	0.49703	-1.0086	0.97695	0.9957	0.92017	-0.12002	0.7234	0.85879	1.2762	0.35182
TAG546-FA181	0.41189	0.92598	1.0867	0.11989	0.25535	0.92112	1.2996	0.37807	0.24662	0.4875	1.0524	0.073656	0.99581	0.99678	1.1278	0.17346	0.002839	0.096167	1.4831	0.56858
TAG481-FA160	0.75764	0.99234	1.0159	0.02279	0.092836	0.92112	0.84162	-0.24875	0.006095	0.062783	0.53072	-0.91397	0.99163	0.99678	1.0042	0.00608	0.026777	0.26272	0.8827	-0.18001
TAG563-FA181	0.19288	0.83997	0.87684	-0.18962	0.86823	0.98395	0.99448	-0.00799	0.00126	0.034775	1.7297	0.79053	0.11167	0.80329	0.84111	-0.24963	0.54875	0.74747	1.0323	0.045843
TAG462-FA120	0.54024	0.94319	0.88215	-0.18091	0.10755	0.92112	0.71864	-0.47666	0.044524	0.16895	0.52444	-0.93116	0.97267	0.9957	1.0054	0.007736	0.15291	0.42754	1.1265	0.1719
TAG543-FA202	0.66652	0.99046	0.87071	-0.19973	0.39955	0.92112	1.2341	0.30351	0.030059	0.13036	1.6792	0.74775	0.43332	0.87102	0.90293	-0.14731	0.26915	0.52713	0.83399	-0.26191
DAG160/160	0.056334	0.61472	0.8118	-0.3008	0.30229	0.92112	1.3416	0.424	0.048471	0.17913	1.6633	0.73406	0.40221	0.87102	0.82584	-0.27607	0.1941	0.47815	1.1122	0.15343
TAG512-FA160	0.28589	0.9254	0.89169	-0.16538	0.26531	0.92112	1.116	0.15829	0.011239	0.078206	1.5557	0.63758	0.42138	0.87102	0.92175	-0.11755	0.40174	0.63004	1.0638	0.089171
TAG522-FA180	0.98639	0.99794	0.9682	-0.04662	0.45606	0.93084	0.62082	-0.68776	0.64237	0.79991	1.0263	0.037505	0.1302	0.80329	0.48287	-1.0503	0.039713	0.2813	0.37109	-1.4302
TAG542-FA182	0.9909	0.99794	0.96936	-0.04489	0.44992	0.93084	1.0999	0.13741	0.013832	0.0835	1.5927	0.67148	0.065674	0.80329	0.6924	-0.53031	0.047159	0.29047	0.61745	-0.6956
TAG492-FA181	0.71134	0.99234	0.97761	-0.03267	0.95899	0.99206	0.99942	-0.00083	0.001142	0.034775	0.49942	-1.0017	0.65756	0.90034	1.0965	0.13291	0.24474	0.51246	0.92644	-0.11023
TAG503-FA161	0.94711	0.99234	0.99552	-0.00648	0.8394	0.97588	0.91657	-0.12568	0.000909	0.034775	0.36779	-1.443	0.30317	0.83668	0.62246	-0.68394	0.045277	0.29047	0.72834	-0.45733
DAG161/182	0.23416	0.89656	1.3298	0.41126	0.37333	0.92112	1.5964	0.67478	0.74574	0.85584	1.3344	0.41623	0.034131	0.80329	1.6543	0.72624	0.26655	0.52446	1.408	0.49367
ADENOSINE PHOSPHOSULFATE_neg	0.72995	0.99234	1.3229	0.40374	0.12605	0.92112	4.9164	2.2976	0.007579	0.063157	6.4435	2.6879	0.62387	0.90034	0.60079	-0.73507	0.16095	0.43848	2.7473	1.458
TAG500-FA160	0.006264	0.42014	0.85646	-0.22354	0.50208	0.96398	1.1425	0.19217	0.052499	0.18271	1.4733	0.55905	0.16937	0.80329	0.80124	-0.31968	0.082273	0.34394	0.83614	-0.25818
CE150	0.020825	0.42014	1.7641	0.81894	0.42001	0.92238	1.3292	0.41058	0.042946	0.16745	0.46112	-1.1168	0.021735	0.80329	1.4875	0.57293	0.96212	0.98058	0.85907	-0.21915
TAG492-FA150	0.69156	0.99234	0.97419	-0.03772	0.42603	0.92378	0.79794	-0.32564	0.004368	0.053283	0.53312	-0.90746	0.91146	0.97744	0.94014	-0.08906	0.002365	0.096167	0.66411	-0.5905
CER160	0.2303	0.89656	1.1297	0.17589	0.05613	0.92112	0.49731	-1.0078	0.041541	0.16646	2.6512	1.4066	0.056374	0.80329	0.56398	-0.82629	0.074951	0.33329	0.27215	-1.8775
CER240	0.60385	0.96479	0.5877	-0.76685	0.39372	0.92112	0.37599	-1.4112	0.75516	0.86023	2.6391	1.4001	0.1463	0.80329	0.40361	-1.309	0.013154	0.16941	0.096508	-3.3732
1-METHYLADENOSINE_pos_1	0.9601	0.99234	1.5179	0.60209	0.18938	0.92112	1.3869	0.4719	0.20248	0.42183	0.69368	-0.52765	0.66785	0.90034	0.99142	-0.01243	0.04316	0.29047	8.0461	3.0083
TAG492-FA161	0.93345	0.99234	1.0005	0.000655	0.80291	0.9749	1.0431	0.060841	0.029292	0.12834	0.42433	-1.2367	0.21693	0.80329	1.2718	0.34689	0.58544	0.76619	1.0199	0.028387
TAG482-FA182	0.94531	0.99234	1.0074	0.010586	0.14565	0.92112	0.87472	-0.1931	0.043422	0.16777	0.56226	-0.83069	0.77581	0.95689	0.95868	-0.06088	0.037203	0.27739	0.87369	-0.1948
TAG522-FA160	0.83203	0.99234	0.98362	-0.02382	0.14821	0.92112	0.61881	-0.69243	0.81837	0.88346	0.35566	-1.4914	0.25167	0.80329	0.67919	-0.55811	0.038301	0.28065	0.44512	-1.1677
TAG483-FA140	0.32402	0.9255	0.80508	-0.3128	0.094476	0.92112	0.71022	-0.49367	0.009343	0.067297	0.40363	-1.3089	0.91144	0.97744	0.82932	-0.26999	0.32832	0.57442	0.7106	-0.49289
TAG564-FA202	0.5512	0.94319	0.85212	-0.23087	0.46142	0.93084	1.2077	0.27227	0.008964	0.065952	1.825	0.86789	0.33248	0.85641	0.88579	-0.17496	0.35265	0.58546	0.84892	-0.23631
TAG503-FA182	0.58483	0.95419	0.97573	-0.03545	0.050442	0.92112	0.67759	-0.56152	0.033864	0.14392	0.39242	-1.3495	0.21027	0.80329	0.56602	-0.82109	0.004666	0.098784	0.5313	-0.91241
TAG545-FA182	0.53941	0.94319	1.0765	0.10639	0.50907	0.96398	1.1351	0.18284	0.039471	0.1613	1.4209	0.50684	0.4359	0.87102	0.81789	-0.29002	0.25963	0.52049	1.1288	0.17484
TAG491-FA140	0.13452	0.74113	1.1414	0.1908	0.24037	0.92112	1.1047	0.14361	0.014231	0.0835	0.61543	-0.70034	0.11473	0.80329	1.2764	0.35212	0.67353	0.82465	0.96009	-0.05875
TAG503-FA160	0.3693	0.92598	1.0368	0.052076	0.14593	0.92112	0.63497	-0.65523	0.11605	0.30259	0.39848	-1.3274	0.03825	0.80329	0.35567	-1.4914	0.035315	0.26873	0.46532	-1.1037
FFA203	0.42674	0.92598	1.1101	0.15075	0.38065	0.92112	1.2002	0.2633	0.006992	0.062783	1.8844	0.91413	0.39262	0.87102	0.90173	-0.14923	0.65583	0.81862	1.0327	0.046374
TAG563-FA202	0.6343	0.98776	0.8571	-0.22246	0.36729	0.92112	1.2511	0.32315	0.014813	0.083943	1.8392	0.8791	0.46911	0.87451	0.90083	-0.15067	0.41859	0.6412	0.86585	-0.20781
TAG542-FA160	0.78845	0.99234	0.97455	-0.0372	0.31165	0.92112	0.7149	-0.48418	0.83859	0.891	0.83971	-0.25204	0.037258	0.80329	0.51365	-0.96115	0.27638	0.53881	0.6326	-0.66063
TAG491-FA161	0.84538	0.99234	0.98666	-0.01937	0.96126	0.99206	1.0139	0.019954	0.044041	0.16863	0.40953	-1.288	0.1906	0.80329	1.2944	0.37232	0.65116	0.81862	0.99911	-0.00129
TAG493-FA182	0.93245	0.99234	0.99392	-0.0088	0.21138	0.92112	0.8555	-0.22516	0.008923	0.065952	0.50841	-0.97595	0.37343	0.86725	1.1256	0.17074	0.9402	0.96751	0.93549	-0.09621
TAG512-FA170	0.8144	0.99234	0.95889	-0.06057	0.36053	0.92112	0.81557	-0.29412	0.012145	0.078206	0.5544	-0.851	0.23746	0.80329	1.2075	0.27199	0.03102	0.26735	0.6853	-0.54518
CER220	0.97062	0.99401	0.81463	-0.29578	0.34412	0.92112	1.0074	0.010688	0.008879	0.065952	3.7927	1.9232	0.29187	0.82697	0.65167	-0.61778	0.003484	0.096167	0.12673	-2.9801
TAG555-FA181	0.34561	0.92598	0.8953	-0.15956	0.21907	0.92112	1.1411	0.1904	0.005434	0.057734	1.672	0.74159	0.43359	0.87102	0.93154	-0.10232	0.39805	0.62889	1.0662	0.092533
TAG504-FA161	0.045428	0.60334	0.7573	-0.40107	0.69144	0.96398	1.0246	0.035038	0.009765	0.069172	0.51541	-0.95622	0.42663	0.87102	0.54238	-0.88262	0.62273	0.79103	0.78884	-0.34219
TAG534-FA182	0.21997	0.87371	0.87316	-0.19568	0.36717	0.92112	1.0967	0.13312	0.004108	0.053283	1.7581	0.81399	0.3365	0.85641	0.89077	-0.16687	0.25462	0.5191	1.0975	0.13416
LACTATE_neg_1	0.74388	0.99234	1.0475	0.066955	0.80976	0.9749	0.60128	-0.7339	0.016341	0.089035	2.9792	1.5749	0.2621	0.80329	0.66409	-0.59055	0.23594	0.51246	2.4709	1.3051
SM180	0.31151	0.9255	0.88747	-0.17223	0.26685	0.92112	1.2017	0.26506	0.74697	0.85584	1.0569	0.079813	0.04019	0.80329	0.67896	-0.5586	0.15945	0.43719	1.1314	0.17812
TAG546-FA226	0.27078	0.9166	1.5156	0.59991	0.81652	0.9749	0.96184	-0.05614	0.017421	0.092552	1.7113	0.77506	0.5139	0.89147	0.862	-0.21423	0.75591	0.87777	0.9227	-0.11607
TAG525-FA205	0.16314	0.8062	0.7715	-0.37426	0.010437	0.92112	1.9158	0.93791	0.007994	0.065332	2.5625	1.3576	0.80187	0.95886	0.95187	-0.07116	0.049199	0.29221	1.289	0.36628
BETAINE_pos_2	0.007406	0.42014	4.8523	2.2787	0.60523	0.96398	1.1577	0.21129	0.78017	0.86714	1.036	0.051082	0.5969	0.90034	0.66887	-0.58021	0.77109	0.88507	2.11	1.0772
TAG481-FA140	0.32406	0.9255	1.0603	0.084499	0.097134	0.92112	0.81939	-0.28738	0.087604	0.25328	0.60702	-0.72017	0.83857	0.96583	1.0816	0.11318	0.012883	0.16941	0.83789	-0.25511
TAG524-FA204	0.054081	0.61472	0.59249	-0.75514	0.10266	0.92112	1.2905	0.36793	0.004764	0.053283	2.054	1.0384	0.016885	0.80329	0.35583	-1.4907	0.68661	0.82933	0.9287	-0.10671
TAG513-FA182	0.93619	0.99234	0.903	-0.1472	0.16667	0.92112	0.609	-0.71549	0.16319	0.36503	0.64237	-0.63852	0.57728	0.8987	0.68395	-0.54804	0.004731	0.098784	0.42901	-1.2209
TAG481-FA161	0.87543	0.99234	0.99272	-0.01055	0.99785	0.99785	1.0241	0.034326	0.001135	0.034775	0.49844	-1.0045	0.48883	0.8803	1.1234	0.16782	0.87684	0.93869	0.97389	-0.03817
TAG492-FA140	0.41939	0.92598	1.2587	0.33191	0.70811	0.96398	1.0422	0.059647	0.012101	0.078206	0.44517	-1.1676	0.54181	0.89334	1.0528	0.074256	0.17973	0.4574	1.1429	0.19267
FFA241	0.47308	0.93952	0.9323	-0.10114	0.21399	0.92112	1.2739	0.3493	0.012867	0.081618	1.9197	0.94091	0.18492	0.80329	0.7429	-0.42875	0.871	0.93715	1.0382	0.054134
O-ACETYLSERINE_pos_3	0.030801	0.51706	3.5665	1.8345	0.28524	0.92112	0.84316	-0.24612	0.59905	0.77209	1.1073	0.14702	0.41825	0.87102	0.8937	-0.16214	0.93315	0.96751	1.1696	0.22602
TAG524-FA181	0.87726	0.99234	0.98061	-0.02825	0.90335	0.98395	0.82243	-0.28204	0.74752	0.85584	0.46318	-1.1104	0.051163	0.80329	0.39994	-1.3222	0.049977	0.29221	0.50624	-0.9821
TAG502-FA182	0.14116	0.74113	1.0894	0.12352	0.1319	0.92112	0.72215	-0.46964	0.56831	0.76038	0.65721	-0.60557	0.29434	0.82843	0.75878	-0.39824	0.046951	0.29047	0.63963	-0.64469
TAG524-FA161	0.17484	0.80971	1.0938	0.12931	0.28904	0.92112	0.56808	-0.81583	0.11703	0.30321	0.40893	-1.2901	0.16259	0.80329	0.24846	-2.0089	0.011135	0.15266	0.28999	-1.7859
TAG514-FA182	0.049999	0.61472	0.72034	-0.47325	0.90224	0.98395	0.85316	-0.22911	0.63819	0.79903	1.012	0.017202	0.26047	0.80329	0.70873	-0.49669	0.07296	0.33329	0.57724	-0.79275
TAG502-FA140	0.88991	0.99234	1.0164	0.023479	0.044317	0.92112	0.69062	-0.53403	0.33214	0.56544	0.4911	-1.0259	0.92775	0.98083	0.98838	-0.01687	0.022665	0.26022	0.79381	-0.33313
TAG526-FA181	0.11189	0.70975	0.82802	-0.27227	0.45067	0.93084	1.1135	0.15506	0.034871	0.14673	1.4356	0.52167	0.42519	0.87102	0.9208	-0.11904	0.4558	0.67019	1.0471	0.066424
TAG568-FA204	0.53969	0.94319	0.86858	-0.20326	0.81746	0.9749	0.95719	-0.06312	0.001961	0.035955	1.8234	0.86665	0.10973	0.80329	0.79241	-0.33568	0.99014	0.99505	0.96369	-0.05336
SM240	0.23317	0.89656	0.87495	-0.19273	0.10058	0.92112	1.5287	0.61231	0.026232	0.11988	2.1823	1.1259	0.50097	0.88561	0.90393	-0.14572	0.57054	0.75705	1.0445	0.062821
TAG565-FA225	0.35303	0.92598	0.75501	-0.40543	0.27564	0.92112	0.90298	-0.14723	0.007303	0.062783	1.7849	0.83586	0.1926	0.80329	0.77113	-0.37495	0.19512	0.47815	1.1323	0.17922
TAG503-FA181	0.82385	0.99234	0.99322	-0.00982	0.040778	0.92112	0.82067	-0.28512	0.011952	0.078206	0.49122	-1.0256	0.18424	0.80329	0.75673	-0.40214	0.053557	0.29221	0.83752	-0.25581
TAG491-FA170	0.53819	0.94319	1.0314	0.044588	0.75274	0.96398	0.96317	-0.05414	0.020462	0.10105	0.44673	-1.1625	0.13453	0.80329	1.3018	0.3805	0.40759	0.63371	1.0337	0.047853
L-ORNITHINE_pos_1	0.87887	0.99234	0.75833	-0.39909	0.36718	0.92112	1.8191	0.86322	0.046159	0.17361	1.8326	0.87389	0.8457	0.96667	0.77885	-0.36058	0.91666	0.95784	1.4657	0.55154
UREIDOSUCCINIC ACID_neg_2	0.41234	0.92598	0.43413	-1.2038	0.23999	0.92112	0.53862	-0.89267	0.93789	0.95818	1.6391	0.71295	0.008402	0.80329	0.35935	-1.4765	0.088931	0.34675	2.3738	1.2472

Supplementary Table S3D. List of dysregulated metabolites for APCHi mice following exposure to 9.5 Gy of Gamma-radiation, 24 h, 1 week, 1 month, 3 month and 6 months post-irradiation.

	9.5Gy / 0.0Gy				9.5Gy / 0.0Gy				9.5Gy / 0.0Gy				9.5Gy / 0.0Gy				9.5Gy / 0.0Gy			
	24h				1wk				1mo				3mo				6mo			
	APCHi																			
name	p-value	FDR	FC	LOG2FC	p-value	FDR	FC	LOG2FC	p-value	FDR	FC	LOG2FC	p-value	FDR	FC	LOG2FC	p-value	FDR	FC	LOG2FC
CYSTEINE_neg_5	0.015143	0.52739	2.6491	1.4055	0.095072	0.32496	0.62103	-0.68727	0.22497	0.43658	0.75296	-0.40936	0.018044	0.31888	2.0079	1.0057	0.85023	0.97398	1.2538	0.32626
CE204	0.023941	0.55845	1.9966	0.99755	0.016303	0.14742	2.1295	1.0905	0.46448	0.65295	1.3579	0.44139	0.88722	0.9683	0.96502	-0.05137	0.81997	0.95829	0.91003	-0.13601
OXOGLUTARATE_neg_2	0.006983	0.42218	0.13069	-2.9357	0.12542	0.3727	0.37684	-1.408	0.25439	0.46007	0.89789	-0.15539	0.89145	0.9683	0.9642	-0.05259	0.22693	0.7048	0.5624	-0.83034
CE182	0.18226	0.76702	1.2236	0.29116	0.003508	0.053246	1.5209	0.60496	0.5657	0.72417	1.1416	0.19105	0.15997	0.42629	1.1683	0.2244	0.44662	0.8461	0.92695	-0.10944
NORMETANEPHRINE_pos_1	0.018711	0.55845	2.1597	1.1108	0.055209	0.24671	4.0264	2.0095	0.80817	0.88297	0.73842	-0.43748	0.059336	0.33448	3.0632	1.6151	0.54975	0.86495	1.2774	0.35325
ASCORBATE_neg_2	0.64308	0.96666	1.2149	0.28089	0.47417	0.74005	0.92789	-0.10797	0.00514	0.16803	0.47959	-1.0601	0.66684	0.8432	0.82183	-0.28308	0.87338	0.98391	0.90664	-0.1414
AMINOADIPATE_pos_1	0.11708	0.7065	1.4523	0.53838	0.20931	0.47423	0.50501	-0.9856	0.10837	0.34512	0.67133	-0.5749	0.25156	0.49726	3.1215	1.6422	0.036019	0.56697	0.42531	-1.2334
CE226	0.001499	0.21242	3.1033	1.6338	0.21978	0.48649	1.5767	0.65687	0.86081	0.91462	1.0852	0.11801	0.004002	0.28348	2.9239	1.5479	0.14288	0.70044	1.5083	0.59296
TAG566-FA225	0.16656	0.73306	1.3648	0.4487	0.52519	0.76441	1.1634	0.21829	0.039781	0.26836	0.62067	-0.6881	0.91138	0.97812	1.3373	0.41927	0.77536	0.94214	0.84067	-0.25039
TAG544-FA224	0.03792	0.55845	1.598	0.6763	0.66688	0.8694	1.4033	0.48879	0.043448	0.27978	0.57725	-0.79274	0.17017	0.43582	1.5438	0.62649	0.5933	0.87674	1.1322	0.17918
ACETYLPHOSPHATE_pos_1	0.46494	0.91799	0.90115	-0.15017	0.039781	0.21415	0.60095	-0.73468	0.089203	0.32588	0.91799	-0.12346	0.89539	0.9683	1.0725	0.10091	0.18718	0.70044	0.73721	-0.43985
TAG522-FA202	0.063835	0.58978	1.5899	0.66893	0.9578	0.99043	1.2392	0.30943	0.068361	0.30582	0.57663	-0.79428	0.026206	0.33448	1.8093	0.85544	0.60197	0.87674	0.95314	-0.06924
TAG565-FA181	0.74731	0.96748	2.0094	1.0068	0.053927	0.24671	0.49395	-1.0176	0.097123	0.33288	0.45883	-1.124	0.042853	0.33448	0.24502	-2.029	0.077052	0.67237	0.23227	-2.1061
TAG483-FA181	0.048825	0.55845	0.78731	-0.34499	0.58562	0.80546	1.073	0.10162	0.22199	0.43658	1.1765	0.23444	0.35957	0.62121	0.94746	-0.07786	0.99249	0.99956	1.0016	0.002274
TAG501-FA140	0.66174	0.96666	0.97039	-0.04337	0.45992	0.73484	1.4393	0.5254	0.032673	0.25181	1.5252	0.60904	0.19022	0.44626	0.83562	-0.25908	0.81635	0.95829	1.0004	0.000522
ATROLACTIC ACID_neg_2	0.85966	0.99393	1.0023	0.003249	0.070973	0.27795	1.5417	0.62452	0.020336	0.22709	1.6913	0.75809	0.21097	0.46699	1.8881	0.91693	0.084279	0.6773	2.4522	1.2941
TAG582-FA181	0.49982	0.92681	1.0997	0.13708	0.35905	0.62397	0.67686	-0.56308	0.42659	0.62063	0.65551	-0.60931	0.012692	0.31888	0.31193	-1.6807	0.18527	0.70044	0.46286	-1.1113
TAG527-FA160	0.92005	0.99393	0.99907	-0.00134	0.67556	0.87195	1.1168	0.15938	0.25048	0.45886	0.78301	-0.3529	0.12886	0.4002	1.2448	0.31594	0.040443	0.57294	1.1284	0.17429
TAG483-FA182	0.069829	0.59484	0.85193	-0.2312	0.39148	0.66818	0.82584	-0.27607	0.004866	0.16803	1.4046	0.49012	0.15065	0.42122	0.85005	-0.23439	0.072993	0.67237	0.87519	-0.19233
TAG521-FA201	0.48599	0.91799	1.27	0.34484	0.29374	0.55697	0.90031	-0.15151	0.023405	0.22824	0.64953	-0.62254	0.67059	0.8432	1.1001	0.13762	0.63402	0.87674	0.83319	-0.26327
TAG568-FA205	0.89257	0.99393	0.76414	-0.3881	0.96423	0.99053	1.2898	0.36716	0.59985	0.74436	0.65351	-0.61373	0.017164	0.31888	0.61947	-0.6909	0.72787	0.93556	0.89088	-0.16669
TAG521-FA161	0.052104	0.55845	1.3814	0.46612	0.46837	0.73973	0.95807	-0.0618	0.021283	0.22709	0.64927	-0.62311	0.11006	0.38027	1.3306	0.41207	0.98441	0.99956	1.2011	0.26431
CE180	0.64784	0.96666	1.1518	0.20385	8.5E-05	0.01386	1.8644	0.89872	0.92949	0.9496	0.99641	-0.00519	0.32667	0.58581	1.1544	0.20719	0.45988	0.8461	1.1093	0.14959
TAG511-FA170	0.043903	0.55845	1.3645	0.44841	0.47711	0.74005	0.91819	-0.12314	0.061185	0.30521	0.83434	-0.2613	0.49398	0.74904	1.0686	0.09578	0.68188	0.90737	1.0895	0.12371
TAG442-FA182	0.006022	0.42218	0.65944	-0.60069	0.055223	0.24671	1.6758	0.74484	0.015674	0.22433	1.6437	0.71693	0.63511	0.8432	1.0929	0.1281	0.578	0.87674	0.97364	-0.03853
ADP_pos_1	0.68318	0.96666	1.0208	0.029751	0.51959	0.75884	1.0665	0.092893	0.002066	0.16803	2.6607	1.4118	0.36636	0.62784	1.226	0.29394	0.57654	0.87674	1.233	0.30214
TAG564-FA204	0.12303	0.7065	1.3205	0.40104	0.72393	0.87958	1.5727	0.65321	0.036086	0.25181	0.46537	-1.1035	0.54693	0.79333	1.3426	0.42499	0.20489	0.70044	0.75052	-0.41403
CE181	0.34179	0.90224	1.0917	0.12659	0.000234	0.019885	1.5125	0.59692	0.54387	0.70497	1.1882	0.24878	0.96263	0.98693	0.88535	-0.17567	0.062988	0.63737	0.84627	-0.24081
FFA201	0.59026	0.96666	1.3905	0.47556	0.17506	0.4276	1.3717	0.45592	0.15546	0.38012	0.75513	-0.4052	0.01379	0.31888	1.4531	0.53912	0.27117	0.75033	1.2715	0.34657
TAG442-FA120	0.014653	0.52739	0.67664	-0.56353	0.27235	0.53757	0.65592	-0.60842	0.20217	0.4111	1.2276	0.29583	0.96723	0.98693	1.0007	0.001045	0.6035	0.87674	0.93329	-0.09961
IMIDAZOLE_pos_1	0.69048	0.96666	1.0693	0.096619	0.35009	0.62218	1.2107	0.27583	0.038326	0.26272	1.6514	0.72365	0.66492	0.8432	1.2851	0.36186	0.57364	0.87674	0.95826	-0.06151
TAG460-FA140	0.89583	0.99393	1.0507	0.071412	0.3096	0.57388	1.0959	0.13215	0.11069	0.34512	1.1939	0.25566	0.44423	0.7078	1.031	0.044019	0.048083	0.59265	1.2577	0.3308
FFA182	0.43784	0.90782	1.5	0.58496	0.37305	0.64189	1.1336	0.18094	0.40762	0.61605	0.85832	-0.22042	0.005577	0.31888	1.4559	0.54187	0.21893	0.7048	1.3159	0.39602
TAG421-FA160	0.24584	0.80776	0.87495	-0.19273	0.00116	0.043099	2.0637	1.0453	0.053324	0.29916	1.6072	0.68453	0.86632	0.96795	0.95958	-0.05953	0.52338	0.86495	1.1818	0.241
TAG442-FA181	0.041969	0.55845	0.75285	-0.40957	0.006836	0.085445	2.3279	1.219	0.016784	0.22709	1.8858	0.91516	0.73941	0.89321	1.0163	0.023349	0.097521	0.6849	1.3268	0.40792
MEVALONOLACTONE_pos_2	0.48281	0.91799	0.94683	-0.07882	0.090092	0.31385	1.3395	0.42164	0.044569	0.28157	1.6942	0.7606	0.49724	0.74904	1.4534	0.53943	0.95922	0.99778	1.0314	0.044555
TAG567-FA226	0.001346	0.21242	2.8012	1.486	0.98383	0.99053	0.95209	-0.07083	0.19121	0.41042	0.64889	-0.62395	0.62121	0.84081	0.83996	-0.2516	0.94872	0.99778	0.71857	-0.47679
TAG505-FA205	0.73992	0.96748	1.0604	0.084608	0.002169	0.046168	1.69	0.75704	0.60425	0.74436	0.93587	-0.09562	0.63696	0.8432	1.1043	0.14311	0.87476	0.98391	1.0308	0.043714
CE160	0.90334	0.99393	1.0067	0.00959	6.72E-05	0.01386	1.8487	0.88651	0.54407	0.70497	1.1038	0.14247	0.43352	0.70056	1.1045	0.14334	0.84575	0.97398	1.0642	0.089732
ARGININOSUCCINIC ACID_neg_2	0.48408	0.91799	0.86284	-0.21283	0.32681	0.59143	0.7726	-0.37221	0.021275	0.22709	0.48504	-1.0438	0.37126	0.63368	1.4527	0.53878	0.19633	0.70044	0.82093	-0.28467
TAG480-FA140	0.35947	0.90782	1.1614	0.21585	0.010081	0.11275	1.2724	0.34755	0.41044	0.61605	1.0883	0.12209	0.66916	0.8432	1.0241	0.0344	0.38783	0.84095	1.1287	0.17464
FFA141	0.23736	0.80776	1.6736	0.74296	0.12042	0.37084	1.5135	0.5979	0.1355	0.36406	0.71697	-0.48001	0.018246	0.31888	1.5035	0.5883	0.38204	0.83695	1.3012	0.37988
TAG482-FA140	0.15294	0.7065	0.89619	-0.15812	0.74303	0.89458	0.84783	-0.23816	0.013453	0.21636	1.3345	0.41625	0.037483	0.33448	0.75235	-0.41053	0.24957	0.73895	0.89948	-0.15283
INDOLE-3-CARBOXYLIC ACID_pos_3	0.16852	0.73306	2.9367	1.5542	0.009306	0.10952	2.1891	1.1303	0.61206	0.74964	0.90385	-0.14585	0.65289	0.8432	1.0848	0.11749	0.07556	0.67237	0.4813	-1.055
PHENYLPROPIOLIC ACID_neg_1	0.004664	0.42218	0.12578	-2.9911	0.24302	0.50707	0.46372	-1.1087	0.85613	0.91191	1.0562	0.07884	0.1886	0.44626	0.64153	-0.6404	0.57871	0.87674	0.7945	-0.33187
TAG482-FA141	0.029949	0.55845	0.77834	-0.36152	0.48276	0.74076	0.85497	-0.22606	0.013341	0.21636	1.3724	0.45672	0.10412	0.38027	0.84587	-0.24149	0.49421	0.8639	0.94732	-0.07808
TAG568-FA160	0.12039	0.7065	0.63951	-0.64497	0.019451	0.15598	0.58896	-0.76375	0.4184	0.61605	0.84431	-0.24416	0.32563	0.58581	0.83577	-0.25882	0.73346	0.93556	0.90428	-0.14515
TAG562-FA180	0.55613	0.96666	1.0594	0.083228	0.2394	0.50707	0.8173	-0.29106	0.042297	0.27926	0.62181	-0.68546	0.097851	0.37806	0.60708	-0.72004	0.2272	0.7048	0.65911	-0.60142
TAG461-FA181	0.70693	0.96666	1.0229	0.032662	0.089549	0.31385	1.441	0.52704	0.008292	0.21636	1.6365	0.71059	0.9449	0.98186	0.97368	-0.03849	0.088146	0.68076	1.3621	0.4458
FFA181	0.63872	0.96666	1.2361	0.30583	0.28265	0.54602	1.1915	0.25273	0.057861	0.30521	0.76873	-0.37945	0.017773	0.31888	1.4212	0.50709	0.52315	0.86495	1.1783	0.23676
TAG441-FA160	0.10313	0.69575	0.86079	-0.21626	0.059107	0.25633	1.3809	0.46557	0.019462	0.22709	1.4253	0.51122	0.87437	0.96795	0.97648	-0.03434	0.55117	0.86495	1.1013	0.13924
TAG462-FA181	0.19693	0.77762	0.87841	-0.18703	0.015138	0.13987	1.5591	0.64071	0.024603	0.22824	1.5518	0.63396	0.79525	0.93624	0.95907	-0.06029	0.63739	0.87674	1.2892	0.36651
TAG461-FA140	0.80042	0.98603	1.018	0.025782	0.4302	0.70866	0.76197	-0.39219	0.007165	0.20301	1.4581	0.54406	0.63526	0.8432	0.95152	-0.07169	0.13992	0.70044	1.1538	0.20634
TAG441-FA140	0.13839	0.7065	0.84213	-0.24789	0.40384	0.67838	0.76912	-0.37872	0.047972	0.29114	1.4095	0.49517	0.52419	0.78169	1.05	0.070445	0.37501	0.83444	1.1036	0.14221
TAG461-FA120	0.19761	0.77762	0.86843	-0.20352	0.14663	0.40251	1.3133	0.39325	0.015734	0.22433	1.5204	0.60445	0.92678	0.98177	1.0006	0.000825	0.67358	0.90122	1.09	0.12438
TAG513-FA150	0.92999	0.99393	1.3198	0.40029	0.32179	0.58695	0.72926	-0.4555	0.59102	0.74436	0.616	-0.699	0.30074	0.56556	0.42923	-1.2202	0.022643	0.5398	0.29529	-1.7598
CHOLINE_pos_1	0.040165	0.55845	0.44975	-1.1528	0.048475	0.23411	0.60267	-0.73055	0.47277	0.65663	1.2824	0.35881	0.6756	0.8464	0.89761	-0.15584	0.033379	0.56697	1.6611	0.73217
TAG440-FA160	0.97026	0.99393	1.0824	0.11425	0.028798	0.1974	1.266	0.34031	0.080834	0.32588	1.2627	0.33648	0.31956	0.5835	1.0486	0.068431	0.036016	0.56697	1.3177	0.39802
TAG541-FA160	0.59844	0.96666	0.96924	-0.04508	0.011139	0.12139	0.63699	-0.65065	0.49215	0.67255	0.8291	-0.27037	0.050692	0.33448	0.60624	-0.72204	0.26482	0.74045	0.71841	-0.47711
METHYL PHENYLACETATE_neg_3	0.000577	0.21242	0.48516	-1.0435	0.71884	0.879	0.92448	-0.11328	0.13696	0.36406	1.3575	0.441	0.61345	0.83816	1.8916	0.91961	0.16952	0.70044	0.7062	-0.50185
TAG440-FA120	0.24559	0.80776	0.88875	-0.17016	0.028415	0.1974	1.2763	0.35192	0.1979	0.41083	1.1974	0.25992	0.24003	0.48346	1.0984	0.1354	0.010686	0.5398	1.3863	0.47121
TAG501-FA181	0.009319	0.42218	1.4434	0.52951	0.15551	0.40798	1.1877	0.24819	0.2882	0.49042	1.1502	0.20187	0.24811	0.49273	0.85653	-0.22342	0.41692	0.8461	0.91072	-0.13491
FFA205	0.12659	0.7065	1.9653	0.97476	0.062334	0.26492	1.5953	0.67381	0.19404	0.41083	0.78752	-0.34461	0.012472	0.31888	1.559	0.64062	0.21345	0.70044	1.4203	0.5062
TAG440-FA140	0.97756	0.99393	1.0469	0.066122	0.54165	0.77438	1.0419	0.059219	0.11696	0.35093	1.2364	0.30613	0.22153	0.4755	1.0825	0.11438	0.016246	0.5398	1.3407	0.42294
4-IMIDAZOLEACETATE_pos_1	0.16182	0.73162	1.8906	0.91882	0.09787	0.32496	0.66469	-0.58925	0.000904	0.16803	0.49723	-1.008	0.20876	0.46452	0.33065	-1.5966	0.91991	0.9879	1.4028	0.48831
TAG483-FA141	0.035634	0.55845	0.73685	-0.44056	0.21191	0.47423	0.75406	-0.40725	0.51239	0.69352	1.0971	0.13366	0.633	0.8432	0.9172	-0.12469	0.27189	0.75033	0.91811	-0.12326
TAG512-FA161	0.49095	0.92325	0.94554	-0.08079	0.000894	0.042231	0.48903	-1.032	0.7553	0.84697	1.0833	0.11539	0.80513	0.93861	1.0782	0.10859	0.44669	0.8461	1.27	0.34487
DAG140/181	0.97053	0.99393	0.99374	-0.00906	0.021742	0.16217	1.594	0.67261	0.85415	0.91191	0.9594	-0.0598	0.1063	0.38027	1.3713	0.4555	0.92043	0.9879	0.97575	-0.03542
URIDINE 5-DIPHOSPHATE_neg_2	0.96034	0.99393	0.74588	-0.42298	0.40301	0.67838	0.75081	-0.41349	0.029798	0.24396	0.52661	-0.92519	0.79856	0.93701	1.6512	0.72347	0.921	0.9879	1.1442	0.19439
TAG461-FA160	0.96696	0.99393	1.043	0.060741	0.59327	0.80957	0.85155	-0.23184	0.005126	0.16803	1.4423	0.52838	0.73923	0.89321	0.97089	-0.04262	0.14688	0.70044	1.1567	0.21005
TAG525-FA161	0.62347	0.96666	0.77244	-0.37251	0.79927	0.92308	0.84247	-0.24731	0.43814	0.63122	0.79052	-0.33912	0.016136	0.31888	0.23971	-2.0606	0.31312	0.77043	0.25985	-1.9443
4-AMINOBENZOIC ACID_pos_1	0.43402	0.90782	0.57653	-0.79453	0.026412	0.18709	0.51447	-0.95883	0.029062	0.24396	0.57532	-0.79756	0.010325	0.31888	0.45874	-1.1242	0.55728	0.86495	0.92309	-0.11546
TAG525-FA225	0.036992	0.55845	0.77637	-0.36519	0.003001	0.049048	1.5786	0.65869	0.58756	0.74436	0.90772	-0.13968	0.59248	0.83146	1.1001	0.13762	0.5175	0.86495	1.0958	0.13198
DAG181/226	0.12105	0.7065	1.353	0.4362	0.16996	0.42388	1.1728	0.22999	0.53722	0.70323	0.87696	-0.18942	0.023962	0.33448	1.3673	0.45138	0.21364	0.70044	1.1816	0.24074
TAG483-FA161	0.91847	0.99393	1.0364	0.051562	0.041026	0.21415	0.63564	-0.65372	0.54159	0.70497	1.225	0.29282	0.6148	0.83816	0.86703	-0.20585	0.91146	0.9879	1.0748	0.10411
TAG482-FA181	0.13134	0.7065	0.88721	-0.17266	0.44903	0.72014	1.0978	0.13463	0.003703	0.16803	1.4681	0.55393	0.080027	0.34783	0.88709	-0.17285	0.54591	0.86495	0.97528	-0.03611
DAG160/182	0.9741	0.99393	0.68251	-0.55107	0.62396	0.8287	1.1687	0.22485	0.027738	0.24396	0.75479	-0.40586	0.31423	0.58065	1.1186	0.16167	0.80255	0.95275	1.0129	0.018534
TAG522-FA161	0.69882	0.96666	1.0838	0.11606	0.004979	0.067646	0.56886	-0.81386	0.6862	0.80712	0.87162	-0.19823	0.17133	0.43582	0.61001	-0.71309	0.15145	0.70044	0.6428	-0.63755
TAG542-FA202	0.065366	0.59107	4.4539	2.1551	0.007266	0.088233	1.6003	0.67831	0.42435	0.62063	0.91522	-0.12781	0.60433	0.83816	0.99164	-0.01211	0.26258	0.73906	2.0235	1.0169
TAG523-FA161	0.8604	0.99393	1.2171	0.28348	0.001217	0.043099	0.55469	-0.85024	0.74543	0.84228	0.8248	-0.27789	0.12497	0.4002	0.46688	-1.0989	0.047097	0.59265	0.50492	-0.98588
TAG589-FA226	0.50635	0.92681	0.99097	-0.01308	0.81742	0.92641	1.0576	0.080785	0.021245	0.22709	0.48768	-1.036	0.96007	0.98693	0.91936	-0.1213	0.78997	0.94841	0.85463	-0.22663
TAG420-FA160	0.47786	0.91799	0.9484	-0.07644	0.001017	0.043099	1.6126	0.68942	0.14842	0.37771	1.3002	0.37874	0.28432	0.54431	1.0771	0.10715	0.051595	0.59265	1.4521	0.53815
CE140	0.37264	0.90782	0.89769	-0.15571	0.036262	0.21405	1.6975	0.76342	0.37227	0.57954	1.1414	0.19078	0.70278	0.86826	1.0857	0.11858	0.42082	0.8461	0.93274	-0.10045
TAG564-FA181	0.34041	0.90224	0.96953	-0.04465	0.51415	0.7547	0.83329	-0.26311	0.036142	0.25181	0.48473	-1.0447	0.12984	0.4002	0.33895	-1.5608	0.35987	0.82994	0.35929	-1.4768
TAG481-FA141	0.35966	0.90782	0.82516	-0.27725	0.086187	0.30525	0.70466	-0.50501	0.015835	0.22433	1.382	0.46673	0.13321	0.40066	0.87469	-0.19316	0.33115	0.79964	1.1081	0.14815
TAG440-FA180	0.48267	0.91799	0.93277	-0.10041	0.000615	0.041618	1.7743	0.82725	0.13974	0.36406	1.3369	0.41884	0.30846	0.57246	1.0798	0.11071	0.065986	0.63737	1.2258	0.29375
TAG441-FA181	0.28637	0.87497	0.87983	-0.1847	0.01702	0.14762	1.7394	0.79861	0.022441	0.22709	1.7543	0.81086	0.66712	0.8432	1.0025	0.00354	0.082773	0.6773	1.4434	0.52949
TAG472-FA140	0.2492	0.80776	0.8729	-0.1961	0.26137	0.53149	0.66538	-0.58775	0.03604	0.25181	1.4159	0.50168	0.94377	0.98186	1.0184	0.02629	0.45648	0.8461	0.96078	-0.05772
TAG501-FA180	0.97613	0.99393	1.0744	0.10354	0.078998	0.29329	1.3778	0.46237	0.092032	0.32588	1.3401	0.42234	0.065336	0.33448	0.73495	-0.44428	0.04919	0.59265	0.76798	-0.38087
TAG482-FA160	0.089098	0.63111	0.87838	-0.18709	0.15377	0.40798	0.77422	-0.36919	0.013378	0.21636	1.2979	0.37618	0.068622	0.33448	0.79913	-0.3235	0.45596	0.8461	0.95945	-0.05973
TAG491-FA181	0.79449	0.9837	1.0207	0.029603	0.33019	0.59462	0.80603	-0.3111	0.045051	0.28157	1.2412	0.31172	0.073307	0.33448	0.86316	-0.2123	0.035101	0.56697	0.84761	-0.23853
TAG553-FA181	0.72462	0.96748	0.92752	-0.10855	0.43326	0.70883	1.1637	0.21875	0.024704	0.22824	0.73457	-0.44503	0.81109	0.93928	0.99699	-0.00435	0.542	0.86495	0.95058	-0.07312
TAG541-FA181	0.37775	0.90782	1.166	0.22158	0.011847	0.12588	1.388	0.47305	0.072321	0.32017	0.85552	-0.22512	0.16869	0.43582	1.1864	0.24654	0.57671	0.87674	1.0996	0.13702
TAG421-FA181	0.57412	0.96666	0.91853	-0.12261	0.012854	0.12705	1.9691	0.97757	0.026828	0.24259	1.7626	0.8177	0.60152	0.83816	0.98614	-0.02013	0.029347	0.54228	1.5122	0.59665
TAG422-FA182	0.016132	0.52739	0.68804	-0.53943	0.034912	0.20995	1.6522	0.72439	0.053979	0.29916	1.5887	0.66781	0.18594	0.44626	1.206	0.27021	0.17034	0.70044	1.1575	0.21103
TAG421-FA140	0.13995	0.7065	0.84717	-0.23928	0.41787	0.69103	1.1046	0.14355	0.046518	0.28652	1.3655	0.44939	0.14076	0.40421	1.1537	0.20627	0.31361	0.77043	1.1283	0.17416
TAG512-FA150	0.95878	0.99393	1.208	0.2726	0.76931	0.90976	0.87054	-0.20001	0.84805	0.91191	0.82048	-0.28545	0.11364	0.38463	0.62312	-0.68242	0.010935	0.5398	0.56235	-0.83047
TAG547-FA183	0.9845	0.99622	0.44749	-1.1601	0.93714	0.98209	1.5337	0.61701	0.035988	0.25181	0.53533	-0.90151	0.92223	0.9799	1.0361	0.051177	0.47732	0.85957	1.2746	0.35
TAG525-FA181	0.14087	0.7065	0.81817	-0.28952	0.12379	0.3727	1.2199	0.28672	0.029304	0.24396	0.78707	-0.34543	0.99743	0.99874	1.022	0.031342	0.36699	0.82994	0.92274	-0.11601
TAG566-FA202	0.041408	0.55845	4.6865	2.2285	0.002376	0.046168	1.9054	0.93012	0.53777	0.70323	1.0871	0.1205	0.80898	0.93928	1.0603	0.084493	0.10424	0.6849	3.1759	1.6672
TAG460-FA180	0.99263	0.99651	1.036	0.051086	9.78E-05	0.01386	1.689	0.75617	0.19763	0.41083	1.2391	0.30935	0.45466	0.72102	1.0513	0.072192	0.14659	0.70044	1.1771	0.23528
TAG502-FA181	0.35609	0.90782	1.143	0.19278	0.59369	0.80957	1.1083	0.14831	0.088417	0.32588	1.2406	0.31102	0.014255	0.31888	0.65462	-0.61126	0.32738	0.79507	0.84631	-0.24074
TAG481-FA120	0.52064	0.9456	0.88101	-0.18277	0.048207	0.23411	1.6752	0.74436	0.08135	0.32588	1.5937	0.67237	0.12823	0.4002	0.83932	-0.2527	0.086058	0.6773	1.3323	0.41395
TAG511-FA181	0.64523	0.96666	1.0382	0.054063	0.83889	0.93332	0.96635	-0.04939	0.15598	0.38012	1.1209	0.16462	0.01522	0.31888	0.85174	-0.23151	0.039973	0.57294	0.80733	-0.30877
TAG532-FA181	0.58047	0.96666	1.1013	0.13914	0.04242	0.21462	1.2973	0.37554	0.050841	0.29599	0.82183	-0.28308	0.04621	0.33448	1.2231	0.29054	0.69128	0.90958	1.0506	0.071233
HCER160	0.07138	0.59484	2.4868	1.3143	0.034991	0.20995	2.1497	1.1041	0.054202	0.29916	1.7534	0.8102	0.97357	0.98751	1.0386	0.054601	0.54778	0.86495	1.3391	0.42131
TAG521-FA181	0.58741	0.96666	1.3046	0.38361	0.047034	0.23244	1.5248	0.60863	0.88925	0.92801	1.0092	0.013231	0.073456	0.33448	0.64126	-0.64102	0.12455	0.70044	0.69626	-0.52231
HCER201	0.95885	0.99393	1.128	0.17373	0.013829	0.13061	2.1907	1.1314	0.30343	0.50571	1.9761	0.98265	0.002748	0.23372	3.1023	1.6333	0.12144	0.70044	1.1881	0.24861
TAG545-FA225	0.54539	0.96666	0.94055	-0.08843	0.013194	0.12744	1.5004	0.58539	0.28964	0.49042	0.83037	-0.26817	0.067375	0.33448	1.2323	0.30134	0.11334	0.70044	1.451	0.53704
SYMMETRIC DIMETHYLARGININE_po	0.4954	0.92681	2.5229	1.3351	0.040526	0.21415	0.66118	-0.59689	0.44882	0.63654	0.90248	-0.14803	0.78228	0.92353	0.8569	-0.22281	0.77785	0.94214	0.99881	-0.00171
FFA204	0.16903	0.73306	1.7895	0.83952	0.12218	0.37091	1.548	0.63042	0.17715	0.40079	0.77028	-0.37655	0.034115	0.33448	1.3993	0.48466	0.2939	0.77043	1.3489	0.43177
TAG492-FA160	0.99226	0.99651	1.0543	0.076269	0.002077	0.046168	0.7204	-0.47313	0.89962	0.92801	1.0284	0.040468	0.18017	0.44519	0.84647	-0.24047	0.002934	0.5398	0.7642	-0.38798
TAG400-FA160	0.64106	0.96666	0.97379	-0.03831	0.002755	0.04683	1.6498	0.72229	0.16618	0.39544	1.3247	0.40564	0.20586	0.46047	1.1351	0.18288	0.012	0.5398	1.5126	0.59699
TAG521-FA180	0.73094	0.96748	1.2963	0.37445	0.037987	0.21415	1.5243	0.60815	0.9556	0.96866	0.97027	-0.04354	0.096509	0.3763	0.5862	-0.77053	0.10529	0.6849	0.60143	-0.73354
TAG483-FA120	0.32886	0.88458	0.87813	-0.18749	0.018974	0.15598	1.5825	0.66223	0.066364	0.30521	1.3226	0.40343	0.41577	0.67702	0.8524	-0.2304	0.63741	0.87674	1.1126	0.15393
TAG502-FA161	0.29798	0.87614	1.1769	0.23499	0.00146	0.04527	0.59675	-0.7448	0.51648	0.69588	1.0365	0.051716	0.076041	0.33664	0.65173	-0.61765	0.17002	0.70044	0.77691	-0.36417
TAG504-FA182	0.46612	0.91799	0.85879	-0.21961	0.068788	0.27795	0.67509	-0.56684	0.59707	0.74436	1.0452	0.063837	0.043491	0.33448	0.57014	-0.81062	0.17067	0.70044	0.68615	-0.5434
TAG491-FA160	0.26106	0.82799	1.1684	0.22449	0.021749	0.16217	0.65148	-0.61822	0.19104	0.41042	1.1554	0.20836	0.18602	0.44626	0.87384	-0.19456	0.29643	0.77043	0.93323	-0.09969
TAG420-FA140	0.91097	0.99393	1.0291	0.041361	0.1831	0.43345	1.1395	0.18844	0.23423	0.44264	1.1859	0.24594	0.20336	0.4573	1.1244	0.16914	0.018949	0.5398	1.3408	0.42315
DAG120/181	0.27924	0.86645	0.90668	-0.14133	0.29487	0.55697	1.2333	0.30247	0.7696	0.85847	0.91807	-0.12333	0.020153	0.32942	1.3829	0.46768	0.98949	0.99956	0.97258	-0.04011
TAG563-FA182	0.61034	0.96666	0.86486	-0.20946	0.096005	0.32496	0.69476	-0.52542	0.44598	0.63654	0.75028	-0.41449	0.047962	0.33448	0.45441	-1.1379	0.097985	0.6849	0.49625	-1.0108

TAG587-FA182	0.60977	0.96666	0.91622	-0.12624	0.24115	0.50707	1.4079	0.49353	0.04271	0.27926	0.56344	-0.82767	0.90187	0.97037	1.0497	0.069924	0.48892	0.8639	0.79779	-0.32591
DAG181/181	0.65402	0.96666	0.89477	-0.16041	0.032616	0.20385	1.323	0.40382	0.17729	0.40079	0.8842	-0.17756	0.083347	0.34908	1.162	0.21658	0.46663	0.85115	1.0959	0.1321
TAG482-FA161	0.79012	0.98188	1.0731	0.10172	0.039764	0.21415	0.55516	-0.84903	0.1115	0.34512	1.3522	0.4353	0.4585	0.72171	0.87779	-0.18805	0.89173	0.9879	1.0189	0.02707
TAG561-FA181	0.38291	0.90782	1.1106	0.1514	0.03071	0.20385	1.2198	0.28664	0.018115	0.22709	0.81571	-0.29387	0.20221	0.4573	1.1557	0.20875	0.44167	0.8461	0.94093	-0.08784
N-ACETYLALANINE_neg_1	0.029921	0.55845	3.0959	1.6303	0.57708	0.79889	1.2351	0.30467	0.60195	0.74436	1.1367	0.18488	0.59278	0.83146	0.76396	-0.38843	0.017134	0.5398	0.51894	-0.94637
DAG180/181	0.4803	0.91799	1.0857	0.11868	0.040411	0.21415	1.4438	0.52987	0.15501	0.38012	0.85262	-0.23003	0.018758	0.31888	1.2748	0.35032	0.80001	0.95275	0.9917	-0.01202
ALLANTOIN_neg_2	0.94162	0.99393	0.85824	-0.22055	0.092768	0.32054	0.56045	-0.83534	0.10065	0.3395	0.76558	-0.38538	0.041824	0.33448	1.7998	0.84782	0.29657	0.77043	1.5442	0.62688
INDOLEACRYLIC ACID_pos_1	0.12866	0.7065	0.70433	-0.50568	0.8587	0.94691	1.2355	0.30509	0.035929	0.25181	3.9306	1.9748	0.10582	0.38027	1.0346	0.049027	0.97006	0.99778	1.2565	0.32943
TAG481-FA180	0.65255	0.96666	0.99002	-0.01448	0.035074	0.20995	1.4039	0.48947	0.011844	0.21636	1.4907	0.57597	0.6055	0.83816	1.0494	0.069589	0.60086	0.87674	0.99641	-0.00518
TAG400-FA140	0.71149	0.96748	0.96278	-0.05472	0.23529	0.50707	1.1271	0.17268	0.56261	0.72238	1.1148	0.15682	0.046781	0.33448	1.3358	0.41775	0.002668	0.5398	1.4613	0.54729
TAG543-FA183	0.22484	0.80776	1.3103	0.38986	0.68979	0.87195	1.3833	0.46814	0.012534	0.21636	0.44998	-1.1521	0.064068	0.33448	1.5541	0.63608	0.43588	0.8461	0.92926	-0.10584
TAG522-FA181	0.60549	0.96666	1.1821	0.24136	0.19221	0.44776	1.3275	0.4087	0.88879	0.92801	0.86788	-0.20443	0.03691	0.33448	0.5606	-0.83496	0.2882	0.77043	0.69617	-0.5225
TAG541-FA180	0.81588	0.99092	1.1708	0.22753	0.032139	0.20385	1.483	0.56855	0.73433	0.84122	0.86506	-0.20913	0.21788	0.47269	0.63407	-0.65729	0.10166	0.6849	0.58415	-0.77558
TAG484-FA182	0.079818	0.61677	0.60361	-0.72831	0.005253	0.067646	0.48575	-1.0417	0.092528	0.32588	1.3754	0.45984	0.26997	0.52153	0.77114	-0.37493	0.7322	0.93556	0.86977	-0.2013
XANTHURENIC ACID_pos_2	0.96005	0.99393	1.751	0.8082	0.61382	0.81779	0.59892	-0.73957	0.71858	0.82539	0.87882	-0.18636	0.001051	0.23372	0.46406	-1.1076	0.84965	0.97398	0.97555	-0.03572
TAG541-FA200	0.1234	0.7065	0.72718	-0.45962	0.113	0.35839	0.71446	-0.48507	0.50846	0.6904	0.78597	-0.34745	0.002734	0.23372	0.5804	-0.78487	0.5316	0.86495	1.1014	0.13938
TAG400-FA120	0.6758	0.96666	0.93938	-0.09023	0.83813	0.93332	0.96338	-0.05382	0.98905	0.99138	1.0332	0.04705	0.054078	0.33448	1.3858	0.4707	0.008467	0.5398	1.4414	0.52743
TAG480-FA180	0.99649	0.99651	1.0085	0.012189	0.002732	0.04683	1.43	0.51601	0.41585	0.61605	1.0966	0.13305	0.55052	0.79572	1.0448	0.063161	0.14402	0.70044	1.2766	0.35226
METHIONINE_pos_1	0.54779	0.96666	1.1023	0.14053	0.5116	0.7547	0.79041	-0.33933	0.013728	0.21636	0.53885	-0.89205	0.89093	0.9683	1.037	0.052479	0.61525	0.87674	1.5766	0.65681
TAG492-FA182	0.23614	0.80776	0.90279	-0.14753	0.27836	0.54518	0.76781	-0.38117	0.2084	0.41976	1.3093	0.38884	0.10784	0.38027	0.78407	-0.35094	0.020146	0.5398	0.74807	-0.41875
TAG483-FA160	0.76473	0.96748	0.86908	-0.20243	0.83429	0.93309	0.98418	-0.02301	0.18024	0.40419	1.2433	0.31421	0.007337	0.31888	0.61718	-0.69623	0.34288	0.81411	0.79482	-0.3313
SM241	0.67809	0.96666	0.87519	-0.19234	0.012612	0.12705	1.7614	0.81673	0.15956	0.38313	0.78312	-0.35269	0.24481	0.48848	1.437	0.52303	0.75518	0.93556	1.2275	0.29578
TAG565-FA202	0.051831	0.55845	5.4308	2.4412	0.00239	0.046168	1.7305	0.79117	0.84464	0.91191	1.0565	0.07935	0.23447	0.48293	1.1753	0.23303	0.17723	0.70044	2.9988	1.5844
TAG493-FA161	0.4144	0.90782	0.95093	-0.07259	0.002536	0.04683	0.5308	-0.91377	0.18326	0.40419	1.2224	0.28975	0.39342	0.65494	0.89028	-0.16766	0.21069	0.70044	0.91445	-0.12902
TAG546-FA181	0.41697	0.90782	0.35308	-1.5019	0.63799	0.83979	1.4258	0.51174	0.004011	0.16803	0.56923	-0.81291	0.31731	0.5835	0.85778	-0.22132	0.44963	0.8461	1.1422	0.19182
TAG481-FA160	0.37544	0.90782	1.1117	0.15282	0.60727	0.81161	0.88173	-0.18158	0.004074	0.16803	1.3949	0.48019	0.025524	0.33448	0.8088	-0.30614	0.7349	0.93556	0.99392	-0.0088
TAG563-FA181	0.57616	0.96666	1.1137	0.15541	0.032078	0.20385	1.2897	0.36707	0.031448	0.25181	0.82739	-0.27336	0.27249	0.52403	1.1879	0.24844	0.96797	0.99778	1.0654	0.091385
TAG462-FA120	0.050411	0.55845	0.64069	-0.6423	0.70987	0.87195	1.0874	0.12086	0.003902	0.16803	1.6897	0.75674	0.045428	0.33448	0.79104	-0.33818	0.33698	0.80913	0.94778	-0.07737
TAG543-FA202	0.036055	0.55845	5.2573	2.3943	0.001806	0.046168	1.8086	0.85491	0.9825	0.98715	1.0366	0.051803	0.15765	0.42629	1.1781	0.23644	0.17991	0.70044	2.6229	1.3911
TAG512-FA160	0.65783	0.96666	1.1044	0.1432	0.069881	0.27795	1.2744	0.34985	0.17562	0.40079	0.83936	-0.25264	0.039845	0.33448	1.2658	0.34	0.53945	0.86495	1.1157	0.15794
2-KETOHEXANOIC ACID_pos_2	0.88738	0.99393	1.056	0.078571	0.21135	0.47423	1.2906	0.36801	0.032661	0.25181	1.9187	0.94012	0.049372	0.33448	2.2674	1.181	0.87741	0.98391	1.0603	0.084462
TAG542-FA182	0.76925	0.96748	1.0061	0.008761	0.23082	0.50307	1.3144	0.39445	0.033205	0.25181	0.68038	-0.55559	0.84374	0.95624	0.91381	-0.13003	0.064872	0.63737	0.66204	-0.59501
TAG492-FA181	0.55282	0.96666	0.97321	-0.03917	0.045241	0.2262	0.66203	-0.59504	0.125	0.36388	1.223	0.29048	0.54609	0.79333	0.92882	-0.10653	0.092903	0.68076	0.88755	-0.1721
TAG561-FA160	0.30546	0.87614	0.9618	-0.0562	0.002292	0.046168	0.47805	-1.0648	0.83877	0.91191	0.93791	-0.09248	0.002655	0.23372	0.42007	-1.2513	0.82253	0.95829	0.75995	-0.39603
TAG503-FA161	0.27273	0.8586	1.2067	0.2711	0.000783	0.041618	0.53133	-0.91232	0.93282	0.95071	1.017	0.024265	0.1009	0.38027	0.5817	-0.78166	0.025691	0.54228	0.62725	-0.67288
TAG421-FA120	0.84675	0.99393	0.95753	-0.06261	0.1439	0.40251	0.69267	-0.52977	0.59544	0.74436	1.1013	0.1392	0.11403	0.38463	1.3455	0.42809	0.023553	0.5398	1.4213	0.50722
TAG543-FA181	0.61357	0.96666	0.95084	-0.07272	0.97831	0.99053	1.0936	0.12903	0.41668	0.61605	0.76737	-0.38201	0.014476	0.31888	0.56105	-0.83379	0.7706	0.94111	1.0472	0.066567
TAG525-FA160	0.48477	0.91799	0.3788	-1.4005	0.51497	0.7547	1.6119	0.68875	0.048637	0.29114	0.63654	-0.65167	0.35941	0.62121	0.80356	-0.31551	0.21182	0.70044	1.2669	0.34127
TAG420-FA120	0.71986	0.96748	0.97246	-0.0403	0.71974	0.879	0.98665	-0.01939	0.63903	0.77714	1.0813	0.11279	0.10379	0.38027	1.2377	0.30766	0.004973	0.5398	1.4351	0.52112
ADENOSINE PHOSPHOSULFATE_neg	0.92538	0.99393	1.0525	0.073775	0.00519	0.067646	6.3293	2.6621	0.19913	0.41083	2.228	1.1558	0.30003	0.56556	1.8532	0.88998	0.49391	0.8639	0.80116	-0.31983
DAG140/182	0.99651	0.99651	1.049	0.069	0.097416	0.32496	1.8273	0.8697	0.006184	0.18773	1.7549	0.81143	0.47692	0.7347	1.1715	0.22838	0.76773	0.94031	0.88907	-0.16963
TAG460-FA120	0.20397	0.79448	0.88575	-0.17503	0.020459	0.16102	1.3724	0.45668	0.22382	0.43658	1.2108	0.27593	0.87242	0.96795	1.0383	0.054204	0.10636	0.6849	1.1923	0.25379
TAG492-FA150	0.84123	0.99393	1.0734	0.10213	0.085824	0.30525	0.63512	-0.65491	0.4125	0.61605	1.0453	0.063927	0.12477	0.4002	0.76185	-0.39241	0.029262	0.54228	0.74473	-0.4252
CER160	0.12397	0.7065	1.7762	0.8288	0.13411	0.38512	0.51156	-0.96703	0.081001	0.32588	0.78314	-0.35265	0.22509	0.48072	0.42419	-1.2372	0.009063	0.5398	0.33228	-1.5895
CER240	0.97426	0.99393	1.4913	0.57661	0.019412	0.15598	0.35863	-1.4794	0.39326	0.60467	0.68705	-0.54152	0.20249	0.4573	0.28811	-1.7953	0.051511	0.59265	0.12757	-2.9707
PIPECOLATE_pos_3	0.37087	0.90782	2.9798	1.5752	0.30264	0.56662	0.76144	-0.3932	0.002154	0.16803	0.49982	-1.0005	0.46858	0.72681	0.83771	-0.25547	0.87562	0.98391	0.82043	-0.28554
TAG421-FA161	0.050371	0.55845	0.80578	-0.31154	0.63296	0.83803	0.75664	-0.40232	0.023821	0.22824	1.4597	0.54564	0.87457	0.96795	1.0379	0.053713	0.3	0.77043	1.0876	0.12109
TAG492-FA161	0.90172	0.99393	1.0348	0.049325	0.024083	0.17647	0.53747	-0.89573	0.34701	0.55652	1.2229	0.29033	0.82213	0.9418	0.94539	-0.08102	0.42662	0.8461	0.95209	-0.07083
TAG482-FA182	0.19414	0.77762	0.91219	-0.13259	0.63824	0.83979	1.0231	0.032896	0.01109	0.21636	1.4853	0.57073	0.069156	0.33448	0.81579	-0.29373	0.18945	0.70044	0.8899	-0.16828
TAG522-FA160	0.68651	0.96666	1.1096	0.15003	0.44207	0.71438	1.2131	0.27869	0.60137	0.74436	0.85244	-0.23033	0.044376	0.33448	0.54913	-0.86479	0.43616	0.8461	0.68694	-0.54174
TAG483-FA140	0.14188	0.7065	0.61604	-0.6989	0.097088	0.32496	0.57044	-0.80986	0.36331	0.56977	1.1894	0.25022	0.049997	0.33448	0.56899	-0.81353	0.21306	0.70044	0.66991	-0.57796
TAG564-FA202	0.03639	0.55845	5.4807	2.4544	0.001953	0.046168	1.7715	0.82499	0.87457	0.92692	1.0541	0.076073	0.24138	0.48389	1.145	0.19532	0.19856	0.70044	2.6613	1.4121
TAG503-FA182	0.78714	0.98104	1.0852	0.11791	0.21201	0.47423	0.78094	-0.35672	0.60319	0.74436	1.0094	0.013481	0.054013	0.33448	0.54552	-0.87429	0.044907	0.59265	0.57111	-0.80816
TAG563-FA202	0.037697	0.55845	5.1752	2.3716	0.003299	0.051932	1.796	0.8448	0.89443	0.92801	1.0555	0.077947	0.13668	0.40066	1.2002	0.26323	0.16462	0.70044	2.4405	1.2872
TAG533-FA182	0.054905	0.55845	0.70229	-0.50986	0.98587	0.99053	1.0137	0.019644	0.42787	0.62063	0.91185	-0.13313	0.0375	0.33448	0.70753	-0.49914	0.069943	0.66057	0.68635	-0.54299
TAG513-FA161	0.12659	0.7065	0.83164	-0.26597	0.004826	0.067646	0.52549	-0.92826	0.84476	0.91191	1.0059	0.008545	0.55257	0.79572	1.1194	0.16271	0.99341	0.99956	1.049	0.069003
TAG503-FA141	0.75422	0.96748	0.90265	-0.14777	0.12899	0.37549	0.69307	-0.52893	0.64409	0.77923	1.0455	0.064141	0.007806	0.31888	0.54706	-0.87023	0.6632	0.89765	0.85012	-0.23426
TAG583-FA181	0.48207	0.91799	0.82332	-0.28048	0.51071	0.7547	1.1114	0.15241	0.022129	0.22709	0.52357	-0.93354	0.052467	0.33448	0.34717	-1.5263	0.913	0.9879	0.69847	-0.51772
TAG545-FA181	0.84707	0.99393	0.50232	-0.99333	0.79429	0.92233	1.3851	0.46994	0.013586	0.21636	0.59858	-0.74038	0.21578	0.47269	0.68086	-0.55457	0.63404	0.87674	1.028	0.039907
N-GLYCYL-L-PROLINE_pos_1	0.009438	0.42218	3.0733	1.6198	0.35395	0.62397	0.96891	-0.04557	0.21438	0.42576	1.0187	0.026783	0.3878	0.65403	0.90064	-0.15098	0.60219	0.87674	1.7572	0.81328
TAG493-FA182	0.14435	0.7065	0.84141	-0.24912	0.18018	0.43196	0.68289	-0.55027	0.30683	0.50938	1.2075	0.272	0.65265	0.8432	0.89744	-0.1561	0.024132	0.5398	0.79533	-0.33037
TAG512-FA182	0.93274	0.99393	1.0925	0.12757	0.13662	0.38968	0.66761	-0.58291	0.64	0.77714	1.2212	0.28827	0.017978	0.31888	0.62341	-0.68174	0.18032	0.70044	0.62302	-0.68264
TAG512-FA170	0.41608	0.90782	0.98909	-0.01583	0.016886	0.14762	0.59938	-0.73847	0.53746	0.70323	1.0877	0.12131	0.083778	0.34908	0.78489	-0.34943	0.02767	0.54228	0.74472	-0.42522
CER220	0.02342	0.55845	2.2635	1.1786	0.48074	0.74076	0.93891	-0.09094	0.005035	0.16803	0.48655	-1.0394	0.47712	0.7347	0.58839	-0.76516	0.022114	0.5398	0.21186	-2.2388
TAG555-FA181	0.96946	0.99393	1.0549	0.077058	0.037066	0.21415	1.3438	0.42635	0.14607	0.37398	0.81816	-0.28954	0.056903	0.33448	1.2916	0.36916	0.59034	0.87674	1.116	0.1583
TAG504-FA161	0.087059	0.62712	0.86387	-0.21111	0.004198	0.061525	0.509	-0.97427	0.48547	0.6683	0.8542	-0.22736	0.32358	0.58581	0.56369	-0.82703	0.16613	0.70044	0.60561	-0.72355
TAG534-FA182	0.89725	0.99393	1.084	0.11637	0.041919	0.21462	1.3081	0.38752	0.18239	0.40419	0.84881	-0.23649	0.045841	0.33448	1.3173	0.39756	0.55216	0.86495	1.1438	0.19384
LACTATE_neg_1	0.038272	0.55845	3.8055	1.9281	0.16184	0.41435	0.47732	-1.067	0.1359	0.36406	0.70223	-0.50998	0.23948	0.48346	0.60003	-0.73688	0.24971	0.73895	0.67584	-0.56524
SM180	0.76297	0.96748	1.1119	0.15298	0.92193	0.97918	1.0829	0.11484	0.95969	0.96866	0.87725	-0.18894	0.027882	0.33448	0.71155	-0.49097	0.43941	0.8461	1.1454	0.19589
LCER160	0.006677	0.42218	4.5883	2.198	0.009535	0.10952	4.7942	2.2613	0.020693	0.22709	3.2833	1.7152	0.1824	0.44553	1.8147	0.85973	0.36382	0.82994	2.3543	1.2353
TAG546-FA226	0.29266	0.87614	1.6756	0.74465	0.4909	0.74247	1.1415	0.19095	0.002937	0.16803	0.61691	-0.69687	0.12995	0.4002	1.3845	0.46934	0.69112	0.90958	0.95201	-0.07096
TAG525-FA205	0.6982	0.96666	1.0535	0.075218	0.02484	0.17893	1.8933	0.92088	0.31626	0.52301	1.2862	0.36309	0.00275	0.23372	1.963	0.97305	0.017638	0.5398	1.7611	0.81652
BETAINE_pos_2	0.43358	0.90782	1.5846	0.66409	0.82332	0.92814	0.91663	-0.12559	0.013087	0.21636	0.35035	-1.5131	0.93638	0.98186	0.95397	-0.06799	0.20381	0.70044	1.1552	0.20818
TAG481-FA140	0.38734	0.90782	1.073	0.1016	0.27291	0.53757	1.2403	0.31064	0.011703	0.21636	1.4657	0.55156	0.06835	0.33448	0.86583	-0.20784	0.44825	0.8461	1.0012	0.00174
TAG513-FA182	0.15269	0.7065	0.96246	-0.0552	0.060822	0.2611	0.58078	-0.78394	0.80057	0.87918	0.99932	-0.00098	0.14894	0.41919	0.60583	-0.72301	0.012482	0.5398	0.54851	-0.8664
TAG481-FA181	0.22995	0.80776	1.1363	0.1843	0.11542	0.36335	1.3578	0.44129	0.005124	0.16803	1.5622	0.64356	0.13645	0.40066	0.8789	-0.18622	0.91227	0.9879	1.1133	0.15484
TAG481-FA161	0.13937	0.7065	1.1707	0.2274	0.031708	0.20385	0.61821	-0.69383	0.16839	0.39759	1.2175	0.28396	0.60552	0.83816	0.91813	-0.12323	0.96501	0.99778	1.0298	0.042306
TAG543-FA161	0.50798	0.92681	0.832	-0.26534	0.021525	0.16217	0.45474	-1.1369	0.18192	0.40419	0.78056	-0.35741	0.12643	0.4002	0.59405	-0.75134	0.31338	0.77043	0.67913	-0.55824
TAG492-FA140	0.07555	0.61677	0.70711	-0.49999	0.040409	0.21415	0.54566	-0.87394	0.89675	0.92801	1.0458	0.064603	0.64999	0.8432	1.1305	0.17691	0.037586	0.57051	1.3506	0.43364
TAG472-FA181	0.82747	0.99393	0.98879	-0.01626	0.22519	0.49588	1.1817	0.24086	0.68988	0.80771	0.92329	-0.11515	0.017963	0.31888	1.2844	0.3611	0.25562	0.73895	1.286	0.36284
FFA241	0.68647	0.96666	1.3085	0.3879	0.80602	0.92331	1.0451	0.063581	0.029849	0.24396	0.51508	-0.95713	0.17814	0.44519	1.6633	0.73403	0.29294	0.77043	1.2755	0.35103
CE205	0.009934	0.42218	0.60856	-0.71653	0.001491	0.04527	2.4282	1.2799	0.15947	0.38313	1.2801	0.3562	0.14003	0.40421	1.3714	0.4557	0.48418	0.8639	0.90995	-0.13615
TAG551-FA160	0.69927	0.96666	0.91116	-0.13422	0.000206	0.019885	0.43056	-1.2157	0.74375	0.84228	1.0033	0.00471	0.095646	0.3763	0.69142	-0.53237	0.7877	0.94836	1.0912	0.12593
TAG502-FA180	0.18451	0.76702	0.91562	-0.12717	0.92698	0.98002	1.0521	0.073327	0.065986	0.30521	1.3565	0.43987	0.008719	0.31888	0.5595	-0.83779	0.054227	0.60649	0.60132	-0.73379
TAG510-FA180	0.84033	0.99393	1.0403	0.056951	0.030513	0.20385	1.3045	0.38352	0.53198	0.70215	0.96463	-0.05196	0.072127	0.33448	1.1547	0.2075	0.52473	0.86495	1.068	0.094861
TAG480-FA160	0.035824	0.55845	1.2806	0.35684	0.6057	0.81161	1.1379	0.18634	0.22955	0.44145	0.80052	-0.32099	0.23188	0.48293	1.1574	0.21083	0.10189	0.6849	1.1609	0.2153
TAG461-FA180	0.055342	0.55845	0.68721	-0.54118	0.001934	0.046168	1.7227	0.78464	0.12742	0.36394	1.2654	0.33955	0.45806	0.72171	0.9192	-0.12155	0.97306	0.99778	1.0568	0.079648
TAG544-FA180	0.87233	0.99393	0.56805	-0.81592	0.87121	0.95183	1.2341	0.30348	0.019984	0.22709	0.65596	-0.60832	0.16007	0.42629	0.60116	-0.73417	0.55333	0.86495	0.81492	-0.29526
ASPARTATE_pos_3	0.95569	0.99393	1.0332	0.047059	0.076738	0.28862	1.4333	0.5193	0.013745	0.21636	1.9828	0.98756	0.052165	0.33448	1.7219	0.78399	0.97213	0.99778	1.0355	0.050378
TAG505-FA160	0.14833	0.7065	0.62233	-0.68424	0.038703	0.21415	1.3544	0.43765	0.20201	0.4111	0.77566	-0.3665	0.13668	0.40066	0.80828	-0.30708	0.20095	0.70044	0.7229	-0.46813
TAG472-FA182	0.1566	0.71564	0.88454	-0.177	0.54298	0.77438	0.85509	-0.22584	0.15354	0.38012	1.1657	0.22123	0.037406	0.33448	1.2192	0.2859	0.61349	0.87674	0.96009	-0.05876
TAG526-FA181	0.91868	0.99393	1.0568	0.079638	0.01925	0.15598	1.3899	0.47495	0.54822	0.70818	0.8993	-0.15312	0.025708	0.33448	1.3532	0.43639	0.68999	0.90958	1.0761	0.10581
BIOTIN_pos_4	0.48409	0.91799	1.1017	0.13979	0.78772	0.91721	1.0358	0.050804	0.022153	0.22709	1.5687	0.64959	0.25308	0.49795	1.3513	0.4343	0.95386	0.99778	1.0218	0.031047
TAG568-FA204	0.41499	0.90782	1.2452	0.31633	0.012615	0.12705	1.4338	0.51986	0.45243	0.63882	0.92825	-0.10742	0.15464	0.42401	1.2071	0.27154	0.36462	0.82994	2.1724	1.1193
SM240	0.70737	0.96666	1.1302	0.17663	0.041319	0.21415	1.7705	0.8242	0.67124	0.79464	1.1429	0.19274	0.85222	0.95986	1.067	0.093618	0.41875	0.8461	1.2315	0.3004
TAG565-FA225	0.039263	0.55845	0.68059	-0.55514	0.000695	0.041618	1.6898	0.75689	0.093034	0.32588	0.79893	-0.32387	0.029594	0.33448	1.3498	0.43272	0.085495	0.6773	1.2198	0.28666
TAG503-FA181	0.15025	0.7065	0.90297	-0.14725	0.70192	0.87195	1.0228	0.032477	0.28752	0.49042	1.0749	0.1042	0.060914	0.33448	0.73076	-0.45252	0.023486	0.5398	0.7574	-0.40088
L-ORNITHINE_pos_1	0.14996	0.7065	0.39739	-1.3314	0.10656	0.3457	2.2613	1.1771	0.8799	0.92801	0.93672	-0.09431	0.035454	0.33448	2.6276	1.3937	0.19319	0.70044	1.2414	0.31199

Supplementary Table S4. List of metabolites showing co-relation with heart dysfuction following 9.5 Gy of Gamma-radiation at 1 week and 1 month post-irradiation.

	1 Week				1 Month			
Name	p-value	FDR	Fold Change	Log2(FC)	p-value	FDR	Fold Change	Log2(FC)
TAG544-FA160	0.014408	0.31041	1.4308 ↑	0.51678	0.00076955	0.32706	2.0944 ↑	1.0665
TAG524-FA204	0.20853	0.60702	1.2322 ↑	0.30129	0.0020708	0.44004	1.7497 ↑	0.80709
SPERMIDINE_pos_4	0.15569	0.56567	0.72461 ↓	-0.46473	0.0070866	0.51123	0.66972 ↓	-0.57837
FFA161	0.00031319	0.066554	0.49327 ↓	-1.0195	0.0081376	0.51123	0.64731 ↓	-0.62748
CE226	0.029381	0.31041	1.4628 ↑	0.54874	0.0092877	0.51123	2.9805 ↑	1.5755
INDOLE-3-CARBOXYLIC ACID_neg_1	0.11573	0.53256	0.63227 ↓	-0.66139	0.014323	0.51123	1.5816 ↑	0.66141
TAG522-FA161	0.057556	0.43372	0.79515 ↓	-0.3307	0.01441	0.51123	1.5213 ↑	0.60533
TAG545-FA160	0.020139	0.31041	1.9306 ↑	0.94906	0.016435	0.51123	2.2189 ↑	1.1499
TAG543-FA160	0.065619	0.44981	1.4114 ↑	0.49708	0.019341	0.51123	1.8954 ↑	0.92251
TAG472-FA181	0.86526	0.97815	1.004 ↑	0.0058141	0.022537	0.51123	0.80089 ↓	-0.32033
TAG564-FA204	0.81591	0.95527	1.117 ↑	0.15963	0.024565	0.51123	1.487 ↑	0.57239
SM160	0.060555	0.4362	1.2766 ↑	0.35233	0.024722	0.51123	1.4404 ↑	0.52643
TAG542-FA182	0.69838	0.91456	1.1352 ↑	0.18288	0.025882	0.51123	1.434 ↑	0.52007
CER220	0.9792	0.98616	1.1138 ↑	0.15553	0.028253	0.51123	2.1789 ↑	1.1236
TAG523-FA203	0.98783	0.99072	0.97949 ↓	-0.029896	0.029307	0.51123	1.4785 ↑	0.56412
TAG471-FA181	0.6076	0.88738	0.96988 ↓	-0.044125	0.030319	0.51123	0.83447 ↓	-0.26107
TAG451-FA181	0.60343	0.88434	0.98783 ↓	-0.017671	0.030518	0.51123	0.82418 ↓	-0.27896
TAG523-FA161	0.49301	0.83477	1.0035 ↑	0.0050856	0.03167	0.51123	1.8816 ↑	0.91196
TAG521-FA160	0.12591	0.54053	1.3115 ↑	0.39118	0.033322	0.51123	1.8712 ↑	0.90398
ANTHRANILIC ACID_pos_4	0.010778	0.30971	1.7307 ↑	0.79138	0.035273	0.51123	0.65952 ↓	-0.60052
TAG543-FA161	0.055942	0.43372	0.58385 ↓	-0.77634	0.035527	0.51123	1.465 ↑	0.55091
TAG534-FA160	0.86096	0.97815	0.93521 ↓	-0.096642	0.036364	0.51123	1.4131 ↑	0.49882
HOMOCYSTEINE_pos_2	0.00083077	0.080514	0.37503 ↓	-1.4149	0.040291	0.51123	0.58392 ↓	-0.77615
TAG470-FA170	0.11026	0.53251	0.87683 ↓	-0.18963	0.040591	0.51123	0.80787 ↓	-0.3078
TAG541-FA160	0.19849	0.60053	0.88692 ↓	-0.17312	0.041645	0.51123	1.4168 ↑	0.50268
TAG500-FA140	0.068285	0.46065	0.8511 ↓	-0.2326	0.042557	0.51123	0.82893 ↓	-0.27068
TAG567-FA204	0.0011399	0.080743	1.8834 ↑	0.91331	0.043629	0.51123	1.7223 ↑	0.78432
TAG542-FA180	0.044691	0.39704	1.5887 ↑	0.66781	0.047086	0.51123	1.9379 ↑	0.95449
TAG531-FA181	0.039109	0.38654	1.2567 ↑	0.32966	0.048942	0.51123	1.2781 ↑	0.35398
TAG542-FA181	0.085124	0.4961	1.4516 ↑	0.53769	0.051191	0.51123	1.8945 ↑	0.92181
TAG470-FA140	0.089932	0.49873	0.87058 ↓	-0.19995	0.051599	0.51123	0.82618 ↓	-0.27547
TAG525-FA161	0.42824	0.79826	1.6649 ↑	0.73547	0.051938	0.51123	2.7036 ↑	1.4349
TAG541-FA180	0.022596	0.31041	1.5353 ↑	0.61852	0.053086	0.51123	1.8916 ↑	0.91963
TAG470-FA150	0.09722	0.50224	0.88393 ↓	-0.178	0.053475	0.51123	0.84262 ↓	-0.24705
ADENOSINE PHOSPHOSULFATE_neg_2	0.61355	0.88757	0.96239 ↓	-0.055305	0.053781	0.51123	2.0219 ↑	1.0157

CE204	0.69009	0.91456	0.98164 ↓	-0.02674	0.054074	0.51123	4.1714 ↑	2.0605
TAG484-FA182	0.3529	0.74991	1.1412 ↑	0.19051	0.05449	0.51123	0.75427 ↓	-0.40684
TAG522-FA160	0.17355	0.57755	1.3789 ↑	0.46356	0.055193	0.51123	2.3272 ↑	1.2186
LACTATE_neg_1	0.3251	0.73885	1.6736 ↑	0.74292	0.055442	0.51123	2.0289 ↑	1.0207
TAG524-FA161	0.26284	0.66099	1.5904 ↑	0.6694	0.056067	0.51123	2.5691 ↑	1.3613
TAG451-FA160	0.37556	0.76877	0.94654 ↓	-0.079266	0.057856	0.51123	0.83964 ↓	-0.25216
DAG120/181	0.42584	0.79727	0.91712 ↓	-0.12482	0.058383	0.51123	0.80524 ↓	-0.31252
CER240	0.20598	0.60554	2.5189 ↑	1.3328	0.058433	0.51123	4.1368 ↑	2.0485
TAG502-FA180	0.33183	0.7434	1.1038 ↑	0.14252	0.062135	0.51123	1.3508 ↑	0.43379
TAG440-FA140	0.22649	0.62122	0.90369 ↓	-0.14611	0.067994	0.51123	0.85128 ↓	-0.23229
DAG160/181	0.63003	0.90045	0.98116 ↓	-0.027444	0.068448	0.51123	0.8351 ↓	-0.25997
TAG522-FA181	0.12582	0.54053	1.325 ↑	0.40599	0.071266	0.51123	2.2336 ↑	1.1594
TAG522-FA180	0.094649	0.49873	1.6707 ↑	0.74046	0.071638	0.51123	2.1273 ↑	1.089
TAG564-FA181	0.095052	0.49873	1.546 ↑	0.6285	0.072057	0.51123	1.8379 ↑	0.87803
TAG562-FA201	0.0346	0.35012	1.3665 ↑	0.45051	0.073396	0.51123	1.4399 ↑	0.526
TAG565-FA181	0.026159	0.31041	2.5025 ↑	1.3234	0.074315	0.51123	2.5195 ↑	1.3331
TAG542-FA160	0.9715	0.98616	1.0646 ↑	0.090286	0.075511	0.51123	1.6527 ↑	0.72483
TAG501-FA160	0.099267	0.50224	1.2361 ↑	0.30577	0.076784	0.51123	1.7452 ↑	0.8034
TAG502-FA182	0.46225	0.8244	1.208 ↑	0.27256	0.076884	0.51123	1.6327 ↑	0.70722
TAG462-FA182	0.89257	0.97842	0.99989 ↓	-0.00015971	0.078626	0.51123	0.85709 ↓	-0.22249
TAG503-FA140	0.11654	0.53256	1.2794 ↑	0.3555	0.078777	0.51123	1.8994 ↑	0.92558
DAG160/161	0.056302	0.43372	0.81457 ↓	-0.29588	0.078895	0.51123	0.81287 ↓	-0.2989
TAG450-FA140	0.12427	0.54053	0.87457 ↓	-0.19335	0.079531	0.51123	0.8363 ↓	-0.2579
TAG543-FA181	0.40341	0.77932	1.2773 ↑	0.35305	0.080118	0.51123	1.6481 ↑	0.72084
TAG561-FA160	0.56273	0.86967	1.1499 ↑	0.20149	0.080686	0.51123	1.7216 ↑	0.78378
TAG440-FA160	0.55261	0.86029	0.95767 ↓	-0.062399	0.081305	0.51123	0.85617 ↓	-0.22404
TAG420-FA120	0.16756	0.57186	0.86518 ↓	-0.20893	0.081621	0.51123	0.81991 ↓	-0.28646
FFA160	0.57724	0.86994	0.96068 ↓	-0.057867	0.086666	0.51123	0.77382 ↓	-0.36994
TAG463-FA141	0.013651	0.31041	0.7454 ↓	-0.42391	0.0876	0.51123	0.80883 ↓	-0.30609
TAG460-FA160	0.32343	0.73885	0.93515 ↓	-0.096726	0.088169	0.51123	0.89055 ↓	-0.16723
TAG563-FA201	0.32289	0.73885	1.2545 ↑	0.32714	0.088652	0.51123	1.5019 ↑	0.58674
TAG567-FA180	0.34018	0.74524	1.0534 ↑	0.075029	0.089157	0.51123	0.80431 ↓	-0.31417
TAG470-FA160	0.17661	0.57755	0.89778 ↓	-0.15557	0.089338	0.51123	0.858 ↓	-0.22094
TAG510-FA160	0.47973	0.82568	1.0556 ↑	0.078045	0.089645	0.51123	0.85264 ↓	-0.22999
TAG471-FA170	0.35119	0.74991	0.90002 ↓	-0.15197	0.093143	0.51123	0.75764 ↓	-0.40042
TAG523-FA160	0.64541	0.90045	1.516 ↑	0.60026	0.093387	0.51123	2.3168 ↑	1.2122
TAG527-FA160	0.53424	0.84948	1.0537 ↑	0.0754	0.093721	0.51123	0.8449 ↓	-0.24314
TAG543-FA180	0.25778	0.65998	1.5472 ↑	0.62965	0.094652	0.51123	1.7762 ↑	0.8288
TAG512-FA150	0.058169	0.43372	1.3139 ↑	0.3939	0.095051	0.51123	1.6226 ↑	0.69833

TAG440-FA120	0.51162	0.84511	0.93894 ↓	-0.090897	0.096095	0.51123	0.86704 ↓	-0.20584
TAG460-FA140	0.40778	0.78183	0.94462 ↓	-0.082199	0.097406	0.51123	0.89054 ↓	-0.16725
TAG523-FA181	0.43564	0.8085	1.4977 ↑	0.58272	0.097649	0.51123	2.3174 ↑	1.2125
OROTATE_neg_1	0.029517	0.31041	0.56329 ↓	-0.82805	0.098946	0.51123	0.82506 ↓	-0.27743
TAG502-FA160	0.46864	0.82568	1.3603 ↑	0.44395	0.099306	0.51123	2.0249 ↑	1.0179
METHIONINE_pos_1	0.19551	0.5978	0.67596 ↓	-0.56499	0.09947	0.51123	0.87338 ↓	-0.19532
TAG521-FA180	0.044416	0.39704	1.4338 ↑	0.51981	0.099477	0.51123	1.7988 ↑	0.84704
TAG441-FA161	0.38078	0.76877	0.92376 ↓	-0.11441	0.099762	0.51123	0.77616 ↓	-0.36558
TAG530-FA160	0.58236	0.86994	1.0153 ↑	0.021952	0.099865	0.51123	0.81759 ↓	-0.29055
TAG462-FA141	0.16192	0.56567	0.87627 ↓	-0.19056	0.10104	0.51123	0.83889 ↓	-0.25345
TAG543-FA182	0.27194	0.66919	1.7477 ↑	0.80546	0.10855	0.54277	2.1749 ↑	1.121
1-METHYLADENOSINE_pos_1	0.95405	0.98616	1.0038 ↑	0.0054521	0.11051	0.54488	0.88409 ↓	-0.17773
TAG450-FA160	0.07772	0.48415	0.86181 ↓	-0.21456	0.11334	0.54488	0.87202 ↓	-0.19756
TAG523-FA182	0.58597	0.86994	1.5435 ↑	0.62623	0.11461	0.54488	2.3677 ↑	1.2435
TAG400-FA120	0.15124	0.56567	0.84478 ↓	-0.24335	0.11567	0.54488	0.80705 ↓	-0.30928
TAG544-FA181	0.84208	0.97251	1.456 ↑	0.542	0.11574	0.54488	1.6461 ↑	0.71905
TAG564-FA201	0.025278	0.31041	1.836 ↑	0.87659	0.11729	0.54488	1.7229 ↑	0.78486
TAG483-FA161	0.44006	0.81316	0.89191 ↓	-0.16502	0.11822	0.54488	0.81467 ↓	-0.29571
TAG491-FA140	0.23934	0.63872	0.90596 ↓	-0.14249	0.12557	0.54488	0.83057 ↓	-0.26782
TAG490-FA170	0.14341	0.56567	0.90125 ↓	-0.15	0.12574	0.54488	0.88723 ↓	-0.17261
TAG566-FA160	0.029945	0.31041	1.1645 ↑	0.2197	0.12709	0.54488	1.1784 ↑	0.23682
TAG546-FA160	0.15633	0.56567	1.4375 ↑	0.52353	0.12894	0.54488	1.3931 ↑	0.47829
TAG523-FA180	0.00094722	0.080514	2.0347 ↑	1.0248	0.13073	0.54488	1.9901 ↑	0.99281
DAG181/181	0.46007	0.8244	0.95417 ↓	-0.067689	0.13165	0.54488	0.8853 ↓	-0.17576
TAG482-FA182	0.14289	0.56567	1.086 ↑	0.11902	0.13295	0.54488	1.2154 ↑	0.28149
TAG514-FA182	0.092202	0.49873	1.2687 ↑	0.34336	0.13317	0.54488	1.4481 ↑	0.53416
TAG512-FA170	0.97757	0.98616	1.0307 ↑	0.043598	0.13545	0.54488	1.2838 ↑	0.36043
TAG583-FA181	0.17901	0.57997	1.3521 ↑	0.43522	0.13651	0.54488	1.7753 ↑	0.82806
CE181	0.21546	0.60821	1.1016 ↑	0.13958	0.1371	0.54488	3.1239 ↑	1.6433
TAG420-FA140	0.2368	0.63696	0.89829 ↓	-0.15474	0.1372	0.54488	0.86447 ↓	-0.21011
TAG441-FA140	0.39416	0.7769	0.94799 ↓	-0.077058	0.13836	0.54488	0.80996 ↓	-0.30408
TAG512-FA182	0.05491	0.43372	1.4509 ↑	0.53692	0.13866	0.54488	1.6209 ↑	0.69681
TAG544-FA182	0.86015	0.97815	1.5725 ↑	0.6531	0.13922	0.54488	1.7688 ↑	0.82277
TAG524-FA181	0.34835	0.74884	1.3959 ↑	0.4812	0.13967	0.54488	1.8136 ↑	0.85883
TAG441-FA120	0.69837	0.91456	0.96871 ↓	-0.04586	0.13975	0.54488	0.79348 ↓	-0.33374
TAG422-FA120	0.027026	0.31041	0.47752 ↓	-1.0664	0.14129	0.54589	1.1806 ↑	0.2395
TAG421-FA140	0.306	0.71851	0.92833 ↓	-0.10729	0.14438	0.54709	0.89936 ↓	-0.15303
TAG490-FA160	0.17582	0.57755	0.91039 ↓	-0.13544	0.14639	0.54709	0.90099 ↓	-0.15041
TAG503-FA182	0.15987	0.56567	1.2267 ↑	0.29482	0.14671	0.54709	1.6514 ↑	0.72371

TAG400-FA140	0.26281	0.66099	0.86997 ↓	-0.20096	0.14685	0.54709	0.8422 ↓	-0.24777
TAG462-FA161	0.48582	0.8292	0.92269 ↓	-0.11609	0.14827	0.54709	0.79185 ↓	-0.3367
TAG567-FA160	0.090767	0.49873	1.0196 ↑	0.027989	0.15142	0.54709	0.71542 ↓	-0.48314
TAG510-FA170	0.58747	0.86994	0.95229 ↓	-0.070533	0.1515	0.54709	0.91159 ↓	-0.13354
TAG521-FA181	0.086549	0.49707	1.3155 ↑	0.39564	0.153	0.54709	1.681 ↑	0.74929
METHYL PHENYLACETATE_neg_3	0.21609	0.60821	0.3492 ↓	-1.5179	0.15329	0.54709	1.4383 ↑	0.52435
TAG513-FA150	0.66644	0.907	1.5715 ↑	0.65217	0.15602	0.54709	2.3248 ↑	1.2171
TAG522-FA182	0.094908	0.49873	1.6914 ↑	0.75821	0.15675	0.54709	2.0697 ↑	1.0494
TAG505-FA160	0.35824	0.75373	1.0472 ↑	0.066475	0.15705	0.54709	0.77559 ↓	-0.36664
DAG140/181	0.22762	0.62122	0.8602 ↓	-0.21726	0.16027	0.55378	0.87106 ↓	-0.19916
ASPARTATE_pos_3	0.86058	0.97815	0.80378 ↓	-0.31513	0.16633	0.56375	1.1634 ↑	0.21831
TAG502-FA161	0.73726	0.92978	0.98841 ↓	-0.016814	0.1674	0.56375	1.3533 ↑	0.43645
TAG587-FA160	0.79411	0.94763	0.9954 ↓	-0.0066447	0.16822	0.56375	0.86991 ↓	-0.20106
TAG551-FA160	0.87839	0.97842	0.77047 ↓	-0.37619	0.16961	0.56375	1.2429 ↑	0.31371
AMINOADIPATE_pos_1	0.40873	0.78183	0.95384 ↓	-0.068184	0.16979	0.56375	0.79892 ↓	-0.32387
TAG520-FA200	0.63315	0.90045	0.96393 ↓	-0.053005	0.17261	0.56869	0.71101 ↓	-0.49207
TAG461-FA161	0.53496	0.84948	0.91943 ↓	-0.1212	0.18008	0.57493	0.81224 ↓	-0.30003
TAG525-FA225	0.19282	0.59695	0.87785 ↓	-0.18796	0.18115	0.57493	0.86243 ↓	-0.21352
DAG161/181	0.05969	0.4362	0.76049 ↓	-0.395	0.18246	0.57493	0.87441 ↓	-0.19362
TAG471-FA140	0.2772	0.6714	0.89647 ↓	-0.15767	0.1837	0.57493	0.82113 ↓	-0.28432
TAG514-FA161	0.64223	0.90045	0.89902 ↓	-0.15358	0.18388	0.57493	0.88785 ↓	-0.17161
LCER160	0.085212	0.4961	0.81414 ↓	-0.29664	0.18463	0.57493	0.8489 ↓	-0.23634
FFA181	0.5127	0.84511	0.95224 ↓	-0.070602	0.1849	0.57493	0.61241 ↓	-0.70742
CYSTEINE_neg_5	0.76056	0.94709	0.97633 ↓	-0.034558	0.18533	0.57493	1.6096 ↑	0.68671
TAG526-FA181	0.64622	0.90045	1.054 ↑	0.075816	0.18929	0.58163	0.81094 ↓	-0.30233
DAG160/204	0.94893	0.98616	0.79754 ↓	-0.32638	0.19075	0.58163	1.5305 ↑	0.61403
TAG460-FA120	0.71697	0.91744	0.97097 ↓	-0.042502	0.19159	0.58163	0.91869 ↓	-0.12235
FFA180	0.87314	0.97842	1.0123 ↑	0.01769	0.19568	0.58613	0.82324 ↓	-0.28062
BIOTIN_pos_4	0.97581	0.98616	0.88633 ↓	-0.17408	0.1997	0.58613	1.0757 ↑	0.10529
TAG463-FA161	0.27635	0.6714	0.7953 ↓	-0.33043	0.20027	0.58613	0.91553 ↓	-0.12732
TAG501-FA180	0.051117	0.42597	1.215 ↑	0.28091	0.20124	0.58613	1.2853 ↑	0.36216
MALONYL-COA_pos_1	0.044842	0.39704	1.4725 ↑	0.55825	0.20403	0.58613	1.3494 ↑	0.43234
TAG480-FA160	0.40129	0.77932	0.95457 ↓	-0.067072	0.20445	0.58613	0.87931 ↓	-0.18556
TAG503-FA181	0.13277	0.55322	1.1018 ↑	0.13984	0.20557	0.58613	1.243 ↑	0.31386
TAG582-FA181	0.017103	0.31041	1.79 ↑	0.83999	0.20702	0.58613	1.8262 ↑	0.86886
TAG513-FA182	0.028543	0.31041	1.5139 ↑	0.59822	0.20731	0.58613	1.7027 ↑	0.76784
TAG442-FA161	0.17666	0.57755	0.84407 ↓	-0.24457	0.2078	0.58613	0.81803 ↓	-0.28977
TAG512-FA160	0.8887	0.97842	1.0116 ↑	0.016688	0.20825	0.58613	0.8541 ↓	-0.22753
TAG492-FA150	0.76823	0.94709	1.0374 ↑	0.053031	0.21205	0.58648	1.1439 ↑	0.19391

TAG502-FA181	0.1304	0.5487	1.1464 ↑	0.1971	0.21214	0.58648	1.4728 ↑	0.55858
TAG511-FA150	0.86535	0.97815	1.0279 ↑	0.039652	0.21327	0.58648	1.2792 ↑	0.3552
TAG461-FA141	0.01166	0.30971	0.78077 ↓	-0.35702	0.21516	0.58648	0.9084 ↓	-0.1386
CER160	0.78055	0.94763	1.2038 ↑	0.26765	0.21527	0.58648	2.5575 ↑	1.3547
MESACONIC ACID_pos_1	0.31038	0.72361	0.72214 ↓	-0.46964	0.22154	0.59451	1.3918 ↑	0.4769
TAG524-FA160	0.71225	0.91456	1.4319 ↑	0.51795	0.22167	0.59451	1.4725 ↑	0.55825
TAG532-FA170	0.57453	0.86994	0.95192 ↓	-0.071082	0.22242	0.59451	1.2001 ↑	0.2631
DAG160/160	0.95751	0.98616	1.0093 ↑	0.01331	0.2249	0.59501	0.78332 ↓	-0.35233
TAG511-FA160	0.15713	0.56567	0.92962 ↓	-0.10528	0.22746	0.59501	1.1191 ↑	0.16229
TAG504-FA182	0.3792	0.76877	1.1634 ↑	0.21832	0.228	0.59501	1.3775 ↑	0.46202
TAG544-FA183	0.39667	0.7769	1.4385 ↑	0.52456	0.22821	0.59501	1.2807 ↑	0.35699
TAG525-FA182	0.95324	0.98616	1.4457 ↑	0.53178	0.23058	0.59754	1.5659 ↑	0.64702
TAG568-FA204	0.53429	0.84948	1.0658 ↑	0.091934	0.23564	0.59786	0.8249 ↓	-0.27771
TAG521-FA201	0.71884	0.91744	0.90928 ↓	-0.13721	0.23596	0.59786	1.1305 ↑	0.17697
TAG480-FA180	0.86538	0.97815	0.98431 ↓	-0.022812	0.23597	0.59786	0.93403 ↓	-0.098464
TAG533-FA182	0.21503	0.60821	1.2881 ↑	0.36524	0.2367	0.59786	1.4112 ↑	0.49689
DAG140/182	0.47573	0.82568	0.79373 ↓	-0.33328	0.23774	0.59786	0.84782 ↓	-0.23817
TAG563-FA202	0.89566	0.97842	1.0403 ↑	0.056987	0.24028	0.60071	0.82273 ↓	-0.2815
SM240	0.00055593	0.078757	2.3847 ↑	1.2538	0.24343	0.60339	0.76802 ↓	-0.38078
TAG534-FA182	0.51082	0.84511	1.0597 ↑	0.083591	0.24612	0.60339	0.84057 ↓	-0.25056
TAG524-FA183	0.92736	0.98532	1.3083 ↑	0.38774	0.24638	0.60339	1.563 ↑	0.64428
TAG555-FA181	0.56027	0.86903	1.0526 ↑	0.07391	0.2505	0.60339	0.84011 ↓	-0.25135
TAG471-FA160	0.34368	0.74884	0.89113 ↓	-0.1663	0.2512	0.60339	0.85433 ↓	-0.22713
TAG503-FA160	0.2125	0.60821	1.4502 ↑	0.53626	0.25124	0.60339	1.9573 ↑	0.96888
TAG451-FA150	0.33584	0.7434	0.91097 ↓	-0.13453	0.25515	0.60339	0.81104 ↓	-0.30215
TAG462-FA140	0.70104	0.91456	0.93964 ↓	-0.089813	0.25521	0.60339	0.86196 ↓	-0.2143
TAG540-FA160	0.22598	0.62122	1.0593 ↑	0.08313	0.25547	0.60339	0.73096 ↓	-0.45213
ATROLACTIC ACID_neg_2	0.1239	0.54053	0.57065 ↓	-0.80932	0.25739	0.60339	1.354 ↑	0.43722
PYRIDOXAMINE_pos_1	0.0088346	0.30971	1.5529 ↑	0.63499	0.25805	0.60339	0.67669 ↓	-0.56344
ALLANTOIN_neg_2	0.70842	0.91456	1.0274 ↑	0.038955	0.25839	0.60339	0.95917 ↓	-0.060144
DAG180/182	0.18248	0.58265	0.86007 ↓	-0.21747	0.26083	0.60501	1.2403 ↑	0.31074
ARGININOSUCCINIC ACID_neg_2	0.018969	0.31041	0.56015 ↓	-0.83611	0.26194	0.60501	1.0459 ↑	0.064713
TAG524-FA182	0.7901	0.94763	1.4869 ↑	0.57232	0.26677	0.61285	1.6211 ↑	0.69698
TAG565-FA225	0.93936	0.98616	0.96339 ↓	-0.053806	0.27037	0.61349	0.8715 ↓	-0.19842
N-ACETYLORNITHINE_neg_3	0.1401	0.56567	0.8215 ↓	-0.28367	0.2725	0.61349	1.2297 ↑	0.29826
TAG490-FA150	0.19021	0.59441	0.92362 ↓	-0.11462	0.27256	0.61349	0.92851 ↓	-0.10702
TAG567-FA226	0.0022488	0.13654	1.751 ↑	0.80821	0.27533	0.61349	1.5023 ↑	0.58719
TAG482-FA141	0.20206	0.60053	0.88418 ↓	-0.17759	0.27826	0.61349	1.0857 ↑	0.11861
PHENYLPROPIOLIC ACID_neg_1	0.0072568	0.30971	1.515 ↑	0.59929	0.28064	0.61349	0.80911 ↓	-0.3056

TAG543-FA202	0.84866	0.97745	1.047 ↑	0.066261	0.28312	0.61349	0.82916 ↓	-0.27028
TAG512-FA181	0.41023	0.78183	1.059 ↑	0.082721	0.2854	0.61349	0.92805 ↓	-0.10773
TAG491-FA161	0.54609	0.85327	0.9111 ↓	-0.13431	0.28683	0.61349	0.82722 ↓	-0.27365
TAG510-FA180	0.48195	0.82593	1.0455 ↑	0.064151	0.28777	0.61349	0.95243 ↓	-0.070311
TAG491-FA181	0.90756	0.98112	0.99645 ↓	-0.0051269	0.28787	0.61349	1.0554 ↑	0.07783
GLUCOSE 1-PHOSPHATE_pos_2	0.24806	0.65077	0.64373 ↓	-0.63547	0.28814	0.61349	0.87181 ↓	-0.19791
TAG420-FA160	0.78394	0.94763	0.95459 ↓	-0.067047	0.28822	0.61349	0.91282 ↓	-0.1316
TAG480-FA140	0.64186	0.90045	0.9675 ↓	-0.047673	0.28909	0.61349	0.94683 ↓	-0.078817
TAG421-FA120	0.16238	0.56567	0.88133 ↓	-0.18225	0.28929	0.61349	0.79785 ↓	-0.3258
TAG540-FA180	0.7982	0.94763	0.97978 ↓	-0.029467	0.29073	0.61349	0.8407 ↓	-0.25033
TAG564-FA202	0.7689	0.94709	1.046 ↑	0.064925	0.29159	0.61349	0.83655 ↓	-0.25748
TAG511-FA181	0.1139	0.53256	1.1563 ↑	0.20952	0.2941	0.61572	1.1548 ↑	0.20769
TAG564-FA182	0.28468	0.68283	1.7076 ↑	0.77201	0.29827	0.61602	2.031 ↑	1.0222
TAG502-FA140	0.52484	0.84948	1.0893 ↑	0.12339	0.30014	0.61602	1.4279 ↑	0.51393
TAG505-FA205	0.388	0.77418	0.93916 ↓	-0.090557	0.30453	0.61602	0.90119 ↓	-0.1501
TAG460-FA180	0.80944	0.95031	1.0153 ↑	0.02188	0.3052	0.61602	0.93718 ↓	-0.093602
TAG566-FA202	0.15339	0.56567	1.123 ↑	0.16731	0.30634	0.61602	0.85328 ↓	-0.2289
TAG532-FA181	0.72818	0.92381	1.0322 ↑	0.045672	0.30722	0.61602	0.88407 ↓	-0.17776
TAG501-FA161	0.68259	0.91227	1.0057 ↑	0.0082388	0.3074	0.61602	1.1219 ↑	0.16594
TAG501-FA181	0.50505	0.84511	1.057 ↑	0.079982	0.30893	0.61602	1.2818 ↑	0.3582
TAG520-FA160	0.80047	0.94763	0.98749 ↓	-0.01816	0.31051	0.61602	0.845 ↓	-0.24298
TAG565-FA202	0.66602	0.907	1.0528 ↑	0.074216	0.31174	0.61602	0.83237 ↓	-0.26471
L-CARNITINE_pos_1	0.050559	0.42597	0.47649 ↓	-1.0695	0.31276	0.61602	0.8066 ↓	-0.31007
4-AMINOBENZOIC ACID_pos_1	0.45589	0.8244	1.2684 ↑	0.34296	0.31555	0.61602	0.84079 ↓	-0.25019
TAG562-FA160	0.20659	0.60554	1.2289 ↑	0.29739	0.31699	0.61602	1.5384 ↑	0.62145
TAG545-FA182	0.7231	0.92012	1.4836 ↑	0.56915	0.31743	0.61602	1.2358 ↑	0.30544
BUTYRYL-COA_pos_1	0.67701	0.90766	0.62669 ↓	-0.67418	0.31865	0.61602	1.5721 ↑	0.65272
3-METHYLAMINO-L-ALANINE_pos_2	0.80364	0.9483	0.98248 ↓	-0.025498	0.31898	0.61602	0.82363 ↓	-0.27993
TAG545-FA225	0.33535	0.7434	0.91146 ↓	-0.13374	0.32004	0.61602	0.8618 ↓	-0.21458
TAG471-FA150	0.53694	0.84948	0.90172 ↓	-0.14926	0.32114	0.61602	0.85871 ↓	-0.21975
TAG442-FA120	0.66929	0.907	0.97794 ↓	-0.032176	0.32264	0.61602	0.84422 ↓	-0.24431
TAG492-FA160	0.91879	0.98112	1.0194 ↑	0.027666	0.32323	0.61602	1.1427 ↑	0.19249
TAG492-FA161	0.39587	0.7769	0.88192 ↓	-0.18127	0.3269	0.61915	0.83008 ↓	-0.26868
TAG555-FA182	0.94592	0.98616	1.0753 ↑	0.10467	0.32778	0.61915	0.84334 ↓	-0.24581
ACETYLPHOSPHATE_pos_1	0.37313	0.76877	1.2038 ↑	0.26759	0.33413	0.62833	0.86084 ↓	-0.21619
ACETYL-COA_pos_1	0.18371	0.58265	0.96733 ↓	-0.047921	0.33706	0.63106	1.2708 ↑	0.34569
TAG472-FA140	0.8595	0.97815	0.95327 ↓	-0.069049	0.33905	0.63201	0.81929 ↓	-0.28756
TAG545-FA181	0.67418	0.907	1.379 ↑	0.46367	0.34513	0.63967	1.1566 ↑	0.20987
HCER201	0.75127	0.94186	0.66548 ↓	-0.58754	0.34617	0.63967	0.69376 ↓	-0.52749

TAG481-FA160	0.523	0.84948	1.0314 ↑	0.044628	0.35175	0.64717	1.1075 ↑	0.14732
TAG542-FA201	0.16234	0.56567	1.1878 ↑	0.2483	0.35452	0.64945	1.3979 ↑	0.4833
TAG491-FA150	0.87686	0.97842	1.0268 ↑	0.038208	0.3573	0.64995	1.1062 ↑	0.14557
TAG482-FA161	0.52944	0.84948	0.88934 ↓	-0.16919	0.36019	0.64995	0.84707 ↓	-0.23945
ADP_pos_1	0.31413	0.72556	0.98866 ↓	-0.016453	0.36076	0.64995	1.2042 ↑	0.26805
TAG472-FA182	0.69655	0.91456	1.0018 ↑	0.0025886	0.36091	0.64995	0.87701 ↓	-0.18934
MEVALONOLACTONE_pos_2	0.95592	0.98616	0.84707 ↓	-0.23945	0.36916	0.662	1.0743 ↑	0.10333
CE182	0.49224	0.83477	0.45996 ↓	-1.1204	0.37856	0.676	7.2083 ↑	2.8497
GERANYL-PP_-HPO3_neg_2	0.79502	0.94763	0.93578 ↓	-0.095761	0.39315	0.69732	1.195 ↑	0.25703
N-ACETYLALANINE_pos_2	0.76522	0.94709	1.0597 ↑	0.083667	0.39384	0.69732	1.5383 ↑	0.62133
BUTYRYL-COA_neg_2	0.54482	0.85327	0.83118 ↓	-0.26677	0.39622	0.69732	1.5296 ↑	0.61315
TAG554-FA181	0.88967	0.97842	1.0207 ↑	0.029494	0.39789	0.69732	0.83313 ↓	-0.26339
TAG483-FA181	0.87504	0.97842	0.99613 ↓	-0.0055985	0.40106	0.69732	1.1235 ↑	0.16798
SYMMETRIC DIMETHYLARGININE_pos_1	0.97782	0.98616	1.0331 ↑	0.046969	0.40351	0.69732	1.3991 ↑	0.48445
TAG566-FA225	0.11448	0.53256	1.1163 ↑	0.15867	0.40409	0.69732	0.92011 ↓	-0.12012
TAG492-FA182	0.34887	0.74884	1.1291 ↑	0.17523	0.40659	0.69732	1.2276 ↑	0.29579
DAG181/182	0.65256	0.90045	0.98527 ↓	-0.021403	0.408	0.69732	0.91303 ↓	-0.13127
TAG503-FA161	0.95025	0.98616	1.0103 ↑	0.014714	0.40959	0.69732	1.236 ↑	0.30568
TAG491-FA170	0.61829	0.89076	0.92131 ↓	-0.11824	0.40992	0.69732	0.82326 ↓	-0.28057
TAG490-FA180	0.9478	0.98616	0.9946 ↓	-0.0078077	0.41071	0.69732	0.95922 ↓	-0.060069
TAG587-FA225	0.069515	0.46162	1.0147 ↑	0.021117	0.41213	0.69732	0.6592 ↓	-0.60122
TAG500-FA180	0.90096	0.9793	1.0117 ↑	0.016735	0.41677	0.69732	0.87971 ↓	-0.1849
TAG461-FA140	0.66747	0.907	0.95063 ↓	-0.073038	0.4177	0.69732	0.88641 ↓	-0.17395
TAG421-FA161	0.25977	0.66099	0.92173 ↓	-0.11759	0.41804	0.69732	0.80219 ↓	-0.31798
TAG504-FA160	0.019008	0.31041	1.3096 ↑	0.38912	0.42106	0.69732	1.2199 ↑	0.28675
TAG440-FA180	0.78943	0.94763	1.0235 ↑	0.033449	0.42303	0.69732	0.93849 ↓	-0.09159
TAG400-FA160	0.97532	0.98616	0.98532 ↓	-0.021331	0.42313	0.69732	0.94348 ↓	-0.083935
TAG442-FA140	0.014235	0.31041	0.60969 ↓	-0.71385	0.42332	0.69732	1.0472 ↑	0.066497
SM220	0.000026818	0.011398	2.3142 ↑	1.2105	0.43064	0.70664	0.992 ↓	-0.011584
TAG520-FA180	0.8194	0.95671	0.99094 ↓	-0.013133	0.4377	0.71546	0.86288 ↓	-0.21277
TAG462-FA181	0.71012	0.91456	1.0885 ↑	0.12234	0.44068	0.71759	0.97103 ↓	-0.042407
TAG568-FA205	0.68749	0.91456	0.98449 ↓	-0.022552	0.4535	0.73564	0.8021 ↓	-0.31815
TAG481-FA161	0.67251	0.907	0.93354 ↓	-0.099211	0.46045	0.74257	0.89854 ↓	-0.15435
TAG483-FA160	0.78927	0.94763	0.97077 ↓	-0.042794	0.46126	0.74257	1.2385 ↑	0.30865
TAG472-FA150	0.79417	0.94763	0.90351 ↓	-0.14638	0.46469	0.74525	0.86258 ↓	-0.21327
O-ACETYLSERINE_pos_3	0.53631	0.84948	0.98292 ↓	-0.024858	0.47036	0.75091	1.036 ↑	0.051046
L-ACETYLCARNITINE_pos_1	0.97146	0.98616	0.90076 ↓	-0.15078	0.47175	0.75091	1.1539 ↑	0.20648
TAG544-FA180	0.93677	0.98616	1.3423 ↑	0.42472	0.48381	0.76724	1.1989 ↑	0.26175
CE205	0.67438	0.907	1.0556 ↑	0.078076	0.48784	0.77076	2.468 ↑	1.3033

TAG563-FA181	0.24777	0.65077	1.0933 ↑	0.12865	0.49253	0.77117	0.89035 ↓	-0.16755
TAG441-FA160	0.89701	0.97842	1.0221 ↑	0.031565	0.49327	0.77117	0.97455 ↓	-0.037189
TAG501-FA140	0.12524	0.54053	1.1976 ↑	0.26013	0.49512	0.77117	1.1505 ↑	0.20232
TAG461-FA180	0.64197	0.90045	1.0694 ↑	0.096841	0.49536	0.77117	0.99071 ↓	-0.013471
TAG463-FA181	0.20021	0.60053	1.1664 ↑	0.22205	0.5045	0.77678	1.1301 ↑	0.17644
TAG482-FA160	0.89564	0.97842	0.98286 ↓	-0.024937	0.50595	0.77678	1.0743 ↑	0.10335
TAG481-FA180	0.47684	0.82568	1.0509 ↑	0.071575	0.50626	0.77678	1.0261 ↑	0.037211
PIPECOLATE_pos_3	0.45484	0.8244	0.75461 ↓	-0.40619	0.50901	0.77678	1.1401 ↑	0.18921
TAG441-FA181	0.52323	0.84948	1.1492 ↑	0.20067	0.51007	0.77678	0.98086 ↓	-0.027881
TAG568-FA160	0.070961	0.46397	0.74672 ↓	-0.42136	0.51041	0.77678	1.1541 ↑	0.20675
TAG511-FA180	0.023828	0.31041	1.2331 ↑	0.30233	0.51322	0.77678	1.1463 ↑	0.19701
TAG587-FA180	0.020808	0.31041	1.1229 ↑	0.16717	0.51359	0.77678	0.74815 ↓	-0.4186
TAG553-FA181	0.53767	0.84948	0.97485 ↓	-0.036742	0.51715	0.77818	0.89034 ↓	-0.16758
DAG160/182	0.1716	0.57755	0.91397 ↓	-0.12978	0.51817	0.77818	0.92312 ↓	-0.11542
TAG541-FA200	0.64339	0.90045	1.0262 ↑	0.037378	0.52073	0.77926	1.5291 ↑	0.61264
TAG421-FA160	0.70207	0.91456	1.0841 ↑	0.11643	0.52698	0.78585	1.0018 ↑	0.002578
TAG503-FA141	0.71228	0.91456	0.98898 ↓	-0.015987	0.53325	0.79242	1.1742 ↑	0.23171
CHOLINE_pos_1	0.075417	0.47839	0.42092 ↓	-1.2484	0.53639	0.7943	1.1178 ↑	0.16063
CE150	0.33297	0.7434	0.92017 ↓	-0.12003	0.54231	0.79859	0.89271 ↓	-0.16373
SM180	0.16819	0.57186	1.085 ↑	0.11765	0.54372	0.79859	0.87068 ↓	-0.19979
TAG481-FA140	0.2724	0.66919	1.0897 ↑	0.12389	0.54492	0.79859	1.1252 ↑	0.17016
TAG522-FA202	0.14703	0.56567	0.8583 ↓	-0.22044	0.5508	0.80195	1.075 ↑	0.10438
FFA204	0.13619	0.55653	0.81698 ↓	-0.29163	0.55099	0.80195	1.1324 ↑	0.17936
TAG513-FA161	0.0099529	0.30971	0.65199 ↓	-0.61708	0.56215	0.80548	0.9586 ↓	-0.060996
N-ACETYLGLUTAMINE_pos_2	0.28599	0.68283	0.84536 ↓	-0.24236	0.56231	0.80548	1.373 ↑	0.45735
CE160	0.78254	0.94763	1.0354 ↑	0.050257	0.56288	0.80548	1.1637 ↑	0.2187
TAG492-FA140	0.76742	0.94709	0.92926 ↓	-0.10585	0.56555	0.80548	0.99185 ↓	-0.0118
TAG461-FA181	0.59941	0.88149	1.0968 ↑	0.13326	0.56588	0.80548	0.98977 ↓	-0.01484
TAG492-FA181	0.5905	0.8714	0.91758 ↓	-0.12409	0.56661	0.80548	0.89933 ↓	-0.15308
TAG545-FA183	0.34659	0.74884	1.2682 ↑	0.34279	0.56824	0.80548	1.1347 ↑	0.18233
TAG561-FA181	0.23044	0.62381	1.0834 ↑	0.11555	0.57103	0.80548	0.95019 ↓	-0.073705
TAG562-FA180	0.2541	0.65849	1.2121 ↑	0.27746	0.57266	0.80548	1.2405 ↑	0.31096
SPERMIDINE_pos_2	0.91556	0.98112	1.0182 ↑	0.026063	0.57425	0.80548	0.80689 ↓	-0.30956
TAG568-FA161	0.098253	0.50224	1.1499 ↑	0.20157	0.57588	0.80548	0.95821 ↓	-0.061584
TAG493-FA161	0.64992	0.90045	0.92193 ↓	-0.11727	0.57616	0.80548	0.8708 ↓	-0.19959
TAG512-FA161	0.0087817	0.30971	0.65627 ↓	-0.60763	0.58138	0.80582	0.9634 ↓	-0.053794
FFA201	0.22665	0.62122	0.88412 ↓	-0.17769	0.5815	0.80582	0.97363 ↓	-0.038558
TAG589-FA226	0.084658	0.4961	1.5366 ↑	0.61978	0.58279	0.80582	1.1854 ↑	0.24543
TAG545-FA205	0.5848	0.86994	0.85511 ↓	-0.22581	0.58398	0.80582	0.87671 ↓	-0.18983

2-HYDROXY-3-METHYLBUTYRIC ACID_p	0.88193	0.97842	0.90997 ↓	-0.13611	0.58959	0.81093	0.98204 ↓	-0.026145
TAG461-FA160	0.77327	0.94709	0.97917 ↓	-0.03037	0.59546	0.81611	0.93019 ↓	-0.10441
TAG526-FA160	0.041052	0.39652	1.3942 ↑	0.47939	0.60028	0.81611	1.1312 ↑	0.1779
TAG525-FA205	0.91301	0.98112	1.0737 ↑	0.10253	0.60055	0.81611	1.1991 ↑	0.262
TAG567-FA225	0.078603	0.48415	1.0431 ↑	0.060917	0.60104	0.81611	0.64328 ↓	-0.63649
URIDINE 5-DIPHOSPHATE_neg_2	0.0093381	0.30971	0.53983 ↓	-0.88943	0.60402	0.81754	1.0139 ↑	0.019965
FFA226	0.15121	0.56567	0.82832 ↓	-0.27174	0.60733	0.81942	0.93226 ↓	-0.1012
TAG500-FA160	0.97529	0.98616	1.0188 ↑	0.026836	0.61385	0.82559	0.90041 ↓	-0.15134
TAG442-FA182	0.61066	0.88757	1.0872 ↑	0.12065	0.61793	0.82846	1.0018 ↑	0.0025802
TAG521-FA161	0.029483	0.31041	0.79878 ↓	-0.32414	0.6202	0.82889	1.045 ↑	0.063456
TAG523-FA183	0.88563	0.97842	1.0674 ↑	0.094083	0.62582	0.8331	1.0394 ↑	0.055801
OXOGLUTARATE_neg_2	0.011436	0.30971	2.5043 ↑	1.3244	0.62728	0.8331	0.8907 ↓	-0.16699
TAG441-FA141	0.8703	0.97842	0.98426 ↓	-0.022886	0.63374	0.83653	0.88701 ↓	-0.17298
TAG532-FA160	0.16459	0.5687	0.76318 ↓	-0.3899	0.6338	0.83653	1.0517 ↑	0.072792
TAG461-FA120	0.7584	0.94709	1.0712 ↑	0.099253	0.63939	0.83739	0.94164 ↓	-0.086746
TAG493-FA160	0.52584	0.84948	0.78727 ↓	-0.34507	0.64158	0.83739	1.1777 ↑	0.23599
TAG491-FA160	0.975	0.98616	0.98836 ↓	-0.01689	0.64379	0.83739	1.0108 ↑	0.01548
TAG526-FA182	0.24046	0.63872	1.2349 ↑	0.30439	0.64426	0.83739	1.0194 ↑	0.027778
DAG181/204	0.028678	0.31041	0.67151 ↓	-0.57452	0.6443	0.83739	1.1242 ↑	0.16886
ASCORBATE_neg_2	0.10931	0.53251	0.73765 ↓	-0.439	0.64811	0.83978	0.79561 ↓	-0.32987
FFA241	0.56857	0.86994	0.93372 ↓	-0.098935	0.65594	0.84199	0.87944 ↓	-0.18535
TAG528-FA161	0.92287	0.983	0.90044 ↓	-0.15129	0.6581	0.84199	0.88331 ↓	-0.179
TAG482-FA140	0.29782	0.70319	1.0455 ↑	0.064199	0.65888	0.84199	1.0744 ↑	0.10353
DAG180/181	0.22802	0.62122	0.86148 ↓	-0.21511	0.66091	0.84199	0.97144 ↓	-0.041809
SM261	0.027958	0.31041	1.6345 ↑	0.70885	0.66214	0.84199	0.98469 ↓	-0.02226
NADPH_pos_1	0.84075	0.97251	0.95863 ↓	-0.060953	0.66289	0.84199	0.64243 ↓	-0.63839
4-IMIDAZOLEACETATE_pos_1	0.044438	0.39704	0.56138 ↓	-0.83296	0.66582	0.84199	1.1826 ↑	0.242
2-KETOHEXANOIC ACID_pos_2	0.91434	0.98112	0.8459 ↓	-0.24145	0.66653	0.84199	1.0091 ↑	0.013065
TAG482-FA181	0.98838	0.99072	0.9904 ↓	-0.013913	0.66765	0.84199	0.99014 ↓	-0.014298
TAG546-FA226	0.063156	0.44735	1.3306 ↑	0.41208	0.6716	0.84447	0.98521 ↓	-0.021498
TAG533-FA160	0.65256	0.90045	1.0493 ↑	0.069447	0.67793	0.8482	1.0233 ↑	0.033253
TAG563-FA182	0.90907	0.98112	1.2047 ↑	0.26869	0.67976	0.8482	1.4835 ↑	0.56898
UREIDOSUCCINIC ACID_neg_2	0.9715	0.98616	0.90964 ↓	-0.13663	0.68065	0.8482	1.3336 ↑	0.41533
FFA202	0.27142	0.66919	0.89649 ↓	-0.15763	0.68255	0.8482	0.98147 ↓	-0.02698
TAG542-FA202	0.51303	0.84511	1.0579 ↑	0.081239	0.68684	0.85104	0.95686 ↓	-0.063613
TAG531-FA180	0.73611	0.92978	1.0519 ↑	0.072976	0.69318	0.8564	1.0032 ↑	0.0045745
TAG462-FA120	0.33874	0.74524	1.1214 ↑	0.16526	0.70043	0.86285	0.92425 ↓	-0.11365
TAG462-FA160	0.050566	0.42597	0.79281 ↓	-0.33495	0.70541	0.86647	1.0145 ↑	0.02073
CE161	0.37957	0.76877	0.92525 ↓	-0.11209	0.71269	0.8704	0.92264 ↓	-0.11615

L-2-HYDROXYGLUTARIC ACID_neg_1	0.065294	0.44981	1.8861 ↑	0.91543	0.7127	0.8704	1.021 ↑	0.029987
TAG511-FA170	0.20138	0.60053	0.9041 ↓	-0.14544	0.71601	0.87194	0.96894 ↓	-0.04552
DCER260	0.61399	0.88757	0.99247 ↓	-0.010898	0.72066	0.87363	0.94206 ↓	-0.086111
N-GLYCYL-L-PROLINE_pos_1	0.77212	0.94709	1.0784 ↑	0.10892	0.72191	0.87363	1.0508 ↑	0.071493
TAG533-FA170	0.57017	0.86994	0.93801 ↓	-0.092332	0.72357	0.87363	1.0361 ↑	0.051148
PROPIONYL-COA_pos_1	0.10174	0.50868	1.3275 ↑	0.40873	0.72625	0.87426	1.1964 ↑	0.25874
TAG482-FA120	0.45151	0.8244	1.1034 ↑	0.14194	0.72821	0.87426	1.1659 ↑	0.22146
TAG552-FA181	0.31158	0.72361	0.83019 ↓	-0.26849	0.73222	0.8766	0.95948 ↓	-0.05967
BETAINE_pos_2	0.36864	0.76801	0.80015 ↓	-0.32166	0.74072	0.88202	0.96494 ↓	-0.051484
TAG546-FA182	0.4636	0.8244	1.4502 ↑	0.53629	0.74161	0.88202	0.90009 ↓	-0.15185
FFA203	0.16024	0.56567	0.84543 ↓	-0.24224	0.74297	0.88202	0.92849 ↓	-0.10704
L-DIHYDROOROTIC ACID_neg_1	0.33463	0.7434	0.72602 ↓	-0.46192	0.7574	0.89665	1.1545 ↑	0.20732
HCER160	0.11185	0.53256	0.71283 ↓	-0.48837	0.75954	0.89668	0.90171 ↓	-0.14926
MYOINOSITOL_neg_3	0.8055	0.9483	1.0603 ↑	0.084504	0.77309	0.90242	0.99934 ↓	-0.0009508
DAG161/182	0.025004	0.31041	0.59619 ↓	-0.74616	0.77343	0.90242	1.1024 ↑	0.14062
TAG483-FA120	0.01534	0.31041	1.3103 ↑	0.38988	0.77454	0.90242	1.1703 ↑	0.22683
TAG541-FA181	0.38284	0.76877	1.0802 ↑	0.1113	0.77722	0.90242	0.93592 ↓	-0.095539
TAG481-FA181	0.4587	0.8244	1.0729 ↑	0.10148	0.77948	0.90242	1.0798 ↑	0.11072
MESACONIC ACID_neg_1	0.66417	0.907	1.2123 ↑	0.27778	0.7813	0.90242	1.0882 ↑	0.12198
FFA205	0.29663	0.70319	0.8646 ↓	-0.2099	0.78298	0.90242	1.0814 ↑	0.11286
TAG525-FA181	0.21391	0.60821	1.089 ↑	0.12297	0.78531	0.90242	0.98068 ↓	-0.028144
CE140	0.13475	0.55602	1.1445 ↑	0.19471	0.78555	0.90242	0.88331 ↓	-0.17901
TAG421-FA181	0.57802	0.86994	1.1272 ↑	0.17268	0.78564	0.90242	1.0254 ↑	0.036238
TAG524-FA180	0.27804	0.6714	1.1183 ↑	0.16129	0.7949	0.90713	0.98499 ↓	-0.021819
TAG442-FA160	0.35597	0.75267	0.9091 ↓	-0.1375	0.79515	0.90713	1.0906 ↑	0.12517
N-ACETYLALANINE_neg_1	0.10636	0.5256	1.5384 ↑	0.62146	0.79614	0.90713	0.79986 ↓	-0.32218
TAG503-FA183	0.090845	0.49873	1.1542 ↑	0.20692	0.79981	0.90799	0.97278 ↓	-0.039807
SM241	0.024886	0.31041	1.4469 ↑	0.53298	0.80302	0.90799	0.86877 ↓	-0.20295
CE180	0.99508	0.99508	0.99321 ↓	-0.0098355	0.8033	0.90799	1.0885 ↑	0.12228
TAG543-FA183	0.054902	0.43372	0.80123 ↓	-0.31971	0.80832	0.91123	#N/A	#N/A
TAG546-FA181	0.50776	0.84511	1.2896 ↑	0.36696	0.81456	0.91215	0.92352 ↓	-0.11479
TAG442-FA181	0.39206	0.7769	1.1846 ↑	0.24442	0.81474	0.91215	1.052 ↑	0.073179
FFA141	0.25745	0.65998	0.86673 ↓	-0.20635	0.81691	0.91215	1.1933 ↑	0.25502
CE141	0.58211	0.86994	0.92034 ↓	-0.11976	0.81772	0.91215	0.858 ↓	-0.22096
TAG492-FA170	0.79824	0.94763	0.868 ↓	-0.20423	0.82557	0.91762	0.90462 ↓	-0.14461
CITRACONIC ACID_neg	0.18013	0.57997	1.3072 ↑	0.38653	0.83375	0.91762	0.96854 ↓	-0.046109
TAG481-FA141	0.38348	0.76877	0.86498 ↓	-0.20926	0.83465	0.91762	1.0145 ↑	0.020801
TAG483-FA141	0.26975	0.66919	0.85146 ↓	-0.23199	0.83585	0.91762	0.97642 ↓	-0.034432
TAG422-FA182	0.40259	0.77932	1.0994 ↑	0.13675	0.84062	0.91762	1.1811 ↑	0.24014

TAG534-FA170	0.47618	0.82568	0.86791 ↓	-0.20438	0.8409	0.91762	0.93618 ↓	-0.095137
INDOLEACRYLIC ACID_pos_1	0.42262	0.79475	1.2456 ↑	0.31686	0.84221	0.91762	0.71994 ↓	-0.47405
TAG545-FA180	0.64909	0.90045	1.3713 ↑	0.45555	0.84252	0.91762	0.8438 ↓	-0.24502
TAG483-FA140	0.7463	0.9384	1.047 ↑	0.066266	0.84449	0.91762	1.0838 ↑	0.11609
TAG502-FA141	0.38067	0.76877	1.0322 ↑	0.04573	0.84474	0.91762	1.0553 ↑	0.077621
TAG483-FA182	0.95053	0.98616	0.98316 ↓	-0.024499	0.84637	0.91762	0.96376 ↓	-0.053255
N-GLYCYL-L-PROLINE_neg_3	0.45341	0.8244	0.94472 ↓	-0.082041	0.85827	0.92449	0.81145 ↓	-0.30142
TAG544-FA224	0.12254	0.54053	0.81506 ↓	-0.29503	0.85942	0.92449	0.95867 ↓	-0.060887
TAG525-FA160	0.82445	0.95998	1.3736 ↑	0.45796	0.85997	0.92449	0.98816 ↓	-0.017177
RIBOFLAVIN_pos_1	0.41339	0.78434	1.0741 ↑	0.1031	0.86141	0.92449	1.173 ↑	0.23017
METANEPHRINE_pos_1	0.017648	0.31041	0.66907 ↓	-0.57978	0.86656	0.92767	1.0786 ↑	0.10913
XANTHURENIC ACID_pos_2	0.15476	0.56567	0.53441 ↓	-0.90397	0.8707	0.92977	0.92844 ↓	-0.10712
TAG525-FA183	0.46558	0.82447	1.2962 ↑	0.37424	0.88199	0.93946	1.0576 ↑	0.080828
TAG531-FA170	0.70568	0.91456	0.91366 ↓	-0.13027	0.88772	0.94188	0.97392 ↓	-0.038119
FFA182	0.49753	0.83908	0.93556 ↓	-0.096096	0.88869	0.94188	0.8759 ↓	-0.19116
XANTHOSINE_neg_1	0.57485	0.86994	1.1985 ↑	0.26127	0.89488	0.94421	1.2451 ↑	0.31631
TAG504-FA161	0.42209	0.79475	1.0322 ↑	0.04579	0.89551	0.94421	1.0601 ↑	0.084252
TAG546-FA183	0.57075	0.86994	1.3872 ↑	0.47219	0.89798	0.94421	0.8979 ↓	-0.15537
TAG481-FA120	0.47672	0.82568	1.0917 ↑	0.12653	0.89978	0.94421	1.0741 ↑	0.10308
TAG463-FA182	0.18526	0.58323	1.1217 ↑	0.16569	0.9053	0.94568	1.0156 ↑	0.022275
N-ACETYLPUTRESCINE_pos_1	0.19383	0.59695	0.69742 ↓	-0.51991	0.90563	0.94568	1.2942 ↑	0.37207
TAG587-FA182	0.0087084	0.30971	1.5551 ↑	0.63697	0.91122	0.94919	1.0224 ↑	0.031983
CE225	0.69799	0.91456	1.0866 ↑	0.11984	0.91506	0.95086	1.1123 ↑	0.15357
PYROPHOSPHATE_neg_3	0.022643	0.31041	0.40345 ↓	-1.3095	0.91967	0.95329	1.4951 ↑	0.58025
TAG493-FA182	0.83831	0.97251	0.94598 ↓	-0.080123	0.92189	0.95329	0.95878 ↓	-0.06073
2-PHOSPHOGLYCERATE_pos_4	0.91663	0.98112	0.94756 ↓	-0.077706	0.92599	0.9552	1.1011 ↑	0.13892
NORMETANEPHRINE_pos_1	0.54123	0.85194	1.0721 ↑	0.10042	0.93482	0.96198	1.0322 ↑	0.045779
FFA225	0.085045	0.4961	0.81424 ↓	-0.29648	0.94235	0.9662	1.0798 ↑	0.11078
TAG531-FA160	0.16096	0.56567	0.70428 ↓	-0.50577	0.94347	0.9662	0.98068 ↓	-0.028144
TAG547-FA182	0.36611	0.76648	1.4154 ↑	0.50119	0.94807	0.96858	0.81727 ↓	-0.29111
DAG181/226	0.15058	0.56567	0.7952 ↓	-0.3306	0.95211	0.97038	1.0357 ↑	0.050639
TAG547-FA183	0.44299	0.81502	1.4076 ↑	0.49324	0.96791	0.98412	0.84267 ↓	-0.24696
TAG589-FA181	0.47986	0.82568	1.0365 ↑	0.05174	0.97387	0.98781	0.99269 ↓	-0.010578
2-HYDROXY-3-METHYLBUTYRIC ACID_n	0.89784	0.97842	0.87792 ↓	-0.18783	0.98733	0.99881	0.86312 ↓	-0.21237
IMIDAZOLE_pos_1	0.95872	0.98616	0.89246 ↓	-0.16414	0.99114	0.99881	0.76898 ↓	-0.37899
INDOLE-3-CARBOXYLIC ACID_pos_3	0.64397	0.90045	1.336 ↑	0.41796	0.99571	0.99881	1.4863 ↑	0.57175
CE170	0.073825	0.47539	0.69734 ↓	-0.52007	0.99689	0.99881	1.0935 ↑	0.12892
TAG504-FA181	0.25189	0.65676	1.1136 ↑	0.15519	0.99873	0.99881	1.0009 ↑	0.0012678
L-ORNITHINE_pos_1	0.12934	0.5487	1.7142 ↑	0.77756	0.99881	0.99881	1.0104 ↑	0.014863
